# Steric Influence on Reactions of Benzyl Potassium Species with CO

**DOI:** 10.1002/asia.202101127

**Published:** 2021-10-13

**Authors:** Tongtong Wang, Maotong Xu, Andrew R. Jupp, Zheng‐Wang Qu, Stefan Grimme, Douglas W. Stephan

**Affiliations:** ^1^ Department of Chemistry University of Toronto 80 St. George St. Toronto Ontario M5S3H6 Canada; ^2^ School of Chemistry Faculty of Chemical Environmental and Biological Science and Technology Dalian University of Technology (P. R. China); ^3^ Mulliken Center for Theoretical Chemistry University of Bonn Beringstr. 4 53115 Bonn Germany

**Keywords:** C−C bond formation, carbon monoxide, radical, carbene, tropolone

## Abstract

Reactions of benzyl potassium species with CO are shown to proceed via transient carbene‐like intermediates that can undergo either dimerization or further CO propagation. In a sterically unhindered case, formal dimerization of the carbene is the dominant reaction pathway, as evidenced by the isolation of ((Ph_3_SiO)(PhCH_2_)C)_2_ **2** and PhCH_2_C(O)CH(OH)CH_2_Ph **3**. Reactions with increasingly sterically encumbered reagents show competitive reaction pathways involving intermolecular dimerization leading to species analogous to **2** and **3** and those containing newly‐formed five‐membered rings *t*Bu_2_C_6_H_2_(C(OSiR_3_)C(OSiR_3_)CH_2_) (R=Me **6**, Ph **7**). Even further encumbered reagents proceed to either dimerize or react with additional CO to give a ketene‐like intermediates, thus affording a 7‐membered tropolone derivative **14** or the dione (3,5‐*t*Bu_2_C_6_H_3_)_3_C_6_H_2_CH_2_C(O))_2_ **15**.

## Graphical abstract

**Reactions of benzyl potassium species with CO** proceed via transient carbene‐like intermediates, resulting in dimerization. Increasingly sterically encumbered benzyl potassium reagents show competitive reaction pathways involving intramolecular cyclizations affording five‐membered rings, while even further encumbrance results the competitive with reaction with additional CO leading to a 7‐membered tropolone derivative.

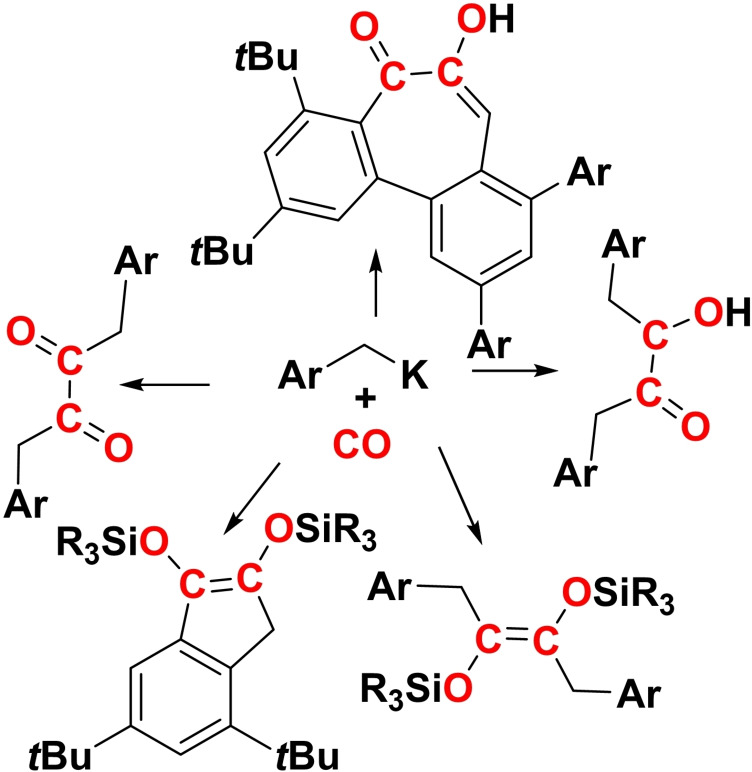

Carbon monoxide (CO) is used as a fundamental C1 building block for the synthesis of valuable organic products including hydrocarbons (Fischer–Tropsch process),^[1]^ aldehydes (Gattermann–Koch reaction^[2]^ and oxo synthesis^[3]^), acetic acid,^[4]^ esters^[5]^ among others.^[6]^ These processes are mediated by transition metal catalysts and indeed the chemistry of CO has been dominated by transition metal reagents. Despite lesser attention, reactions of CO with alkali metal reagents have a long history. In a 1970 study, Jutzi and Schröder^[7]^ reacted phenyl‐ and *n*‐butyllithium with CO and silylhalides to generate β‐ketosilanes, while the sterically bulky *t*‐butyllithium generated the silyl ketone. In the 1980s, Nudelman and Vitale^[8]^ described the carbonylation of phenyl lithium in the presence of alkyl halides, afforded diarylalkylcarbinols, while bulkier aryl lithium regents afforded 1,2‐diketone diaryl derivatives.^[9]^ In 1982, Seyferth and Weinstein^[10]^ used alkyl lithium reagents, CO and Me_3_SiCl to prepare a series of acyltrimethylsilanes (Scheme 1). In 1984, Murai et al.^[11]^ used α‐silylalkyllithium in reactions with CO to generate lithium enolates as a result of intramolecular 1,2 silyl migration (Scheme 1). The same group subsequently exploited such migrations to access cyclopropanone enolate, allenolate, and indene derivatives.^[12]^ Reactions of nitrogen derived lithium carbanions with CO have provided routes to N‐heterocyclic compounds^[13]^ while dialkenyllithium,^[14]^ or 1‐lithio‐butadienes^[15]^ reagents react with CO to generate 3‐cyclopenten‐1‐one derivatives (Scheme 1).

**Scheme 1:**
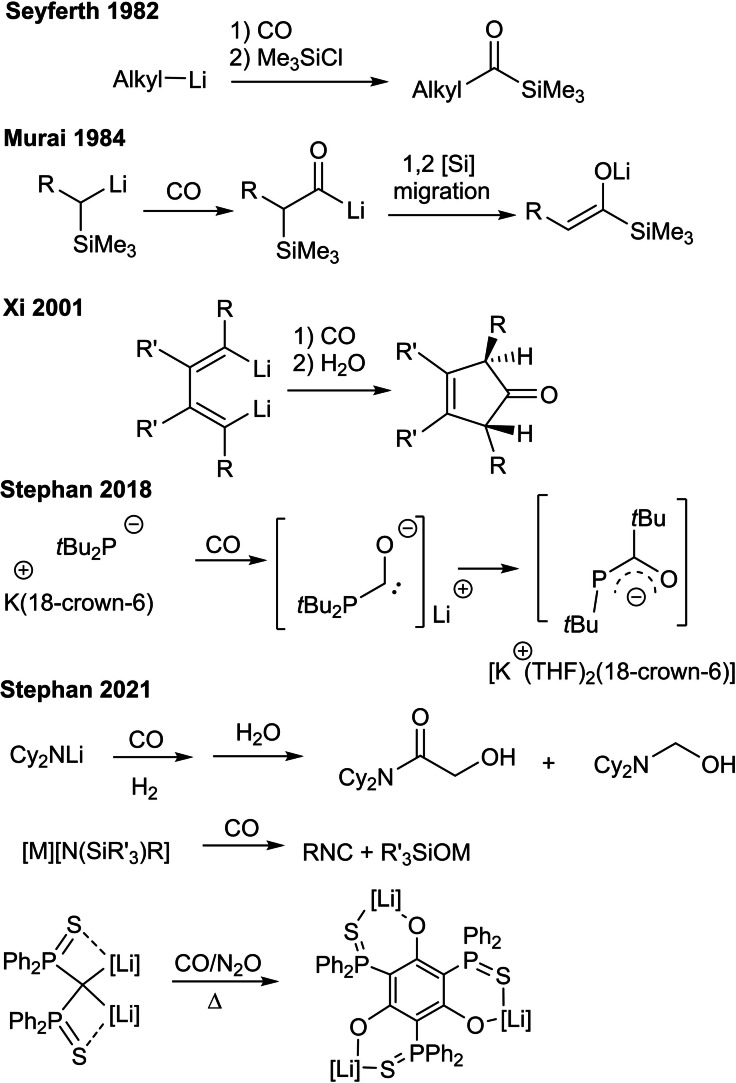
Selected reactions of alkali‐metal reagents with CO.

More recently, we have explored reactions of alkali metal reagents, noting the absence of a covalent linkage between the Lewis acidic alkali‐metal and the basic anion suggests an analogy to frustrated Lewis pairs (FLPs).^[16]^ Indeed, in our first test of this notion, alkali metal amides and phosphides were shown to reversibly activate H_2_ and were effective catalysts for the hydrogenation of imines or alkenes.^[17]^ We subsequently showed that like FLPs, alkali metal phosphides reacted with CO to generate transient anionic carbene‐like intermediates, prompting either alkyl group migration from phosphorus to carbon or dimerization (Scheme 1).^[18]^ The analogous reactions of alkali metal amides, were shown to effect CO homologation, affording (CO)_n_‐containing products (n=2,3,4).^[19]^ In the presence of syn‐gas, concurrent activation of H_2_ and CO resulted in the both C−C and C−H bond formations, demonstrating the potential for transition metal‐free reactions mimicking the Fischer−Tropsch process.^[19]^ In our most recent work, we have demonstrated the reactivity silylamides with CO to generate cyanide and isocyanides, respectively^[20]^ as well as the formation of a hexafunctionalized aromatic compound from the reactions of an dilithiomethane species with CO (Scheme 1).^[21]^

In the present study, we describe the reactions of benzyl potassium derivatives with CO. These reactions are shown to generate carbene‐like intermediates. In addition, steric encumbrance of the benzyl anion is also shown to impact on the nature of the resulting products. While sterically unencumbered benzyl potassium afforded products derived from carbene dimerization, more encumbered reagents lead to multiple CO propagation followed by intramolecular cyclization reactions affording 5 or 7‐membered ring products. The mechanism of these reactions is supported by both experimental evidence and a detailed computational study.

A solution of benzyl potassium **1** in THF was exposed to 4 atm pressure of CO in a Schlenk bomb. A color change from clear red solution to reddish brown suspension was observed on warming to room temperature. To this suspension, one equivalent of Ph_3_SiCl was added and the concentrated yellow filtrate in Et_2_O/pentane was stored at −25 °C freezer affording yellow crystals of the product **2** in 70% yield (Scheme 2). Repetition of the experiment using ^13^CO (1 atm) in THF proceeded in a similar fashion. The red suspension showed no signal in the ^13^C NMR spectrum consistent with the insolubility of the initial di‐anionic species. However, addition of Ph_3_SiCl led to a clean formation of **2**‐^**13**^ **C** as evidenced by the intense singlet signal at 138.72 ppm in the ^13^C{^1^H} NMR spectrum. A crystallographic study of **2** confirmed the formulation as the symmetric olefin ((Ph_3_SiO)(PhCH_2_)C)_2_. Correspondingly, the central C−C bond length was found to be 1.334(2) Å, while the remaining metric parameters were unexceptional (Scheme 2). Repetition of the reaction of **1**, followed by aqueous work up, PhCH_2_C(O)CH(OH)CH_2_Ph **3** was isolated as a yellow solid in 98% yield (See ESI).

**Scheme 2:**
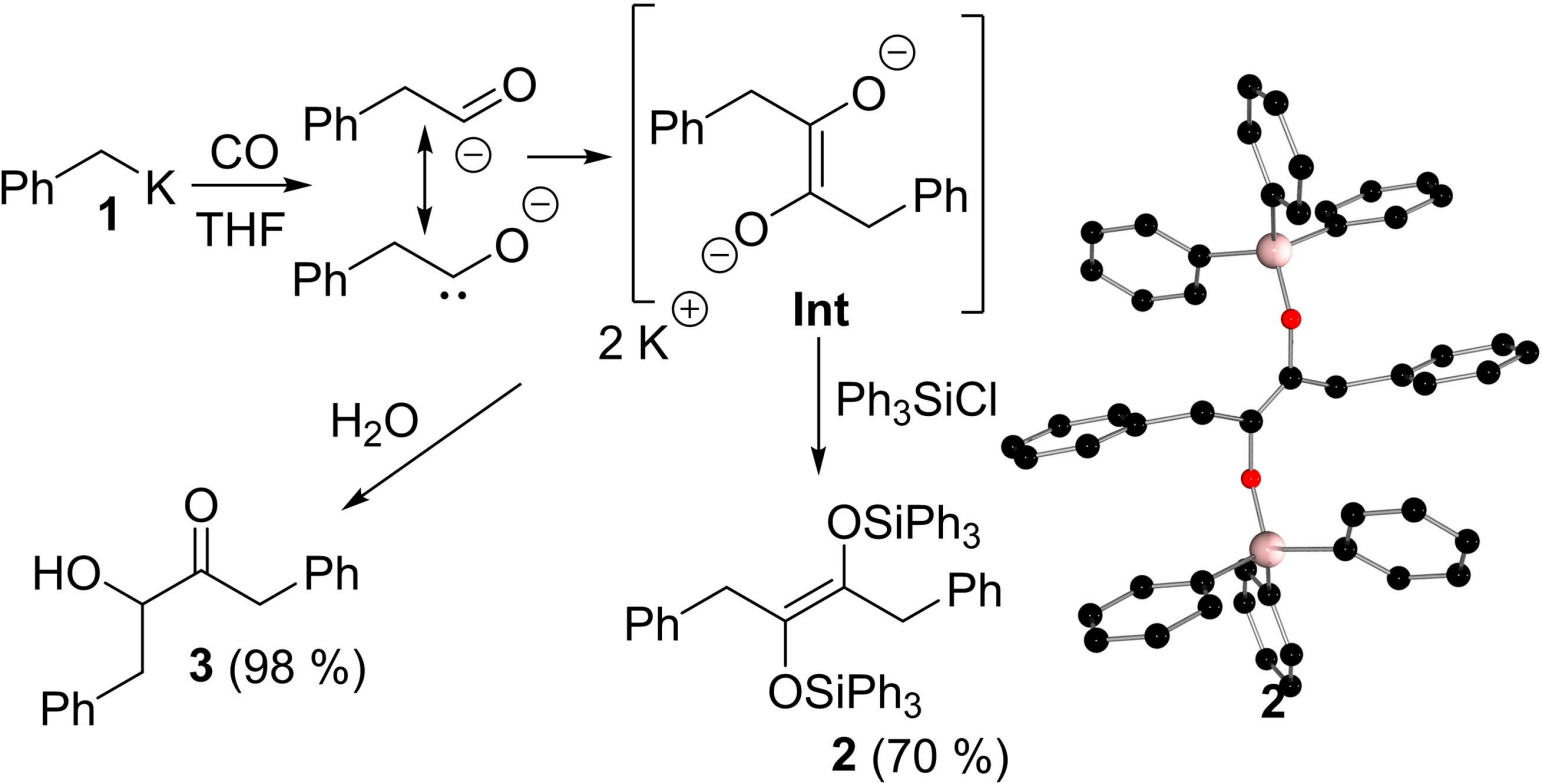
Reactions of benzyl potassium with CO, POV‐ray depiction of the molecular structure of **2**. Hydrogen atoms have been omitted for clarity. C: black; O: red; Si: pink.

The formation of **2** and **3** is initiated by the nucleophilic attack of CO by benzyl potassium, generating an intermediate with carbene‐character that reacts with an additional equivalent of CO to form the anionic ketene structure, which undergoes nucleophilic attack by another benzyl potassium to generate an insoluble *bis*‐alkoxide species **Int** (Scheme 2). Efforts to detect the carbene intermediate, by low temperature ^13^C NMR spectroscopy were unsuccessful. Similarly, efforts to intercept the proposed carbene via reaction with 1‐hexene failed to yield the anticipated cyclopropane derivative (see ESI). These observations imply that the formation of **Int** is rapid.

To gain mechanistic insight into this reactivity of benzyl potassium reagents, detailed reaction course of **1** with CO in THF solution was probed computationally using the dispersion corrected hybrid DFT PW6B95‐D3/def2‐QZVP+COSMO‐RS//TPSS‐D3/def2‐TZVP+COSMO level of theory including thermo‐statistical as well as continuum solvation contributions^[22]^ (Figure 1, for details see ESI). The dimer of **1** is computed to be 2.4 kcal/mol less stable than the separate monomers while the separated ion pair, K(THF)^+^ and benzyl anion is 6.6 kcal/mol less stable than the monomer KCH_2_Ph. These data suggest that the neutral monomer is the dominant species in solution at room temperature. However, the insertion of CO molecules into ionic K−C bonds to form carbene‐ or ketene‐like species is both kinetically and thermodynamically favored for the dimer form of **1** (see ESI). The initial CO insertion into the dimer of **1** is 3.8 kcal/mol endergonic over a low barrier (via transition state **TS1**, 8.9 kcal/mol with respect to two monomers of **1**). This gives the carbene‐like intermediate **A**. A second CO addition to the carbene‐site of **A** is −12.1 kcal/mol exergonic over a low barrier of 7.8 kcal/mol (via **TS2**) generates a ketene‐like species **B**, which is −8.3 kcal/mol exergonic over an overall barrier of only 11.6 kcal/mol). Subsequent intramolecular nucleophilic attack of the electrophilic ketene **B** by the benzyl anion (via **TS3**) is −26.3 kcal/mol exergonic over a low barrier of 12.4 kcal/mol. This affords complex **C** which ultimately gives **2** and **3**. In principle **C** could also be formed via the coupling of two carbenes **A**, however this is kinetically 3.1 kcal/mol less favorable.

**Figure 1:**
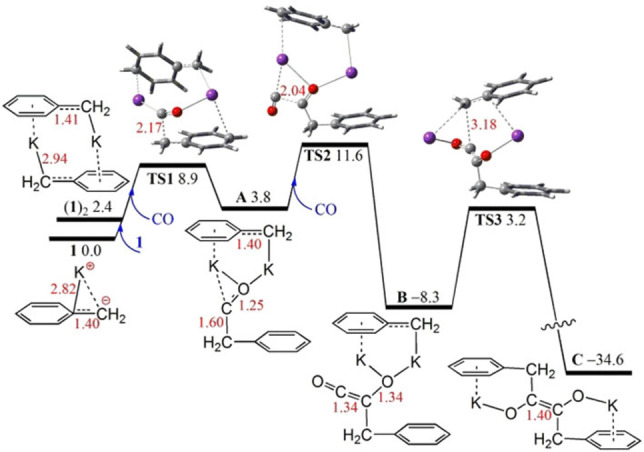
DFT‐computed reaction Gibbs free energy profile (in kcal/mol, at 298 K and 1 mol/L) of **1** with CO in THF solution. In ball‐and‐stick models, crucial K, C, H and O atoms are highlighted as violet, grey, white and red balls, respectively, with selected bond lengths (in red) shown in Å.

To further probe this reactivity, the sterically hindered benzyl potassium, K[2,4,6‐(*t*Bu)_3_C_6_H_2_CH_2_] **4** was synthesized from 2,4,6*‐*tri‐*tert‐*butyl toluene and a slight excess *n*BuLi/KO*t*Bu. Exposure of a clear orange THF solution of **4** to 1 atm of ^13^CO, resulting in a brownish‐orange suspension. Subsequently, addition of Me_3_SiCl resulted in formation of two products, **5**‐^**13**^ **C** and **6**‐^**13**^ **C** in an approximately 1 : 1 ratio based on the integration in the ^13^C{^1^H} NMR spectrum. The single intense ^13^C NMR signal at 136.15 ppm for **5**‐^**13**^ **C** was consistent with the linear olefinic species ((Me_3_SiO)(2,4,6‐(*t*Bu)_3_C_6_H_2_CH_2_)^13^C)_2_. This formulation was confirmed as X‐ray quality crystals were isolated in 30% yield from Et_2_O/toluene (Scheme 3a). In the case of **6**‐^**13**^ **C**, two olefinic ^13^C{^1^H} resonances at 137.45 ppm and 132.75 ppm, with a coupling constant of 94 Hz were consistent with the formulation as *t*Bu_2_C_6_H_2_(^13^C(OSiMe_3_) ^13^C (OSiMe_3_)CH_2_). This latter formulation was further supported by repetition of the reaction followed by addition of Ph_3_SiCl. Work‐up and cooling of the pentane extract at −25 °C afforded the product **7**‐^**13**^ **C** in 52% isolated yield. Similar to **6**‐^**13**^ **C**, the ^13^C NMR spectrum contained two olefinic carbon resonances at 138.99 ppm and 133.25 ppm with a coupling constant of 96 Hz, consistent with a dissymmetric olefinic linkage. X‐ray data confirmed that compound **7** is formulated as *t*Bu_2_C_6_H_2_(C(OSiPh_3_)C(OSiPh_3_)CH_2_) in which a five‐membered ring is fused to the arene ring at adjacent carbon atoms, indicating the loss of a *t*Bu‐group (Scheme 3). Within the five membered ring the olefinic C−C bond length is 1.331(4) Å (Scheme 3b).

**Scheme 3:**
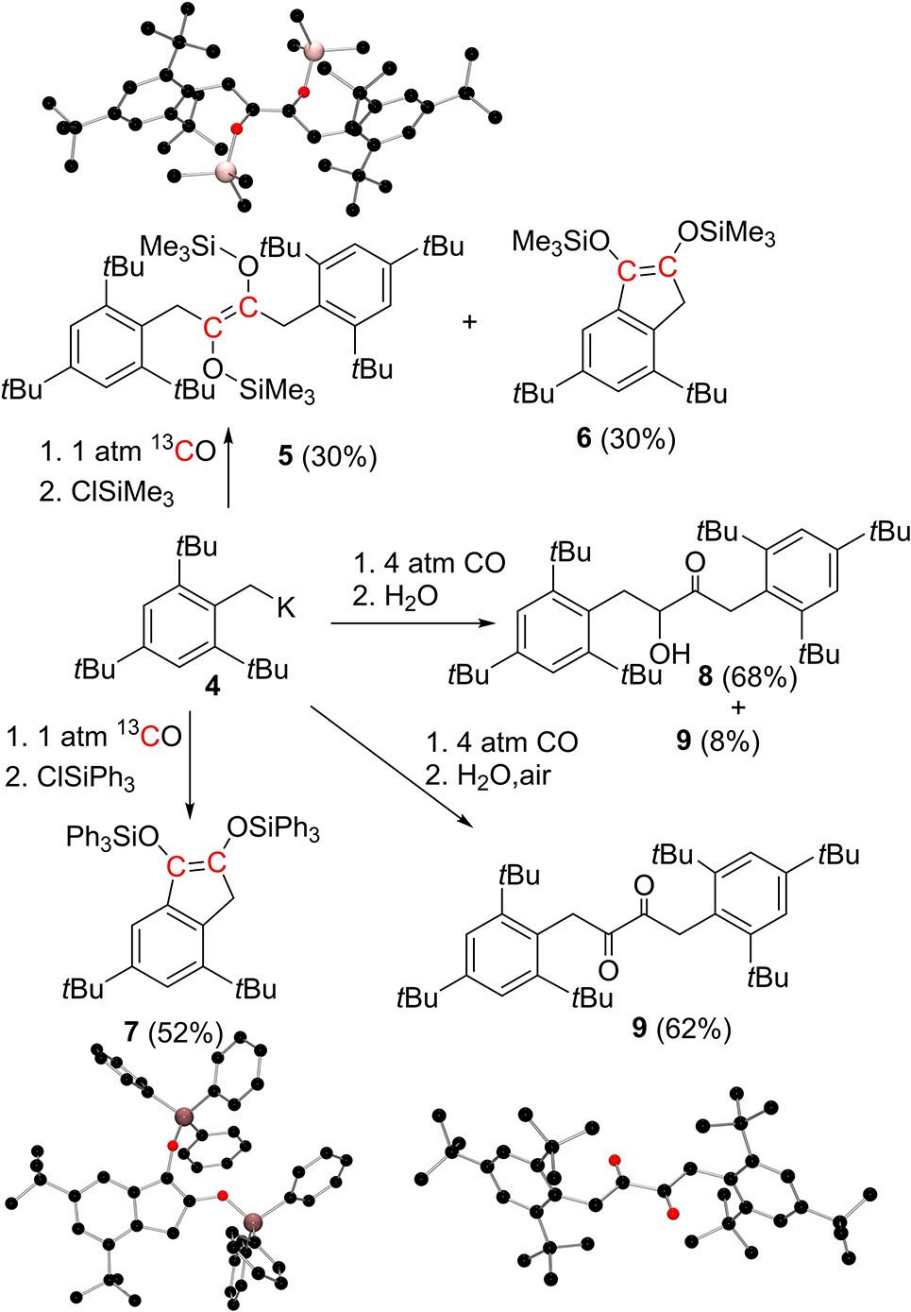
Reactions of **4** with CO; POV‐ray depiction of the molecular structure of (a) **5**, (b) **7** (c) **9**. Hydrogen atoms have been omitted for clarity. C: black; O: red; Si: pink.

Interestingly, repetition of the reaction of **4** with CO, followed by anaerobic aqueous work up with degassed water, afforded a mixture of products in an overall 76% isolated yield. The mixture was consists of a *α*‐hydroxy ketone (2,4,6‐(*t*Bu)_3_C_6_H_2_)CH_2_C(O)CH(OH)CH_2_(2,4,6‐(*t*Bu)_3_C_6_H_2_) **8** and a 1,2‐diketone species **9** formulated as (2,4,6‐(*t*Bu)_3_C_6_H_2_CH_2_CO)_2_ in an approximately 9 : 1 ratio according to the integrations in the ^1^H NMR spectrum (Scheme 3). The ^13^C resonances attributable to the *C*−OH and *C*=O of **8** were observed at 79.62 ppm and 210.89 ppm, respectively. Under similar condition reaction of **4** with ^13^CO afforded the formation of the isotopologues **8**‐^**13**^ **C** and **9**‐^**13**^ **C** which was confirmed by high resolution mass spectrometry (HRMS), suggesting both carbon atoms are sourced from CO. Performing the reaction with an aqueous workup in air afforded yellow crystals of the 1,2‐diketone derivative (2,4,6‐(*t*Bu)_3_C_6_H_2_CH_2_CO)_2_ **9** in 62% isolated yield which were recrystallized from a saturated EtOAc solution at −10 °C. In this case, the ^13^C resonances attributable to the carbonyl carbon was seen at 200 ppm in the ^13^C NMR spectrum. The formulation of **9** was also unambiguously confirmed by X‐ray crystallographic study (Scheme 3c).

The isolation of **5**–**9** infers steric influence over the nature of the products. Indeed, our DFT calculations show that the dimer (**4**)_2_ of bulky benzyl potassium **4** is 5.9 kcal/mol less stable than two monomers, thus is further disfavored in solution. The first CO addition to **4** leads to an unstable carbene‐like complex **At** (Figure 2), which rapidly react with another CO to form the ketene‐like intermediate **Bt** in a process that is exergonic by only −0.2 kcal/mol (assumed 1 M CO) over a low free‐energy barrier of 12.9 kcal/mol (via **TS2t**). Nucleophilic trapping of **Bt** with **4** to form **Ct** is −15.5 kcal/mol exergonic over a very low free‐energy barrier of 3.5 kcal/mol (via **TS3t**). While this is kinetically and thermodynamically facile, it will be limited by low concentration of **Bt** at low CO pressure, consistent with the formation of linear products **5**, **8** and **9**. At low CO pressure, intramolecular nucleophilic cyclization of **Bt** affording the five‐membered‐ring species **Dt** is −3.1 kcal/mol exergonic over a very low barrier of 8.4 kcal/mol (via **TS4t**). Deprotonation by **4**, and elimination of butene reduces **Dt** to the cyclic salt **Et** in a highly exergonic process over a low barrier of 15.6 kcal/mol (via **TS5t**). This is consistent with the formation of **6** and **7** after treatment with silyl chlorides.

**Figure 2:**
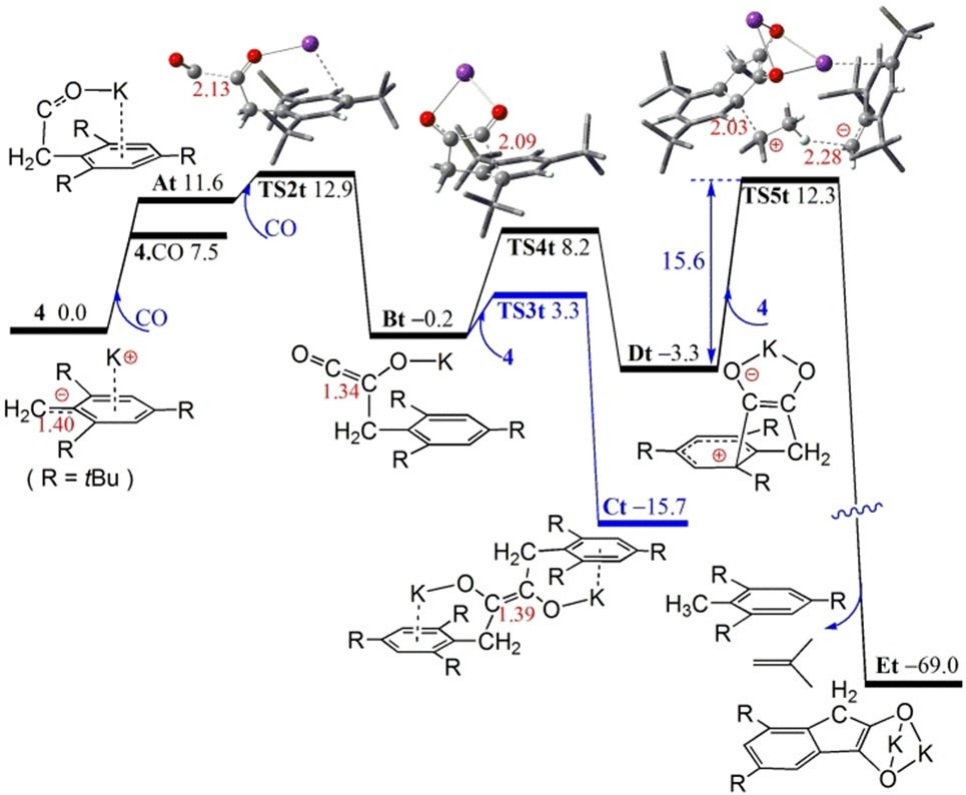
DFT‐computed reaction Gibbs free energy profile (in kcal/mol, at 298 K and 1 mol/L) of **4** with CO in THF solution. In ball‐and‐stick models, crucial K, C, H and O atoms are highlighted as violet, grey, white and red balls, respectively, with selected bond lengths (in red) shown in Å.

A final modification of the benzyl potassium reagent was derived from deprotonation of the triaryl toluene, 2,4,6‐(3,5*‐t*Bu_2_C_6_H_3_)_3_C_6_H_2_Me **10** with *n*BuLi/KO*t*Bu affording K[2,4,6‐(3,5‐*t*Bu_2_C_6_H_3_)_3_C_6_H_2_CH_2_] **11** in 80% isolated yield. Reaction of **11** with ^13^CO in diethyl ether also led to a color change from dark green to dark red brown, the reaction yielded a distinct paramagnetic product **12**, as evidenced by the EPR spectrum (see ESI). Simulation revealed a g=2.007 with couplings to two ^13^C carbon atoms (3.0, 3.9 G), the two methylene (7.0 G), and four aromatic protons (0.4, 0.3, 0.3, 1.5 G) and one *t‐*butyl group (0.4 G) consistent with DFT calculations for the incorporation of two ^13^CO fragments in the radical anion salt, K[3,5‐(*t*Bu)_2_C_6_H_2_)C(O)C(O)CH_2_C_6_H_2_(C_6_H_3_3,5‐(*t*Bu)_2_)_2_]^.^ **12** (Scheme 4). The ^13^C NMR spectrum of the reaction mixture revealed intense singlet signal at 130.75 ppm, consistent with the formation of the diamagnetic *bis*‐alkoxide salt **13**. These observations suggest that the initial carbene intermediate reacts with additional CO to give a ketene‐like species, while competing intramolecular cyclization (with formal H‐atom loss) and dimerization give **12** and **13**, respectively. Quenching the reaction with water provided [3,5‐(*t*Bu)_2_C_6_H_2_)C(O)C(OH)CHC_6_H_2_(C_6_H_3_3,5‐(*t*Bu)_2_)_2_] **14** and ((2,4,6‐(3,5‐*t*Bu_2_C_6_H_3_)_3_C_6_H_2_CH_2_C(O))_2_ **15** in 12% and 13% yield, respectively. HRMS and spectroscopic data were consistent with these formulations (see ESI).^[23]^ In particular, the ^13^C NMR data for **14** showed two intense doublets at 196.95 and 147.41 ppm with ^1^ *J*_C‐C_=61 Hz, consistent with the incorporation of two ^13^CO molecules. As such, this represents a unique single‐step synthesis of a tropolone derivative. Such seven‐membered aromatic species are well known for their biological activity.^[24]^

**Scheme 4:**
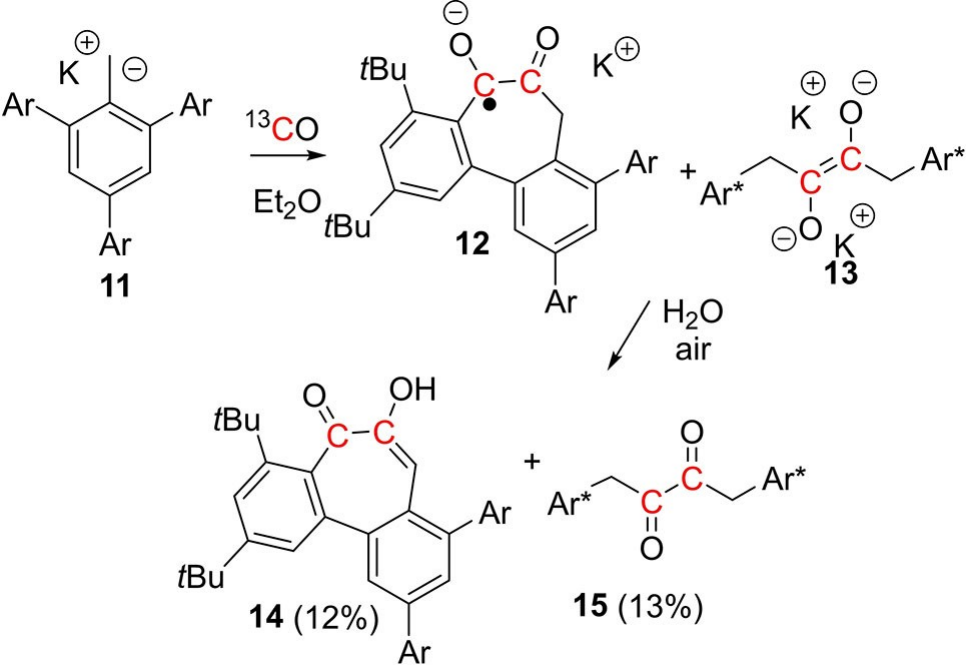
Reactions of **11** with CO in THF and ether affording **12**–**15**. (Ar=3,5‐(*t*Bu)_2_C_6_H_3_, Ar*=2,4,6‐Ar_3_C_6_H_2_). The yields of **14** and **15** were determined from the integration in ^13^C{^1^H} NMR spectrum, using a ^13^C enriched urea as the internal standard.

In conclusion, this report has demonstrated that benzyl potassium species react with CO to generate transient carbene‐like intermediates. In sterically unhindered cases, formal dimerization of the carbene is the dominant reaction pathway. In contrast, for reactions with increasingly sterically encumbered systems, intramolecular cyclization is increasingly competitive as carbene attack of aromatic substituents or additional CO affords avenues to 5 and 7 membered ring species. The latter 7 membered ring derivative is derived from a transient radical intermediate. We are continuing to examine the reactions of alkali metal species with CO in efforts to exploit CO as a building block for more complex organic products.

**Supporting Information**: Electronic Supplementary Information (ESI) available: Synthetic and spectral data, computational details and DFT‐computed energies and Cartesian coordinates are deposited. X‐ray crystallographic data can be obtained from the CCDC 2089943‐2089946.

## Conflict of interest

The authors declare no conflict of interest.

## Supporting information

As a service to our authors and readers, this journal provides supporting information supplied by the authors. Such materials are peer reviewed and may be re‐organized for online delivery, but are not copy‐edited or typeset. Technical support issues arising from supporting information (other than missing files) should be addressed to the authors.

Supporting Information

## References

[1a] H. Schulz , Appl. Catal. A 1999, 186, 3–12;

[1b] F. Fischer , K. Meyer , Brennst.-Chem. 1931, 12;

[1c] D. W. McKee , J. Catal. 1967, 8, 240–249.

[2a] L. Gattermann , W. Berchelmann , Ber. Dtsch. Chem. Ges. 1898, 31, 1765–1769;

[2b] R. Adams , I. Levine , J. Am. Chem. Soc. 1923, 45, 2373–2377.

[3] R. Franke , D. Selent , A. Börner , Chem. Rev. 2012, 112, 5675–5732. 22937803 10.1021/cr3001803

[4a] G. J. Sunley , D. J. Watson , Catal. Today 2000, 58, 293–307;

[4b] A. Haynes , P. M. Maitlis , G. E. Morris , G. J. Sunley , H. Adams , P. W. Badger , C. M. Bowers , D. B. Cook , P. I. P. Elliott , T. Ghaffar , H. Green , T. R. Griffin , M. Payne , J. M. Pearson , M. J. Taylor , P. W. Vickers , R. J. Watt , J. Am. Chem. Soc. 2004, 126, 2847–2861. 14995202 10.1021/ja039464y

[5a] G. Kiss , Chem. Rev. 2001, 101, 3435–3456; 11840990 10.1021/cr010328q

[5b] M. Beller , B. Cornils , C. D. Frohning , C. W. Kohlpaintner , J. Mol. Catal. A 1995, 104, 17–85;

[5c] A. Schoenberg , I. Bartoletti , R. F. Heck , J. Org. Chem. 1974, 39, 3318–3326.

[6a] J.-B. Peng , H.-Q. Geng , X.-F. Wu , Chem 2019, 5, 526–552 ;

[6b] J. Falbe , Carbon monoxide in organic synthesis, Springer-Verlag, Berlin, 1970.

[7] P. Jutzi , F.-W. Schröder , J. Organomet. Chem. 1970, 24, 1–5.

[8] N. S. Nudelman , A. A. Vitale , J. Org. Chem. 1981, 46, 4625–4626.

[9] N. S. Nudelman , P. Outumuro , J. Org. Chem. 1982, 47, 4347–4348.

[10] D. Seyferth , R. M. Weinstein , J. Am. Chem. Soc. 1982, 104, 5534–5535.

[11] S. Murai , I. Ryu , J. Iriguchi , N. Sonoda , J. Am. Chem. Soc. 1984, 106, 2440–2442.

[12] I. Ryu , Y. Hayama , A. Hirai , N. Sonoda , A. Orita , K. Ohe , S. Murai , J. Am. Chem. Soc. 1990, 112, 7061–7063.

[13a] A. Orita , M. Fukudome , K. Ohe , S. Murai , J. Org. Chem. 1994, 59, 477–481;

[13b] K. Smith , G. A. El-Hiti , G. J. Pritchard , A. Hamilton , J. Chem. Soc.-Perkin Trans. 1999, 2299–2303;

[13c] K. Iwamoto , N. Chatani , S. Murai , J. Org. Chem. 2000, 65, 7944–7948. 11073602 10.1021/jo000977m

[14] Q. Song , J. Chen , X. Jin , Z. Xi , J. Am. Chem. Soc. 2001, 123, 10419–10420. 11604006 10.1021/ja0165638

[15] Q. Song , Z. Li , J. Chen , C. Wang , Z. Xi , Org. Lett. 2002, 4, 4627–4629. 12489946 10.1021/ol026977l

[16a] D. W. Stephan , Chem 2020, 6, 1520–1526;

[16b] A. R. Jupp , D. W. Stephan , Trends Chem. 2019, 1, 35–48;

[16c] D. W. Stephan , Science 2016, 354, aaf7229;27940818

[16d] D. W. Stephan , G. Erker , Angew. Chem. Int. Ed. 2015, 54, 6400–6441; 10.1002/anie.201409800 25974714

[16e] D. W. Stephan , J. Am. Chem. Soc. 2015, 137, 10018–10032; 26214241 10.1021/jacs.5b06794

[16f] D. W. Stephan , Acc. Chem. Res. 2015, 48, 306–316. 25535796 10.1021/ar500375j

[17] M. Xu , A. R. Jupp , Z. W. Qu , D. W. Stephan , Angew. Chem. Int. Ed. 2018, 57, 11050–11054; 10.1002/anie.201806849 29961980

[18] M. Xu , A. R. Jupp , D. W. Stephan , Angew. Chem. Int. Ed. 2019, 58, 3548–3552; 10.1002/anie.201814562 30650223

[19] M. Xu , Z.-W. Qu , S. Grimme , D. W. Stephan , J. Am. Chem. Soc. 2021, 143, 634–638. 33399459 10.1021/jacs.0c11482

[20] M. Xu , B. Kooij , T. Wang , J. H. Lin , Z. W. Qu , S. Grimme , D. W. Stephan , Angew. Chem. Int. Ed. 2021, 60, 16965–16969. 10.1002/anie.202105909 34004079

[21] M. Xu , T. Wang , Z.-w. Qu , S. Grimme , D. W. Stephan , Angew. Chem. Int. Ed. 2021, 10.1002/anie.202111486.

[22a] TURBOMOLE, V 7.4, TURBOMOLE GmbH, Karlsruhe, **2019**, See http://www.turbomole.com;

[22b] J. Tao , J. P. Perdew , V. N. Staroverov , G. E. Scuseria , Phys. Rev. Lett. 2003, 91, 146401; 14611541 10.1103/PhysRevLett.91.146401

[22c] S. Grimme , J. Antony , S. Ehrlich , H. Krieg , J. Chem. Phys. 2010, 132, 154104; 20423165 10.1063/1.3382344

[22d] S. Grimme , S. Ehrlich , L. Goerigk , J. Comput. Chem. 2011, 32, 1456–1465; 21370243 10.1002/jcc.21759

[22e] S. Grimme , Chem. Eur. J. 2012, 18, 9955–9964; 22782805 10.1002/chem.201200497

[22f] F. Weigend , R. Ahlrichs , Phys. Chem. Chem. Phys. 2005, 7, 3297–3305; 16240044 10.1039/b508541a

[22g] F. Weigend , Phys. Chem. Chem. Phys. 2006, 8, 1057–1065; 16633586 10.1039/b515623h

[22h] A. Klamt , G. Schüürmann , J. Chem. Soc.-Perkin Trans. 1993, 2, 799–805;

[22i] F. Eckert , A. Klamt , AIChE J. 2002, 48, 369–385;

[22j] F. Eckert, A. Klamt, COSMOtherm, Version C3.0, Release 16.01, COSMOlogic GmbH & Co., Leverkusen, Germany, **2015**, ;

[22k] Y. Zhao , D. G. Truhlar , J. Phys. Chem. A 2005, 109, 5656–5667. 16833898 10.1021/jp050536c

[23] The reaction of **11** and CO in THF, followed by workup also gave **15** but not **14**. The six-membered 3,5-(tBu)_2_C_6_H_2_)C(O)CH_2_C_6_H_2_(C_6_H_3_)3,5-(tBu)_2_)_2_ **17** was isolated in 1% yield and its radical precursor characterized by EPR spectroscopy (see ESI).

[24a] S. Chakrabarty , E. O. Romero , J. B. Pyser , J. A. Yazarians , A. R. H. Narayan , Acc. Chem. Res. 2021, 54, 1374–1384; 33600149 10.1021/acs.accounts.0c00810 PMC8210581

[24b] N. Liu , W. Song , C. M. Schienebeck , M. Zhang , W. Tang , Tetrahedron 2014, 70, 9281–9305; 25400298 10.1016/j.tet.2014.07.065 PMC4228802

[24c] J. Zhao , Curr. Med. Chem. 2007, 14, 2597–2621. 17979713 10.2174/092986707782023253

