# Supplementary material for: Steric Influence on Reactions of Benzyl Potassium Species with CO
Source: Chem Asian J. 2021 Oct 13;16(22):3640–4. doi: 10.1002/asia.202101127 (PMC9292647; doi:10.1002/asia.202101127)
Supplement: Supplementary file 1 — Supporting Information [file ASIA-16-3640-s001.pdf]

CHEMISTRY
AN ASIAN JOURNAL

Supporting Information

Steric Influence on Reactions of Benzyl Potassium Species with CO

Tongtong Wang, Maotong Xu, Andrew R. Jupp, Zheng-Wang Qu,* Stefan Grimme, and Douglas W. Stephan*© 2021 The Authors. Chemistry - An Asian Journal published by Wiley-VCH GmbH. This is an open access article under the terms of the Creative Commons Attribution License, which permits use, distribution and reproduction in any medium, provided the original work is properly cited.

Supplementary Materials for

Steric Influence on Reactions of Benzyl Potassium Species with CO

Tongtong Wang[a,b], Maotong Xu[a], Andrew Jupp[a], Zheng-Wang Qu[c*], Stefan Grimme[c], Douglas W. Stephan[a*]

Contents

General Considerations 4

Formation of **2** from benzyl potassium **1** with CO/$ClSiPh_3$ 5

Figure S 1. ^{1}H NMR of **2**, C_6D_6 5

Figure S 2. $^{13}C\{^{1}H\}$ NMR of **2**, C_6D_6 6

Figure S 3. HRMS of **2**, ESI ionization 6

Formation of ^{13}C-**2** from benzyl potassium **1** with $^{13}CO/ClSiPh_3$ 7

Figure S 4. $^{13}C\{^{1}H\}$ NMR spectrum, the brownish suspension from **1** and ^{13}CO, THF 7

Figure S 5. $^{13}C\{^{1}H\}$ NMR spectrum, crude mixture contains **2-^{13}C** after $ClSiPh_3$ addition, THF 8

Figure S 6. HRMS of **2-^{13}C**, DART ionization 8

Formation of **3** from benzyl potassium **1** with CO/H_2O 9

Figure S 7. ^{1}H NMR of **3**, $CDCl_3$ 9

Benzyl potassium **1** with CO at -78°C/1-hexene 10

1,3,5-Tris-*tert*-butyl toluene[4] 11

Figure S 8. ^{1}H NMR of 1,3,5-tris-*tert*-butyl toluene, C_6D_6 11

2,4,6-Tris-*tert*-butyl benzyl potassium **4** 12

Figure S 9. ^{1}H NMR of 2,4,6-tris-*tert*-butyl benzyl potassium **4**, THF 12

Formation of **5** and **6** from **4** with $^{13}CO/ClSiMe_3$ 13

Figure S 10. $^{13}C\{^{1}H\}$ NMR of crude reaction mixture containing **5-^{13}C** and **6-^{13}C**, THF 14

Figure S 11. ^{1}H NMR spectrum of **5-^{13}C**, C_6D_6 14

Figure S 12. $^{13}C\{^{1}H\}$ NMR of **5-^{13}C**, C_6D_6 15

Figure S 13. HRMS of **5-^{13}C**, DART ionization 15

Figure S 14. HRMS of **6-^{13}C**, DART ionization 16

Formation of **7-^{13}C** from **4** with $^{13}CO/ClSiPh_3$ 17

Figure S 15. ^{1}H NMR of **7-^{13}C**·0.5 Et_2O, C_6D_6 18

Figure S 16. $^{13}C\{^{1}H\}$ NMR of **7-^{13}C**·0.5 Et_2O, C_6D_6 18

Figure S 17. gCOSY spectrum of **7-^{13}C**·0.5 Et_2O, C_6D_6 19

Figure S 18. HSQC spectrum of **7-^{13}C**·0.5 Et_2O, C_6D_6 19

Figure S 19. HMBC spectrum of **7-^{13}C**·0.5 Et_2O, C_6D_6 20

Figure S 20. HRMS of **7-^{13}C**·0.5 Et_2O, ESI ionization ... 20
Formation of **7** from **4** with CO/$ClSiPh_3$ ... 21
Figure S 21. HRMS of **7**, DART ionization ... 21
Formation of **8** and **9** from **4** with CO/degassed water ... 22
Figure S 22. ^{1}H NMR spectra: **9** (top); mixture of **8** and **9** (bottom), $CDCl_3$ ... 23
Figure S 23. ^{1}H NMR spectrum of **8**, $CDCl_3$ ... 23
Figure S 24. ^{13}C{^{1}H} NMR of **8**, $CDCl_3$ ... 24
Figure S 25. HRMS of **8**, DART ionization ... 24
Formation of **9** from **4** with CO/aqueous work in air ... 25
Figure S 26. ^{1}H NMR of **9**, $CDCl_3$ ... 26
Figure S 27. ^{13}C{^{1}H} NMR of **9**, $CDCl_3$ ... 26
Figure S 28. ^{13}C NMR of **9**, $CDCl_3$ ... 27
Figure S 29. HRMS of **9**, DART ionization ... 27
In situ formation of **8-^{13}C** and **9-^{13}C** from of **4** with ^{13}CO/degassed water ... 28
Figure S 30. HRMS of **8-^{13}C**, DART ionization ... 28
Figure S 31. HRMS of **9-^{13}C**, DART ionization ... 29
Reaction of **4** with CO at -78°C/1-hexene ... 29
1,3,5-Tri-aryltoluene (aryl= 3,5-di-*tert*-butylphenyl) **10** ... 30
Figure S 32. ^{1}H NMR of **10**, THF-d_8 ... 31
Figure S 33. ^{13}C{^{1}H} NMR of **10**, THF-d_8 ... 31
Figure S 34. ^{1}H NMR of **10**, $CDCl_3$ ... 32
Figure S 35. ^{13}C{^{1}H} NMR of **10**, $CDCl_3$ ... 32
Figure S 36. ^{1}H NMR of **10**, C_6D_6 ... 33
Figure S 37. HRMS of **10**, EI ionization ... 33
2,4,6-(3,5-*t*$Bu_2C_6H_3)_3C_6H_2CH_2K$ **11** ... 34
Figure S 38. ^{1}H NMR spectrum of **11**, C_6D_6 ... 35
Figure S 39. ^{13}C{^{1}H} NMR of **11**, C_6D_6 ... 35
Figure S 40. ^{1}H NMR spectrum of **11**, Et_2O ... 36
Figure S 41. ^{1}H NMR spectrum of **11**, THF ... 36
Reaction of **11** with ^{13}CO in Et_2O/ aqueous work up ... 37
Figure S 42. ^{13}C{^{1}H} NMR study of a mixture containing **10**, **14** and **15**, $CDCl_3$ ... 37
Figure S 43. HRMS of **14**, DART ionization ... 38
Figure S 44. EPR of radical **12**, experimental: black; simulation red. ... 38
Reaction of **11** with ^{13}CO in THF/ aqueous work up ... 39
Figure S 45. EPR spectrum of the radical anion **16**; experimental: black; simulation red. ... 40
Figure S 46. ^{1}H NMR study of a mixture containing **10**, **17** and **15**, $CDCl_3$ ... 41

Figure S 47. $^{13}C\{^1H\}$ NMR study of a mixture containing **10**, **17** and **15**, $CDCl_3$ 41

Figure S 48. HRMS of **17**, DART ionization 42

Figure S 49. HRMS of **15**, DART ionization 42

DFT calculations 43

Computational Details: 43

Figure S 50. The DFT-computed Gibbs free energy paths in THF solution (in kcal/mol, at 298 K and 1M concentration) at PW6B95-D3 + COSMO-RS level for the reactions of benzyl potassium **1** KCH_2Ph with CO: (A) via dimeric (**1**)$_2$; (B) via monomeric **1**. 44

Figure S 51. The DFT-computed Gibbs free energy paths in THF solution (in kcal/mol, at 298 K and 1M concentration) at PW6B95-D3 + COSMO-RS level for the reactions of bulky *tert*-butyl-substituted benzyl potassium **4** KBzt with CO: (A) via monomer **4**; (B) via dimeric (**4**)$_2$. 45

Figure S 52. DFT-computed Gibbs free energy paths in THF solution (in kcal/mol, at 298 K and 1M concentration) at PW6B95-D3 + COSMO-RS level for the reactions of benzyl potassium **11**: (A) the first CO insertion; (B) the second CO insertion. Some potentially facile processes are shown in dashed lines. With very bulkyl aryl-substituents, only monomeric mechanism is expected............ 46

References 127

General Considerations

Unless otherwise specified, all manipulations were performed under N_2 using Schlenk techniques or a Mbraun Atmospheres glovebox. 4 Å molecular sieves was activated by heating at 300°C under vacuum for 2 days. Pentane, hexane and toluene were dried by a Grubbs type Innovative Technologies solvent purification system, THF and diethyl ether were dried over sodium and distilled. All the solvents were degassed on Schlenk line and stored over activated 4 Å molecular sieves prior to use. THF-d_8 and C_6D_6 were degassed on Schlenk line and stored over activated 4 Å molecular sieves prior to use. $CDCl_3$ was dried over CaH_2 and distilled. All glassware was dried in a 180 °C oven overnight prior to use. All reagents were used as received without further purifications, unless otherwise specified. Carbon monoxide and carbon-^{13}C monoxide (99 atom % ^{13}C, <5 atom % ^{18}O) were purchased from Sigma Aldrich. Triphenyl chlorosilane was recrystallized from diethyl ether at -25°C prior to use. Trimethyl chlorosilane was dried over CaH_2 and distilled. ^{13}C enriched 1,3-bis(2,6-diisopropylphenyl)urea was synthesized from a known method.[1] Benzyl potassium was synthesized from a known method.[2] NMR spectra were obtained at room temperature on Bruker Advance III 400 MHz, Bruker Ultrashield 400 MHz, Agilent DD2 500 MHz and Agilent DD2 600 MHz spectrometer. NMR chemical shifts are reported in ppm and referenced to $SiMe_4$ (^{1}H and ^{13}C). Coupling constants are listed in hertz. Multiplicity is reported as follows: s = singlet, d = doublet, t = triplet, m = multiplet, b = broad. EPR spectrum was obtained at room temperature on Bruker CW X-band ECS-EMXplus EPR Spectrometer. Mass Spectrometry was operated by staff at AIMS Mass Spectrometry Laboratory at Chemistry department, University of Toronto. Single crystal X-ray crystallographic analyses were performed on crystals coated in Paratone-N oil and mounted on a Bruker Kappa Apex II diffractometer.

Formation of **2** from benzyl potassium **1** with CO/ClSiPh3

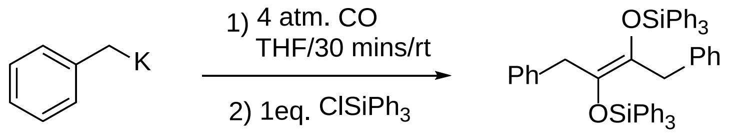

2

Benzyl potassium (50 mg, 0.38 mmol) was dissolved in 20 mL THF and transferred to a Schlenk bomb. The solution was freeze-thaw degassed under vacuum, then 4 atm CO was added to the Schlenk bomb in a liquid N_2 bath. The reaction turned from dark red clear to red brownish suspension immediately after warming up to room temperature. The reaction was left at room temperature for 30 minutes, $ClSiPh_3$ (113 mg, 0.38 mmol) was added, resulted in a pale-yellow clear solution. The reaction was left overnight at room temperature, then the volatiles were removed under vacuum. The residue was extract with diethyl ether, the orange solution was added a few drops of pentane, stored at -25°C freezer afforded yellow crystals of 2 (102 mg, 70%).

^{1}H NMR (400 MHz, C_6D_6) δ 7.56 (d, *J* = 7.2 Hz, 10H), 7.19 – 7.02 (m, 30H), 3.79 (s, 4H).

^{13}C NMR (101 MHz, C_6D_6) δ 139.43, 139.41, 136.02, 134.65, 130.32, 129.30, 128.50 128.17, 126.16, 36.79.

HRMS (ESI ionization, *m/z*): calcd for $C_{52}H_{48}Si_2O_2N$, $[M+NH_4]^+$: 774.3228; found: 774.3218.

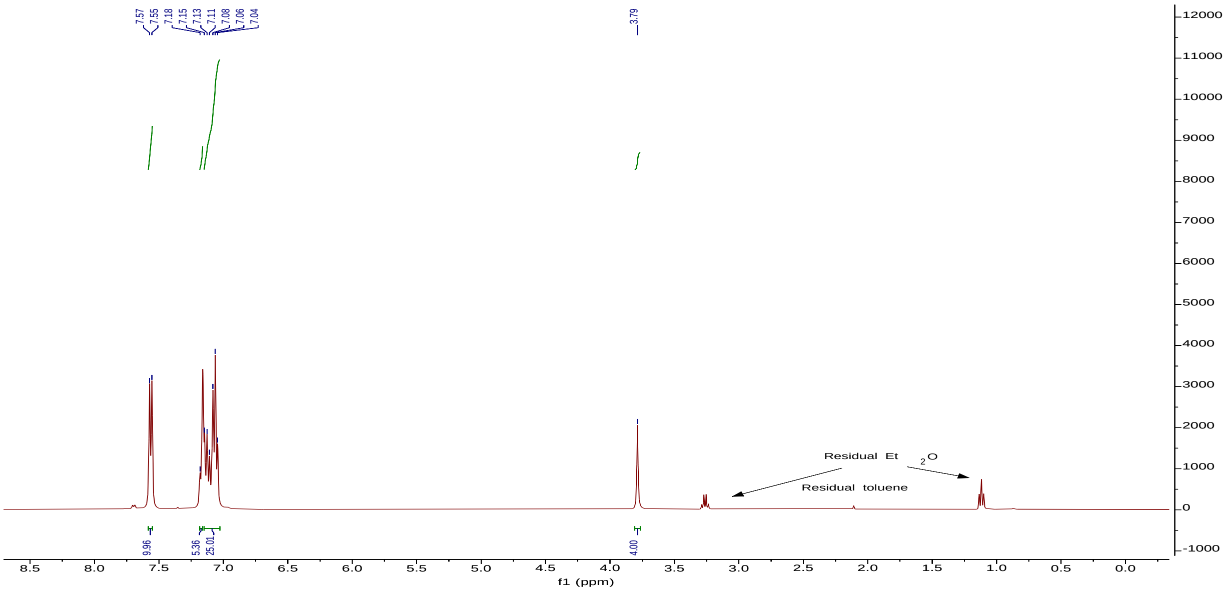

Figure S 1. ^{1}H NMR of **2**, C_6D_6

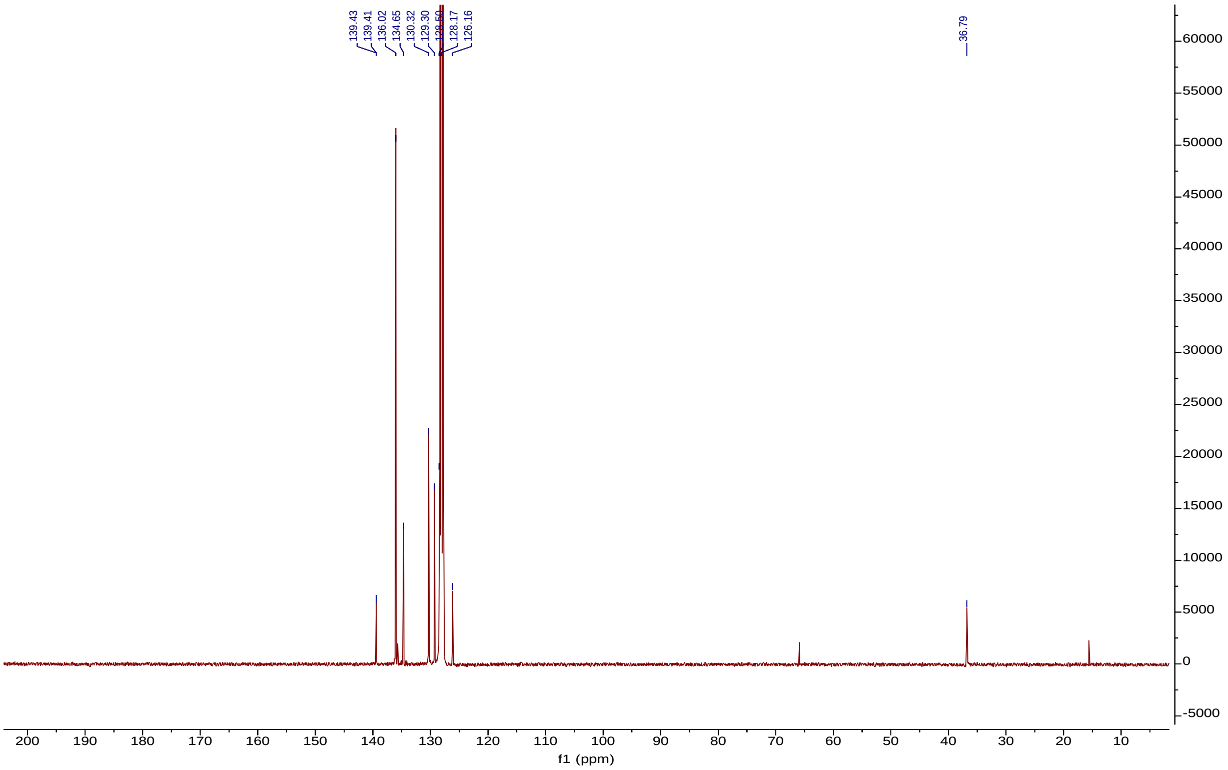

Figure S 2. $^{13}C\{^{1}H\}$ NMR of **2**, C_6D_6

Target Ion Species

Ion Species	*m/z*	*Ionic Formula*
(M+NH4)+	774.3228	C52 H48 N O2 Si2

MFG Calculator Results

Target m/z	*Ionic Formula*	*Calc m/z*	*+/- (mDa)*	*+/- (ppm)*	*DBE*	*MFG Score*
774.3228	C52 H48 N O2 Si2	774.3218	1.0	1.3	33.0	98.22
774.3228	C40 H52 N3 O9 Si2	774.3237	-0.9	-1.2	20.0	96.99
774.3228	C46 H40 N11 Si	774.3232	-0.4	-0.5	35.0	96.52
774.3228	C45 H44 N7 O4 Si	774.3219	0.9	1.2	30.0	95.99
774.3228	C41 H48 N7 O5 Si2	774.3250	-2.2	-2.8	25.0	94.24
774.3228	C49 H48 N O6 Si	774.3245	-1.7	-2.2	29.0	92.63
774.3228	C44 H48 N3 O8 Si	774.3205	2.3	3.0	25.0	91.34
774.3228	C36 H48 N9 O7 Si2	774.3210	1.8	2.3	21.0	90.93
774.3228	C53 H44 N O5	774.3214	1.4	1.8	34.0	90.51
774.3228	C54 H40 N5 O	774.3227	0.1	0.1	39.0	89.85

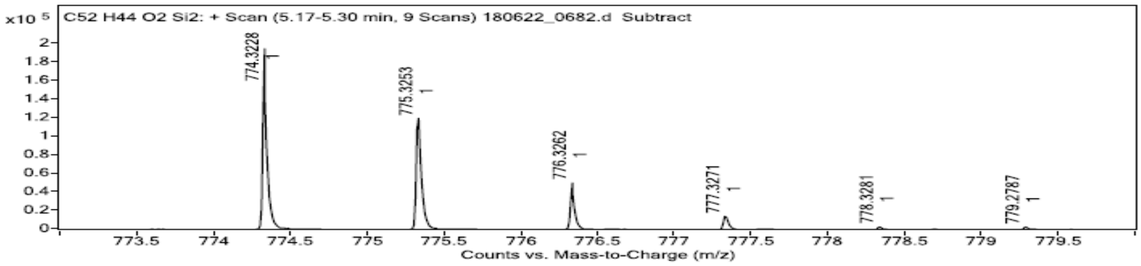

Predicted Isotope Match Table

Isotope	*m/z*	*Calc m/z*	Diff (mDa)	Abund (%)	Calc Abund (%)	+/-
1	774.3228	774.3218	1.0	100.0	100.0	0.0
2	775.3253	775.3246	0.7	62.4	67.4	5.0
3	776.3262	776.3254	0.8	26.2	29.3	3.1
4	777.3271	777.3263	0.8	7.7	9.2	1.5
5	778.3281	778.3273	0.8	1.8	2.2	0.4

Figure S 3. HRMS of **2**, ESI ionization

Formation of ^{13}C-**2** from benzyl potassium **1** with $^{13}CO/ClSiPh_3$

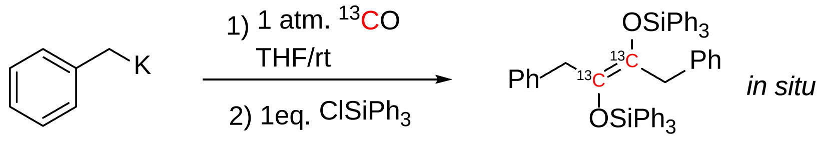

2-^{13}C

Benzyl potassium (9 mg, 0.070 mmol) was dissolved in 0.5 mL THF and transferred to a J-young tube. The solution was freeze-thaw degassed under vacuum, then 1 atm ^{13}CO was added to the head space of the J-young tube while the bottom part was kept frozen in a liquid N_2 bath. The reaction turned from dark red clear to brownish suspension immediately after warming up to room temperature. The suspension was measured with $^{13}C\{^1H\}$ NMR study, no signal was observed other than the solvent THF and residual toluene, indicating the THF insoluble material contains the ^{13}CO incorporated product. $ClSiPh_3$ (20 mg, 0.07 mmol) was added to the J-young tube in the glovebox, a pale-brown slight cloudy solution was formed. The $^{13}C\{^1H\}$ NMR study revealed **2-^{13}C** was the only product incorporated ^{13}CO**.**

$^{13}C\{^1H\}$ NMR (101 MHz, THF): 138.72 (s, **2-^{13}C**).

HRMS (DART ionization, *m/z*): calcd for $^{13}C_2C_{50}H_{45}Si_2O_2$, $[M+H]^+$: 759.30252; found: 759.30139.

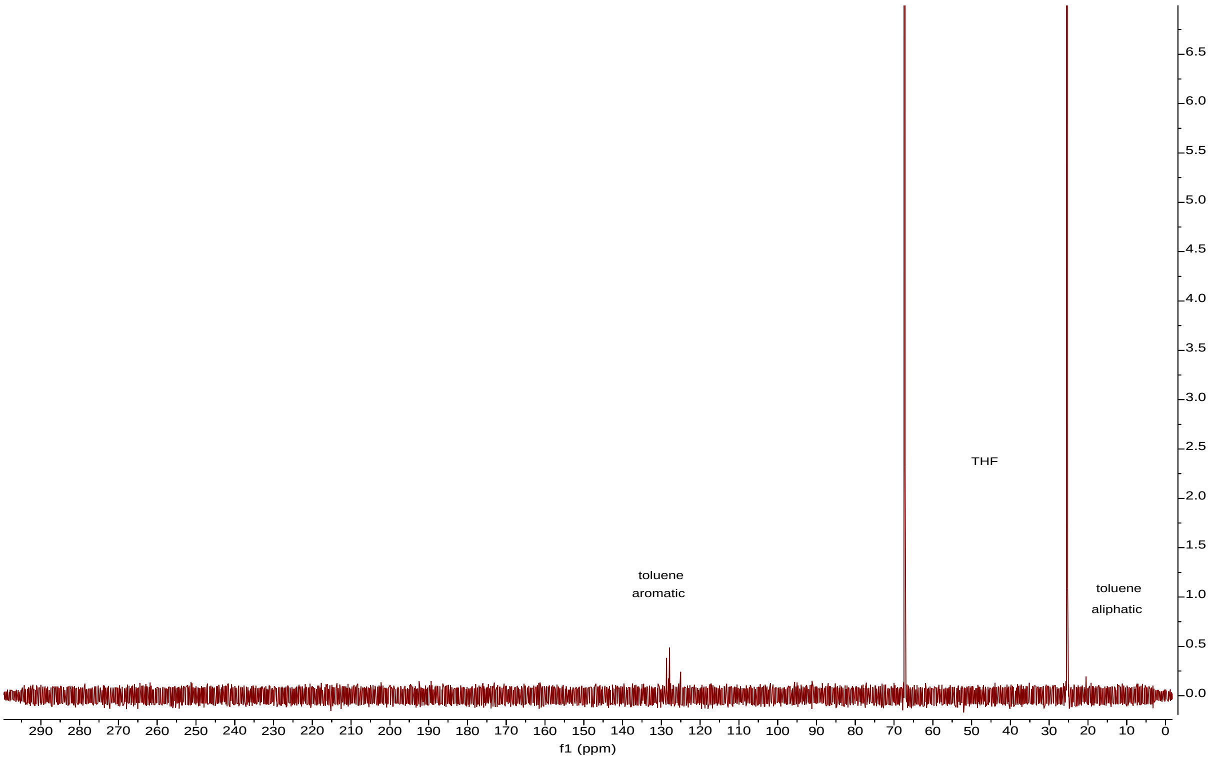

Figure S 4. $^{13}C\{^1H\}$ NMR spectrum, the brownish suspension from **1** and ^{13}CO, THF

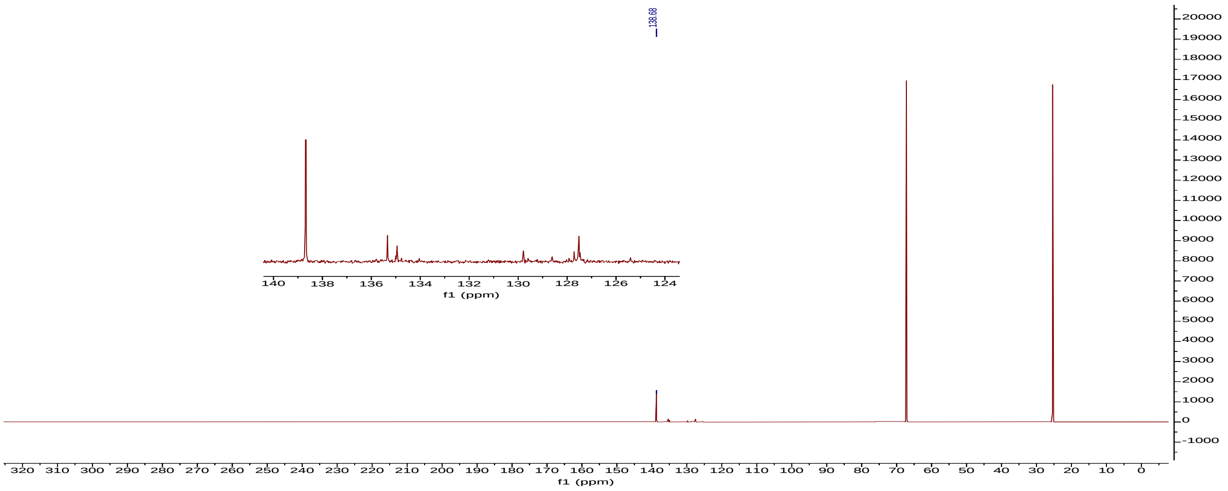

Figure S 5. $^{13}C\{^{1}H\}$ NMR spectrum, crude mixture contains **2-^{13}C** after $ClSiPh_3$ addition, THF

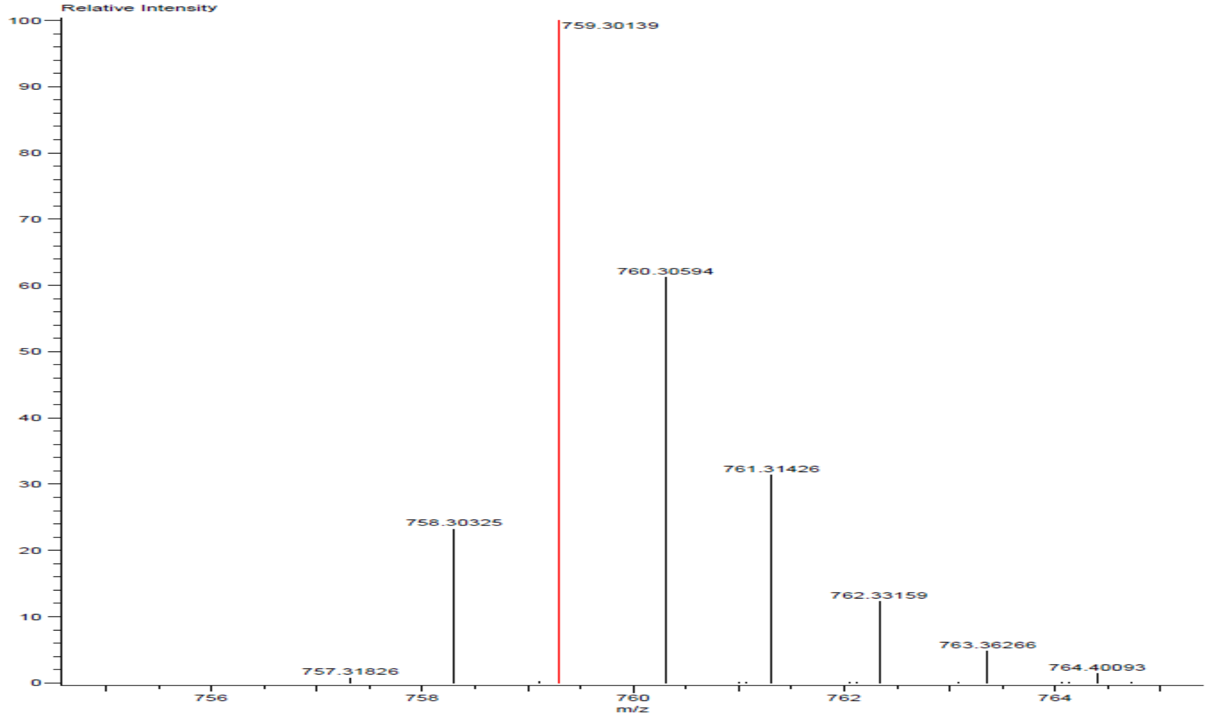

Charge number:1 Tolerance:5.00(mmu) Unsaturation Number:-1.5 .. 40.0 (Frac..

Element:^{12}C:0 .. 100, ^{13}C:0 .. 2, ^{1}H:0 .. 200, ^{16}O:0 .. 10, ^{28}Si:2 .. 2

Mass	Intensity	Calc. Mass	Mass Difference (mmu)	Mass Difference (ppm)	Possible Formula
759.30139	183483.50	759.30207	-0.68	-0.90	$^{12}C_{41}{}^{1}H_{51}{}^{16}O_{10}{}^{28}Si_2$
		759.30252	-1.12	-1.48	$^{12}C_{50}{}^{13}C_2{}^{1}H_{45}{}^{16}O_2{}^{28}Si_2$
		759.29760	3.79	4.99	$^{12}C_{40}{}^{13}C_1{}^{1}H_{50}{}^{16}O_{10}{}^{28}Si_2$

Figure S 6. HRMS of **2-^{13}C**, DART ionization

Formation of **3** from benzyl potassium **1** with CO/H_2O

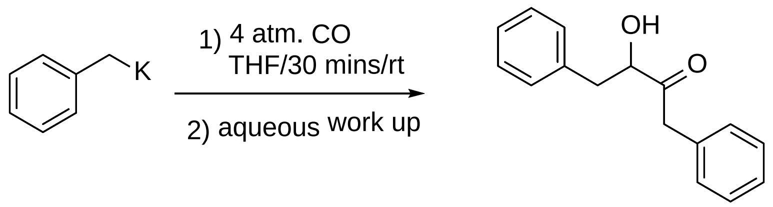

Benzyl potassium (130 mg, 1.0 mmol) was dissolved in 20 mL THF and transferred to a Schlenk bomb. The solution was freeze-thaw degassed under vacuum, then 4 atm CO was added to the Schlenk bomb in a liquid N_2 bath. The reaction turned from dark red clear to red brownish suspension immediately after warming up to room temperature. The reaction was left at room temperature for 30 minutes before excess water (5 mL) was added via syringe, a pale orange-yellow cloudy solution was formed. The reaction was left 1 hour at room temperature, then the volatiles were removed under vacuum. The residue was extract with EtOAc (2*5 mL) and water (10 mL), the organic layer was washed with brine and dried over $MgSO_4$, after removing the volatiles, **3** was obtained as pale-yellow oily solids (118 mg, 98%). The ^{1}H NMR spectrum matches with the previously reported data.[3]

^{1}H NMR (400 MHz, $CDCl_3$) δ 7.35 – 7.13 (m , 10H), 4.51 (dd, *J* = 7.4, 4.7 Hz, 1H), 3.83 – 3.73 (m, 2H), 3.20 (br, 1H, -O*H*), 3.16 (dd, *J* = 14.1, 4.7 Hz, 1H), 2.90 (dd, *J* = 14.1, 7.4 Hz, 1H).

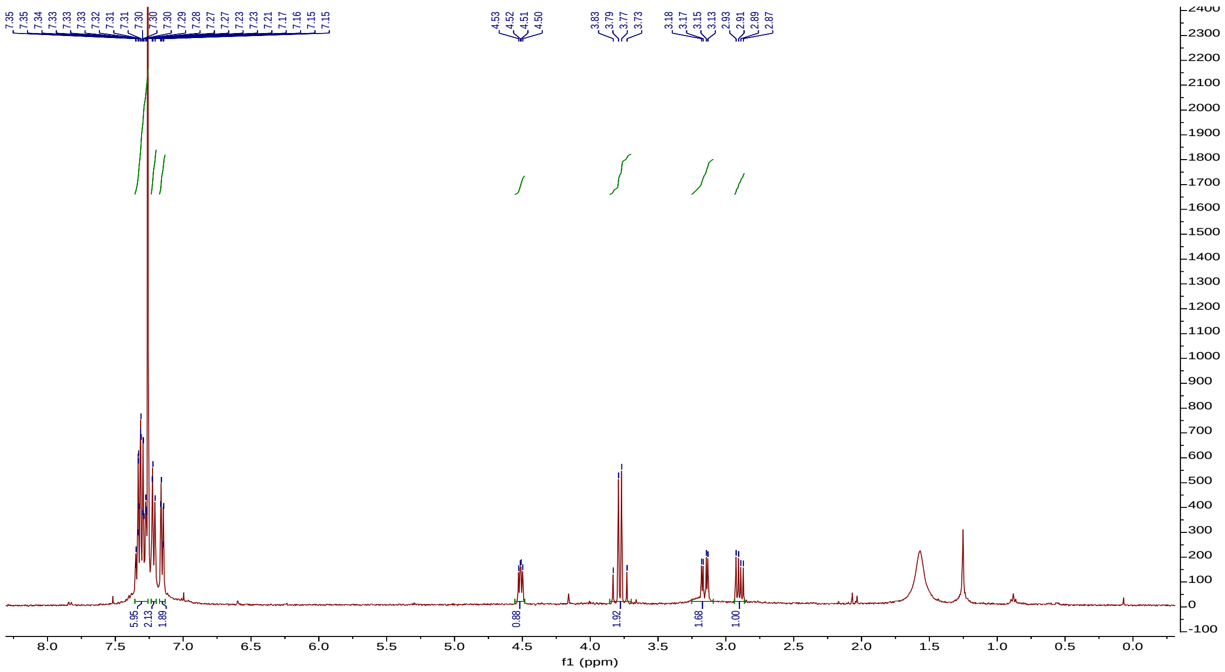

Figure S 7. ^{1}H NMR of **3**, $CDCl_3$

Benzyl potassium **1** with CO at -78°C/1-hexene

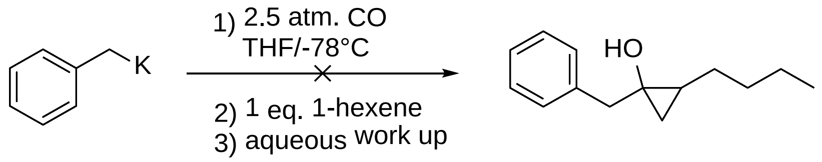

Benzyl potassium (260 mg, 2.0 mmol) was dissolved in 20 mL THF and transferred to a Schlenk bomb. The solution was freeze-thaw degassed under vacuum in a liquid N_2 bath. The Schlenk bomb was transferred to a dry ice acetone bath, then CO was added at this temperature. 1-Hexene (0.25 mL, 2 mmol, 1 eq.) was added to via syringe, and the reaction was slowly warmed up to room temperature overnight. Excess water (5 mL) was added and the reaction was stirred for 30 minutes. The volatiles were removed under vacuum. The residue was extract with EtOAc (2*5 mL) and water (10 mL), the organic layer was washed with brine and dried over $MgSO_4$. The volatiles in the organic layer were removed, ^{1}H NMR spectrum indicated the formation of **3** instead of the cyclopropane derivative.

1,3,5-Tris-*tert*-butyl toluene[4]

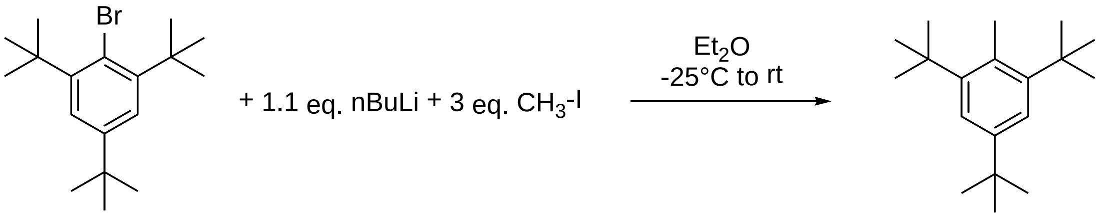

1,3,5-Tris-*tert*-butyl bromobenzene (177 mg, 0.54 mmol, 1 eq) was dissolved in 10 mL Et_2O and cooled to -25°C, *n*BuLi (0.24 mL, 2.5 M, 1.1 eq) was added dropwise to the solution via syringe. The solution was warmed up to room temperature and stirred for 2 hours. Excess methyl iodide (0.1 mL, 1.61 mmol, 3 eq) was added and the mixture was stirred for 3 hours. The volatiles were removed under vacuum, the residue was extracted with pentane. After removing pentane, 1,3,5-tris-*tert*-butyl toluene (133 mg, 94 % yield) was isolated as white crystals.

^{1}H NMR (400 MHz, C_6D_6) δ 7.55 (s, 2H), 2.59 (s, 3H), 1.46 (s, 18H), 1.36 (s, 9H).

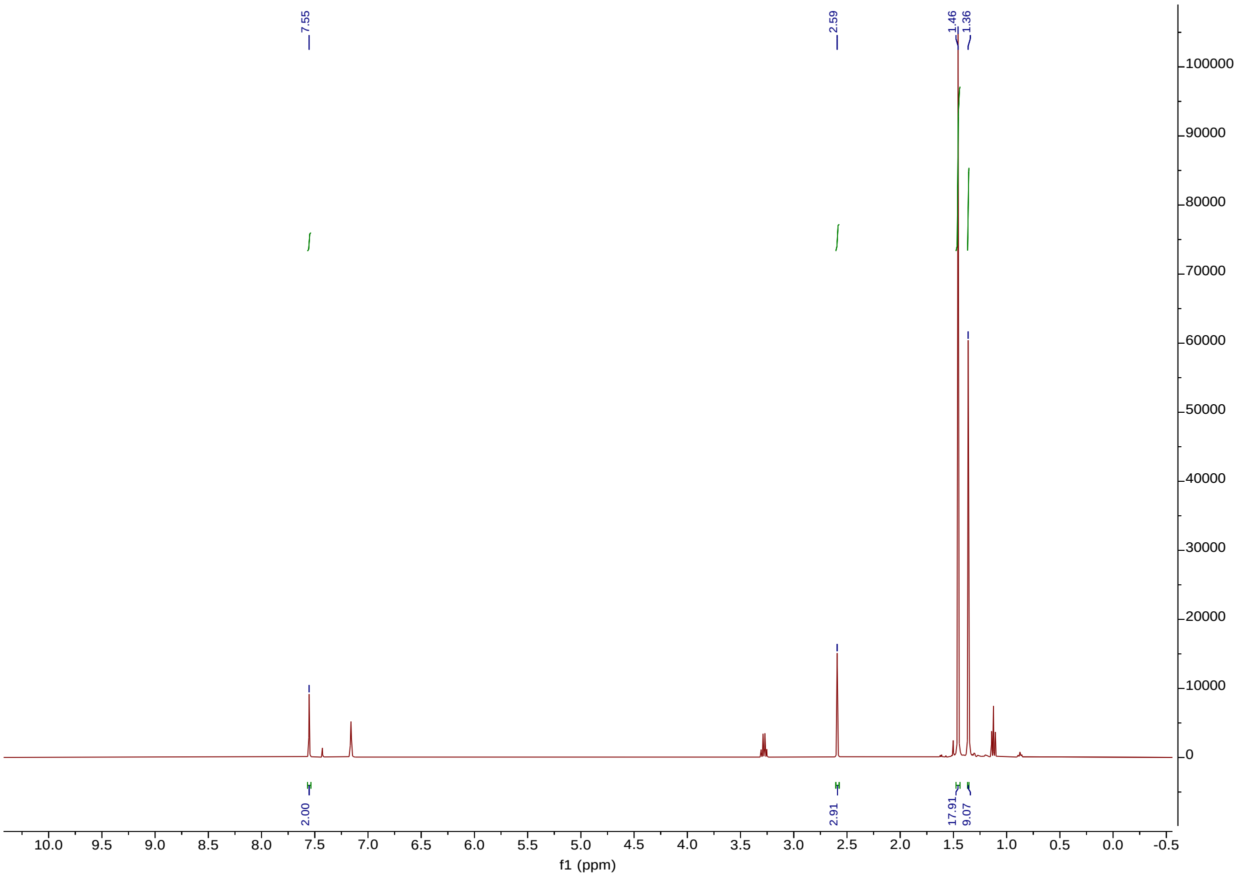

Figure S 8. ^{1}H NMR of 1,3,5-tris-*tert*-butyl toluene, C_6D_6

2,4,6-Tris-*tert*-butyl benzyl potassium **4**

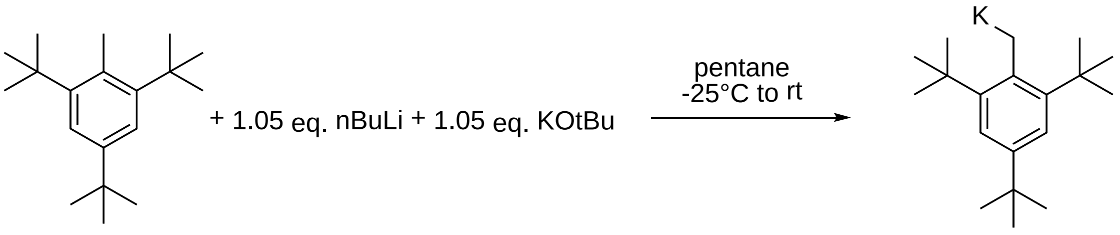

1,3,5-Tris-*tert*-butyl toluene (434 mg, 1.67 mmol, 1 eq) and potassium tert-butoxide (197 mg, 1.75 mmol, 1.05 eq) was suspended in 10 mL pentane and cooled to -25°C, *n*BuLi (0.7 mL, 2.5 M, 1.05 eq) was added dropwise to the solution via syringe. A pale-yellow slurry was formed immediately, the mixture was stirred overnight, resulted in an orange suspension. The orange solids were collected on a frit and washed with cold toluene and pentane. After dried under vacuum, 2,4,6-tris-*tert*-butyl benzyl potassium **4** (411 mg, 83 % yield) was isolated as orange solids.

^{1}H NMR (400 MHz, THF) δ 6.38 (s, 2H), 2.82 (s, 2H), 1.30 (s, 18H), 1.08 (s, 9H).

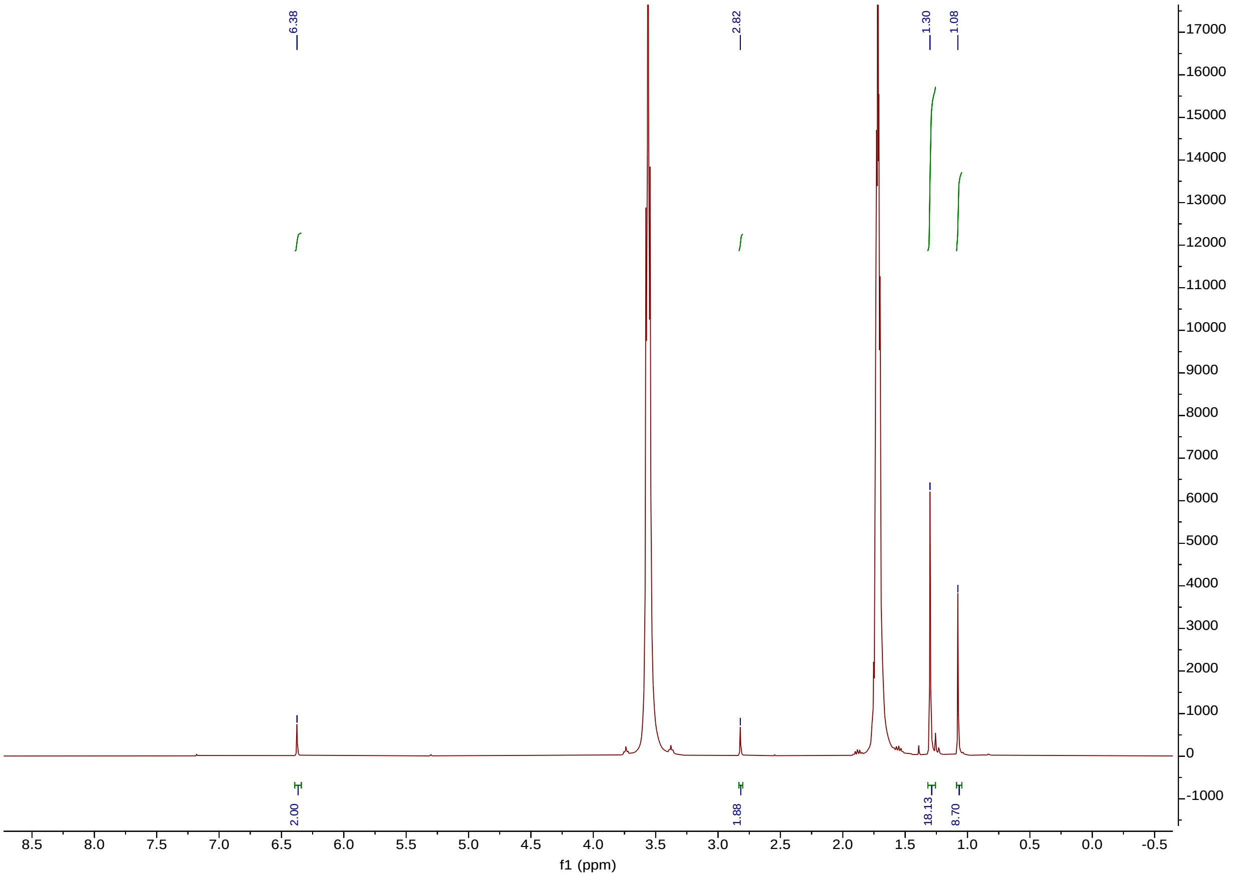

Figure S 9. ^{1}H NMR of 2,4,6-tris-*tert*-butyl benzyl potassium **4**, THF

Formation of **5** and **6** from **4** with $^{13}CO/ClSiMe_3$

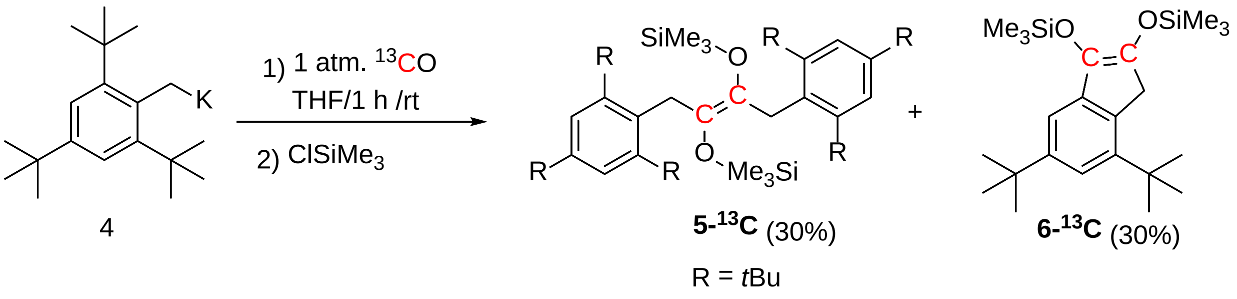

2,4,6-Tris-*tert*-butyl benzyl potassium (10 mg, 0.03 mmol) **4** was dissolved in 0.5 mL THF and transferred to a J-young tube. The solution was freeze-thaw degassed under vacuum, then 1 atm ^{13}CO was added to the head space of J-young tube while the bottom part was kept frozen in a liquid N_2 bath. The reaction turned from orange clear to brownish orange suspension after warming up to room temperature. The reaction was left at room temperature for 1 hour, followed by the addition of $ClSiMe_3$ (4 µL, 0.03 mmol) via micro-syringe, a yellow cloudy solution was formed immediately. The crude mixture contains approximately 1:1 ratio of **5-^{13}C** and **6-^{13}C** based on the integration in the $^{13}C\{^1H\}$ NMR spectrum. The volatiles were removed under vacuum and the residue was extracted with pentane to remove KCl. Pentane was removed under vacuum, the residue was redissolved in diethyl ether, and a drop of toluene was added. The solution was slowly evacuated under weak vacuum, **5-^{13}C** was recrystallized out of solution as colorless crystals (3.6 mg, 30%). Attempts to isolate crystals of **6-^{13}C** were unsuccessful, yellow oil was obtained instead.

$^{13}C\{^1H\}$ NMR of crude reaction mixture containing **5-^{13}C** and **6-^{13}C** (151 MHz, THF) δ 137.45 (d, J = 94.0 Hz, 0.5 ^{13}C, from **6-^{13}C**), 136.15 (s, 1 ^{13}C, from **5-^{13}C)**, 132.75 (d, J = 94.0 Hz, 0.5 ^{13}C, from **6-^{13}C**).

^1H NMR of **5-^{13}C** (500 MHz, C_6D_6) δ 7.58 (s, 4H), 4.40 (s, 4H), 1.60 (s, 36H), 1.40 (s, 18H), -0.03 (s, 18H).

$^{13}C\{^1H\}$NMR of **5-^{13}C** (126 MHz, C_6D_6) δ 150.32, 147.38, 136.64, 133.24, 122.51, 37.62, 35.07, 33.39, 31.75, 30.25, 2.26.

HRMS of **5-^{13}C** (ESI ionization, *m/z*): calcd for $C_{44}{}^{13}C_2H_{80}O_2Si_2$, $[M]^+$: 722.57858; found: 722.57549.

HRMS of **6-^{13}C** (ESI ionization, *m/z*): calcd for $C_{21}{}^{13}C_2H_{41}O_2Si_2$, $[M+H]^+$: 407.27067; found: 407.26969.

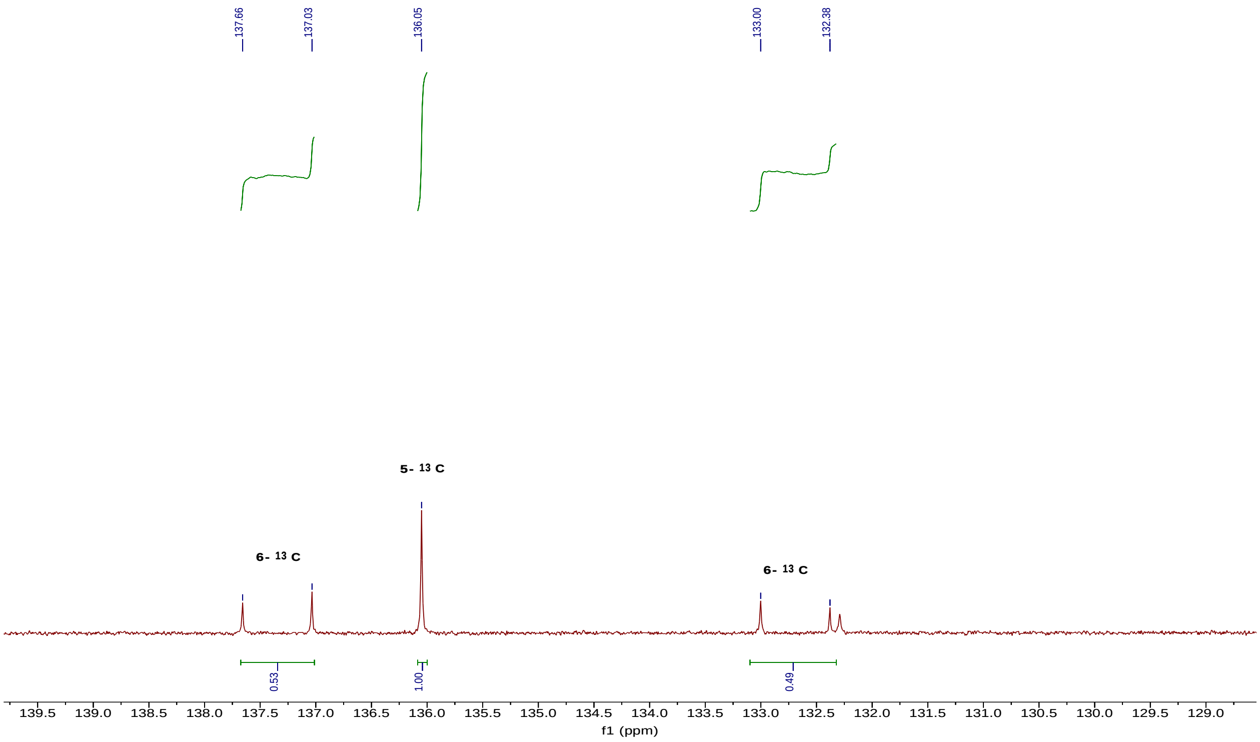

Figure S 10. $^{13}C\{^{1}H\}$ NMR of crude reaction mixture containing **5-^{13}C** and **6-^{13}C**, THF

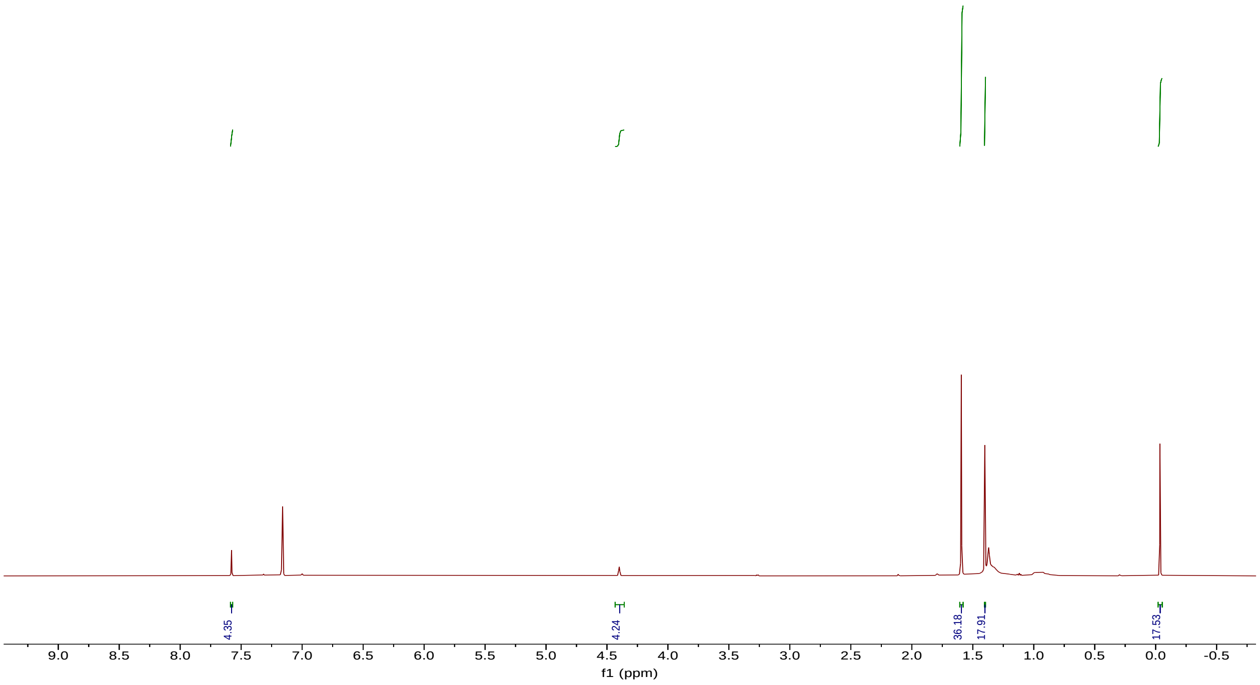

Figure S 11. ^{1}H NMR spectrum of **5-^{13}C**, C_6D_6

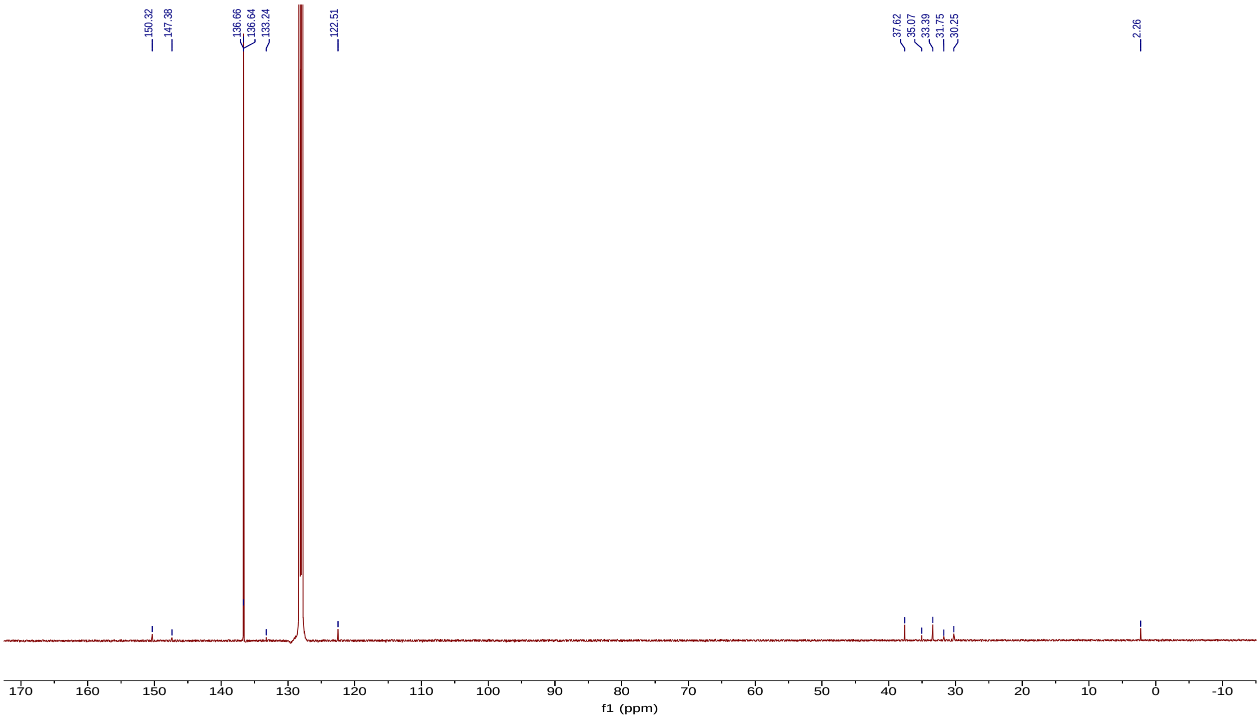

Figure S 12. $^{13}C\{^{1}H\}$ NMR of **5-^{13}C**, C_6D_6

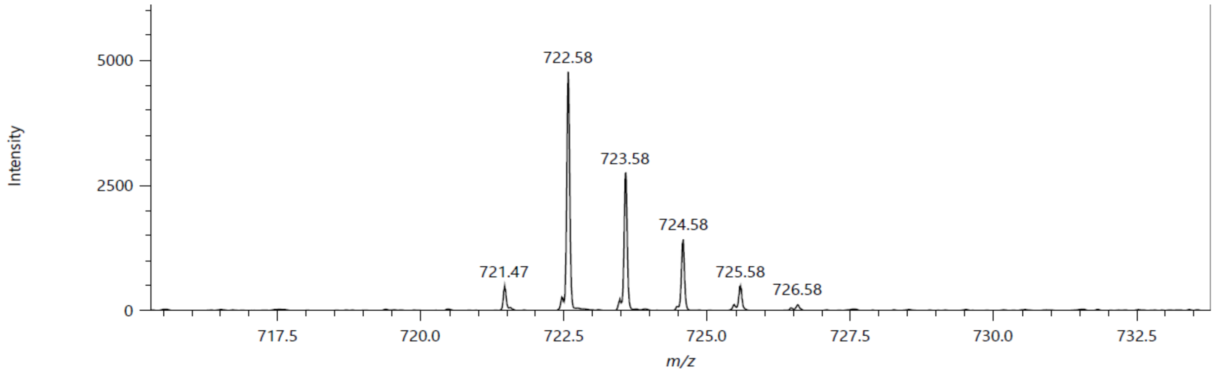

Elemental Composition

Parameters		Elements Set 1:						
Tolerance:	±10.00 mDa	Symbol	C	H	O	N	Cx	Si
Electron:	Odd/Even	Min	0	0	0	0	2	2
Charge:	+1	Max	100	100	10	10	2	2
DBE:	-1.5 - 100.0							

Results

Mass	Intensity	Formula	Calculated Mass	Mass Difference [mDa]	Mass Difference [ppm]	DBE
722.57549	4774.51	C44 H80 O2 Si2 Cx2	722.57585	-0.35	-0.49	9.0
		C42 H78 N3 O Si2 Cx2	722.57450	0.99	1.37	9.5
		C31 H78 N9 O4 Si2 Cx2	722.57769	-2.20	-3.04	1.5
		C40 H76 N6 Si2 Cx2	722.57316	2.33	3.23	10.0
		C33 H80 N6 O5 Si2 Cx2	722.57903	-3.54	-4.90	1.0
		C39 H80 N2 O4 Si2 Cx2	722.57182	3.67	5.08	5.0

Figure S 13. HRMS of **5-^{13}C**, DART ionization

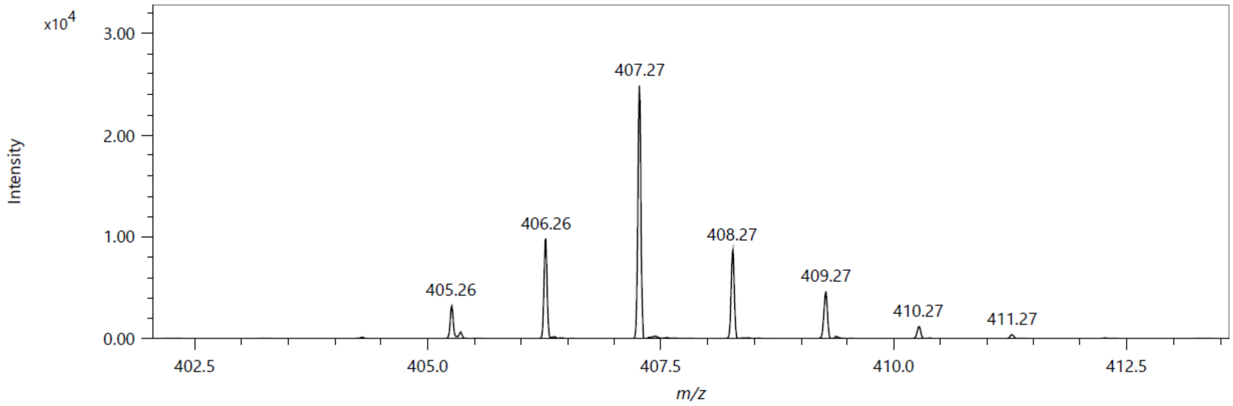

Elemental Composition

Parameters		Elements Set 1:						
Tolerance:	±10.00 mDa	Symbol	C	H	O	N	Cx	Si
Electron:	Odd/Even	Min	0	0	0	0	2	2
Charge:	+1	Max	100	100	10	10	2	2
DBE:	-1.5 - 100.0							

Results

Mass	Intensity	Formula	Calculated Mass	Mass Difference [mDa]	Mass Difference [ppm]	DBE
407.26969	24870.71	C19 H39 N3 O Si2 Cx2	407.26933	0.36	0.89	6.0
		C21 H41 O2 Si2 Cx2	407.27067	-0.98	-2.40	5.5
		C17 H37 N6 Si2 Cx2	407.26798	1.71	4.19	6.5
		C16 H41 N2 O4 Si2 Cx2	407.26665	3.04	7.47	1.5
		C14 H39 N5 O3 Si2 Cx2	407.26530	4.39	10.77	2.0
		C11 H37 N10 O Si2 Cx2	407.27520	-5.50	-13.51	2.5

Figure S 14. HRMS of **6-^{13}C**, DART ionization

Formation of **7-^{13}C** from **4** with $^{13}CO/ClSiPh_3$

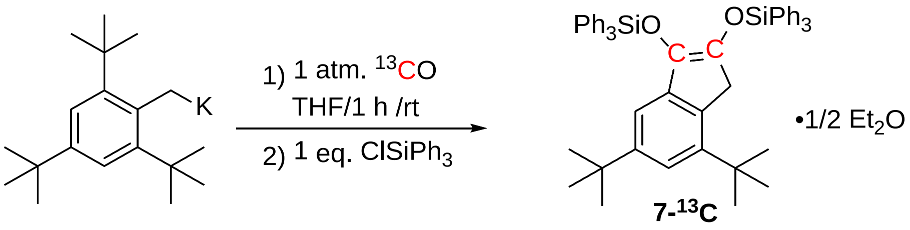

2,4,6-Tris-*tert*-butyl benzyl potassium **4** (10 mg, 0.03 mmol) was dissolved in 0.5 mL THF and transferred to a J-young tube. The solution was freeze-thaw degassed under vacuum, then 1 atm ^{13}CO was added to the head space of J-young tube while the bottom part was kept frozen in a liquid N_2 bath. The reaction turned from orange clear to brownish orange suspension after warming up to room temperature. The reaction was left at room temperature for 1 hour, followed by the addition of $ClSiPh_3$ (10 mg, 0.03 mmol), a yellow cloudy solution was formed after 3 hours. The volatiles were removed under vacuum and the residue was extracted with pentane. The pentane solution was stored at a -25°C freezer, colorless crystals of **7-^{13}C**·0.5 Et_2O was obtained (7.1 mg, 52% yield).

^{1}H NMR (400 MHz, C_6D_6) δ 7.92 – 7.87 (m, 6H), 7.70 – 7.64 (m, 10H), 7.41 (dd, J = 3.9, 1.8 Hz, 1H), 7.23 (d, J = 1.8 Hz, 1H), 7.19– 7.05 (m, 14H), 3.27 (q, J = 7.0 Hz, 2H, Et_2O), 3.13 (dd, J = 7.7, 4.4 Hz, 2H), 1.25 (s, 9H), 1.11 (s, 9H), 1.11 (t, J = 7.0 Hz, 3H, Et_2O)

$^{13}C\{^{1}H\}$NMR (101 MHz, C_6D_6) δ 138.99 (d, J = 96 Hz), 136.26, 136.11, 135.97, 135.70, 135.47, 135.05, 134.30, 133.25 (d, J = 96 Hz), 130.53, 130.33, 130.21, 35.34 (d, J = 85 Hz), 31.79, 30.94. Not all the expected signals were observed.

HRMS (ESI ionization, *m/z*): calcd for $C_{51}{}^{13}C_2H_{53}O_2Si_2$, $[M+H]^+$: 779.3646; found: 779.3642.

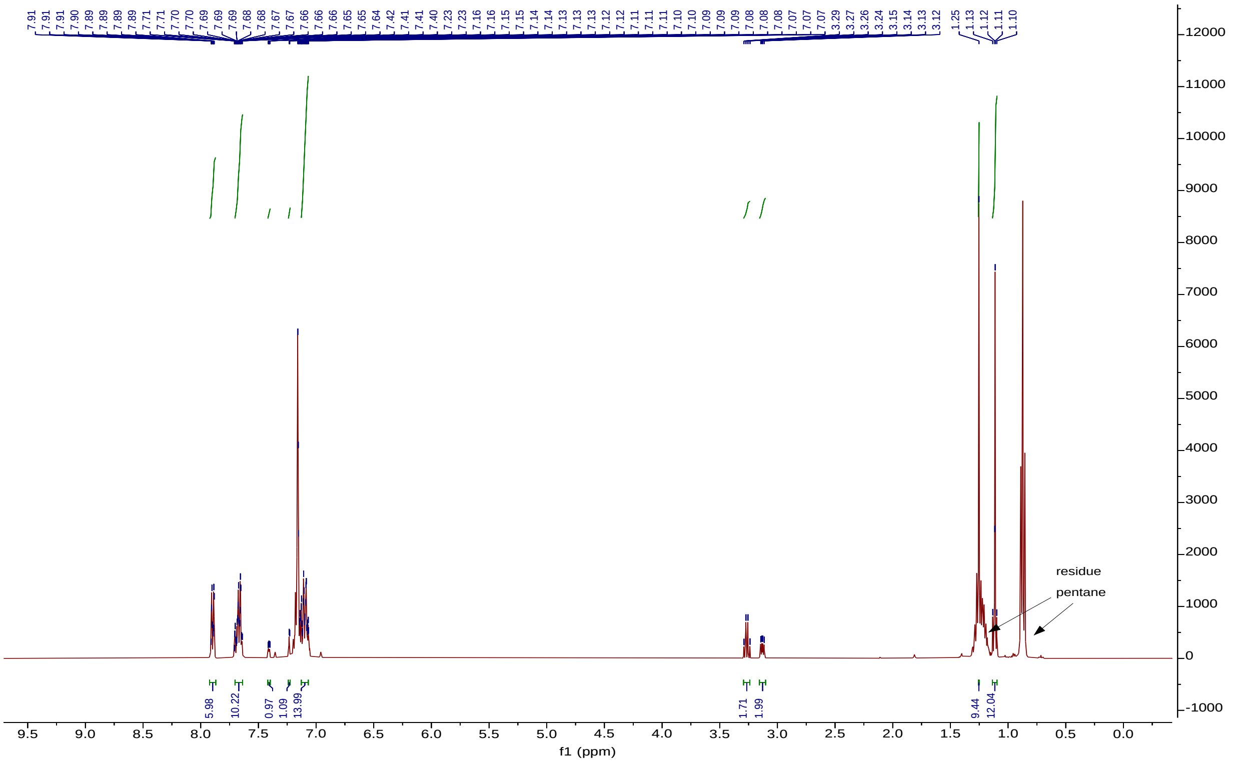

Figure S 15. ^{1}H NMR of **7-^{13}C**·0.5 Et_2O, C_6D_6

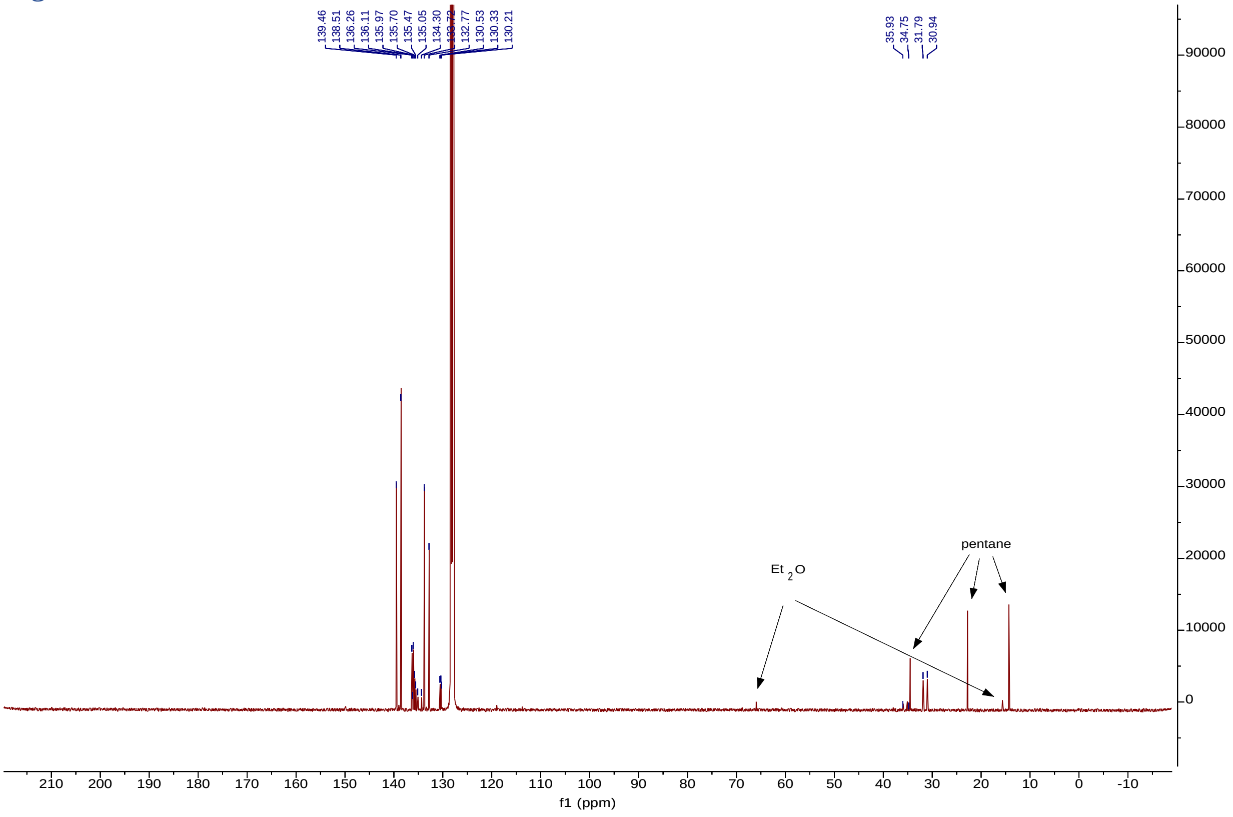

Figure S 16. $^{13}C\{^{1}H\}$ NMR of **7-^{13}C**·0.5 Et_2O, C_6D_6

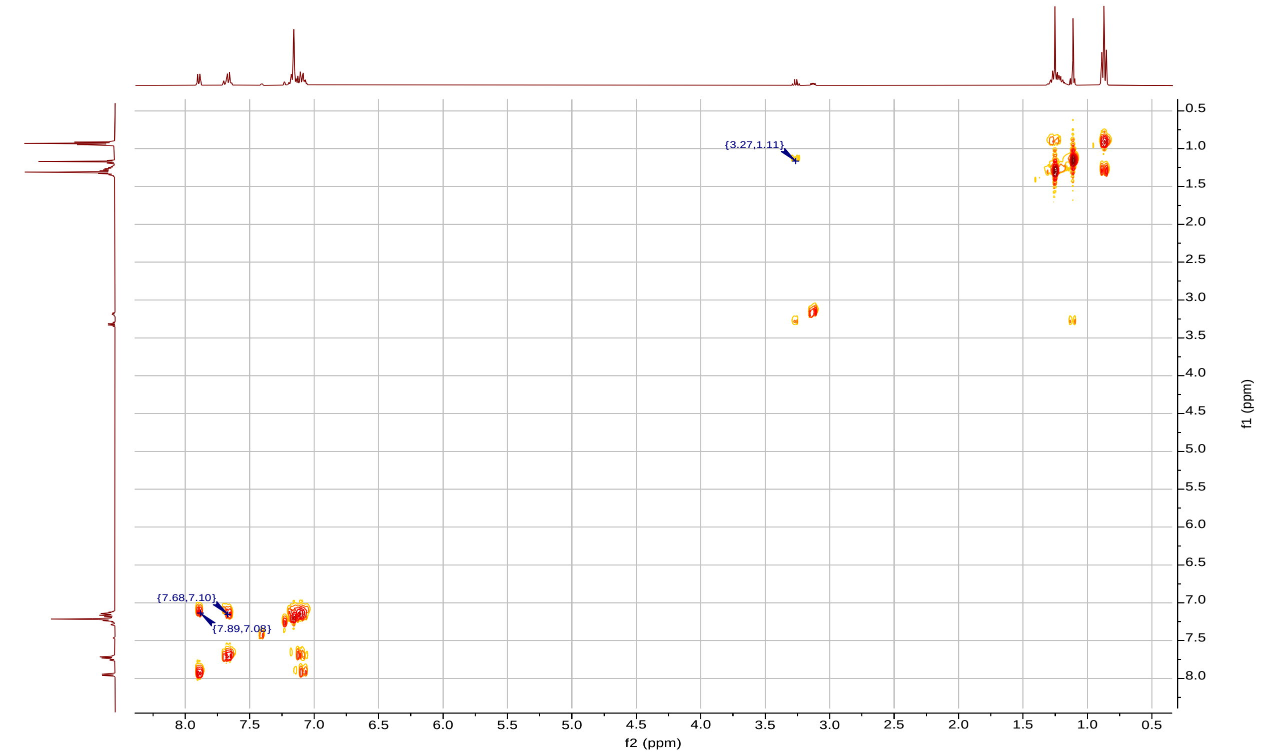

Figure S 17. gCOSY spectrum of **7-^{13}C**·0.5 Et_2O, C_6D_6

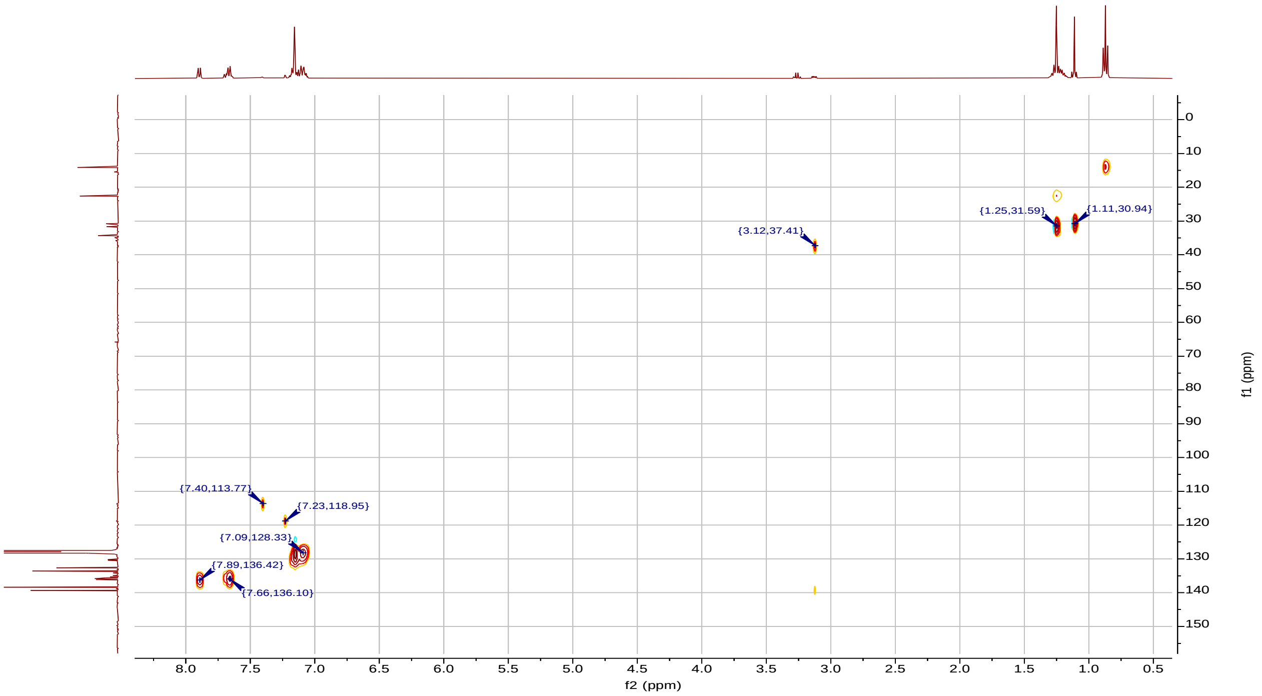

Figure S 18. HSQC spectrum of **7-^{13}C**·0.5 Et_2O, C_6D_6

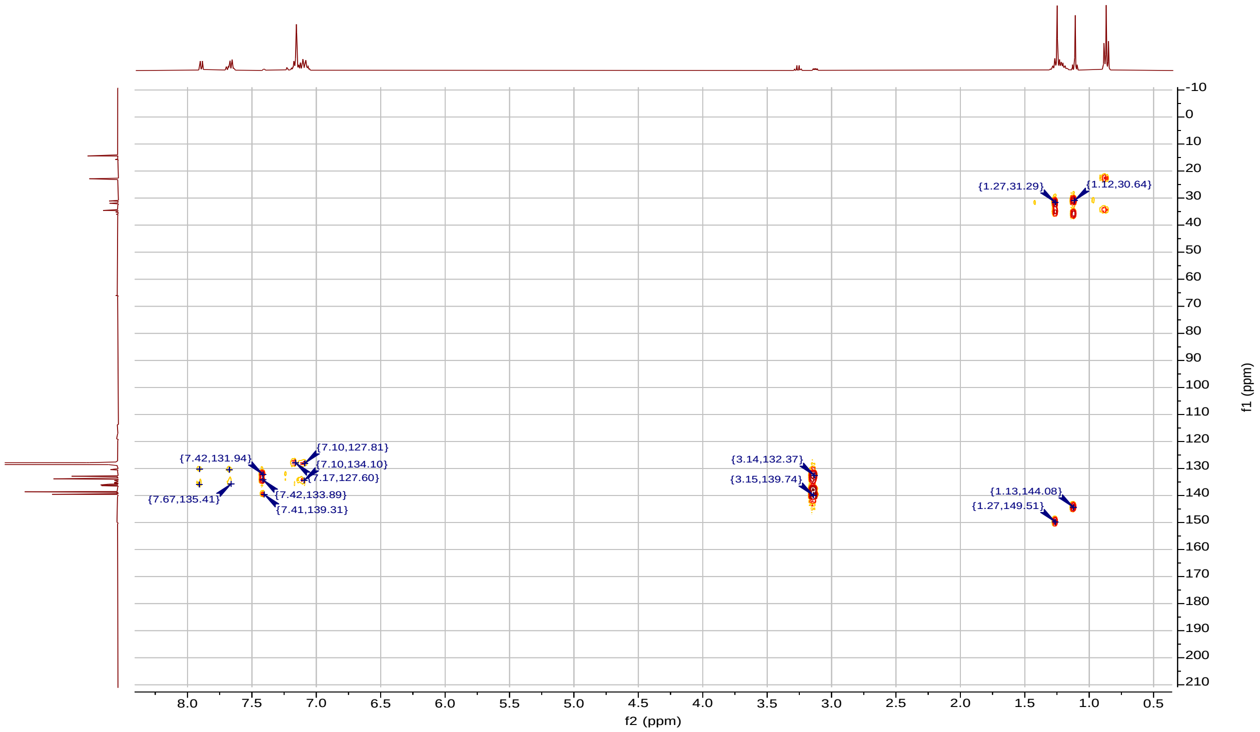

Figure S 19. HMBC spectrum of **7-^{13}C**·0.5 Et_2O, C_6D_6

Target Ion Species

Ion Species	m/z	Ionic Formula
(M+H)+	779.3642	C51 [13C]2 H53 O2 Si2

MFG Calculator Results

Target m/z	Ionic Formula	Calc m/z	+/- (mDa)	+/- (ppm)	DBE	MFG Score
779.3642	C51 [13C]2 H53 O2 Si2	779.3646	-0.4	-0.5	30.0	99.00
779.3642	C52 H52 F N2 Si2	779.3648	-0.6	-0.8	30.0	98.67
779.3642	C45 [13C] H52 N5 O3 Si2	779.3637	0.5	0.6	26.0	96.92
779.3642	C43 H55 N4 O6 Si2	779.3655	-1.3	-1.7	21.0	96.25
779.3642	C42 [13C] H53 F N5 O4 Si2	779.3648	-0.6	-0.8	22.0	96.24
779.3642	C42 H59 O10 Si2	779.3641	0.1	0.1	16.0	96.10
779.3642	C42 H50 F3 N8 Si2	779.3644	-0.2	-0.3	23.0	95.77
779.3642	C46 [13C]2 H53 F4 O Si2	779.3633	0.9	1.2	23.0	95.57
779.3642	C43 [13C]2 H54 F5 O2 Si2	779.3644	-0.2	-0.3	19.0	95.50
779.3642	C41 [13C]2 H51 F2 N6 O2 Si2	779.3642	0.0	0.0	23.0	94.96

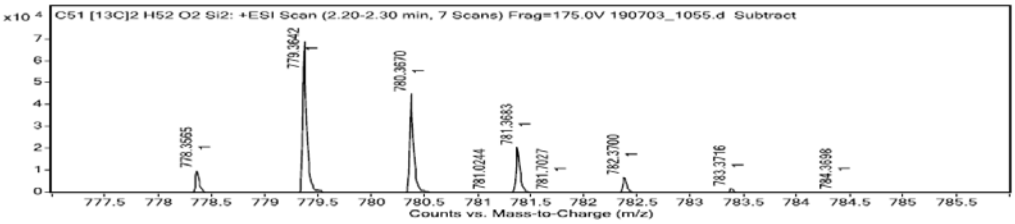

Predicted Isotope Match Table

Isotope	m/z	Calc m/z	Diff (mDa)	Abund (%)	Calc Abund (%)	+/-
1	779.3642	779.3646	-0.4	100.0	100.0	0.0
2	780.3670	780.3674	-0.4	65.7	66.0	0.3
3	781.3683	781.3681	0.2	30.5	28.3	-2.2
4	782.3700	782.3691	0.9	10.4	8.8	-1.6
5	783.3716	783.3701	1.5	2.8	2.1	-0.7

Figure S 20. HRMS of **7-^{13}C**·0.5 Et_2O, ESI ionization

Formation of **7** from **4** with CO/ClSiPh3

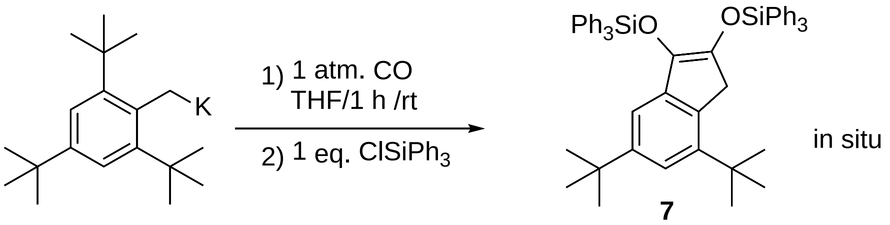

2,4,6-Tris-*tert*-butyl benzyl potassium (10 mg, 0.03 mmol) **4** was dissolved in 0.5 mL THF and transferred to a J-young tube. The solution was freeze-thaw degassed under vacuum, then 1 atm CO was added to the head space of J-young tube while the bottom part was kept frozen in a liquid N_2 bath. The reaction turned from orange clear to brownish orange suspension after warming up to room temperature. The reaction was left at room temperature for 1 hour, followed by the addition of $ClSiPh_3$ (10 mg, 0.03 mmol), The reaction was stirred at room temperature, the crude mixture was submitted for HRMS study.

HRMS (DART ionization, *m/z*): calcd for $C_{53}H_{53}O_2Si_2$, $[M+H]^+$: 777.35786; found: 777.35761.

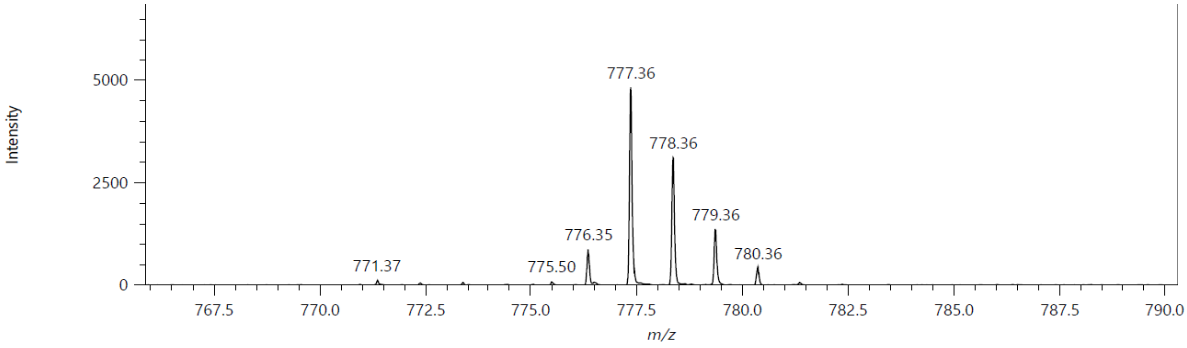

Elemental Composition

Parameters	
Tolerance:	±10.00 mDa
Electron:	Odd/Even
Charge:	+1
DBE:	-1.5 - 100.0

Elements Set 1:

Symbol	C	H	O	N	Si
Min	0	0	0	0	2
Max	100	100	10	10	2

Results

Mass	Intensity	Formula	Calculated Mass	Mass Difference [mDa]	Mass Difference [ppm]	DBE
777.35761	4832.51	C53 H53 O2 Si2	777.35786	-0.25	-0.32	29.5
		C37 H53 N8 O7 Si2	777.35703	0.59	0.76	17.5
		C39 H55 N5 O8 Si2	777.35837	-0.75	-0.97	17.0
		C51 H51 N3 O Si2	777.35652	1.10	1.41	30.0
		C40 H51 N9 O4 Si2	777.35971	-2.09	-2.69	22.0
		C41 H57 N2 O9 Si2	777.35971	-2.10	-2.70	16.5

Figure S 21. HRMS of **7**, DART ionization

Formation of **8** and **9** from **4** with CO/degassed water

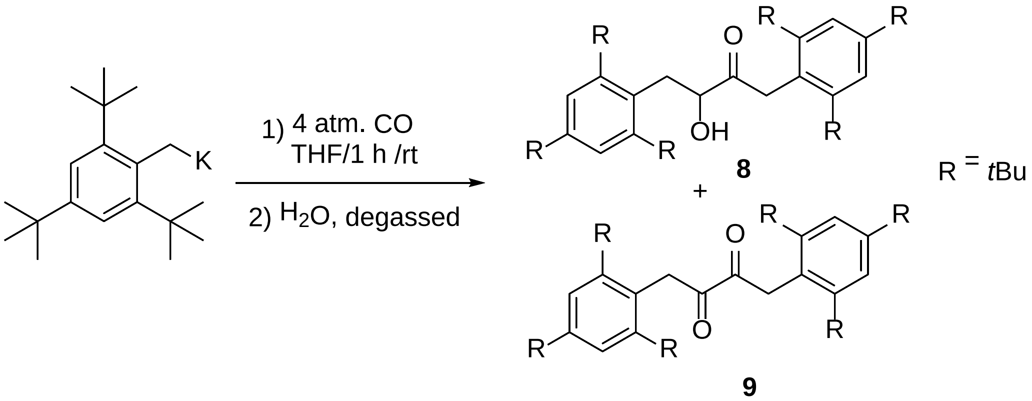

2,4,6-Tris-*tert*-butyl benzyl potassium (100 mg, 0.34 mmol) **4** was dissolved in 5 mL THF and transferred to a 100 mL Schlenk bomb. The Schlenk bomb was freeze-thaw degassed under vacuum, then 4 atm CO was added in a liquid N_2 bath. The reaction was left at room temperature for 1 hour, degassed water (1 mL) was added to Schlenk bomb. Volatiles were removed under vacuum, the residue was extract with pentane (2*10 mL), afforded a mixture of **8** and **9** (73.1 mg, 76% yield) in approximately 9:1 ratio. Recrystallization in pentane at -25°C afforded small amount of **8** as colorless crystals.

^{1}H NMR (500 MHz, $CDCl_3$) of **8**: δ 7.42 – 7.35 (m, 4H), 4.78 – 4.60 (m, 2H), 4.29 (dt, *J* = 11.6, 3.1 Hz, 1H), 3.83 (dd, *J* = 14.7, 3.3 Hz, 1H), 3.54 (dd, *J* = 14.8, 11.6 Hz, 1H), 1.65 (d, *J* = 2.5 Hz, 1H), 1.47 (s, 18H), 1.39 (s, 9H), 1.35 (s, 9H), 1.32 (s, 9H, overlapped), 1.31 (s, 9H).

13C{^{1}H} NMR (126 MHz, $CDCl_3$) of **8**: δ 210.89, 148.93, 148.78, 147.86, 147.23, 132.50, 128.52, 122.35, 122.27, 79.62, 43.22, 36.77, 35.10, 34.88, 33.97, 33.80, 32.91, 32.84, 31.64, 31.57.

HRMS (DART ionization, *m/z*) of **8**: calcd for $C_{40}H_{68}O_2N$, $[M+NH_4]^+$: 594.52446; found: 594.52355.

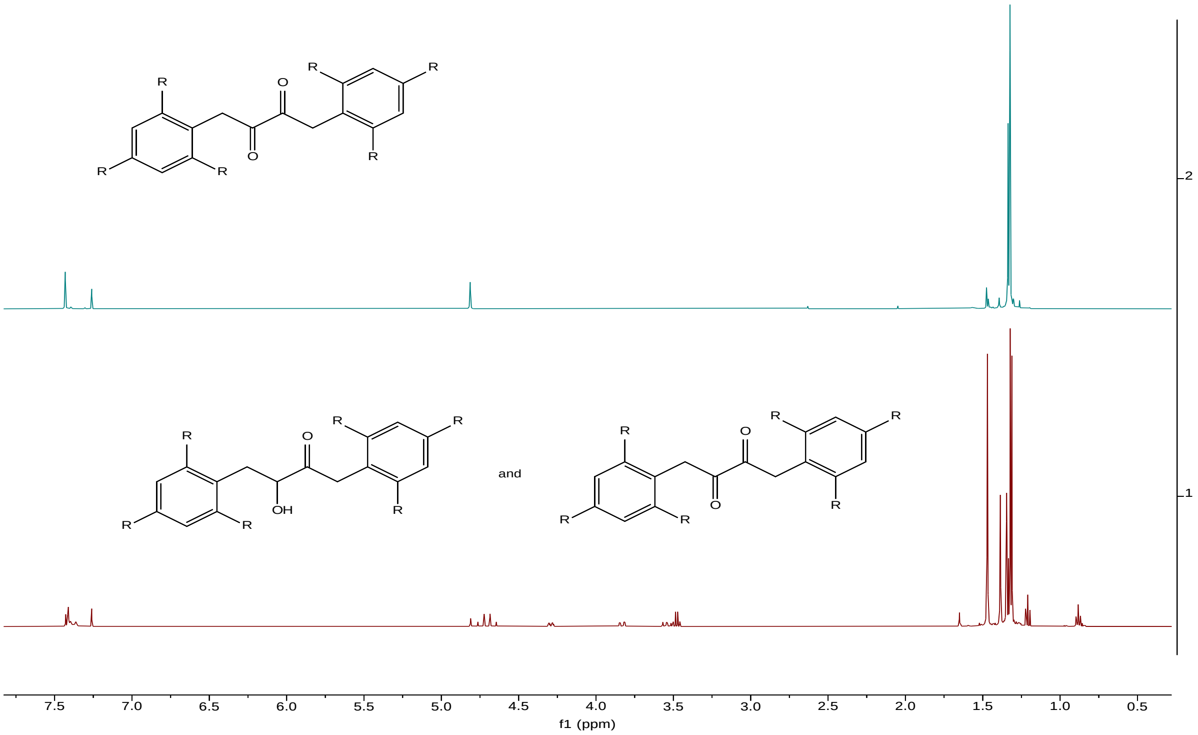

Figure S 22. ^{1}H NMR spectra: **9** (top); mixture of **8** and **9** (bottom), $CDCl_3$

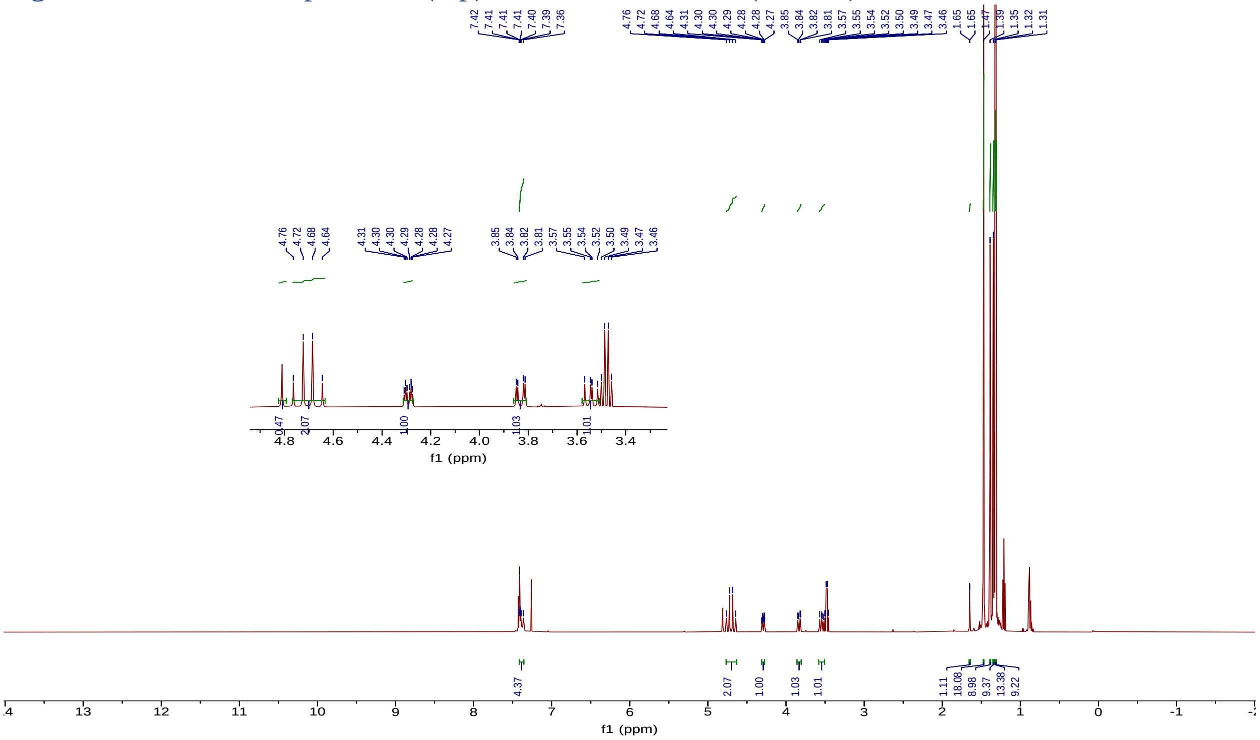

Figure S 23. ^{1}H NMR spectrum of **8**, $CDCl_3$

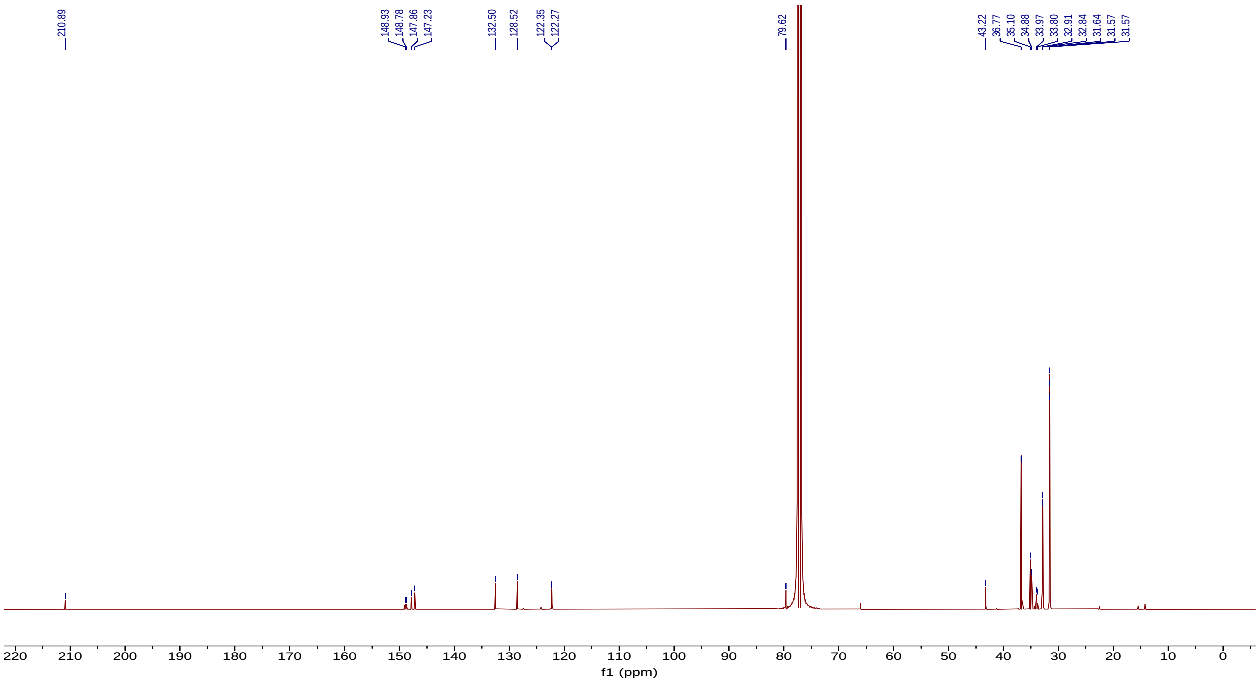

Figure S 24. $^{13}C\{^{1}H\}$ NMR of **8**, $CDCl_3$

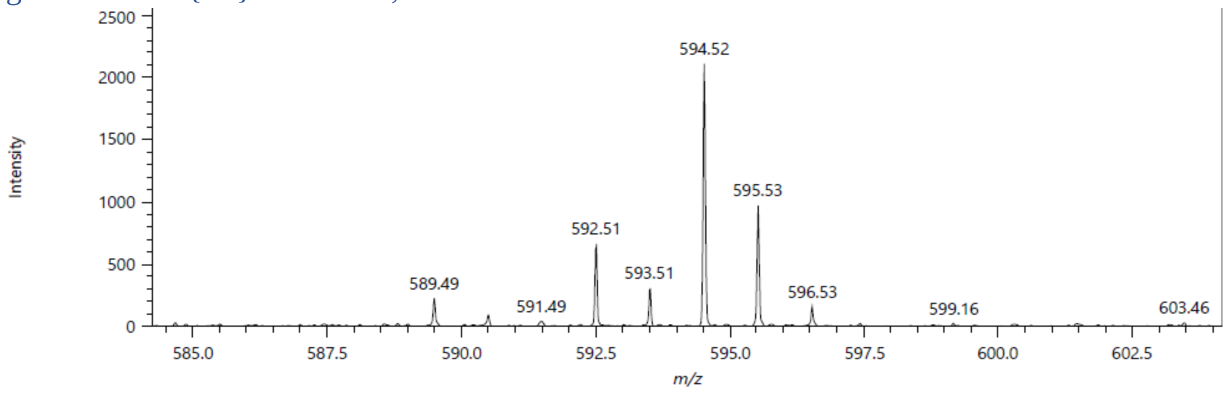

Elemental Composition

Parameters	
Tolerance:	±10.00 mDa
Electron:	Odd/Even
Charge:	+1
DBE:	-1.5 - 100.0

Elements Set 1:

Symbol	C	H	O	N
Min	0	0	0	0
Max	100	100	10	10

Results

Mass	Intensity	Formula	Calculated Mass	Mass Difference [mDa]	Mass Difference [ppm]	DBE
594.52355	2108.92	C38 H66 N4 O	594.52311	0.44	0.74	8.0
		C40 H68 N O2	594.52446	-0.90	-1.52	7.5
		C37 H70 O5	594.52178	1.78	2.99	3.0
		C36 H64 N7	594.52177	1.78	3.00	8.5
		C27 H66 N10 O4	594.52630	-2.75	-4.62	0.0
		C35 H68 N3 O4	594.52043	3.12	5.25	3.5

Figure S 25. HRMS of **8**, DART ionization

Formation of **9** from **4** with CO/aqueous work in air

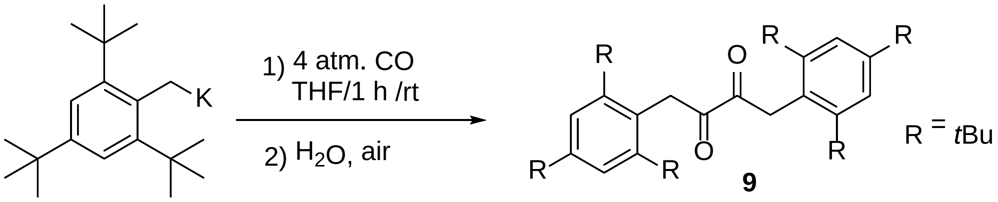

2,4,6-Tris-*tert*-butyl benzyl potassium (10 mg, 0.03 mmol) **4** was dissolved in 0.5 mL THF and transferred to a J-young tube. The solution was freeze-thaw degassed under vacuum, then 4 atm CO was added to J-young tube in a liquid N_2 bath. The reaction was left at room temperature for 1 hour, then water (0.5 mL) was added to the J-young tube. The solution in the J-young tube was poured into a vial and stirred overnight, then the volatiles were removed under vacuum. The residue was extract with EtOAc (5 mL) and water (5 mL), the organic layer was washed with brine and dried over $MgSO_4$. The EtOAc solution was concentrated and stored in a -10°C freezer, **9** was recrystallized as yellow crystals (6 mg, 62% yield).

^{1}H NMR (500 MHz, $CDCl_3$) δ 7.43 (s, 4H), 4.81 (s, 4H), 1.34 (s, 18H), 1.32 (s, 36H).

^{13}C{^{1}H} NMR (126 MHz, $CDCl_3$) δ 199.47, 149.06, 148.32, 127.48, 122.36, 41.28, 36.68, 35.14, 32.86, 31.61.

^{13}C NMR (126 MHz, $CDCl_3$) δ 199.47 (t, *J* = 7 Hz), 149.07 (m), 148.34 (m), 127.48 (m), 122.39 (dd, *J* = 153, 7 Hz), 41.28 (t, *J* = 125 Hz), 36.68 (m), 35.12 (m), 32.86 (q, *J* = 126 Hz), 31.61 (q, *J* = 126 Hz).

HRMS (DART ionization, *m/z*): calcd for $C_{40}H_{66}O_2N$, $[M+NH_4]^+$: 592.50881; found: 592.50875.

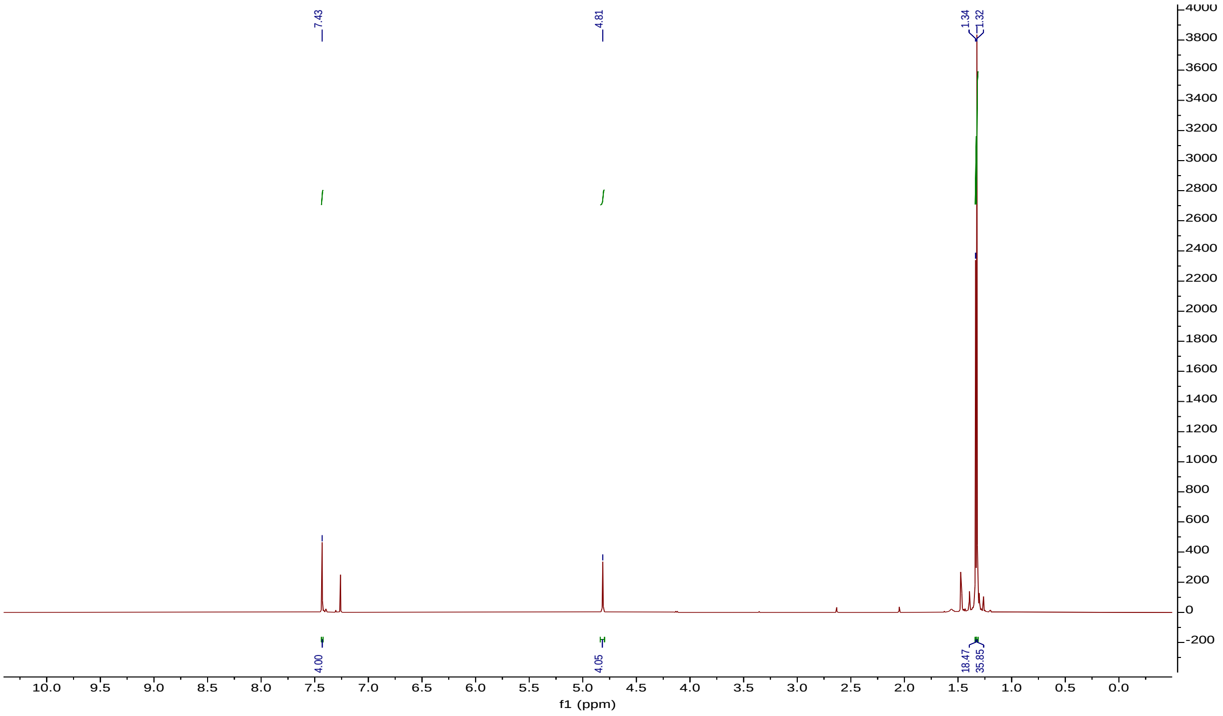

Figure S 26. ^{1}H NMR of **9**, $CDCl_3$

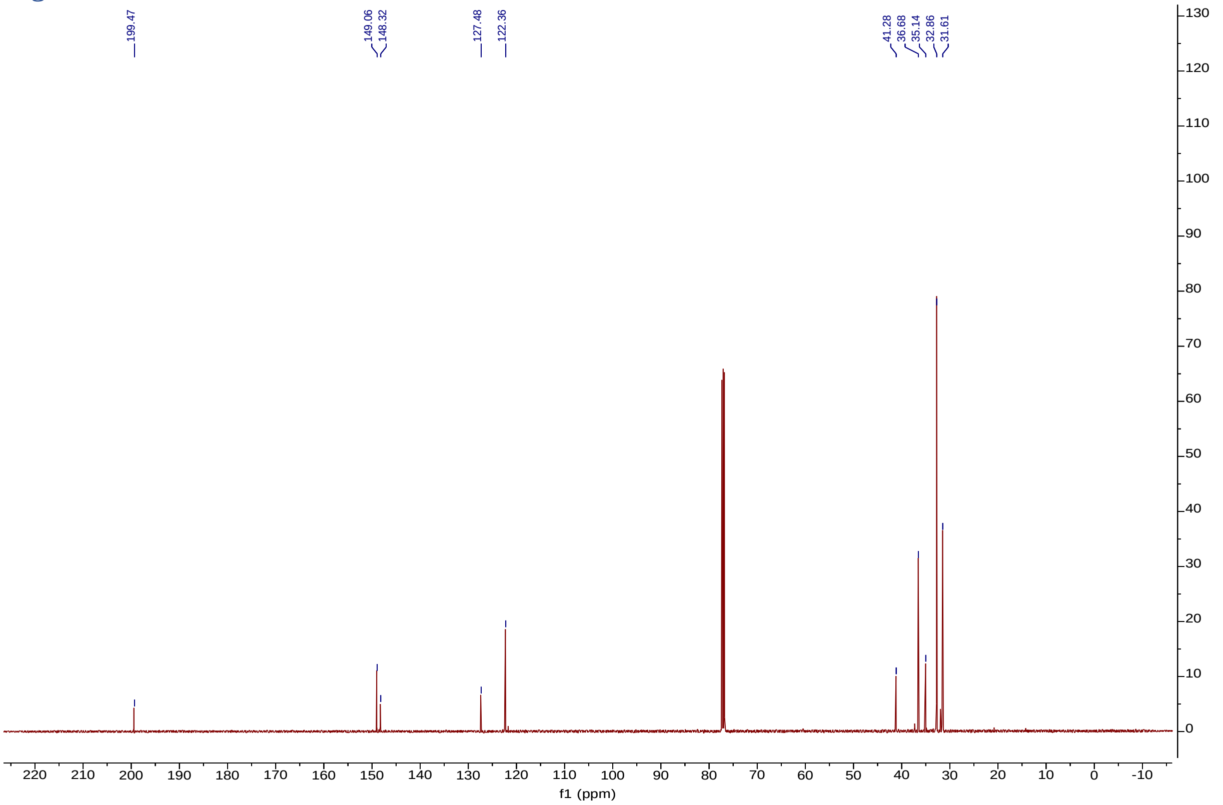

Figure S 27. ^{13}C{^{1}H} NMR of **9**, $CDCl_3$

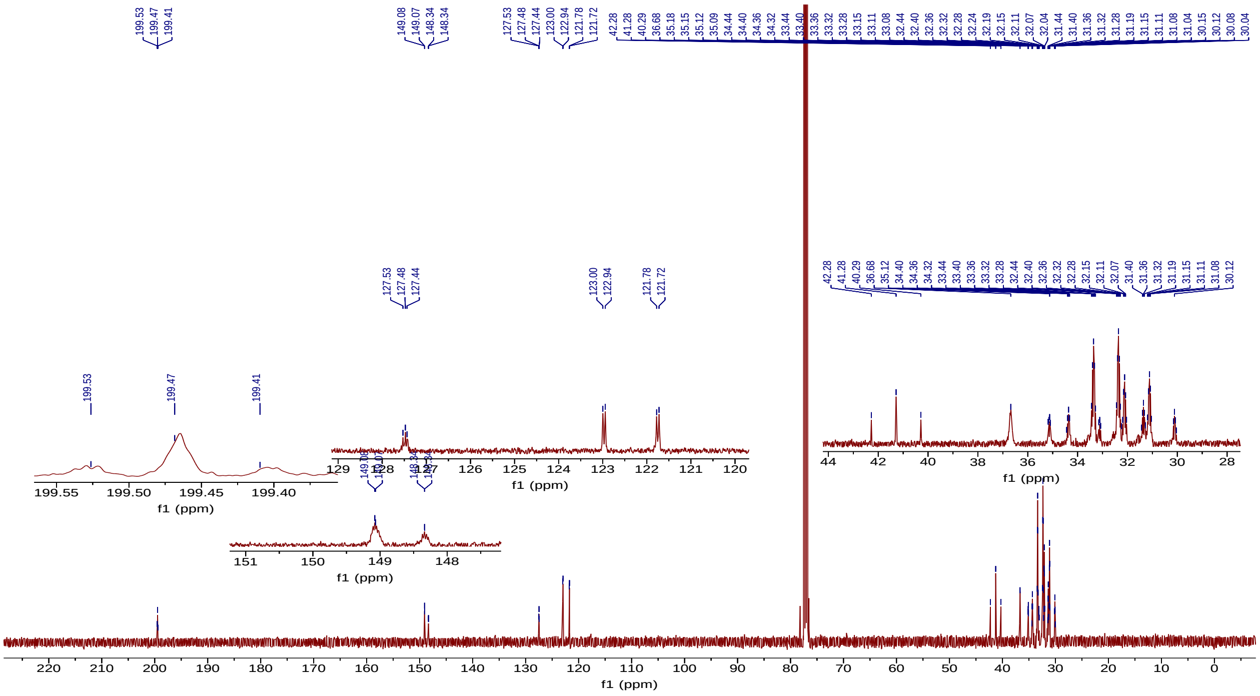

Figure S 28. ^{13}C NMR of **9**, $CDCl_3$

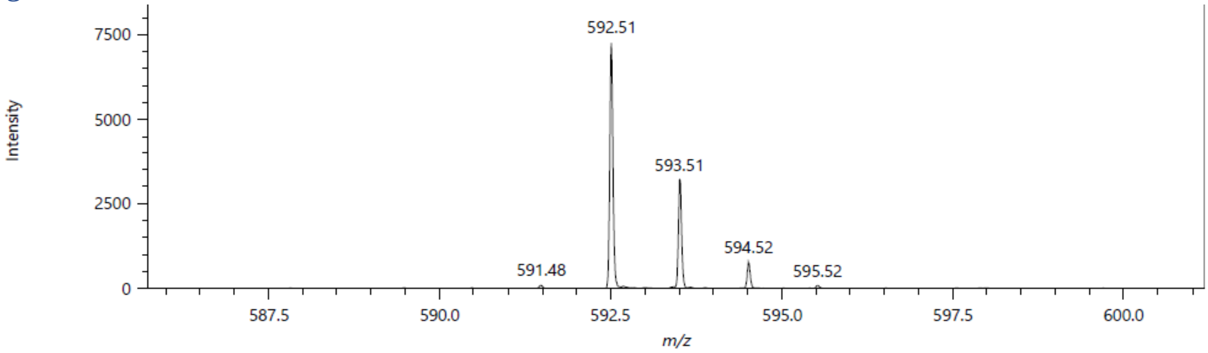

Elemental Composition

Parameters	
Tolerance:	±10.00 mDa
Electron:	Odd/Even
Charge:	+1
DBE:	-1.5 - 100.0

Elements Set 1:

Symbol	C	H	O	N	Br
Min	0	0	0	0	0
Max	100	100	10	10	1

Results

Mass	Intensity	Formula	Calculated Mass	Mass Difference [mDa]	Mass Difference [ppm]	DBE
592.50875	7255.01	C40 H66 N O2	592.50881	-0.06	-0.10	8.5
		C38 H64 N4 O	592.50746	1.28	2.16	9.0
		C27 H64 N10 O4	592.51065	-1.91	-3.22	1.0
		C37 H68 O5	592.50613	2.62	4.42	4.0
		C36 H62 N7	592.50612	2.63	4.43	9.5
		C29 H66 N7 O5	592.51199	-3.25	-5.48	0.5

Figure S 29. HRMS of **9**, DART ionization

In situ formation of **8-^{13}C** and **9-^{13}C** from of **4** with ^{13}CO/degassed water

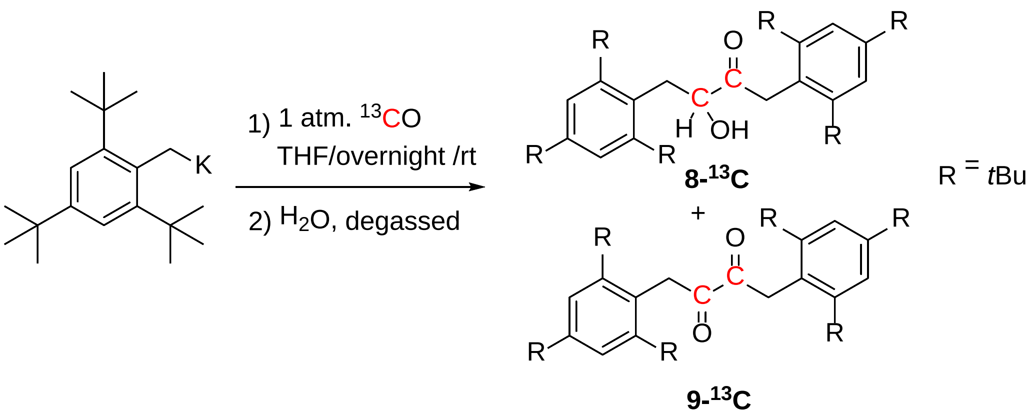

2,4,6-Tris-*tert*-butyl benzyl potassium (10 mg, 0.03 mmol) **4** was dissolved in 0.5 mL THF and transferred to a J-young tube. The solution was freeze-thaw degassed under vacuum, then 1 atm ^{13}CO was added to the head space of J-young tube while the bottom part was kept frozen in a liquid N_2 bath. The reaction turned from orange clear to brownish orange suspension after warming up to room temperature. The reaction was left at room temperature overnight, a drop of degassed water was added, the crude mixture was submitted for HRMS study.

HRMS (DART ionization, *m/z*): **8-^{13}C** calcd for $^{13}C_2C_{38}H_{68}O_2N$, $[M+NH_4]^+$: 596.53117; found: 596.52929.

HRMS (DART ionization, *m/z*): **9-^{13}C** calcd for $^{13}C_2C_{38}H_{63}O_2$, $[M+H]^+$: 577.48897; found: 577.48865.

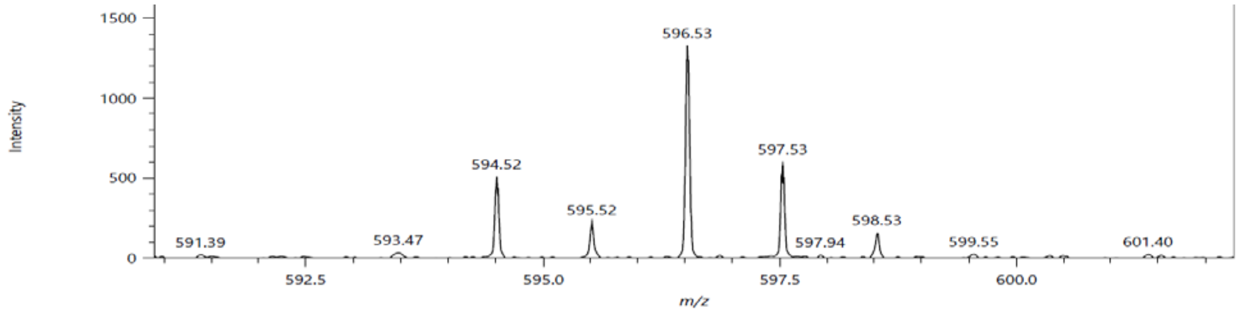

Elemental Composition

Parameters	
Tolerance:	±10.00 mDa
Electron:	Odd/Even
Charge:	+1
DBE:	-1.5 - 100.0

Elements Set 1:

Symbol	C	H	O	N	Cx
Min	0	0	0	0	2
Max	100	100	10	10	2

Results

Mass	Intensity	Formula	Calculated Mass	Mass Difference [mDa]	Mass Difference [ppm]	DBE
596.52929	1329.11	C36 H66 N4 O Cx2	596.52982	-0.53	-0.89	8.0
		C35 H70 O5 Cx2	596.52849	0.81	1.35	3.0
		C34 H64 N7 Cx2	596.52848	0.81	1.36	8.5
		C38 H68 N O2 Cx2	596.53117	-1.87	-3.14	7.5
		C33 H68 N3 O4 Cx2	596.52714	2.15	3.60	3.5
		C31 H66 N6 O3 Cx2	596.52580	3.49	5.86	4.0

Figure S 30. HRMS of **8-^{13}C**, DART ionization

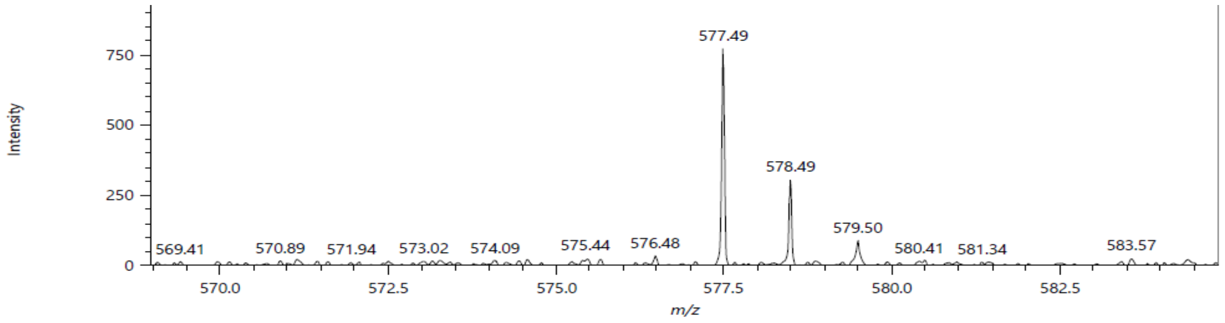

Elemental Composition

Parameters	
Tolerance:	±10.00 mDa
Electron:	Odd/Even
Charge:	+1
DBE:	-1.5 - 100.0

Elements Set 1:

Symbol	C	H	O	N	Cx
Min	0	0	0	0	2
Max	100	100	10	10	2

Results

Mass	Intensity	Formula	Calculated Mass	Mass Difference [mDa]	Mass Difference [ppm]	DBE
577.48865	772.15	C38 H63 O2 Cx2	577.48897	-0.32	-0.56	9.5
		C36 H61 N3 O Cx2	577.48762	1.02	1.77	10.0
		C25 H61 N9 O4 Cx2	577.49081	-2.17	-3.75	2.0
		C34 H59 N6 Cx2	577.48628	2.36	4.09	10.5
		C27 H63 N6 O5 Cx2	577.49216	-3.51	-6.08	1.5
		C33 H63 N2 O4 Cx2	577.48494	3.70	6.41	5.5

Figure S 31. HRMS of **9-^{13}C**, DART ionization

Reaction of **4** with CO at -78°C/1-hexene

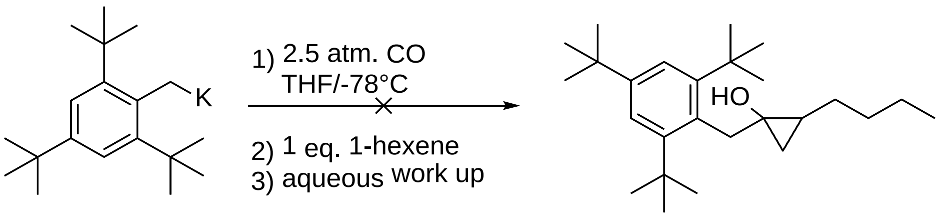

2,4,6-Tris-*tert*-butyl benzyl potassium (85 mg, 2.0 mmol) **4** was dissolved in 20 mL THF and transferred to a Schlenk bomb. The solution was freeze-thaw degassed under vacuum in a liquid N_2 bath. The Schlenk bomb was transferred to a dry ice acetone bath, then CO was added at this temperature. 1-Hexene (0.036 mL, 2 mmol, 1 eq.) was added to the reaction, and the reaction was slowly warmed up to room temperature overnight. Excess water (5 mL) was added via syringe and the reaction was stirred for 30 minutes. The volatiles were removed under vacuum. The residue was extract with EtOAc (2*5 mL) and water (10 mL), the organic layer was washed with brine and dried over $MgSO_4$. The volatiles in the organic layer were removed, ^{1}H NMR spectrum and HRMS study indicated the formation of **9** instead of the cyclopropane derivative.

1,3,5-Tri-aryltoluene (aryl= 3,5-di-*tert*-butylphenyl) **10**

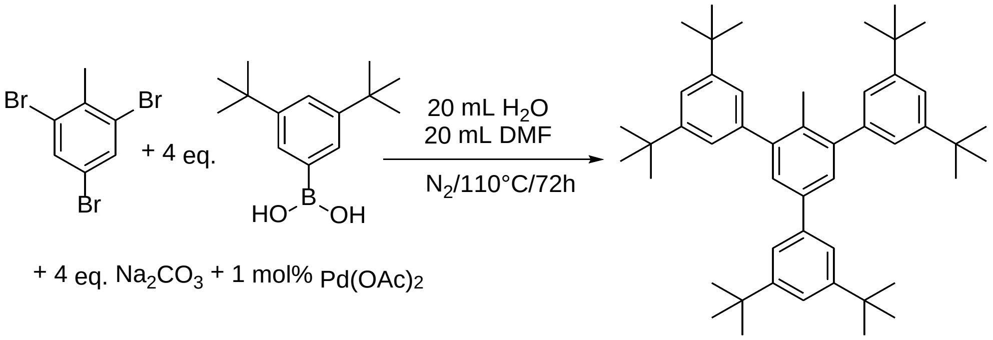

The synthesis was adopted from a known method[5]. To a 100 mL Schlenk flask filled with N_2, 2,4,6-tribromotoluene (1.5 g, 4.55 mmol, 1 eq), sodium carbonate (1.93 g, 18.2 mmol, 4 eq), palladium acetate (20 mg, 0.09 mmol, 0.02 eq) and 3,5-di-*tert*-butylphenylboronic acid (4.27 g, 18.2 mmol, 4 eq) was added via addition funnel. Water (20 mL) and DMF (20 mL) was added to the flask and the slurry was refluxed at 110°C for 72 hours. Volatiles were removed on rotovap under gentle heating, the residue was washed with 2*100 mL hexane. The suspension was filtered on a frit with a thick layer of Celite, the filtrate was collected. Next, the flask was washed with EtOAc and filtered through Celite pad until the filtrate is colorless. The filtrate was combined, the solvent was removed on rotovap, left with pale brownish solids. The residue was redissolved in minimum amount of EtOAc at 60°C, the purple brownish solution was left at room temperature, off-white solids were formed the next day. The solids were collected on frit and washed with 40 mL cold EtOAc. The same process was repeated to obtain the second portion of product, the overall yield of **10** was 2.14 g, 72%.

^{1}H NMR (600 MHz, THF-d_8) δ 7.45 (t, *J* = 1.9 Hz, 2H), 7.43 (bs, 3H), 7.39 (s, 2H), 7.26 (d, *J* = 1.9 Hz, 4H), 2.09 (s, 3H), 1.36 (s, 36H), 1.35 (s, 18H).

^{13}C{^{1}H} NMR (151 MHz, THF-d_8) δ 155.95, 151.55, 150.94, 144.97, 142.77, 128.27, 124.41, 122.28, 121.62, 121.09, 35.37, 31.70, 31.67. Not all the signals were observed.

^{1}H NMR (500 MHz, $CDCl_3$) δ 7.54 (s, 2H), 7.48 (d, *J* = 1.8 Hz, 2H), 7.44 (t, *J* = 1.8 Hz, 2H), 7.43 (t, *J* = 1.8 Hz, 1H), 7.31 (d, *J* = 1.8 Hz, 4H), 2.15 (s, 3H), 1.38 (s, 36H), 1.37 (s, 18H).

^{13}C{^{1}H} NMR (101 MHz, $CDCl_3$) δ 151.22, 150.49, 144.27, 141.82, 140.65, 139.76, 132.25, 128.08, 124.10, 122.00, 121.42, 120.72, 35.15, 35.13, 31.73, 31.72, 18.54.

^{1}H NMR (400 MHz, C_6D_6) δ 7.99 (s, 2H), 7.73 (s, 2H), 7.57 (s, 2H), 7.55-7.52 (s, 5H), 2.49 (s, 3H), 1.31 (s, 36H), 1.25 (s,18).

HRMS (EI ionization, *m/z*): calcd for $C_{49}H_{68}$, $[M]^+$: 656.5321; found: 656.5319.

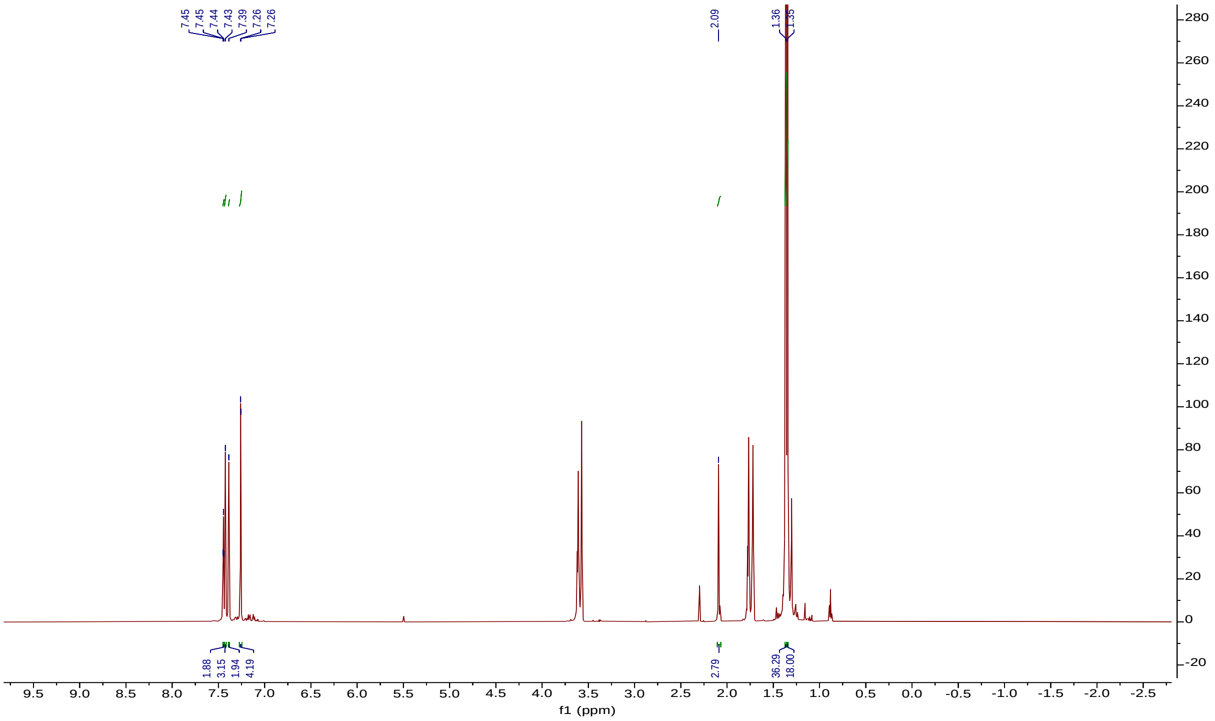

Figure S 32. ^{1}H NMR of **10**, THF-d8

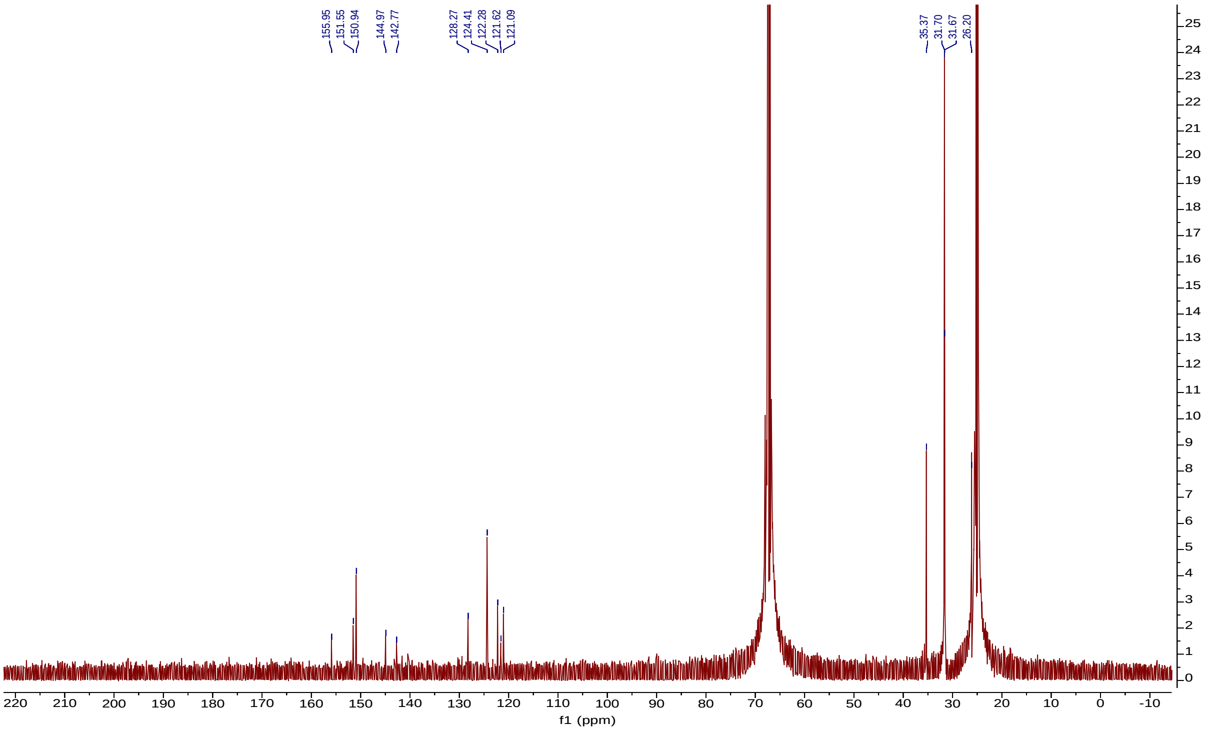

Figure S 33. ^{13}C{^{1}H} NMR of **10**, THF-d8

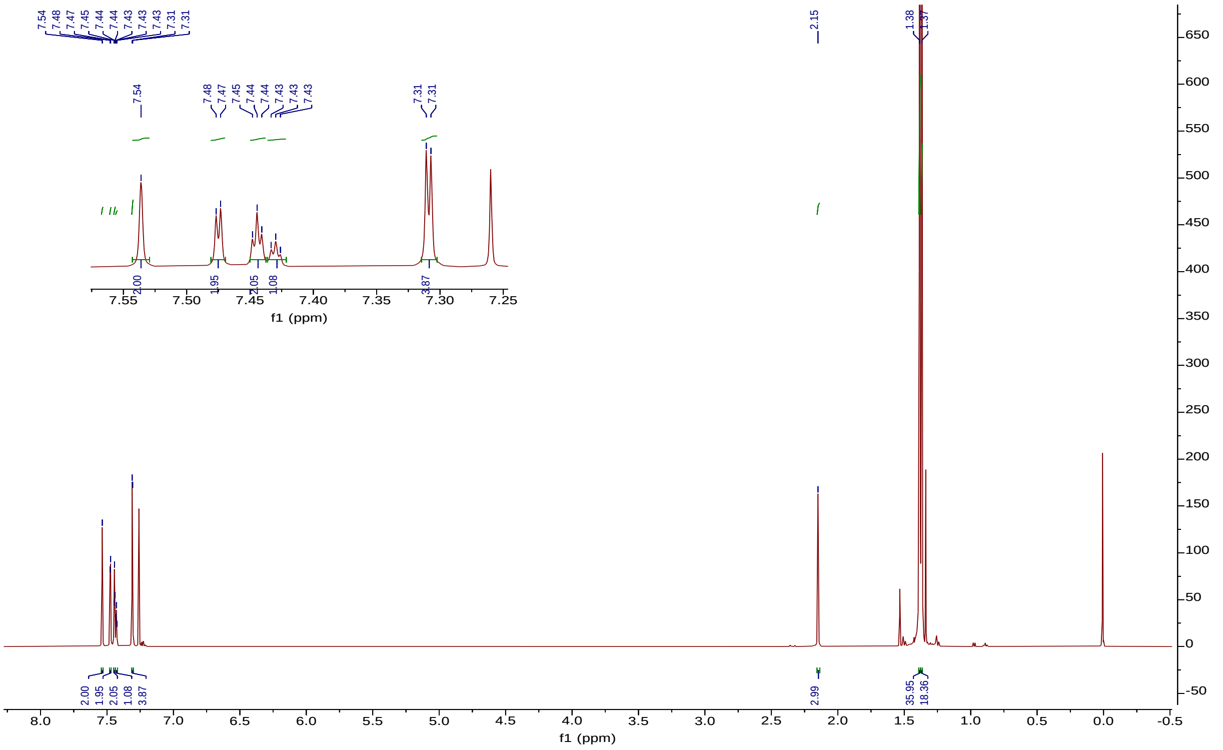

Figure S 34. ^{1}H NMR of **10**, $CDCl_3$

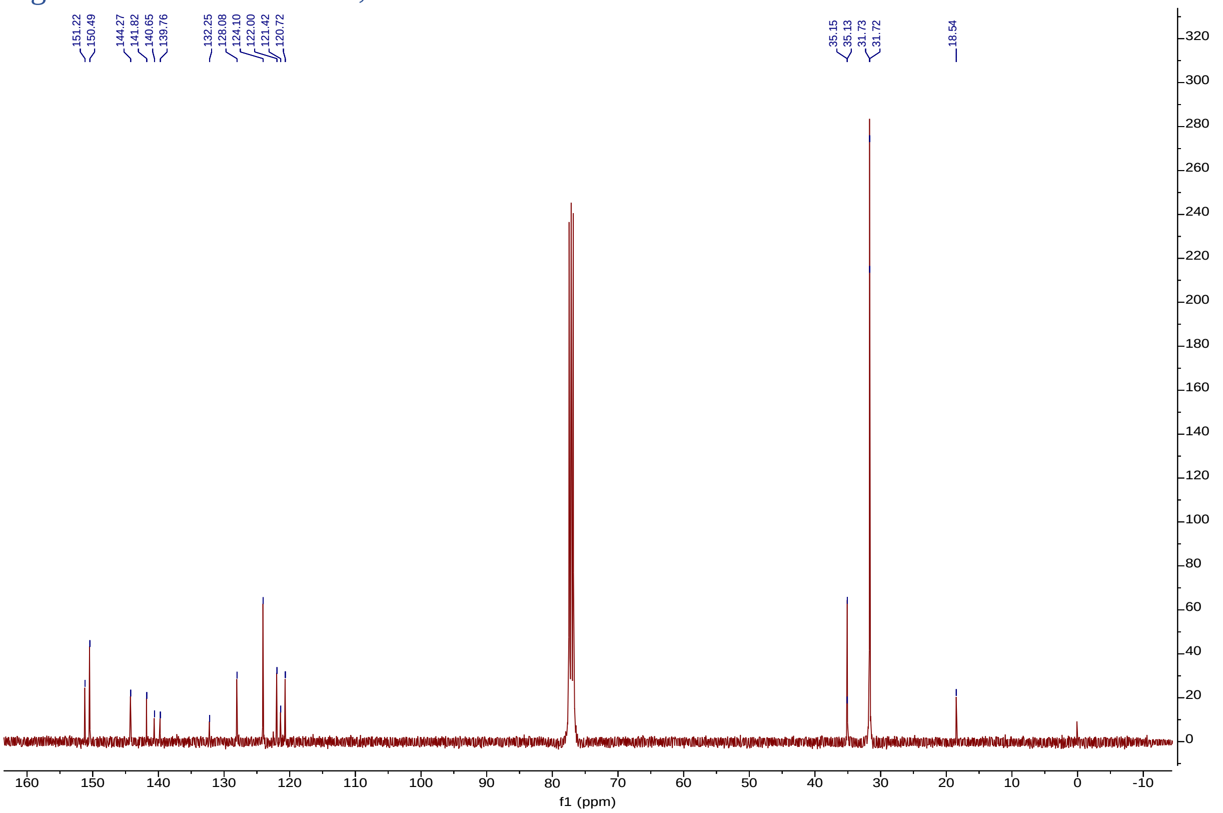

Figure S 35. $^{13}C\{^{1}H\}$ NMR of **10**, $CDCl_3$

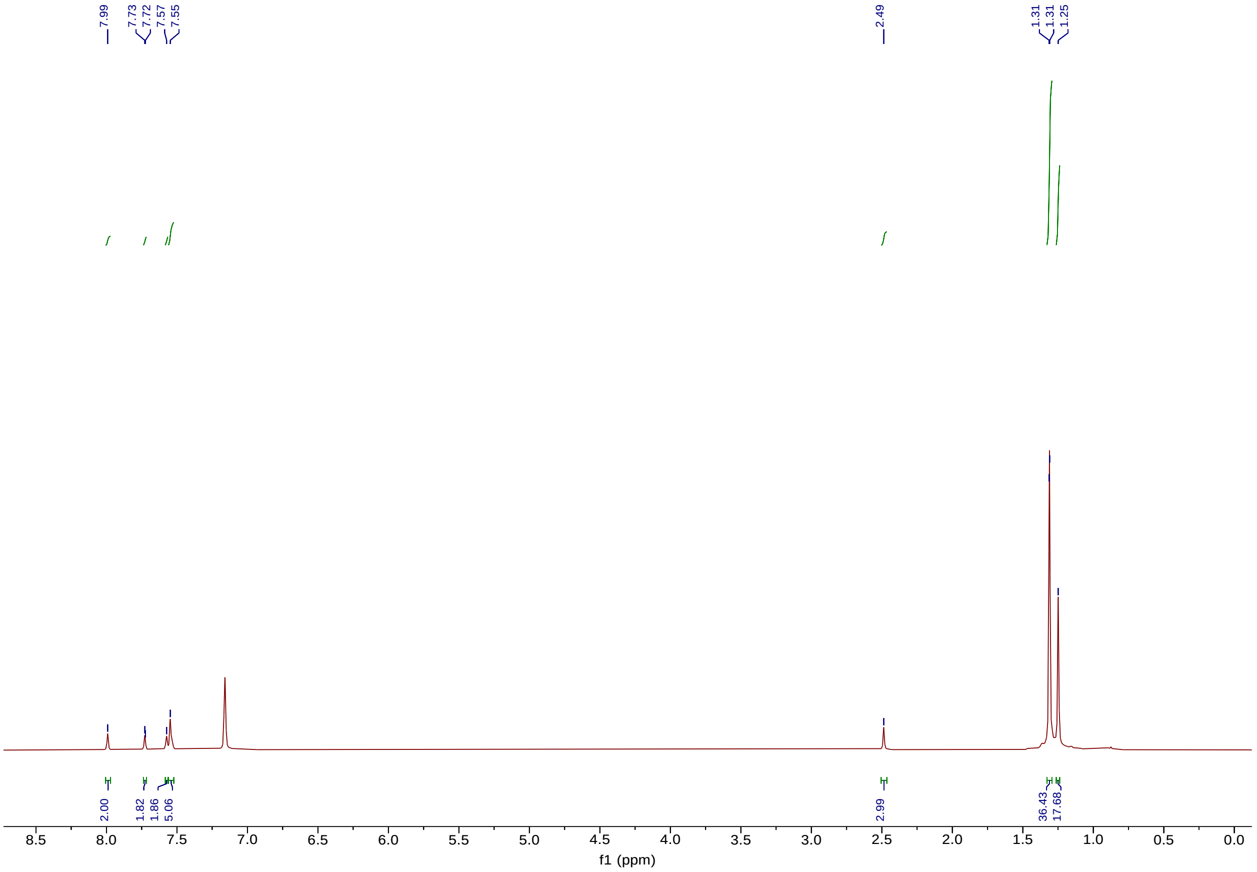

Figure S 36. ^{1}H NMR of **10**, C_6D_6

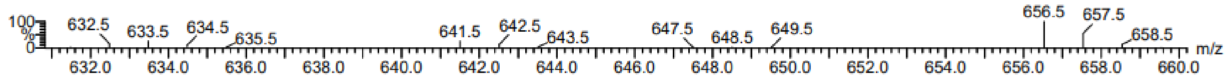

Minimum:				-1.5		
Maximum:		5.0	10.0	50.0		
Mass	Calc. Mass	mDa	PPM	DBE	i-FIT	Formula
656.5319	656.5321	-0.2	-0.3	16.0	14.1	C49 H68
	656.5313	0.6	0.9	4.0	123.7	C33 H68 N8 O5
	656.5326	-0.7	-1.1	3.5	95.2	C35 H70 N5 O6
	656.5340	-2.1	-3.2	3.0	71.8	C37 H72 N2 O7
	656.5286	3.3	5.0	-0.5	204.8	C30 H70 N7 O8
	656.5353	-3.4	-5.2	8.0	45.3	C38 H68 N6 O3
	656.5281	3.8	5.8	12.0	7.3	C44 H68 N2 O2
	656.5366	-4.7	-7.2	7.5	31.3	C40 H70 N3 O4
	656.5267	5.2	7.9	12.5	12.6	C42 H66 N5 O
	656.5380	-6.1	-9.3	12.5	16.5	C41 H66 N7
	656.5380	-6.1	-9.3	7.0	22.4	C42 H72 O5
	656.5254	6.5	9.9	13.0	22.7	C40 H64 N8
	656.5254	6.5	9.9	7.5	28.5	C41 H70 N O5

Figure S 37. HRMS of **10**, EI ionization

2,4,6-(3,5-tBu$_2$C$_6$H$_3$)$_3$C$_6$H$_2$CH$_2$K **11**

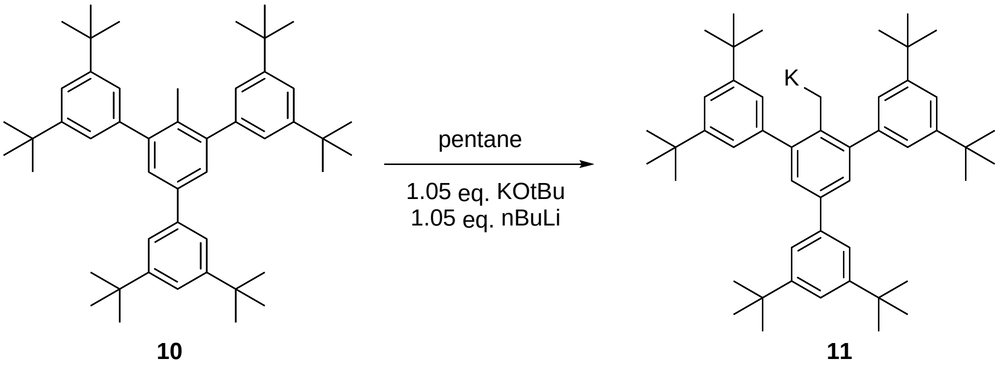

10 (450 mg, 0.68 mmol, 1 eq) and potassium tert-butoxide (81 mg, 0.71 mmol, 1.05 eq) was suspended in 10 mL pentane at room temperature. *n*BuLi (0.29 mL, 2.5 M, 1.05 eq) was added dropwise via syringe, the reaction was stirred overnight. The black solids **11** were collected on a frit after washed with pentane (380 mg, 80% yield).

^{1}H NMR (400 MHz, C$_6$D$_6$) δ 7.90 (d, J = 1.9 Hz, 4H), 7.64 (d, J = 1.8 Hz, 2H), 7.43 (t, J = 1.8 Hz, 2H), 7.37 (s, 1H), 7.36 (s, 1H), 7.21 (t, J = 1.7 Hz, 1H), 3.78 (s, 2H), 1.43 (s, 36H), 1.39 (s, 18H).

^{1}H NMR (400 MHz, Et$_2$O, set to 3.38 and 1.12 ppm) δ 7.61 (4H), 7.15 (2H), 7.11 (2H), 6.79 (3H), 3.49 (2H), 1.34 (36H), 1.28 (18H).

^{1}H NMR (400 MHz, THF) δ 7.53 (4H), 7.07 (4H), 6.71 (1H), 6.66 (2H), 3.29 (2H), 1.29 (36H), 1.23 (18H).

^{13}C{^{1}H} NMR (101 MHz, C$_6$D$_6$) δ 150.80, 150.65, 146.23, 123.41, 119.00, 116.44, 35.16, 35.08, 31.92. Not all the signals were observed.

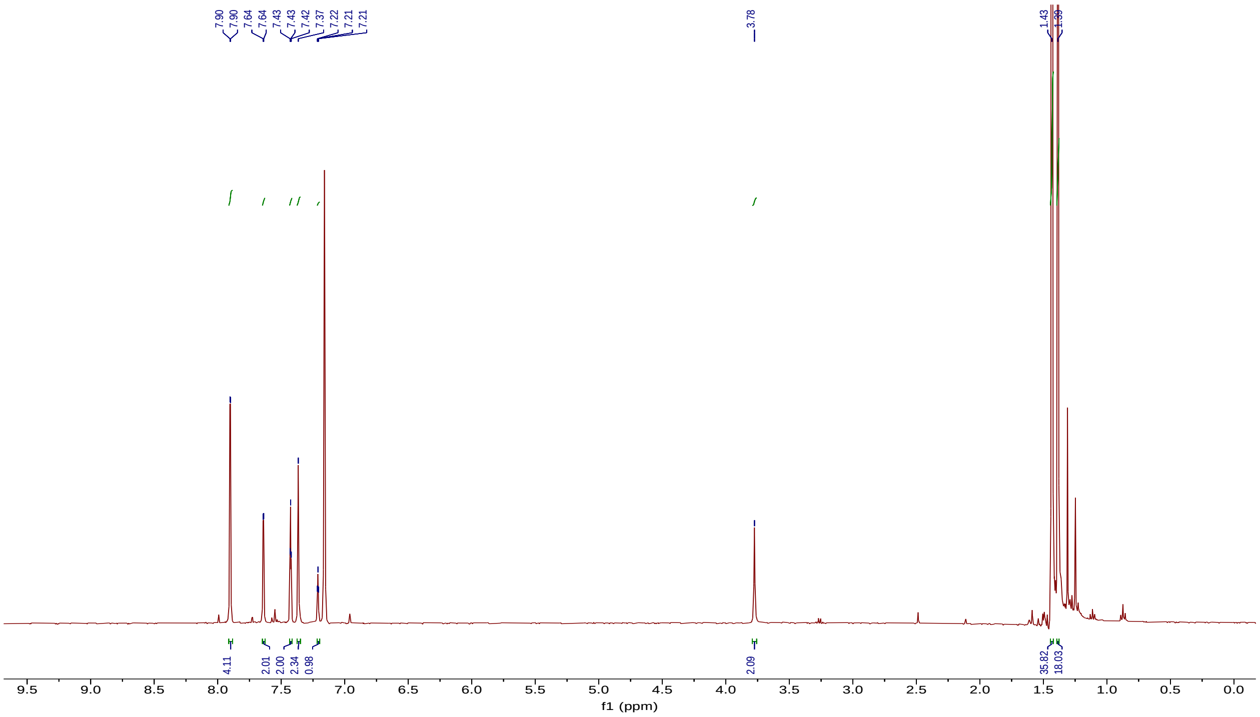

Figure S 38. ^{1}H NMR spectrum of **11**, C_6D_6

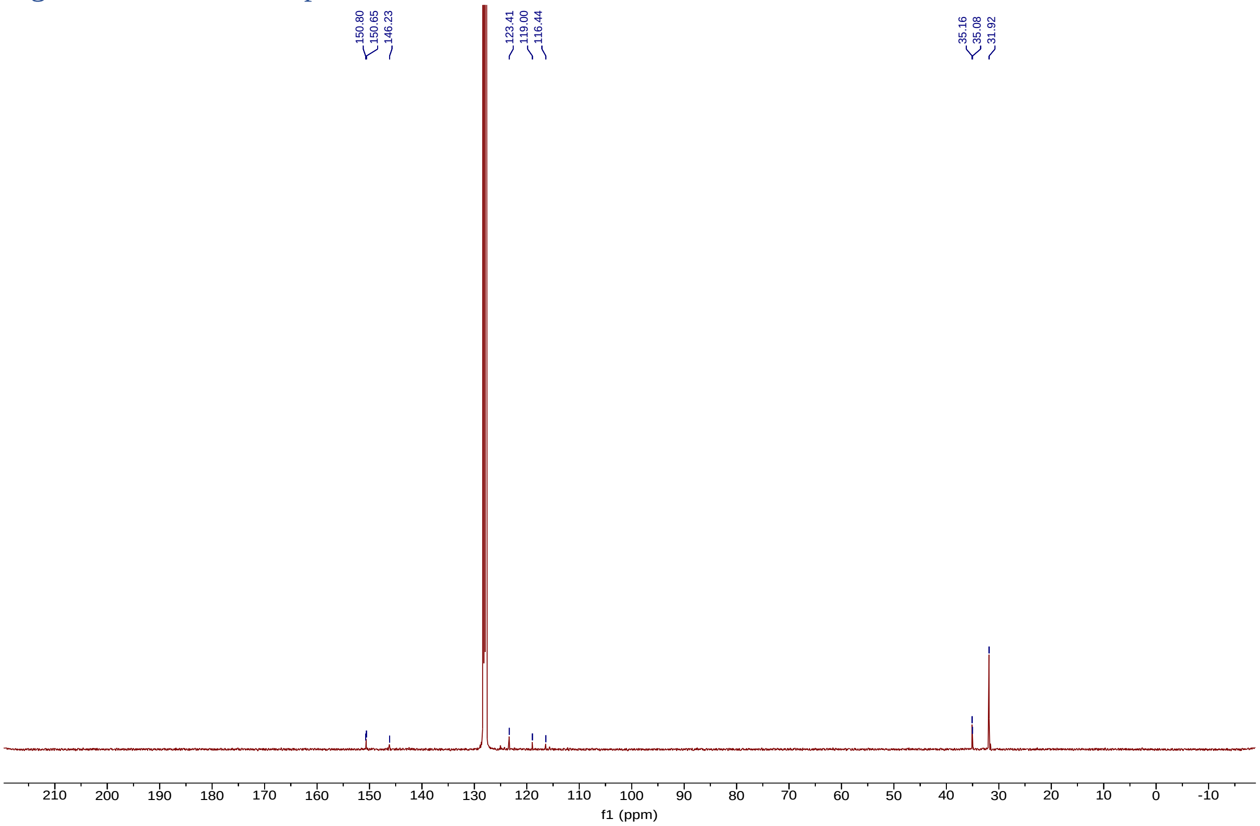

Figure S 39. ^{13}C{^{1}H} NMR of **11**, C_6D_6

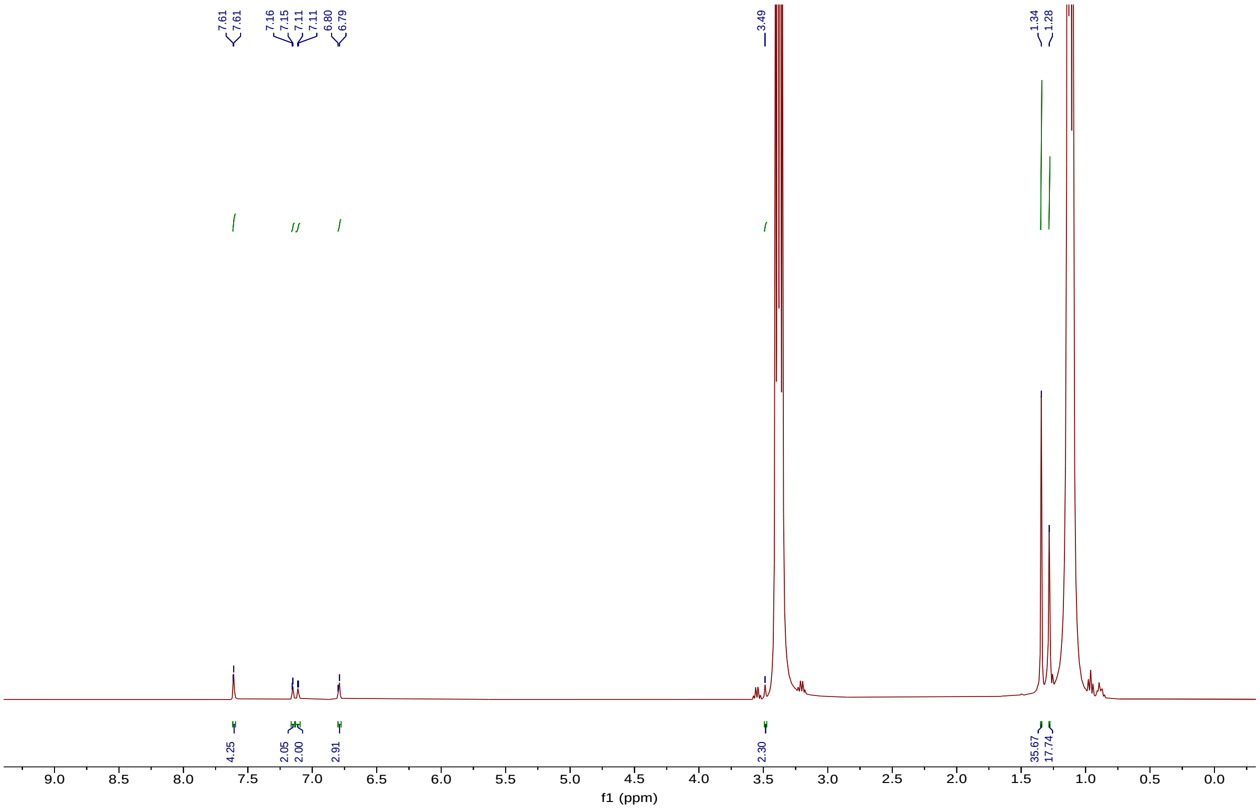

Figure S 40. ^{1}H NMR spectrum of **11**, Et_2O

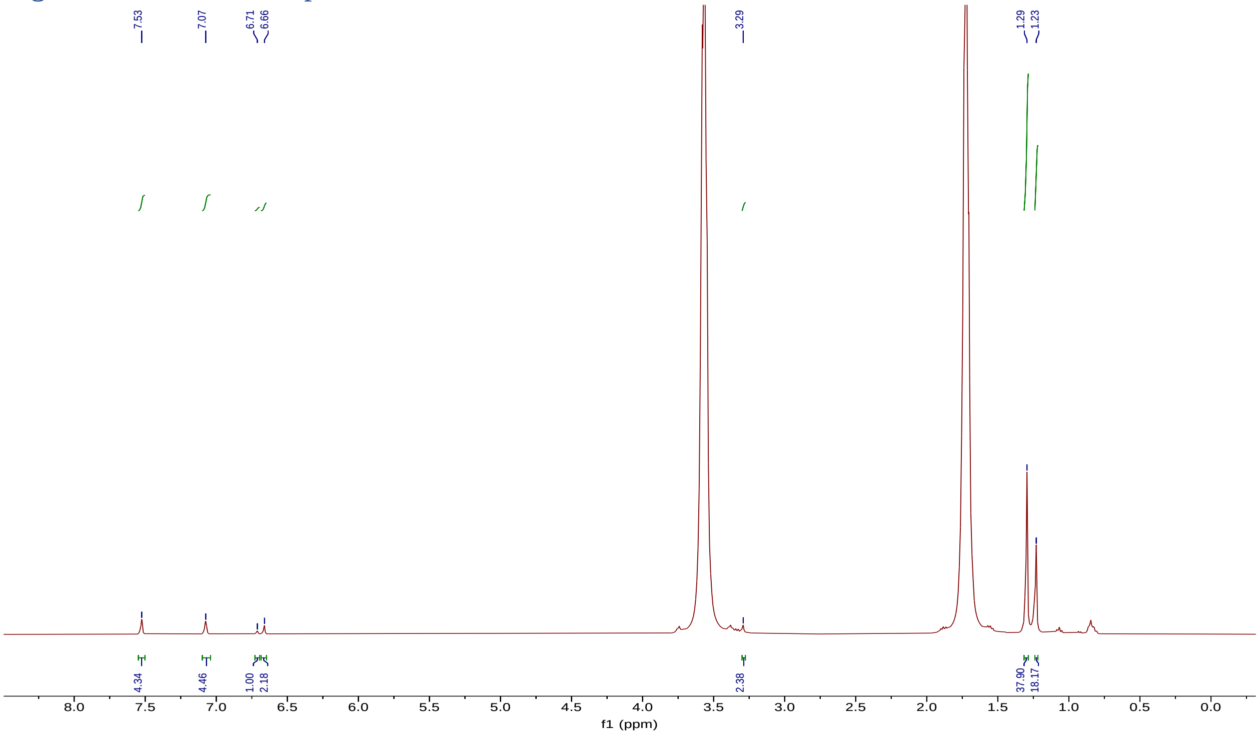

Figure S 41. ^{1}H NMR spectrum of **11**, THF

Reaction of **11** with ^{13}CO in Et_2O/ aqueous work up

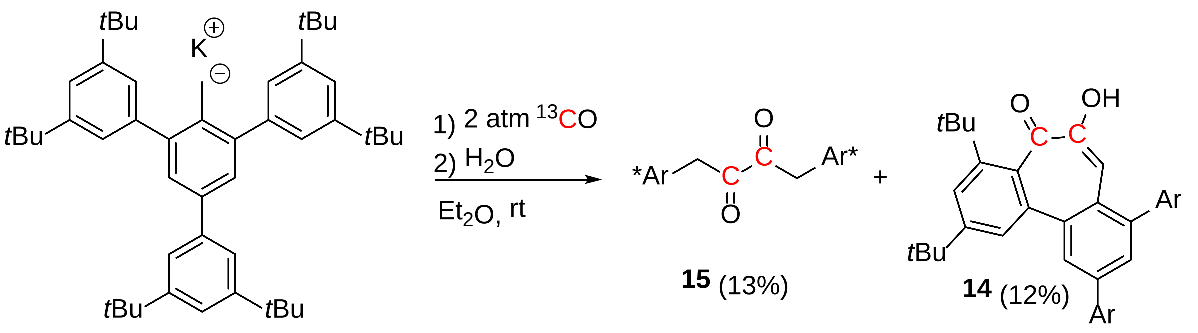

11 (15 mg, 0.02 mmol) was dissolved in Et_2O. The solution was transferred to a J-young tube, 2 atm. ^{13}CO was added to the head space of J-young tube while the bottom part was kept frozen in a liquid N_2 bath. The reaction was left at room temperature for 24 hours, a color change from dark green to red brown was observed. ^{1}H NMR study suggests a complete consumption of **11.** EPR study of the mixture revealed the formation of radical species **12**. To this solution, excess water was added, an immediate color change to pale yellow was observed. The mixture was dried under vacuum, afforded yellow solids. Attempts to separate the products by recrystallization or column chromatography on silica gel were unsuccessful due to their similar solubility. The yield of **14** and **15** was determined using 4 mg ^{13}C enriched 1,3-bis(2,6-diisopropylphenyl)urea (MW = 381.28 g/mol, ^{13}C resonance =156 ppm) as internal standard in $CDCl_3$. The yield of each species was determined by the following equation:

$$Yield = \frac{4mg\ [13C]DippUrea}{381.28\ mg/mmol} \times \frac{integration\ of\ [13C]\ product}{integration\ of\ [13C]DippUrea} \div 0.02\ mmol \times 100\%$$

Yield of each species: **15** (12.9 %), **14** (12.2 %).

$^{13}C\{^{1}H\}$ NMR of **15** and **14** (151 MHz, $CDCl_3$) δ 202.03 (s, **15**), 196.95 (d, J = 61.0 Hz, **14**), 147.41 (d, J = 61.0 Hz, **14**).

HRMS (DART ionization, *m/z*) **14**: calcd for $C_{49}{}^{13}C_2H_{67}O_2$, $[M+H]^+$: 713.52027; found: 713.51956.

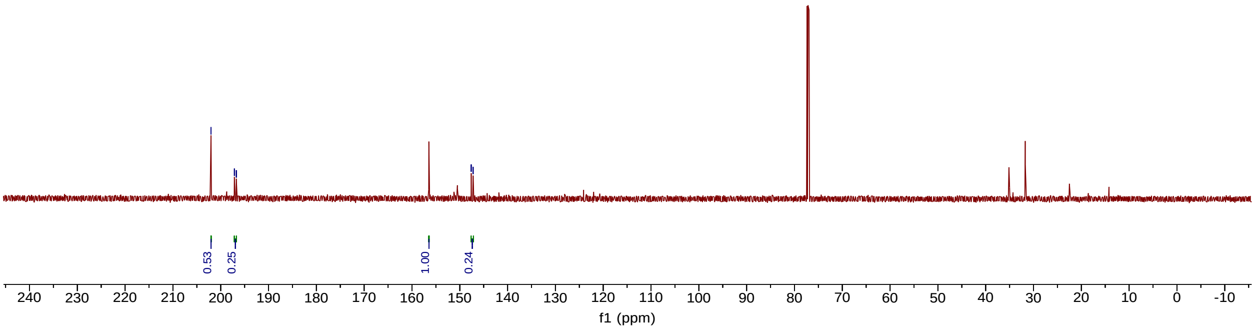

Figure S 42. $^{13}C\{^{1}H\}$ NMR study of a mixture containing **10**, **14** and **15**, $CDCl_3$

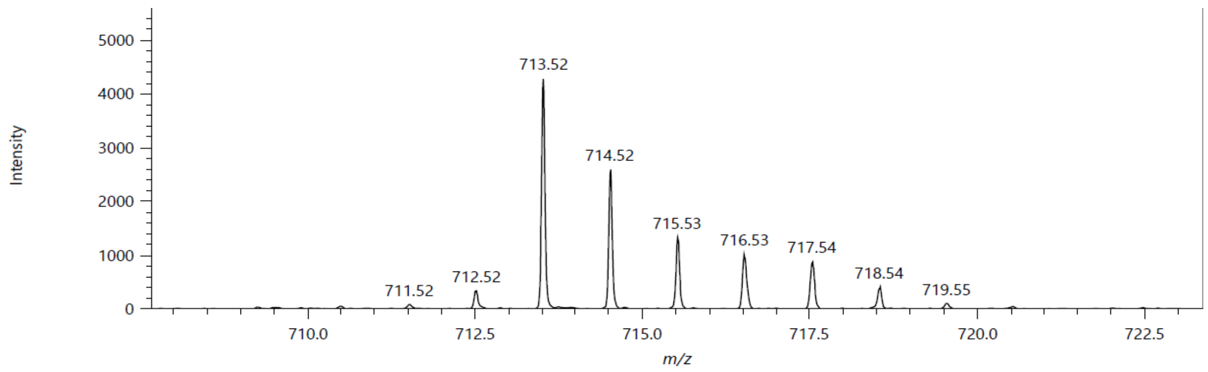

Elemental Composition

Parameters

Tolerance:	±10.00 mDa
Electron:	Odd/Even
Charge:	+1
DBE:	-1.5 - 100.0

Elements Set 1:

Symbol	C	H	O	N	Cx
Min	0	0	0	0	2
Max	100	100	10	10	2

Results

Mass	Intensity	Formula	Calculated Mass	Mass Difference [mDa]	Mass Difference [ppm]	DBE
713.51956	4276.23	C33 H67 N8 O7 Cx2	713.51943	0.12	0.17	6.5
		C47 H65 N3 O Cx2	713.51892	0.63	0.89	19.0
		C49 H67 O2 Cx2	713.52027	-0.71	-1.00	18.5
		C35 H69 N5 O8 Cx2	713.52078	-1.22	-1.71	6.0
		C45 H63 N6 Cx2	713.51758	1.97	2.77	19.5
		C36 H65 N9 O4 Cx2	713.52211	-2.56	-3.58	11.0

Figure S 43. HRMS of **14**, DART ionization

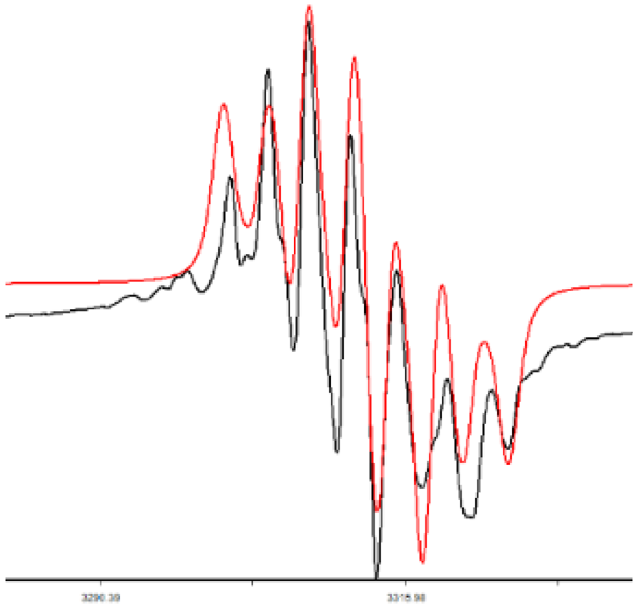

Figure S 44. EPR of radical **12**, experimental: black; simulation red.

Reaction of **11** with ^{13}CO in THF/ aqueous work up

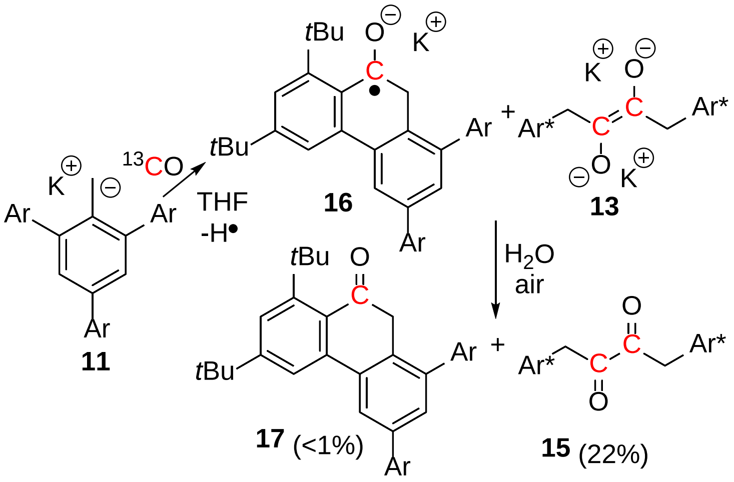

11 (5 mg, 0.007 mmol) was dissolved in THF, the solution was transferred to a J-young tube and charged with 1 atm. ^{13}CO to head space of the J-young tube while the bottom part was kept frozen in a liquid N_2 bath. A color change from dark green to red brown was observed. Upon standing overnight, the ^1H NMR data revealed the consumption of **11**. Examination of the red-brown solution by EPR revealed a strong multiplet signal centered at g = 2.004. Using the DFT predicted hyperfine couplings as initial parameters, the interactively fit with couplings to a ^{13}C carbon (5.7 G), the methylene (12.2 G), 4 aromatic protons (0.1, 1.3, 1.3, 2.7 G). These observations were consistent with the formulation of the product as the radical anion salt, K^+ $[3,5\text{-}(tBu)_2C_6H_2)C(O)CH_2C_6H_2(C_6H_3 3,5\text{-}(tBu)_2)_2]^{-\bullet}$ **16**. Our DFT calculations suggest that monomer **11** takes one CO molecule to form a carbene-like site that insert into an adjacent aromatic C–H bond to give the radical **16** with loss of a hydrogen radical. Quenching the reaction mixture with water in air and subsequent dissolution in $CDCl_3$ affording 2 new diamagnetic products $3,5\text{-}(tBu)_2C_6H_2)C(O)CH_2C_6H_2(C_6H_3 3,5\text{-}(tBu)_2)_2$ **17** and $((2,4,6\text{-}(3,5\text{-}tBu_2C_6H_3)_3C_6H_2CH_2C(O))_2$ **15**. Compound **17** was formed in <1% yield and its formulation was confirmed by HRMS. The major species **15** was formed in 21.7% yield, the rest of the **11** was converted to **10**.

^1H NMR of **15** (400 MHz, $CDCl_3$) δ 7.42 (d, *J* = 1.8 Hz, 2H), 7.40 (s, 4H), 7.35 (t, *J* = 1.8 Hz, 4H), 7.30 (d, *J* = 1.8 Hz, 8H), 7.06 (d, *J* = 1.8 Hz, 4H), 3.44 (t, *J* = 3.3 Hz, 4H), 1.34 (s, 36H), 1.22 (s, 72H).

^{13}CNMR of **15** (101 MHz, $CDCl_3$) δ 202.00 (t, *J* = 3.3 Hz), only the ^{13}C incorporated signal (*C*=O) was observed.

HRMS of **15** (DART ionization, *m/z*): calcd for $C_{98}{}^{13}C_2H_{134}O_2$, $[M+H]^+$: 1370.05237; found: 1370.05206.

HRMS of **17** (DART ionization, *m/z*): calcd for $C_{49}{}^{13}C_1H_{67}O$, $[M+H]^+$: 684.52200; found: 684.52135.

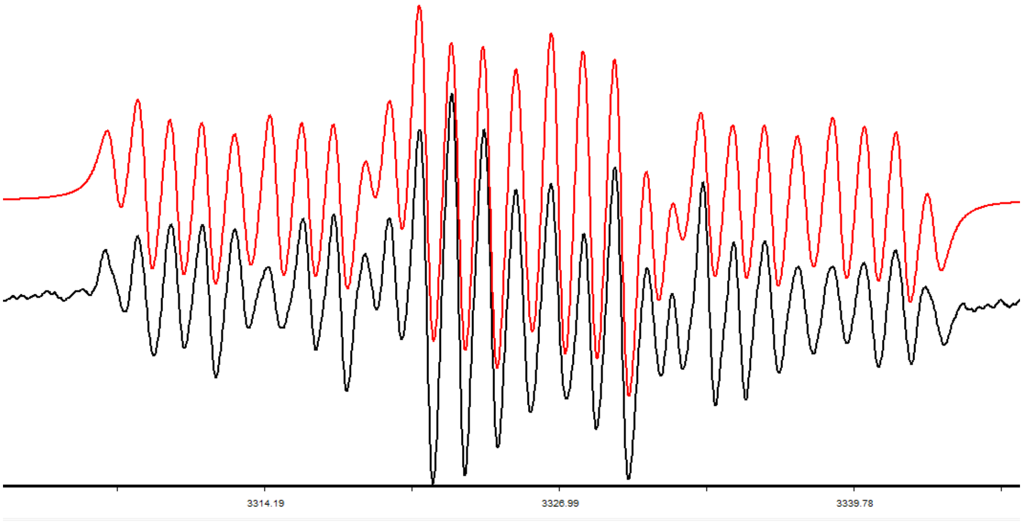

Figure S 45. EPR spectrum of the radical anion **16**; experimental: black; simulation red.

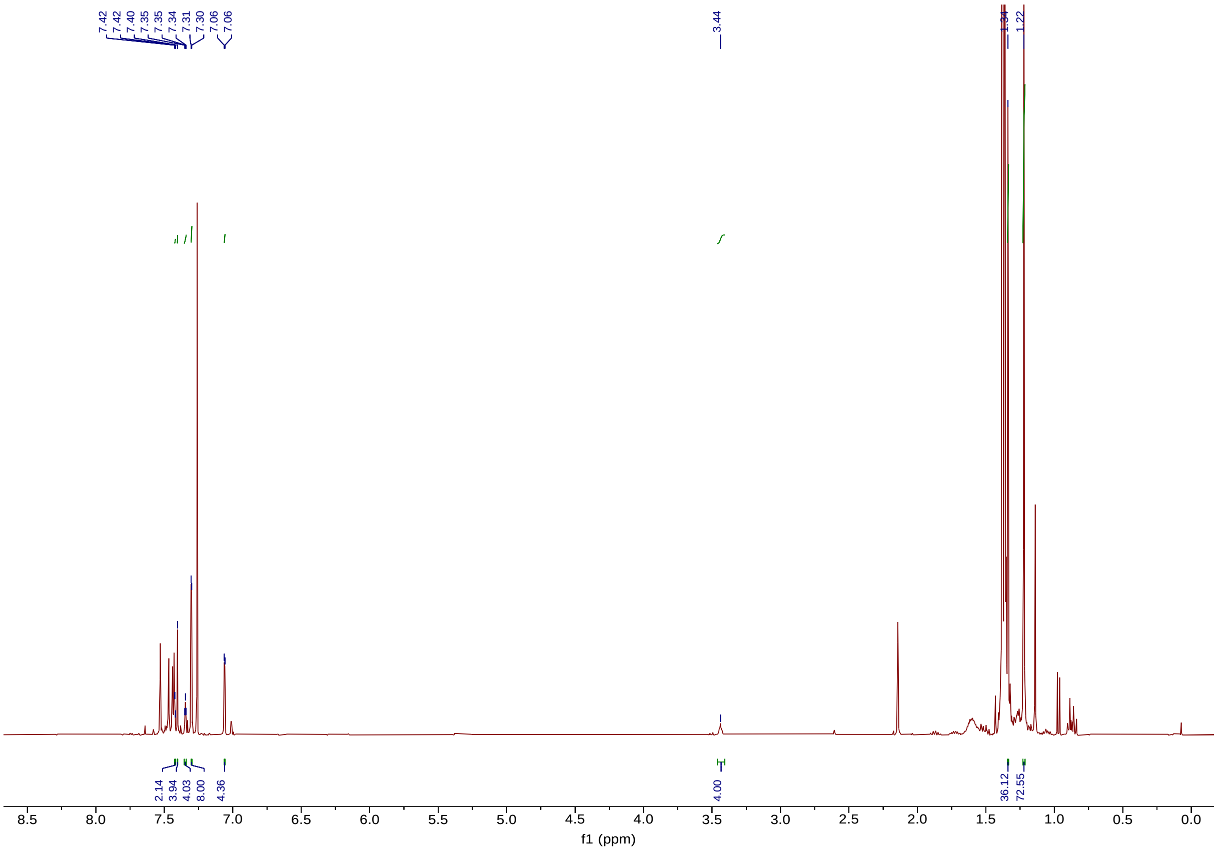

Figure S 46. ^{1}H NMR study of a mixture containing **10**, **17** and **15**, $CDCl_3$

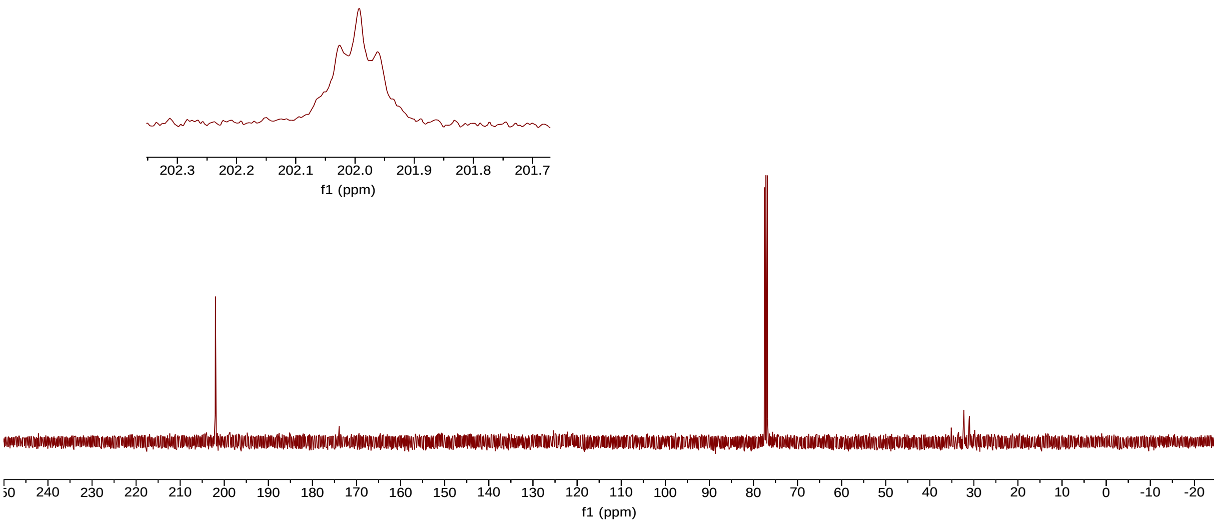

Figure S 47. ^{13}C{^{1}H} NMR study of a mixture containing **10**, **17** and **15**, $CDCl_3$

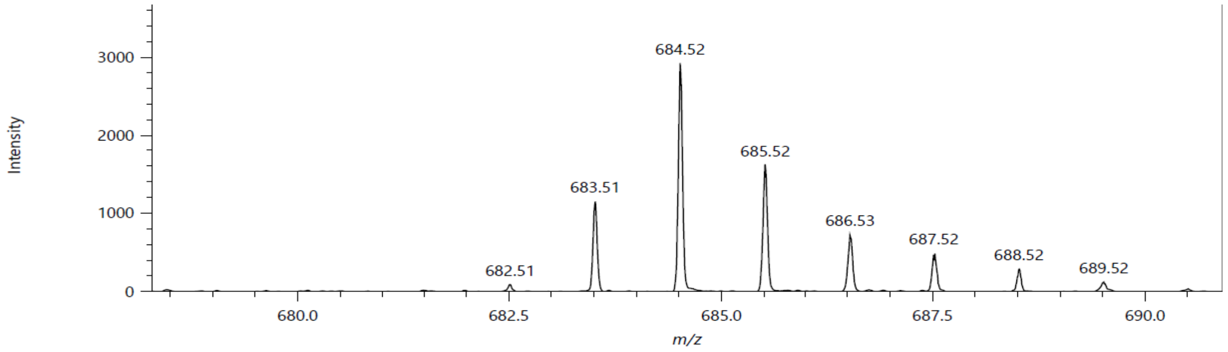

Elemental Composition

Parameters		Elements Set 1:					
Tolerance:	±10.00 mDa	Symbol	C	H	O	N	Cx
Electron:	Odd/Even	Min	0	0	0	0	1
Charge:	+1	Max	100	100	10	10	1
DBE:	-1.5 - 100.0						

Results

Mass	Intensity	Formula	Calculated Mass	Mass Difference [mDa]	Mass Difference [ppm]	DBE
684.52135	2921.36	C33 H67 N8 O6 Cx	684.52116	0.19	0.28	5.5
		C49 H67 O Cx	684.52200	-0.64	-0.94	17.5
		C47 H65 N3 Cx	684.52066	0.70	1.02	18.0
		C35 H69 N5 O7 Cx	684.52251	-1.15	-1.68	5.0
		C32 H71 N4 O10 Cx	684.51983	1.53	2.23	0.5
		C36 H65 N9 O3 Cx	684.52384	-2.49	-3.64	10.0

Figure S 48. HRMS of **17**, DART ionization

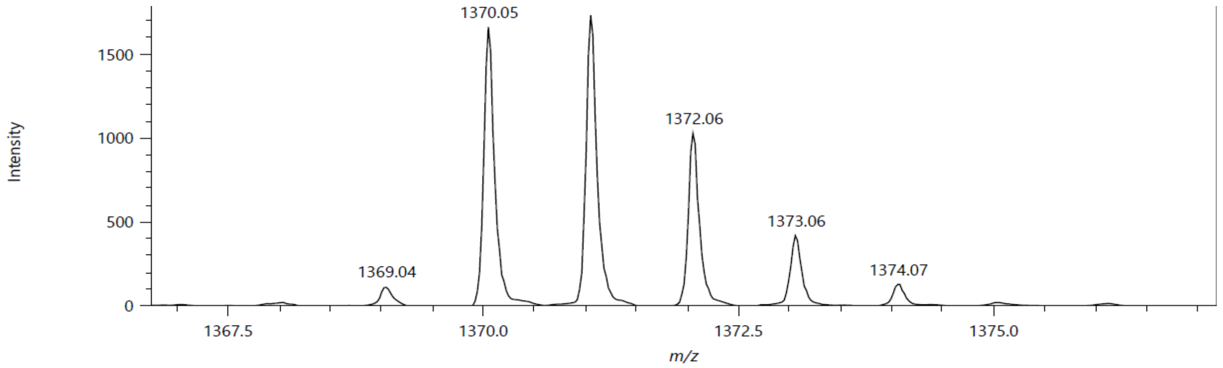

Elemental Composition

Parameters		Elements Set 1:					
Tolerance:	±10.00 mDa	Symbol	C	H	O	N	Cx
Electron:	Odd/Even	Min	80	100	0	0	2
Charge:	+1	Max	100	140	10	10	2
DBE:	-1.5 - 100.0						

Results

Mass	Intensity	Formula	Calculated Mass	Mass Difference [mDa]	Mass Difference [ppm]	DBE
1370.05206	1661.97	C98 H135 O2 Cx2	1370.05237	-0.31	-0.22	33.5
		C82 H135 N8 O7 Cx2	1370.05153	0.53	0.39	21.5
		C84 H137 N5 O8 Cx2	1370.05288	-0.81	-0.59	21.0
		C96 H133 N3 O Cx2	1370.05103	1.04	0.76	34.0
		C85 H133 N9 O4 Cx2	1370.05421	-2.15	-1.57	26.0
		C86 H139 N2 O9 Cx2	1370.05422	-2.16	-1.57	20.5

Figure S 49. HRMS of **15**, DART ionization

DFT calculations

Computational Details:

The quantum chemical DFT calculations have been performed with the TURBOMOLE 7.4 suite of programs[6] The structures are fully optimized at the TPSS-D3/def2-TZVP + COSMO(THF) level of theory, which combines the TPSS meta-GGA density functional[7] with the BJ-damped DFT-D3 dispersion correction[8, 9] and the def2-TZVP basis set,[10, 11] using the Conductor-like Screening Model (COSMO) continuum solvation model[12] for THF solvent (dielectric constant ε = 7.58 and solvent diameter R_{solv} = 3.18 Å). The density-fitting RI-J approach[10, 13, 14] is used to accelerate the geometry optimization and numerical harmonic frequency calculations[15] in solution. The optimized structures are characterized by frequency analysis to identify the nature of located stationary points (no imaginary frequency for true minima and only one imaginary frequency for transition state) and to provide thermal corrections (at 298.15 K and 1 atm) according to the modified ideal gas–rigid rotor–harmonic oscillator model.[16] This choice of dispersion-corrected meta-GGA functional makes the efficient exploration of all potential reaction paths possible.

The final solvation free energies in THF are computed with the COSMO-RS solvation model[17] (parameter file: BP_TZVP_C30_1601.ctd) using the COSMOtherm program package[18] on the above TPSS-D3 optimized structures, and corrected by +1.89 kcal·mol^{-1} to account for higher reference solute concentration of 1 mol·L^{-1} usually used in solution. For THF molecule, an additional free energy correction of 1.49 kcal/mol is added to account for its high concentration as solvent. To check the effects of the chosen DFT functional on the reaction energies and barriers, single-point calculations at the meta-GGA TPSS-D3[7] and hybrid-meta-GGA PW6B95-D3[19] levels are performed using a larger def2-QZVP basis set.[11, 20] The final reaction Gibbs free energies (ΔG) are determined from the electronic single-point energies plus TPSS-D3 thermal corrections and COSMO-RS solvation free energies. The computed reaction free energies from both DFT functionals are mostly in good mutual agreement of about 0.1 ± 2.1 kcal/mol (average ± standard deviations), excluding the overbinding for each CO by about 4 kcal/mol to benzyl potassium observed at lower TPSS-D3 level. As expected, about 1.7 ± 2.2 kcal/mol (average ± standard deviations) higher barriers are observed at the PW6B95-D3 level. In our discussion, higher-level PW6B95-D3 Gibbs free energies (in kcal/mol, at 298.15 K and 1 mol/L concentration) will be used in our discussion unless specified otherwise. The applied DFT methods in combination with the large AO basis set provide usually accurate electronic energies leading to errors for chemical energies (including barriers) on the order of typically 1-2 kcal/mol. This has been tested thoroughly for the huge data base GMTKN55[20] which is the common standard in the field of DFT benchmarking. To provide inputs for the simulation of experimental EPR spectra of radicals **12** and **16**, EPR parameters such as the g-tensor and hyperfine coupling are calculated by at both the PBE0/def2-TZVP + COSMO(THF) and TPSS/def2-TZVP COSMO(THF) levels using the eprnmr module within ORCA 3.0 program,[21] leading to similar values at both PBE0 and TPSS levels.

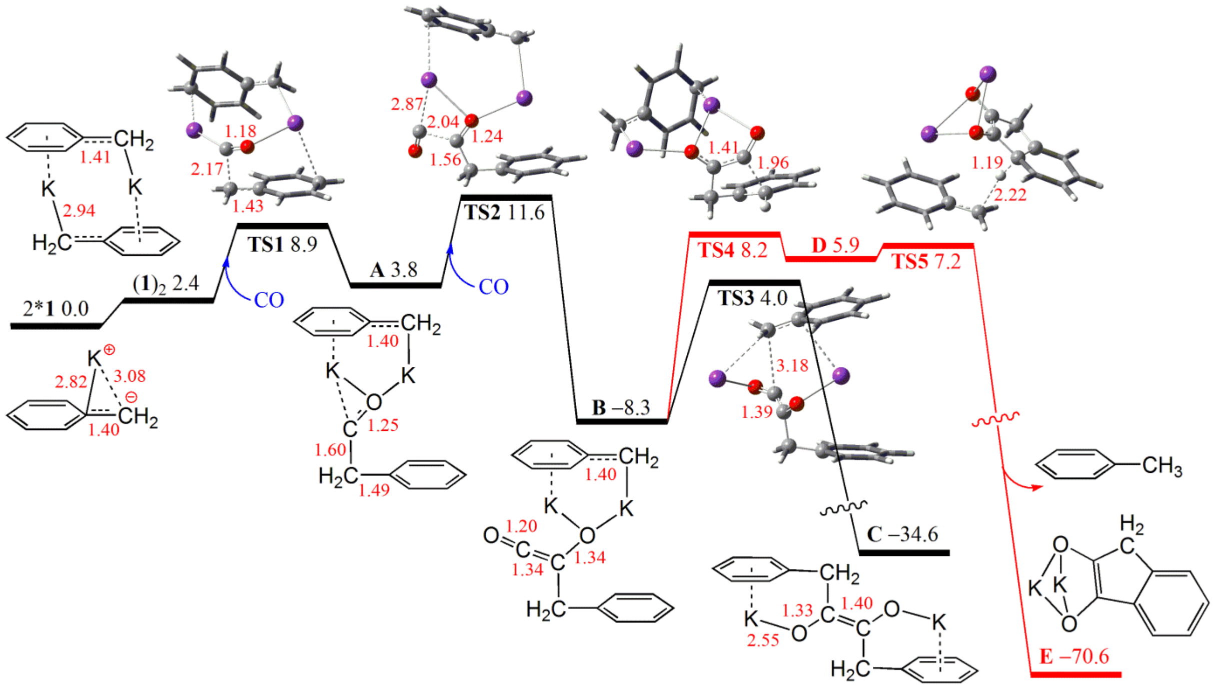

(A) Mechanism via dimeric (**1**)2

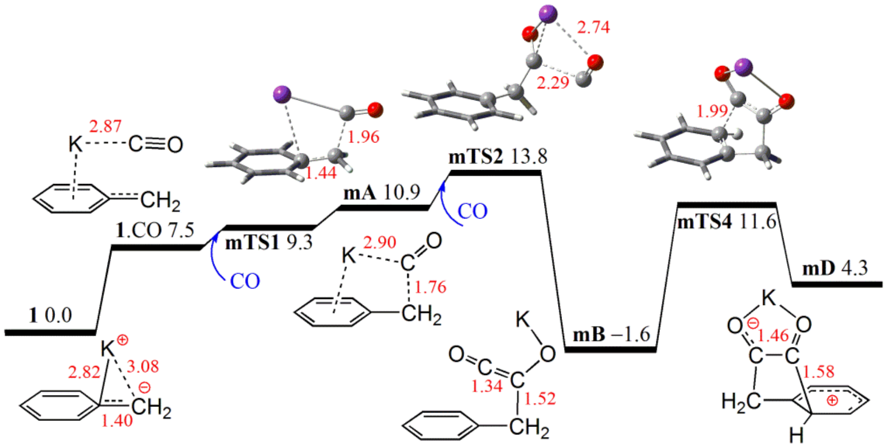

(B) Mechanism via monomeric **1**

Figure S 50. The DFT-computed Gibbs free energy paths in THF solution (in kcal/mol, at 298 K and 1M concentration) at PW6B95-D3 + COSMO-RS level for the reactions of benzyl potassium **1** KCH_2Ph with CO: (A) via dimeric (**1**)$_2$; (B) via monomeric **1**.

Without bulky substition to the phenyl ring, the dimeric mechanism (A) is kinetically favored than the monoeric mechanism (B), even though monomeric reactant **1** is dominant in THF solution. From the linear ketene-like intermediate **B** after two CO insertion, two competing processes are possible via nucleophilic additions of either the benzyl anion unit or the phenyl ring to the electrophilic kentene site, with the former process being kinetically 4.2 kcal/mol more favorable under standard (1 M concentration for all species), leading to formal carbene dimerization via ketene intermediate.

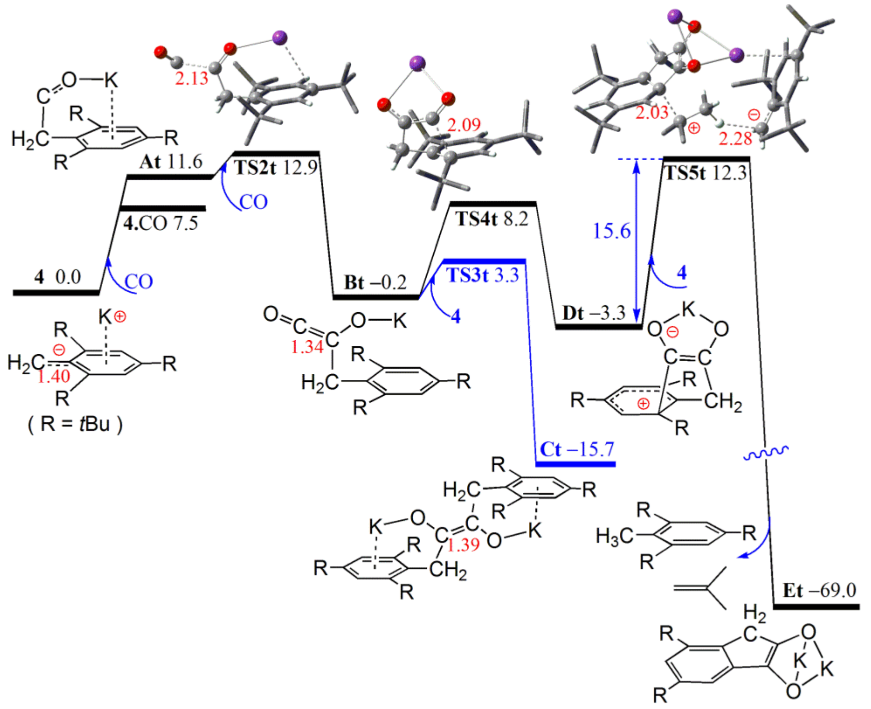

(A) Mechanism via monomeric **4**

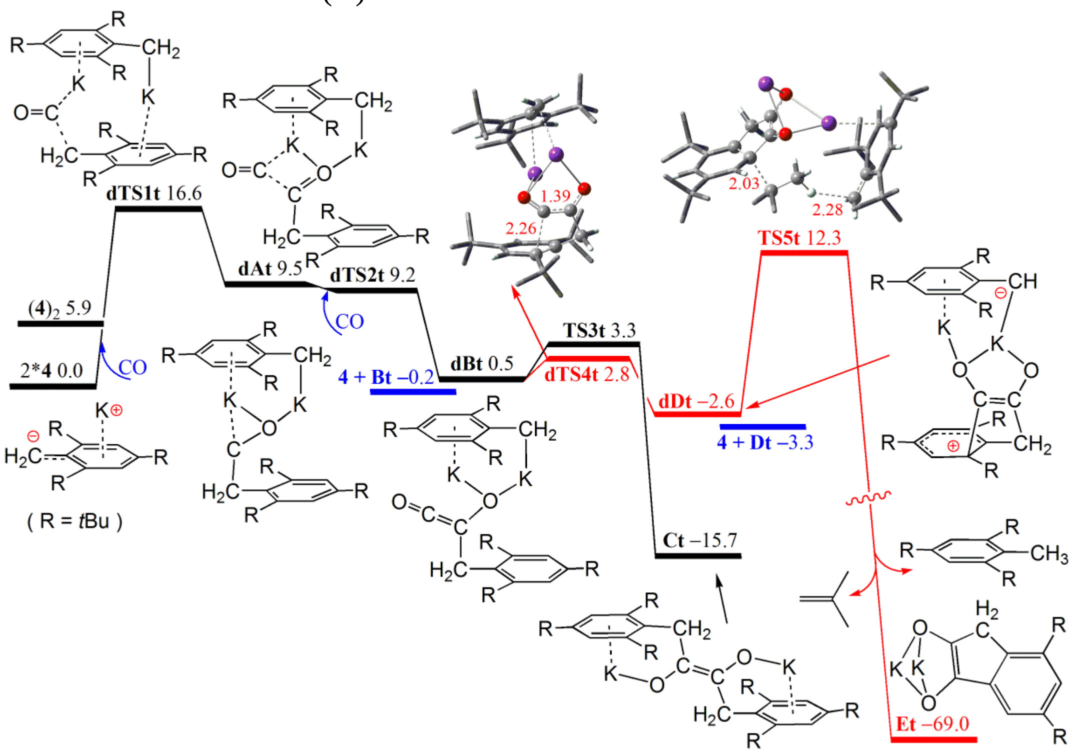

(B) Mechanism via dimeric (**4**)$_2$

Figure S 51. The DFT-computed Gibbs free energy paths in THF solution (in kcal/mol, at 298 K and 1M concentration) at PW6B95-D3 + COSMO-RS level for the reactions of bulky *tert*-butyl-substituted benzyl potassium **4** KBzt with CO: (A) via monomer **4**; (B) via dimeric (**4**)$_2$.

With additional bulky *tert*-butyl-substitution to the phenyl ring, the benzyl potassium dimer (**4**)$_2$ is further disfavored in solution and its reaction with CO becomes kinetically less favorable, with spontaneous **4** elimination (shown in blue) after two CO insertion. From the linear ketene-like intermediate **Bt** resulted from two CO insertion, two competing processes

are possible via nucleophilic additions of either the benzyl anion unit (black line) or the phenyl ring (red line) to the kentene site, with the former being kinetically more favorable and with the latter being favored at low CO pressure.

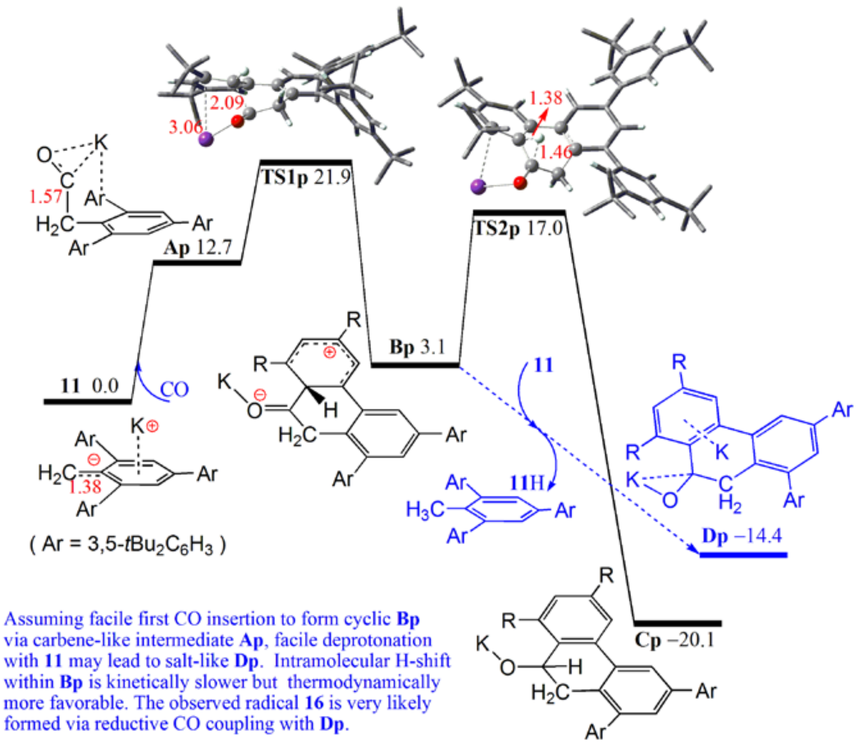

(A) the first CO insertion to bulky aryl-substituted **11**

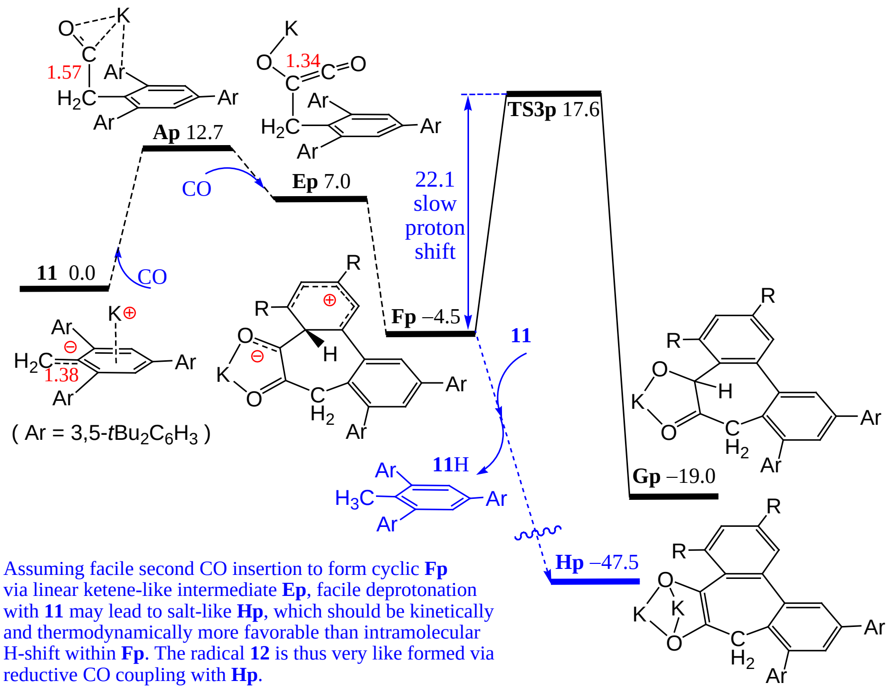

(B) the second CO insertion to bulky aryl-substituted **11**

Figure S 52. DFT-computed Gibbs free energy paths in THF solution (in kcal/mol, at 298 K and 1M concentration) at PW6B95-D3 + COSMO-RS level for the reactions of benzyl potassium **11**: (A) the first CO insertion; (B) the second CO insertion. Some potentially facile processes are shown in dashed lines. With very bulkyl aryl-substituents, only monomeric mechanism is expected.

Table S1. TPSS-D3/def2-TZVP + COSMO computed imaginary frequency (ImF), zero-point energies (ZPE), gas-phase enthalpic (Hc) and Gibbs free-energy (Gc) corrections; the COSMO-RS computed solvation enthalpic (Hsol) and Gibbs free-energy (Gsol) corrections in THF solution; TPSS-D3/def2-QZVP and PW6B95-D3/def2-QZVP single-point energies (TPSS-D3 and PW6B95-D3); the total PW6B95-D3 free energies G_P; the relative electronic energies (ΔE_T and ΔE_P) and Gibbs free-energies (ΔG_T and ΔG_P) at the TPSS-D3 and PW6B95-D3 levels. See also **Figures S1-S3** for most labellings of structures.

Reactions	ImF cm^{-1}	ZPE kcal /mol	Hc kcal /mol	Gc kcal /mol	Hsol kcal /mol	Gsol kcal /mol	TPSS-D3 Eh	PW6B95-D3 Eh	G_P Eh	ΔE_T kcal /mol	ΔE_P kcal /mol	*ΔG_P kcal /mol*	ΔG_T kcal /mol
THF (1 mol/L)	0	72.27	75.88	55.35	-6.76	-4.40	-232.60386	-232.84221	-232.75801	0.00	0.00	*0.00*	0.00
THFs (solvent)	0	72.27	75.88	55.35	-7.35	-1.01	-232.60386	-232.84221	-232.75563	0.00	0.00	*1.49*	1.49
Free K^+ is bound to single THF in solution													
K^+ + 2THFs	0	144.54	153.24	101.17	-99.53	-72.58	-1064.98810	-1065.87190	-1065.82333	0.00	0.00	*0.00*	0.00
K^+·THF + THFs	0	145.36	153.91	108.84	-78.88	-61.22	-1065.02485	-1065.90847	-1065.82956	-23.06	-22.95	*-3.91*	-4.02
$K^+(THF)_2$	0	146.61	155.49	121.49	-62.74	-53.26	-1065.05616	-1065.93958	-1065.82785	-42.71	-42.47	*-2.84*	-3.08
*Dimeric benzyl potassium is 2.4 kcal/mol less stable than two monomers of **1** KCH_2Ph in THF*													
**1** + **1**	0	141.65	152.72	100.72	-67.99	-49.53	-1742.17084	-1743.58049	-1743.49288	0.00	0.00	*0.00*	0.00
K^+·**1** + $PhCH_2^-$	0	141.72	152.80	101.15	-148.76	-126.83	-1742.03380	-1743.44348	-1743.47837	85.99	85.97	*9.11*	9.13
**(1)**$_2$	0	142.708	154.201	115.592	-37.436	-26.687	-1742.22072	-1743.63371	-1743.48902	-31.30	-33.40	*2.42*	4.51
*Benzyl potassium **1** should exist as stable contact ion pair without coordinating THF*													
**1** + THFs	0	143.09	152.24	105.71	-41.34	-25.78	-1103.68928	-1104.63246	-1104.50207	0.00	0.00	*0.00*	0.00
K^+·THF + $PhCH_2^-$	0	143.04	152.24	105.57	-129.42	-113.89	-1103.54265	-1104.48433	-1104.49156	92.01	92.95	*6.59*	5.65
**1**·THF	0	144.91	154.23	119.76	-30.13	-22.38	-1103.71093	-1104.65503	-1104.49684	-13.59	-14.17	*3.28*	3.85
*Monomer **1** reaction with CO: unstable carbene-like complex **mA***													
CO	0	3.06	5.13	-8.94	-2.12	0.07	-113.38074	-113.50012	-113.51124				
**1** + CO	0	73.88	81.49	41.42	-36.12	-24.69	-984.46616	-985.29037	-985.25768	0.00	0.00	*0.00*	0.00
**1**·CO	0	75.63	82.95	52.87	-28.62	-20.30	-984.48483	-985.30063	-985.24571	-11.72	-6.44	*7.51*	2.24
**mTS1**	120i	75.30	82.22	52.83	-29.57	-21.12	-984.48102	-985.29641	-985.24287	-9.32	-3.79	*9.29*	3.76
**mA**	0	76.60	83.55	54.21	-30.88	-22.18	-984.47835	-985.29439	-985.24033	-7.65	-2.53	*10.89*	5.76
Second CO insertion is facile and exergonic													
**1** + 2CO	0	76.94	86.63	32.48	-38.24	-24.62	-1097.84690	-1098.79049	-1098.76893	0.00	0.00	*0.00*	0.00

**mTS2**	96i	79.80	88.69	54.89	-29.99	-21.74	-1097.86553	-1098.80154	-1098.74570	-11.69	-6.94	*14.58*	9.82
**mB**	0	83.99	91.86	60.32	-42.33	-31.43	-1097.88798	-1098.82057	-1098.77153	-25.78	-18.88	*-1.63*	-8.54
...followed by facile electrophilic ketene-like attack at Ph-ring abd deprotonation with KBz													
**mTS4**	357i	82.78	90.06	59.86	-34.90	-25.33	-1097.88251	-1098.80846	-1098.75043	-22.35	-11.28	*11.60*	0.54
**mC**	0	84.83	91.81	62.29	-35.88	-26.19	-1097.89302	-1098.82260	-1098.76207	-28.94	-20.15	*4.30*	-4.49
**mC** + **1**	0	155.66	168.18	112.65	-69.88	-50.96	-1968.97844	-1970.61284	-1970.50851	0.00	0.00	*0.00*	0.00
**TS5**	124i	152.70	165.65	123.62	-50.89	-37.70	-1969.01663	-1970.64388	-1970.50395	-23.97	-19.48	*2.86*	-1.63
**E** + $PhCH_3$	0	158.06	170.25	115.88	-61.92	-45.09	-1969.10133	-1970.74668	-1970.62784	-77.12	-83.99	*-74.88*	-68.02
*Direct coupling (via transition state **TS3a**) between two unstable carbene-like mA is slow over a sizeable barrier of 21.8 kcal/mol*													
2***1** + 2CO	0	147.77	162.99	82.84	-72.23	-49.38	-1968.93232	-1970.58073	-1970.51536	0.00	0.00	*0.00*	0.00
2 **mA**	0	153.21	167.10	108.42	-61.76	-44.35	-1968.95671	-1970.58879	-1970.48067	-15.30	-5.05	*21.77*	11.52
**Ba**	0	155.56	169.36	125.38	-35.49	-25.65	-1969.02398	-1970.66589	-1970.50395	-57.52	-53.44	*7.17*	3.08
**TS3a**	174i	153.16	167.17	122.58	-41.75	-29.98	-1969.00320	-1970.64136	-1970.49079	-44.48	-38.05	*15.42*	8.99
**C**	0	158.94	171.55	130.24	-50.15	-36.84	-1969.07929	-1970.72242	-1970.57055	-92.22	-88.91	*-34.63*	-37.95
*Reaction with CO via dimeric (**1**)$_2$ complex is kinetically 3 kcal/mol more favorable than via monomer **1***													
2***1** + 2CO	0	147.77	162.99	82.84	-72.23	-49.38	-1968.93232	-1970.58073	-1970.51536	0.00	0.00	*0.00*	0.00
(**1**)$_2$ + 2CO	0	148.83	164.47	97.71	-41.67	-26.54	-1968.98220	-1970.63395	-1970.51151	-31.30	-33.40	*2.42*	4.51
**TS1** + CO	64i	149.02	163.84	107.88	-40.97	-27.60	-1968.99324	-1970.63519	-1970.50122	-38.23	-34.17	*8.87*	4.82
**A** + CO	0	152.02	166.80	111.05	-39.63	-27.00	-1969.00384	-1970.64927	-1970.50932	-44.88	-43.01	*3.80*	1.92
**TS2**	86i	152.40	166.81	121.22	-38.34	-27.83	-1969.01165	-1970.64868	-1970.49685	-49.78	-42.64	*11.62*	4.48
**B**	0	155.79	169.65	125.66	-39.00	-28.08	-1969.04470	-1970.68715	-1970.52865	-70.52	-66.78	*-8.33*	-12.08
**TS2a**	39i	152.36	166.85	121.12	-38.27	-27.60	-1969.01058	-1970.64762	-1970.49557	-49.11	-41.97	*12.42*	5.29
**Ba**	0	155.56	169.36	125.38	-35.49	-25.65	-1969.02398	-1970.66589	-1970.50395	-57.52	-53.44	*7.17*	3.08
**TS3**	122i	153.98	167.64	123.90	-45.92	-33.79	-1969.01739	-1970.65683	-1970.51022	-53.39	-47.75	*3.23*	-2.40
**C**	0	158.94	171.55	130.24	-50.15	-36.84	-1969.07929	-1970.72242	-1970.57055	-92.22	-88.91	*-34.63*	-37.95
**TS4**	363i	153.95	167.50	123.98	-42.35	-30.65	-1969.02022	-1970.65403	-1970.50230	-55.16	-46.00	*8.20*	-0.96
**D**	0	156.91	169.90	127.88	-42.26	-30.93	-1969.02606	-1970.66341	-1970.50590	-58.83	-51.88	*5.94*	-1.01
**TS5**	124i	152.70	165.65	123.62	-50.89	-37.70	-1969.01663	-1970.64388	-1970.50395	-52.91	-39.63	*7.16*	-6.11
**E** + $PhCH_3$	0	158.06	170.25	115.88	-61.92	-45.09	-1969.10133	-1970.74668	-1970.62784	-106.06	-104.14	*-70.58*	-72.50
*After H_2O hydrolysis: **3a** with two COH groups instead is 8.7 kcal/mol less stable than the final product **3**.*													
**3**	0	173.66	184.07	147.77	-20.36	-14.25	-770.29664	-771.12532	-770.90954	0.00	0.00	*0.00*	0.00

**3a**	0	173.30	183.99	147.15	-31.13	-18.55	-770.27222	-771.10356	-770.89563	15.33	13.65	*8.73*	10.41
*Effects of additional bulky tert-butyl substitution to phenyl ring of **1:***													
*Monomericbenzyl potassium **4** (KBzt) in THF solution: dimer (**4**)$_2$ is further disfavored.*													
**4** + THFs	0	353.69	372.32	307.58	-42.11	-25.49	-1575.76693	-1577.21090	-1576.75836	0.00	0.00	*0.00*	0.00
K^+·THF + Bzt^-	0	353.46	372.21	306.06	-129.32	-110.92	-1575.62483	-1577.06407	-1576.74707	89.17	92.14	*7.08*	4.12
2***4**	0	562.84	592.89	504.46	-69.52	-48.95	-2686.32616	-2688.73738	-2688.00546	0.00	0.00	*0.00*	0.00
(**4**)$_2$	56	565.53	595.64	523.15	-43.81	-30.73	-2686.37256	-2688.78384	-2687.99610	-29.12	-29.15	*5.87*	5.91
*Reaction of KBzt monomer **4** with CO is kinetically facile*													
**4** + 2CO	0	287.54	306.71	234.34	-39.00	-24.33	-1569.92456	-1571.36893	-1571.02522	0.00	0.00	*0.00*	0.00
**TS1t** + CO	52i	287.22	306.42	243.51	-36.93	-24.61	-1569.93354	-1571.36757	-1571.01272	-5.63	0.85	*7.84*	1.36
**4.**CO + CO	0	289.81	308.46	246.92	-34.33	-22.38	-1569.94199	-1571.37706	-1571.01321	-10.94	-5.10	*7.53*	1.69
**At** + CO	0	291.04	309.16	248.69	-41.77	-28.71	-1569.92744	-1571.36333	-1571.00674	-1.81	3.52	*11.59*	6.27
**TS2t**	88i	289.01	307.88	254.84	-40.76	-29.87	-1569.93647	-1571.36622	-1571.00470	-7.48	1.70	*12.87*	3.70
**Bta**	0	294.73	312.03	263.00	-41.06	-30.34	-1569.96578	-1571.39808	-1571.02430	-25.87	-18.29	*0.58*	-7.00
**Bt**	0	294.79	312.07	263.08	-45.44	-33.26	-1569.96360	-1571.39485	-1571.02560	-24.50	-16.26	*-0.24*	-8.48
**TS4t**	229i	291.26	308.88	259.22	-38.23	-27.42	-1569.95982	-1571.38461	-1571.01219	-22.13	-9.84	*8.17*	-4.11
**Dt**	0	294.94	311.50	264.03	-42.47	-30.63	-1569.97779	-1571.40536	-1571.03041	-33.40	-22.86	*-3.26*	-13.81
*…Nucleophilic trapping of ketene-like **Bt** with **4** is kinetically more facile*													
2***4** + 2CO	0	568.96	603.15	486.57	-73.76	-48.81	-2913.08764	-2915.73762	-2915.02795	0.00	0.00	*0.00*	0.00
**TS3t**	66i	570.75	605.27	523.45	-50.69	-36.51	-2913.16800	-2915.80169	-2915.02268	-50.43	-40.20	*3.31*	-6.92
**Ct**	0	582.20	612.76	539.48	-57.37	-40.70	-2913.21400	-2915.85090	-2915.05302	-79.29	-71.08	*-15.73*	-23.94
*…further reaction of 5-ring **Dt** with another **4** to eliminate tBu*													
**Dt** + 4	0	576.37	607.94	516.26	-77.23	-55.11	-2913.14087	-2915.77405	-2915.03314	-33.40	-22.86	*-3.26*	-13.81
**Dt**.K^++ Bzt^-	0	576.85	608.38	516.78	-141.05	-117.27	-2913.03206	-2915.66497	-2915.02230	34.88	45.59	*3.54*	-7.17
**TS5t**	459i	570.23	604.43	523.15	-56.36	-40.85	-2913.12354	-2915.77989	-2915.00829	-22.53	-26.52	*12.34*	16.33
**Et** + **4**H + Me_2CCH_2	0	575.19	607.32	501.06	-79.95	-56.49	-2913.21487	-2915.85540	-2915.13790	-79.84	-73.91	*-68.99*	-74.93
K+ induced tBu-elimination is kinetically 2.5 kcal/mol less favorable													
**Dt**.K^+	0	296.49	314.20	264.21	-83.26	-66.56	-2169.82822	-2171.66716	-2171.34916	0.00	0.00	*0.00*	0.00
**TS5t**$^+$	465i	291.00	310.18	257.09	-82.57	-65.45	-2169.80916	-2171.63951	-2171.33110	11.96	17.35	*11.33*	5.95
**Et**.tBu^+	0	293.44	312.12	260.01	-86.74	-68.61	-2169.83954	-2171.66983	-2171.36180	-7.10	-1.68	*-7.93*	-13.36
*High barrier of 25.6 kcal/mol for direct coupling via two carbene-like **At***													

2*4 + 2CO	0	568.96	603.15	486.57	-73.76	-48.81	-2913.08764	-2915.73762	-2915.02795	0.00	0.00	*0.00*	0.00
**(At)**$_2$	0	577.36	609.85	532.60	-43.76	-31.36	-2913.17255	-2915.81084	-2915.00904	-53.28	-45.95	*11.86*	4.53
**TS3td**	130i	569.70	604.25	522.05	-51.95	-36.50	-2913.13218	-2915.76393	-2914.98716	-27.95	-16.51	*25.60*	14.16
**Ct**	0	582.20	612.76	539.48	-57.37	-40.70	-2913.21400	-2915.85090	-2915.05302	-79.29	-71.08	*-15.73*	-23.94
*Reaction via unstable (**4**)$_2$ (K$_2$Bzt$_2$) dimer with CO is kinetically 3.7 kcal/mol less favorable (**dTS1t** vs. **TS2t**)*													
2***4** + 2CO	0	568.96	603.15	486.57	-73.76	-48.81	-2913.08764	-2915.73762	-2915.02795	0.00	0.00	*0.00*	0.00
**dTS1t** + CO	47i	567.10	602.83	508.19	-52.68	-35.74	-2913.12228	-2915.76034	-2915.00142	-21.74	-14.26	*16.65*	9.17
**dAt** + CO	0	574.98	608.11	519.68	-43.88	-29.70	-2913.15638	-2915.79968	-2915.01282	-43.14	-38.94	*9.49*	5.30
**dTS2t**	77i	570.38	605.19	522.74	-41.08	-29.69	-2913.16568	-2915.80208	-2915.01334	-48.97	-40.45	*9.16*	0.64
**dBt**	0	578.43	610.89	533.84	-40.26	-28.75	-2913.19532	-2915.83502	-2915.02710	-67.58	-61.12	*0.53*	-5.93
**Bt** + **4**	0	576.21	608.51	515.31	-80.20	-57.73	-2913.12668	-2915.76354	-2915.02833	-24.50	-16.26	*-0.24*	-8.48
**TS3t**	66i	570.75	605.27	523.45	-50.69	-36.51	-2913.16800	-2915.80169	-2915.02268	-50.43	-40.20	*3.31*	-6.92
**Ct**	0	582.20	612.76	539.48	-57.37	-40.70	-2913.21400	-2915.85090	-2915.05302	-79.29	-71.08	*-15.73*	-23.94
**dTS4t**	139i	571.19	605.56	524.07	-42.24	-30.34	-2913.18048	-2915.81331	-2915.02348	-58.26	-47.49	*2.80*	-7.97
**dDt**	0	580.70	612.04	537.35	-42.70	-30.79	-2913.20392	-2915.84228	-2915.03203	-72.97	-65.68	*-2.56*	-9.85
**Dt** + **4**	0	576.37	607.94	516.26	-77.23	-55.11	-2913.14087	-2915.77405	-2915.03314	-33.40	-22.86	*-3.26*	-13.81
*After H_2O hydrolysis: Enol **8** with two CHOH groups is 7.6 kcal/mol less stable than **8** with one C=O and one CHOH sites*													
**8**	0	593.27	623.28	551.16	-34.35	-24.79	-1714.42957	-1716.25582	-1715.41398	0.00	0.00	*0.00*	0.00
**8a**	0	594.30	624.19	552.53	-34.24	-24.23	-1714.42071	-1716.24683	-1715.40192	5.56	5.64	*7.57*	7.49
Effects of very bulky aryl-substitution													
*Monomeric **11p** (KBzp with three Ph-substituents) in THF solution; Stable monomer **11** (KBzpt) with further bulky tBu-substitution is expected in solution*													
**11p** + THFs	0	294.37	312.00	247.66	-55.73	-36.69	-1797.32881	-1799.04367	-1798.70446	0.00	0.00	*0.00*	0.00
Bzp^- + K^+·THF	0	294.90	312.32	247.30	-134.52	-115.04	-1797.20440	-1798.91361	-1798.69683	78.07	81.61	*4.78*	1.25
2***11p**	0	444.20	472.23	384.63	-96.76	-71.36	-3129.44991	-3132.40291	-3131.89766	0.00	0.00	*0.00*	0.00
**(11p)**$_2$	0	444.26	473.25	400.37	-57.83	-43.85	-3129.50558	-3132.46201	-3131.89085	-34.93	-37.09	*4.27*	6.43
*Stable contact ion pair of radical **16** in solution*													
**16** + THFs	0	721.38	757.20	660.42	-72.05	-48.14	-2854.32834	-2857.15858	-2856.17984	0.00	0.00	*0.00*	0.00
**16**$^-$ + K^+·THF	0	721.53	757.19	659.44	-150.38	-125.51	-2854.20399	-2857.02834	-2856.17144	78.03	81.73	*5.27*	1.58
*Facile reaction of very bulky benzyl potassium **11** (KBzpt) with CO*													
**11** + CO	0	646.54	681.91	589.29	-66.37	-46.40	-2622.29117	-2624.88707	-2624.01589	0.00	0.00	*0.00*	0.00
**Ap**	0	649.54	684.13	603.04	-63.16	-46.36	-2622.29555	-2624.88582	-2623.99569	-2.75	0.78	*12.68*	9.15

**TS1p**	291i	641.62	678.27	592.23	-59.75	-43.29	-2622.27346	-2624.85887	-2623.98106	11.12	17.70	*21.86*	15.28
**Bp**	0	649.94	683.95	604.07	-63.36	-46.26	-2622.31220	-2624.90293	-2624.01099	-13.19	-9.95	*3.08*	-0.16
**TS2p**	1040i	640.92	676.74	592.65	-65.23	-47.60	-2622.27039	-2624.86044	-2623.98884	13.04	16.71	*16.98*	13.31
**Cp**	44	651.05	684.78	605.59	-64.57	-47.22	-2622.34915	-2624.94068	-2624.04785	-36.38	-33.64	*-20.05*	-22.79
*KH salt (modeled as tetramer K_4H_4 in solution) elimination from 6-ring **Bp** to form the cyclic ketone **17** is energetically favorable*													
**Bp**	0	649.94	683.95	604.07	-63.36	-46.26	-2622.31220	-2624.90293	-2624.01099	0.00	0.00	*0.00*	0.00
**17** + 0.25*K_4H_4	0	644.76	679.35	594.72	-60.02	-43.09	-2622.30695	-2624.89831	-2624.01546	3.29	2.90	*-2.81*	-2.41
*…but **14** cannot be reduced by **11** into radical **16***													
**17** + **11**	0	1285.80	1351.99	1195.78	-110.70	-80.07	-4530.66154	-4535.32287	-4533.53886	0.00	0.00	*0.00*	0.00
**16** + Bzpt	0	1300.37	1362.51	1214.55	-109.33	-79.38	-4530.63498	-4535.29165	-4533.47662	16.67	19.59	*39.05*	36.13
*… **Bp** cannot transfer H-atom to CO to form the observed radical **16***													
**Bp** + CO	45	653.64	689.52	595.93	-65.54	-46.21	-2735.69287	-2738.40299	-2737.52094	0.00	0.00	*0.00*	0.00
**16** + HCO	0	657.71	692.23	600.61	-69.66	-49.05	-2735.65134	-2738.35534	-2737.47036	26.06	29.90	*31.73*	27.90
*…**Bp** can be efficiently deprotonated by **11**, but cannot form radical **16** directly*													
**Bp** + **11**	0	1293.43	1360.72	1202.31	-127.61	-92.73	-5131.22263	-5136.28988	-5134.51564	0.00	0.00	*0.00*	0.00
**Dp** + BzptH	0	1292.14	1359.99	1200.43	-119.56	-86.51	-5131.25849	-5136.32472	-5134.54356	-22.51	-21.86	*-17.52*	-18.16
**Cp** + **11**	44	1294.54	1361.56	1203.83	-128.82	-93.69	-5131.25958	-5136.32763	-5134.55250	-23.19	-23.69	*-23.13*	-22.63
**16** + K·BzptH	0	1305.99	1370.05	1218.33	-129.13	-93.11	-5131.22864	-5136.29539	-5134.49621	-3.77	-3.46	*12.19*	11.88
*…**Bp** deprotonation with **Dp** to release KH salt is also endergonic*													
**Bp** + **Dp**	0	1293.82	1362.48	1201.50	-138.08	-100.49	-5844.01489	-5849.60420	-5847.84361	0.00	0.00	*0.00*	0.00
2***16** + 0.25*K_4H_4	0	1301.70	1367.64	1208.59	-144.69	-104.90	-5844.00498	-5849.59651	-5847.83090	6.21	4.83	*7.98*	9.36
*Second CO insertion to **11** is kinetically also facile to form 7-ring **Fp***													
**11** + 2CO	0	649.60	687.04	580.35	-68.49	-46.33	-2735.67191	-2738.38719	-2737.52714	0.00	0.00	*0.00*	0.00
**Ap** + CO	0	652.60	689.26	594.10	-65.28	-46.29	-2735.67629	-2738.38594	-2737.50693	-2.75	0.78	*12.68*	9.15
**Ep**	0	658.72	693.57	611.95	-62.79	-46.52	-2735.71755	-2738.42005	-2737.51597	-28.64	-20.62	*7.01*	-1.02
**Fp**	0	656.42	691.27	609.88	-64.84	-47.40	-2735.73142	-2738.43364	-2737.53426	-37.34	-29.15	*-4.47*	-12.67
**TS3p**	1012i	646.75	683.72	597.31	-66.04	-48.37	-2735.67737	-2738.37682	-2737.49902	-3.43	6.51	*17.64*	7.71
**Gp**	13	655.59	690.61	608.67	-65.25	-47.96	-2735.74701	-2738.45394	-2737.55739	-47.13	-41.89	*-18.98*	-24.22
*…followed by rapid proton transfer to another **11***													
**Fp** + **11**	0	1299.91	1368.04	1208.12	-129.09	-93.88	-5244.64185	-5249.82059	-5248.03891	0.00	0.00	*0.00*	0.00
**Hp** + BzptH	0	1300.75	1369.12	1208.66	-117.60	-85.25	-5244.72174	-5249.90380	-5248.10752	-50.13	-52.22	*-43.05*	-40.96

Table S2. TPSS-D3/def2-TZVP + COSMO optimized Cartesian coordinates (in Å) in THF solution for the reactions of benzyl potassium **1** KCH_2Ph with CO. Each structure is labeled by the specific name (See also **Figure S1** and **Table S1**), followed by the number of atoms, the total energy (in hartrees), and the detailed atomic coordinates (in double-column text list).

1.CO : loose adduct of 1 and CO
17
Energy = -984.4697625487
K 0.6136700 0.1650474 -0.4561627
C 1.4462169 -1.7763002 2.2652245
H 2.3518957 -1.4865323 2.7926198
H 0.6118528 -2.0957079 2.8850432
C 1.5463506 -2.2630933 0.9433379
C 2.7216292 -2.0691706 0.1372967
C 0.4377336 -2.8740761 0.2604912
C 2.7710230 -2.4393410 -1.2025959
H 3.5999420 -1.6230292 0.6014000
C 0.5027570 -3.2386447 -1.0803082
H -0.4769836 -3.0604994 0.8212809
C 1.6648094 -3.0256059 -1.8424541
H 3.6898615 -2.2718835 -1.7621167
H -0.3671677 -3.7014616 -1.5433896
H 1.7102694 -3.3146590 -2.8879080
C 0.6647137 0.4741312 2.3990801
O 0.6448206 0.7216096 3.5356110

(**1**)$_2$.THF : THF-coordinated dimer (**1**)$_2$
43
Energy = -1974.796093175
C -1.6866960 1.5811647 -2.3102517
H -1.6283688 2.6520591 -2.4880536
H -1.5575378 0.9373924 -3.1802019
C -2.3248045 1.0896129 -1.1656245
C -2.7680237 1.9421077 -0.0842125
C -2.5696796 -0.3185637 -0.9403828
C -3.4525375 1.4527085 1.0175304
H -2.6005915 3.0143729 -0.1718226
C -3.2565358 -0.7911314 0.1714967
H -2.2690983 -1.0307930 -1.7096452
C -3.7208156 0.0759620 1.1777900
H -3.7932005 2.1556968 1.7768328
H -3.4369812 -1.8616104 0.2581944
H -4.2767275 -0.2958366 2.0328430
K 0.6378733 -0.1022073 -1.6014448
C 1.4459505 -1.8216356 2.3869059
H 2.3372527 -1.4729291 2.9024974
H 0.6398897 -2.2070711 3.0114062
C 1.5001425 -2.1590165 1.0309709
C 2.6659350 -1.9273883 0.2044908
C 0.3859593 -2.7488854 0.3194405
C 2.7322110 -2.3281553 -1.1220845
H 3.5458017 -1.4825382 0.6669420
C 0.4702385 -3.1408109 -1.0099874
H -0.5339648 -2.9507782 0.8680873
C 1.6408075 -2.9452078 -1.7686744
H 3.6563515 -2.1598228 -1.6736572
H -0.3975426 -3.6081322 -1.4730898
H 1.7065023 -3.2719867 -2.8016150
K -0.7810626 0.0777632 1.9172093
O 1.0253447 1.6310581 0.5653648
C 2.3496867 1.6839978 1.1587199
C 3.1796753 2.4913578 0.1585179
C 2.1418649 3.4488325 -0.4925589
C 0.7869673 2.9776083 0.0679597
H 2.6733392 0.6533938 1.3080466
H 2.2824247 2.1939385 2.1297280
H 3.9957719 3.0275346 0.6487564
H 3.6171514 1.8262109 -0.5922093
H 2.3283461 4.4933849 -0.2322148
H 2.1649641 3.3670478 -1.5827046
H 0.4572326 3.6058849 0.9051488
H -0.0090892 2.8984896 -0.6760041

(**1**)$_2$: dimeric benzyl potassium $(KCH_2Ph)_2$
30
Energy = -1742.184294888
C -1.7176179 -2.0152760 -0.1080164
H -1.8561387 -2.6710151 -0.9680110
H -1.3368640 -2.4865092 0.7990514
C -2.5245843 -0.8716056 0.0142893
C -3.3141403 -0.3483719 -1.0750878
C -2.6034850 -0.0952584 1.2288716
C -4.0759185 0.8069721 -0.9569783
H -3.3171910 -0.8929697 -2.0184025
C -3.3703475 1.0577428 1.3296775
H -2.0425949 -0.4388576 2.0968838
C -4.1137782 1.5507440 0.2388244

H	-4.6546987	1.1433083	-1.8162167
H	-3.3897116	1.5939597	2.2774141
H	-4.7194948	2.4469260	0.3278016
K	1.1542036	-1.8533722	-0.6973103
C	1.7207165	2.0159971	-0.1114979
H	1.8683549	2.6678718	-0.9728458
H	1.3371654	2.4935893	0.7912316
C	2.5250309	0.8716614	0.0236775
C	3.3256754	0.3420537	-1.0542913
C	2.5902714	0.1009898	1.2426191
C	4.0860926	-0.8129958	-0.9221928
H	3.3394861	0.8811932	-2.0006890
C	3.3558602	-1.0513249	1.3579278
H	2.0200331	0.4487983	2.1028423
C	4.1112455	-1.5501405	0.2780499
H	4.6738885	-1.1534559	-1.7736183
H	3.3649717	-1.5821800	2.3088423
H	4.7160516	-2.4457631	0.3780946
K	-1.1524818	1.8472886	-0.6996614

1H : $PhCH_3$ from protonation of **1**
15
Energy = -271.7322731626

C	2.4239912	-0.0000025	0.0083833
H	2.8266671	0.8892173	-0.4864052
H	2.8266749	-0.8882195	-0.4882026
C	0.9144563	0.0000112	-0.0111015
C	0.1948251	1.2029504	-0.0090336
C	0.1948241	-1.2029355	-0.0091197
C	-1.2012257	1.2059378	0.0014542
C	-1.2012107	-1.2059299	0.0022179
C	-1.9055229	0.0000031	0.0087558
H	-1.7387993	2.1505554	-0.0001714
H	-1.7387676	-2.1505541	0.0022736
H	0.7349641	-2.1472819	-0.0197235
H	-2.9919565	-0.0000085	0.0146804
H	0.7349706	2.1473105	-0.0183777
H	2.7991322	-0.0010536	1.0399790

1r : $PhCH_2$ radical
14
Energy = -271.0826261059

C	1.4035174	-1.7272865	2.2060812
H	2.2402742	-1.2323608	2.6896040
H	0.4914349	-1.8675120	2.7781941
C	1.4987473	-2.1752557	0.8751685
C	2.6938091	-1.9964294	0.1159220
C	0.4042940	-2.8270147	0.2317553
C	2.7825494	-2.4426325	-1.1933266
H	3.5414307	-1.5007825	0.5829877
C	0.5049852	-3.2681536	-1.0783475
H	-0.5179439	-2.9741313	0.7885844
C	1.6919759	-3.0813125	-1.8030324
H	3.7039263	-2.2971192	-1.7509483
H	-0.3416535	-3.7627816	-1.5470061
H	1.7659892	-3.4284884	-2.8293768

1.THF : THF-coordinated **1** (KCH_2Ph)
28
Energy = -1103.685124001

K	1.1350080	-0.4323311	-1.4296135
C	2.5403948	-1.5737973	1.7512899
H	3.5831223	-1.2787733	1.8351728
H	1.8801789	-1.3436382	2.5836218
C	2.0870259	-2.3073463	0.6617643
C	2.9354706	-2.6697046	-0.4600195
C	0.7057796	-2.7307505	0.5113780
C	2.4435029	-3.2936841	-1.5966021
H	3.9950582	-2.4226742	-0.4037808
C	0.2369940	-3.3527996	-0.6358821
H	0.0150169	-2.5303671	1.3295339
C	1.0789886	-3.6280044	-1.7351167
H	3.1354136	-3.5253209	-2.4059493
H	-0.8156585	-3.6297458	-0.6866491
H	0.7054277	-4.1266136	-2.6238392
O	1.0280463	1.9198273	-0.1538211
C	1.4455125	1.9241959	1.2362467
C	2.6139990	2.9172352	1.2936838
C	2.3618173	3.8667977	0.0880164
C	1.0875705	3.3045006	-0.5638507
H	1.7172832	0.8950140	1.4964388
H	0.6018842	2.2595514	1.8563882
H	2.6368578	3.4522686	2.2464960
H	3.5655752	2.3914199	1.1773437
H	2.2209402	4.9049769	0.3996864
H	3.2010918	3.8342618	-0.6114468
H	0.1903258	3.8235409	-0.1978148
H	1.0944604	3.3241350	-1.6559370

1$^-$: $PhCH_2^-$ anion
14
Energy = -271.1745054978

C	1.4008921	-1.7161231	2.2341448
H	2.2374609	-1.2198699	2.7218636
H	0.4884287	-1.8547987	2.8104286
C	1.4960680	-2.1622913	0.9153003
C	2.6880136	-2.0024980	0.1099641
C	0.4119656	-2.8279252	0.2250171
C	2.7721278	-2.4486560	-1.1995481
H	3.5502328	-1.5096149	0.5604415
C	0.5164017	-3.2667223	-1.0855286
H	-0.5215076	-2.9861570	0.7661686
C	1.6943283	-3.0928145	-1.8390310
H	3.7035270	-2.2934517	-1.7456746
H	-0.3427608	-3.7607124	-1.5412735
H	1.7681580	-3.4396261	-2.8660132

1 : benzyl potassium KCH_2Ph
15
Energy = -871.0819229817

K	0.5700718	0.2546955	-0.1260718
C	1.3604452	-1.6106223	2.1923400
H	2.2299795	-1.2116487	2.7115361
H	0.4883295	-1.8451055	2.8000796
C	1.5056214	-2.1877406	0.9221077
C	2.7015435	-2.0363784	0.1245048
C	0.4246128	-2.8623212	0.2397143
C	2.7731767	-2.4501575	-1.1991789
H	3.5702686	-1.5639348	0.5828528
C	0.5166689	-3.2686861	-1.0849865
H	-0.5001361	-3.0396037	0.7885689
C	1.6825897	-3.0611750	-1.8447887
H	3.7031731	-2.2967506	-1.7457345
H	-0.3403969	-3.7628645	-1.5415079
H	1.7474602	-3.3842722	-2.8792482

2 : product $((Ph_3SiO)(PhCH_2)C)_2$
100
Energy = -2739.032023059

C	-1.5451866	-0.2474986	1.1980243
H	-1.6979833	0.8283261	1.3395511
H	-2.4908289	-0.6675564	0.8419694
C	-1.1815714	-0.8802255	2.5313356
C	0.0157013	-0.5377059	3.1757242
C	-2.0312582	-1.8078805	3.1420383
C	0.3433724	-1.0951842	4.4098001
H	0.6965841	0.1570048	2.6940358
C	-1.6950943	-2.3847352	4.3687236
H	-2.9520132	-2.0991217	2.6441214
C	-0.5087812	-2.0256505	5.0099890
H	1.2750457	-0.8181646	4.8957045
H	-2.3582551	-3.1194527	4.8171305
H	-0.2458076	-2.4729305	5.9647438
C	-0.5046687	-0.4322529	0.1247642
O	-0.6447700	-1.5374996	-0.6988603
C	1.5352230	0.2471616	-1.1964861
H	2.4805428	0.6684877	-0.8411585
H	1.6893989	-0.8286426	-1.3369530
C	1.1709322	0.8776595	-2.5306279
C	2.0227903	1.8007813	-3.1451538
C	-0.0285765	0.5375314	-3.1720554
C	1.6865231	2.3756562	-4.3727042
H	2.9451610	2.0904393	-2.6492737
C	-0.3563710	1.0928899	-4.4070725
H	-0.7109515	-0.1537161	-2.6875347
C	0.4980128	2.0187521	-5.0111502
H	2.3513652	3.1070224	-4.8241017
H	-1.2898156	0.8178044	-4.8906640
H	0.2350083	2.4644721	-5.9666321
C	0.4947753	0.4316019	-0.1231685
O	0.6344080	1.5364118	0.7010497
Si	-0.3662116	-3.1617242	-0.4116546
Si	0.3646732	3.1619833	0.4117684
C	-1.8352471	-3.9089166	0.4708218
C	-1.7134443	-4.9173000	1.4405980
C	-3.1279828	-3.4600469	0.1430230
C	-2.8377499	-5.4413036	2.0803135
H	-0.7286087	-5.2897203	1.7104396
C	-4.2551620	-3.9788655	0.7784537
H	-3.2513904	-2.6858609	-0.6114321
C	-4.1098298	-4.9678045	1.7553174
H	-2.7215060	-6.2131630	2.8364447
H	-5.2445665	-3.6124033	0.5178090
H	-4.9856820	-5.3689709	2.2580906
C	-0.2095449	-3.8817657	-2.1301905
C	0.2975161	-3.1316780	-3.2050443
C	-0.5457919	-5.2268126	-2.3634768
C	0.4640136	-3.7024208	-4.4673562
H	0.5538506	-2.0863920	-3.0579267
C	-0.3779773	-5.8045980	-3.6225160
H	-0.9515169	-5.8287046	-1.5526894
C	0.1284202	-5.0417074	-4.6776729
H	0.8530984	-3.1032551	-5.2865499
H	-0.6459439	-6.8456011	-3.7830197

H	0.2567373	-5.4882136	-5.6601061
C	1.2225360	-3.4075979	0.5415773
C	2.4345275	-3.5791297	-0.1530463
C	1.2667210	-3.3617501	1.9458149
C	3.6468315	-3.6924650	0.5267732
H	2.4263119	-3.6282644	-1.2399503
C	2.4763472	-3.4774029	2.6305053
H	0.3502846	-3.2249305	2.5121958
C	3.6682605	-3.6415403	1.9227633
H	4.5722679	-3.8223966	-0.0281687
H	2.4876861	-3.4316463	3.7160085
H	4.6111065	-3.7303059	2.4560906
C	0.2140325	3.8836273	2.1302517
C	0.5569938	5.2271660	2.3624274
C	-0.2940577	3.1362088	3.2064575
C	0.3946285	5.8060743	3.6216810
H	0.9635642	5.8270227	1.5505475
C	-0.4551608	3.7080699	4.4689825
H	-0.5560077	2.0922489	3.0599861
C	-0.1128495	5.0458293	4.6782134
H	0.6676874	6.8459009	3.7812752
H	-0.8451248	3.1109353	5.2892480
H	-0.2368933	5.4931657	5.6608180
C	1.8372318	3.9007306	-0.4721520
C	1.7194534	4.9080067	-1.4435733
C	3.1281858	3.4473347	-0.1436018
C	2.8457906	5.4265495	-2.0841233
H	0.7360533	5.2838777	-1.7138535
C	4.2573925	3.9605675	-0.7799485
H	3.2486011	2.6741892	0.6124093
C	4.1159683	4.9484601	-1.7584629
H	2.7325711	6.1976929	-2.8414416
H	5.2453770	3.5907141	-0.5186661
H	4.9933997	5.3453177	-2.2619052
C	-1.2230328	3.4158773	-0.5406735
C	-2.4332743	3.5970359	0.1545810
C	-1.2683502	3.3678152	-1.9448436
C	-3.6450899	3.7172844	-0.5249562
H	-2.4249835	3.6479289	1.2413244
C	-2.4775459	3.4902850	-2.6290501
H	-0.3532048	3.2239197	-2.5115415
C	-3.6678544	3.6638767	-1.9208060
H	-4.5691587	3.8545792	0.0304832
H	-2.4898629	3.4425411	-3.7144697
H	-4.6104252	3.7580562	-2.4537346

3a : less stable enol isomer of **3**

34

Energy = -770.2373329206

C	-1.5004651	1.2410729	-0.0834467
H	-1.4301438	1.9227804	0.7716456
H	-1.3718280	1.8431494	-0.9926937
C	-2.8773372	0.5990970	-0.0960490
C	-3.4408490	0.1059965	1.0890138
C	-3.5760379	0.4307155	-1.2976847
C	-4.6810669	-0.5292642	1.0741696
H	-2.9009166	0.2224005	2.0258074
C	-4.8169353	-0.2139136	-1.3166513
H	-3.1509528	0.8164926	-2.2217342
C	-5.3720933	-0.6931637	-0.1307031
H	-5.1101605	-0.8985894	2.0015177
H	-5.3477610	-0.3356828	-2.2567322
H	-6.3379006	-1.1902897	-0.1416699
C	-0.4279973	0.1844092	-0.0126099
O	-0.5018025	-0.7976846	-1.0036584
C	1.6135188	-0.9640734	0.9136433
H	1.7642669	-1.2517901	1.9611773
H	1.2154480	-1.8272059	0.3738182
C	2.9432717	-0.5437194	0.3114046
C	4.0645661	-0.3250099	1.1178441
C	3.0599792	-0.3489258	-1.0718710
C	5.2807754	0.0755135	0.5581567
H	3.9846664	-0.4698321	2.1928105
C	4.2724243	0.0481266	-1.6348079
H	2.1917259	-0.5099194	-1.7061952
C	5.3885639	0.2626099	-0.8205438
H	6.1422949	0.2410019	1.1998175
H	4.3483466	0.1898170	-2.7097895
H	6.3335195	0.5720491	-1.2586416
C	0.5890218	0.1317723	0.8645252
O	0.8433486	1.1138387	1.8103122
H	-1.4478410	-0.9752761	-1.1686999
H	0.2563506	1.8734970	1.6585183

3 : product $PhCH_2C(O)CH(OH)CH_2Ph$

34

Energy = -770.2610843127

C	-0.7322080	-0.8245145	1.4747864
H	-1.2141751	-1.1322031	2.4074051
H	0.2792107	-0.4700783	1.7025151
C	-1.5035537	0.2940253	0.7960947
C	-2.8371702	0.0992562	0.4124309

C	-0.8923359	1.5236058	0.5334192
C	-3.5463614	1.1190327	-0.2218487
H	-3.3226414	-0.8533153	0.6119784
C	-1.6012159	2.5451097	-0.1001388
H	0.1439896	1.6786003	0.8201957
C	-2.9291355	2.3455781	-0.4807812
H	-4.5798708	0.9561198	-0.5145571
H	-1.1136102	3.4955678	-0.2985029
H	-3.4807712	3.1396084	-0.9761311
C	-0.6362171	-2.0467064	0.5874720
O	-1.1294516	-3.1234558	0.9100594
C	1.6126715	-1.7439378	-0.5985354
H	1.9890943	-2.3956123	0.1971121
H	2.0374533	-2.1036483	-1.5417172
C	2.0348395	-0.3140017	-0.3662582
C	1.8456520	0.6480931	-1.3685616
C	2.6239659	0.0850001	0.8397557
C	2.2268114	1.9734566	-1.1682408
H	1.3955887	0.3560890	-2.3146057
C	3.0044384	1.4131879	1.0469562
H	2.7896805	-0.6517586	1.6228174
C	2.8044286	2.3621691	0.0433207
H	2.0696585	2.7050020	-1.9558606
H	3.4567641	1.7039515	1.9909642
H	3.0978115	3.3958587	0.2022973
C	0.0757216	-1.9530457	-0.7623180
O	-0.1697226	-3.1482312	-1.4895559
H	-0.3351849	-1.0988354	-1.3168786
H	-0.6235850	-3.7385862	-0.8476592

A : carbene-like adduct of dimer (**1**)$_2$ and CO

32

Energy = -1855.581713683

C	-2.1130687	-2.1259874	0.4135021
H	-2.1964799	-2.9226286	-0.3258215
H	-1.7581856	-2.4139461	1.4025267
C	-2.8791306	-0.9599042	0.2635909
C	-3.5765753	-0.6284790	-0.9578489
C	-3.0243039	0.0252801	1.3106026
C	-4.3727457	0.5022889	-1.0806951
H	-3.4984934	-1.3165303	-1.7992427
C	-3.8257668	1.1500982	1.1720058
H	-2.5092831	-0.1466596	2.2550233
C	-4.5262004	1.4215681	-0.0220192
H	-4.8947096	0.6760495	-2.0213703
H	-3.9148458	1.8365698	2.0135646
H	-5.1718590	2.2897121	-0.1152448
K	0.5714968	-1.5181553	-0.6137735
C	2.6074782	2.1834239	-0.3369422
H	3.0492714	2.7721259	-1.1527903
H	2.7064050	2.7812020	0.5766005
C	3.2338741	0.8416317	-0.2037298
C	3.6362015	0.1108690	-1.3396026
C	3.2663190	0.1816027	1.0408110
C	4.0736949	-1.2117974	-1.2339065
H	3.6134156	0.5928154	-2.3145013
C	3.7011055	-1.1409460	1.1511997
H	2.9507033	0.7190190	1.9328625
C	4.1087945	-1.8478776	0.0126150
H	4.3907522	-1.7478555	-2.1252336
H	3.7286779	-1.6201132	2.1267023
H	4.4534915	-2.8746041	0.0969749
K	-1.7484215	2.3799879	-0.9454954
C	1.0407955	2.1830912	-0.6795396
O	0.5647094	1.0720525	-1.0045760

Ba : dimer (**A**)$_2$ of carbene-like **A**

34

Energy = -1968.975436213

K	-1.7491812	-0.7005252	1.0166771
C	-2.2895695	2.2493032	-1.9761815
H	-2.1018300	3.3137147	-1.8031097
H	-2.5017621	2.1206895	-3.0469286
C	-3.3698374	1.7151605	-1.1122319
C	-3.6322357	2.2859404	0.1500619
C	-4.0175015	0.5015617	-1.4256332
C	-4.5162073	1.6878285	1.0494993
H	-3.1357159	3.2152767	0.4227647
C	-4.9047704	-0.0986606	-0.5284302
H	-3.8289783	0.0347148	-2.3891393
C	-5.1602119	0.4884574	0.7160063
H	-4.7097959	2.1595597	2.0098006
H	-5.3997744	-1.0278004	-0.8010678
H	-5.8538543	0.0246208	1.4116720
C	-0.8663004	1.5008090	-1.8011758
O	-0.9345394	0.3480427	-1.3297770
K	1.7362882	0.6492807	-1.0644469
C	2.2907536	-2.2462363	1.9797565
H	2.4887373	-2.0985120	3.0507482
H	2.1212696	-3.3159156	1.8206960
C	3.3724669	-1.7083717	1.1199071

C	3.9964786	-0.4794908	1.4214789
C	3.6591694	-2.2945112	-0.1299501
C	4.8844539	0.1211445	0.5252499
H	3.7887786	-0.0006576	2.3751114
C	4.5440087	-1.6961590	-1.0283740
H	3.1812030	-3.2360938	-0.3936167
C	5.1643021	-0.4812192	-0.7065763
H	5.3607647	1.0626075	0.7887649
H	4.7567179	-2.1796647	-1.9787260
H	5.8583676	-0.0168760	-1.4014838
C	0.8583722	-1.5222571	1.7780477
O	0.9144709	-0.3772400	1.2865937

B : ketene-like adduct of **A** and another CO
34
Energy = -1968.998116667

K	0.2492340	-0.0222855	1.2002205
C	-2.5371097	2.2078780	-0.2892404
H	-3.4229439	2.5703903	0.2444693
H	-2.5027965	2.7004019	-1.2713907
C	-2.5983086	0.7076978	-0.4624184
C	-3.2180962	-0.1004246	0.5038137
C	-1.9214320	0.0830651	-1.5214355
C	-3.1532468	-1.4931281	0.4215605
H	-3.7429359	0.3695951	1.3328677
C	-1.8456019	-1.3108453	-1.6013616
H	-1.4495674	0.6971423	-2.2841223
C	-2.4566468	-2.1029130	-0.6276191
H	-3.6427118	-2.1027155	1.1764313
H	-1.3039343	-1.7770646	-2.4195085
H	-2.3936090	-3.1856345	-0.6850892
C	-1.2671585	2.5910194	0.4732727
O	-0.0599477	2.2920709	-0.0266517
K	1.6133849	2.3446813	-1.8410680
C	3.1497016	-0.1987150	-1.7591459
H	2.8448493	-0.5574281	-2.7413518
H	4.0185769	0.4591255	-1.7287810
C	2.8011310	-0.9309615	-0.6140318
C	1.8450641	-2.0121819	-0.6337682
C	3.3437214	-0.6499068	0.6963583
C	1.5051143	-2.7319344	0.5036472
H	1.3896764	-2.2778348	-1.5864022
C	2.9899432	-1.3766500	1.8252096
H	4.0729479	0.1541980	0.7903535
C	2.0566626	-2.4309027	1.7659778
H	0.7828672	-3.5417380	0.4106907
H	3.4450623	-1.1175622	2.7802590
H	1.7881282	-3.0008461	2.6494015
C	-1.4096537	3.1247598	1.6889944
O	-1.4903356	3.7102773	2.7294296

CO : carbon monoxide
2
Energy = -113.3757872497

C	0.0329935	-0.0001741	0.0000015
O	1.1670416	0.0001728	-0.0000015

C : formal C-C coupling between two carbene **A**
34
Energy = -1969.047360498

K	-2.1164751	-1.8866105	0.7152623
C	-1.1725629	1.1787747	-1.0392974
H	-0.7304041	2.0831010	-0.6048902
H	-1.1628236	1.2376669	-2.1357594
C	-2.5797328	1.0221013	-0.5320410
C	-2.8987179	1.3882483	0.7894317
C	-3.5558618	0.3527905	-1.2928756
C	-4.1496538	1.0950409	1.3338613
H	-2.1318429	1.8708242	1.3912858
C	-4.8085439	0.0485819	-0.7438900
H	-3.3214919	0.0615064	-2.3131393
C	-5.1115023	0.4159395	0.5688400
H	-4.3779093	1.3894648	2.3553607
H	-5.5475857	-0.4764742	-1.3446713
H	-6.0844429	0.1831669	0.9931258
C	-0.3806133	-0.0634219	-0.5841717
O	-0.6330430	-1.1953953	-1.2344749
K	2.1177069	1.8838231	-0.7146685
C	1.1719729	-1.1812405	1.0364077
H	1.1607086	-1.2414219	2.1328071
H	0.7308195	-2.0852422	0.6003215
C	2.5797035	-1.0227727	0.5314072
C	3.5534543	-0.3516112	1.2937211
C	2.9016642	-1.3888785	-0.7893483
C	4.8067010	-0.0457624	0.7469206
H	3.3167329	-0.0602997	2.3134197
C	4.1531533	-1.0940171	-1.3316444
H	2.1367672	-1.8730785	-1.3924039
C	5.1125989	-0.4131666	-0.5651290
H	5.5438467	0.4805992	1.3488860
H	4.3836856	-1.3885321	-2.3526018

H	6.0859756	-0.1791215	-0.9877136
C	0.3798735	0.0608777	0.5813234
O	0.6316726	1.1929380	1.2319978

D : 5-ring from ketene/phenyl sites
34
Energy = -1968.984758105

K	0.8757817	-0.3256064	1.3886292
C	-1.9246109	1.9509637	-0.6527138
H	-2.6425162	2.7804417	-0.7084408
H	-1.3662365	1.8669310	-1.5876691
C	-2.4908845	0.6742775	-0.1317145
C	-3.0974052	0.9485216	1.2179068
C	-2.2496278	-0.6384039	-0.5763737
C	-3.6102554	-0.2664206	1.9047727
H	-3.7968781	1.7945650	1.2332296
C	-2.6499872	-1.7177709	0.1839894
H	-1.7462680	-0.7877572	-1.5288300
C	-3.3433678	-1.5092414	1.4286483
H	-4.1643666	-0.1380876	2.8305255
H	-2.4717759	-2.7296570	-0.1663259
H	-3.6933159	-2.3805723	1.9776401
C	-0.9672693	1.9576757	0.5424619
O	0.2653959	2.2629419	0.4946091
K	2.2570007	3.0910738	-0.8963101
C	3.4593185	0.6498844	-1.9636586
H	3.0357369	0.9296109	-2.9287116
H	4.4821890	0.9729558	-1.7722646
C	2.9584659	-0.4809162	-1.2990059
C	1.7215990	-1.1276726	-1.6699309
C	3.6251121	-1.0890896	-0.1710401
C	1.2596252	-2.2747625	-1.0393645
H	1.1515158	-0.7126786	-2.5002857
C	3.1496854	-2.2378036	0.4479434
H	4.5589745	-0.6477924	0.1751911
C	1.9561869	-2.8630462	0.0347456
H	0.3305939	-2.7250853	-1.3851426
H	3.7180253	-2.6605362	1.2755852
H	1.5963753	-3.7681170	0.5139373
C	-1.6344526	1.3495382	1.6898122
O	-1.0723349	0.9422644	2.7377259

E : aryl deprotonated 5-ring
19
Energy = -1697.344885076

K	1.9373810	1.0412259	-0.1781428
C	-1.4442023	0.2440004	1.9980667
H	-0.8005010	-0.3604501	2.6558275
H	-2.1093310	0.8191141	2.6608888
C	-2.2214300	-0.6143123	1.0266128
C	-1.8780772	-0.2286075	-0.2952007
C	-3.1346309	-1.6270051	1.2582000
C	-2.4722643	-0.8824859	-1.3859724
C	-3.7335356	-2.2857041	0.1595213
H	-3.3945646	-1.9189781	2.2749813
C	-3.3976904	-1.9083092	-1.1436115
H	-2.2129149	-0.5908039	-2.4012360
H	-4.4526897	-3.0830881	0.3291243
H	-3.8633742	-2.4206276	-1.9838900
C	-0.6289490	1.1531771	1.0884919
O	0.2048428	2.0814417	1.5292353
K	-1.0446112	3.6916129	-0.1637324
C	-0.9018500	0.8542692	-0.2603570
O	-0.3626525	1.4605390	-1.3245035

K^{+}·THF : THF-coordinated potassium ion
14
Energy = -832.4700869710

K	-0.4726443	0.8475956	-0.4420862
O	1.5180223	0.3831451	1.2169560
C	2.8576800	-0.0550714	0.8292799
C	1.4549483	0.5156699	2.6712963
C	3.5382936	-0.4914973	2.1226908
H	3.3758187	0.7925885	0.3642930
H	2.7482028	-0.8595569	0.0962108
C	2.9009150	0.4539689	3.1524311
H	0.8598411	-0.3162277	3.0678263
H	0.9543370	1.4609492	2.8998440
H	3.2889430	-1.5323818	2.3541701
H	4.6255129	-0.3970755	2.0682080
H	2.9784586	0.0865374	4.1785312
H	3.3643636	1.4447788	3.0998341

K^{+} : free potassium ion in solution
1
Energy = -599.8649338886

K	0.0000000	0.0000000	0.0000000

mA : carbene-like adduct of **1** KCH_2Ph and CO
17
Energy = -984.4691249464

K	-0.0285301	-0.4703589	-2.1020480
C	0.0364241	-1.0368622	1.5387397
H	-0.8549077	-1.3187169	2.1069219
H	0.9434768	-1.2909462	2.0948557
C	0.0118478	0.3398659	1.0456027
C	-1.2079057	0.9762869	0.7054371
C	1.2058314	1.0061992	0.6726700
C	-1.2312734	2.2109533	0.0551822
H	-2.1456041	0.4906802	0.9685452
C	1.1808800	2.2407907	0.0224871
H	2.1619726	0.5438874	0.9099550
C	-0.0374858	2.8578393	-0.2914036
H	-2.1864969	2.6752833	-0.1792681
H	2.1175794	2.7284802	-0.2378217
H	-0.0562083	3.8209642	-0.7930846
C	0.0475016	-2.2028976	0.2225919
O	0.0778910	-3.3361828	0.6669417

mB : ketene-like adduct of KCH_2Ph and two CO
19
Energy = -1097.883018497

K	-1.5358908	2.5003074	4.2037369
C	-0.1270493	-0.6177893	2.1889879
H	0.5927014	-1.3671250	2.5484340
H	-1.1021841	-0.8697680	2.6315755
C	-0.2075716	-0.6370714	0.6834388
C	0.9411857	-0.8708367	-0.0843015
C	-1.4053668	-0.3340840	0.0234320
C	0.8937765	-0.8107321	-1.4781239
C	-1.4590980	-0.2750344	-1.3706731
C	-0.3077033	-0.5123108	-2.1250274
H	1.7934223	-0.9969075	-2.0587803
H	-2.3975018	-0.0430213	-1.8675439
C	0.3208511	0.7449822	2.6976451
O	0.4592968	0.9893759	4.0098339
C	0.5228842	1.7280635	1.8032696
O	0.8211304	2.6649209	1.1165915
H	-2.3031456	-0.1477819	0.6091521
H	-0.3467229	-0.4664258	-3.2100224
H	1.8780514	-1.0993258	0.4185226

mD : 5-ring from ketene/phenyl sites
19
Energy = -1097.880127986

K	2.0249164	-1.3570465	-4.2601994
C	2.3463977	0.7761086	0.0529302
H	2.9976478	0.3921619	0.8538280
H	2.6224503	1.8059589	-0.1873754
C	0.8894995	0.5432856	0.2771695
C	0.6667524	-0.9421118	0.3950546
C	-0.1743803	1.4422181	0.1315413
C	-0.7721999	-1.3229011	0.4751617
C	-1.4838946	0.9988855	0.1649389
C	-1.7601822	-0.4016631	0.3292847
H	-1.0172829	-2.3722551	0.6186870
H	-2.3058241	1.7024002	0.0737614
C	2.2974842	-0.0764043	-1.2192866
O	2.9601511	0.1284045	-2.2770540
C	1.2703180	-1.1006473	-1.0563753
O	0.8206961	-1.8415917	-1.9713232
H	0.0458105	2.5002989	0.0010526
H	-2.7990287	-0.7240028	0.3662240
H	1.2872424	-1.4312017	1.1630441

mTS1 : transition state (TS) of CO insertion to **1**
17
Energy = -984.4690357492

K	0.0075047	-0.3572959	-1.8680988
C	-0.0025448	-1.1461444	1.6569311
H	-0.9136501	-1.4793930	2.1559554
H	0.9051578	-1.4767950	2.1639079
C	-0.0023717	0.2153658	1.1881646
C	-1.2109059	0.8889451	0.8461401
C	1.2069665	0.8911441	0.8534403
C	-1.2070890	2.1454741	0.2425037
H	-2.1601315	0.4103273	1.0800058
C	1.2044551	2.1476071	0.2495769
H	2.1556608	0.4143556	1.0930925
C	-0.0009543	2.7943521	-0.0642287
H	-2.1543115	2.6281798	0.0109975
H	2.1521736	2.6319352	0.0235338
H	-0.0004581	3.7751676	-0.5302737
C	0.0070337	-2.4097999	0.1643947
O	-0.0046125	-3.5207409	0.6038835

mTS2 : TS of second CO addition to **mA**
19
Energy = -1097.853005822

K	2.1599313	0.7438008	1.8063991
C	0.1805425	0.0048961	-1.8503299

H 0.1665432 0.7627254 -2.6438326
H 0.4650222 -0.9673362 -2.2680629
C -1.1465361 -0.0810062 -1.1525921
C -2.1065097 0.9327265 -1.2815960
C -1.4018283 -1.1309012 -0.2529611
C -3.3008338 0.8852577 -0.5609815
H -1.9155835 1.7621056 -1.9588877
C -2.5934091 -1.1781966 0.4720063
H -0.6620084 -1.9189358 -0.1361382
C -3.5504540 -0.1721569 0.3188059
H -4.0383786 1.6740295 -0.6865114
H -2.7771930 -2.0032958 1.1558182
H -4.4814188 -0.2113700 0.8779519
C 1.2365654 0.3578080 -0.7583172
O 1.7113854 1.5064302 -0.7709412
C 2.4762605 -1.5298035 -0.3852117
O 3.0115802 -1.5898885 0.6600341

mTS4 : TS of **mB** cyclization at phenyl/ketene
19
Energy = -1097.870358270
K -0.2464357 1.0813091 2.7433417
C -0.5857671 -2.0450722 -0.1580933
H -0.0966866 -2.9930205 -0.4294203
H -1.6080045 -2.2560635 0.1708994
C -0.5013681 -0.9926680 -1.2172194
C 0.8625346 -0.7162975 -1.5917461
C -1.5235022 -0.1063076 -1.5805549
C 1.1146989 0.4190965 -2.4328699
C -1.2402832 1.0148113 -2.3511807
C 0.0908539 1.2754153 -2.7659868
H 2.1250222 0.6066898 -2.7856484
H -2.0384094 1.6924104 -2.6407982
C 0.1431482 -1.2412923 0.9194973
O 0.0993189 -1.4743728 2.1855156
C 0.9695359 -0.2402126 0.3412465
O 1.4451967 0.8536809 0.6670581
H -2.5407664 -0.3029411 -1.2486487
H 0.2931255 2.1479035 -3.3825259
H 1.5685189 -1.5426812 -1.6119121

Ph_3SiCl : electrophilic Si reagent
35
Energy = -1445.235248852
Cl 0.0010985 0.0036361 2.7206818
Si 0.0009904 0.0010128 0.6192228
C 1.3982765 -1.1005356 0.0569885
C 2.3035712 -0.6626655 -0.9244335
C 1.5410068 -2.4054040 0.5635294
C 3.3209756 -1.5002653 -1.3860204
H 2.2140389 0.3423405 -1.3294461
C 2.5558952 -3.2438671 0.1054058
H 0.8554861 -2.7675761 1.3263910
C 3.4477787 -2.7912946 -0.8712438
H 4.0139028 -1.1450648 -2.1438615
H 2.6536106 -4.2481959 0.5083874
H 4.2400408 -3.4439111 -1.2276601
C -1.6512442 -0.6597137 0.0576097
C -2.8532052 -0.1453406 0.5772970
C -1.7233864 -1.6483811 -0.9383128
C -4.0862239 -0.6051630 0.1177515
H -2.8249079 0.6179734 1.3515633
C -2.9568844 -2.1106455 -1.4014406
H -0.8077250 -2.0622862 -1.3534893
C -4.1390060 -1.5893223 -0.8735704
H -5.0053660 -0.1990601 0.5311334
H -2.9947323 -2.8772869 -2.1705209
H -5.0998607 -1.9492882 -1.2310586
C 0.2549962 1.7609457 0.0536465
C -0.5739351 2.3196336 -0.9338627
C 1.3080855 2.5425340 0.5631254
C -0.3598204 3.6188076 -1.3985227
H -1.3953999 1.7352277 -1.3409669
C 1.5245352 3.8398049 0.1014902
H 1.9612906 2.1353760 1.3316513
C 0.6895865 4.3796110 -0.8811199
H -1.0121931 4.0367262 -2.1603762
H 2.3414403 4.4308436 0.5064356
H 0.8569103 5.3913524 -1.2402710

$PhCH_2CO^-$: unstable adduct of $PhCH_2^-$ and CO
16
Energy = -384.5579628805
C 0.0414235 -0.9194537 1.6740330
H -0.8681599 -1.3008957 2.1347452
H 0.9726832 -1.2736293 2.1127560
C 0.0151679 0.3607607 1.0713819
C -1.2073965 1.0128112 0.7110959
C 1.2086652 1.0477522 0.6799036
C -1.2283998 2.2345093 0.0508429

H	-2.1485688	0.5305349	0.9734862
C	1.1772343	2.2694816	0.0200629
H	2.1698537	0.5931003	0.9178845
C	-0.0392405	2.8929603	-0.3051797
H	-2.1885076	2.6892468	-0.1916074
H	2.1172062	2.7517591	-0.2468337
H	-0.0596905	3.8511148	-0.8173713
C	0.0486309	-2.4999256	0.0816071
O	0.0526215	-3.5345019	0.6415453

THF : tetrahydrofuran as solvent
13
Energy = -232.5925785907

O	-0.0038131	0.0019480	-1.2563628
C	0.1578892	1.1738424	-0.4157921
C	-0.1577712	-1.1728332	-0.4179848
C	0.2219345	-0.7356644	0.9965392
C	-0.2223784	0.7349873	0.9978266
H	1.2057320	1.5021622	-0.4634859
H	-0.4810388	1.9681663	-0.8144858
H	-1.2038612	-1.5063743	-0.4657976
H	0.4849607	-1.9630810	-0.8187877
H	-0.2704697	-1.3371542	1.7654988
H	1.3059319	-0.8052057	1.1406490
H	-1.3064368	0.8046308	1.1415113
H	0.2699774	1.3350333	1.7679621

TS1 : TS of first CO addition to dimer (**1**)$_2$
32
Energy = -1855.572343472

C	-1.3079059	-2.0537569	-1.1975994
H	-1.4304880	-2.2022982	-2.2703647
H	-0.7912603	-2.8459829	-0.6544102
C	-2.2244266	-1.2467282	-0.5079199
C	-3.1670207	-0.3786219	-1.1756910
C	-2.2985762	-1.1970523	0.9342505
C	-4.1121377	0.3704161	-0.4860991
H	-3.1591560	-0.3498236	-2.2644345
C	-3.2454070	-0.4381036	1.6084239
H	-1.6018139	-1.8073923	1.5062517
C	-4.1796547	0.3655190	0.9220060
H	-4.8147162	0.9787219	-1.0546567
H	-3.2619817	-0.4679602	2.6972711
H	-4.9315195	0.9394617	1.4546965
K	1.4749651	-1.2210730	-1.5926594
C	1.6424057	2.1342769	1.0684555
H	2.3291749	2.9505831	0.8481318
H	0.8460556	2.4157584	1.7603186
C	2.1929884	0.8261585	1.2134154
C	3.4287729	0.4635305	0.5953072
C	1.4763404	-0.2369216	1.8439040
C	3.9080156	-0.8465846	0.6255697
H	4.0182841	1.2386907	0.1118884
C	1.9565344	-1.5407547	1.8621166
H	0.5248387	-0.0159970	2.3256504
C	3.1814741	-1.8699336	1.2520380
H	4.8639352	-1.0730134	0.1567712
H	1.3750046	-2.3153496	2.3575081
H	3.5619997	-2.8867064	1.2811827
K	-1.6571358	2.0009634	0.4119030
C	0.8414330	2.1407118	-0.9493309
O	1.6394576	1.6194593	-1.6491129

TS2a : TS of 2nd CO addition, carbene dimer
34
Energy = -1968.963734996

K	-1.6402423	-0.2121361	1.6383233
C	-2.6943909	0.2864683	-2.3880398
H	-2.8321585	1.2554516	-2.8795932
H	-2.7839027	-0.4869159	-3.1636477
C	-3.6469485	0.0800233	-1.2684212
C	-4.1249513	1.1728987	-0.5164188
C	-3.9435427	-1.2153623	-0.7955446
C	-4.8835364	0.9834055	0.6405239
H	-3.9011725	2.1836838	-0.8518894
C	-4.7048273	-1.4091490	0.3603645
H	-3.5824359	-2.0772179	-1.3517673
C	-5.1794826	-0.3114523	1.0879853
H	-5.2513644	1.8455606	1.1914047
H	-4.9305543	-2.4192521	0.6937256
H	-5.7761199	-0.4609473	1.9833921
C	-1.1360060	0.2160234	-1.9854140
O	-0.8938575	-0.3183444	-0.8817177
K	1.6262021	0.5495764	-1.4924717
C	3.2750030	-0.1603846	2.6008194
H	3.4534657	0.6772926	3.2715637
H	3.0879559	-1.1182039	3.0785881
C	3.8364921	-0.1327936	1.3027791
C	4.2578568	1.0899643	0.6878090
C	3.9043330	-1.2976089	0.4707284
C	4.7449055	1.1293201	-0.6166495

H	4.2206322	2.0063600	1.2731741
C	4.3873430	-1.2466821	-0.8313385
H	3.5844948	-2.2519718	0.8858475
C	4.8199128	-0.0332778	-1.4015604
H	5.0742257	2.0807625	-1.0309661
H	4.4368444	-2.1636832	-1.4159041
H	5.2151602	-0.0010990	-2.4126305
C	1.1480863	0.4440687	2.2129860
O	1.1132463	1.2276438	1.3292522

TS2 : TS of 2nd CO addition, ketene-like

34

Energy = -1968.965188578

C	-1.6489731	-2.9163082	0.5684976
H	-1.6154265	-3.7117122	-0.1764598
H	-1.2405587	-3.1562251	1.5501146
C	-2.6274253	-1.9140817	0.4577220
C	-3.4064108	-1.7061989	-0.7408278
C	-2.9276491	-0.9875398	1.5243322
C	-4.4043822	-0.7443629	-0.8260496
H	-3.2230275	-2.3580113	-1.5945247
C	-3.9311372	-0.0330727	1.4239324
H	-2.3656571	-1.0712254	2.4537377
C	-4.6994203	0.1162535	0.2509611
H	-4.9742384	-0.6606499	-1.7509169
H	-4.1258494	0.6144633	2.2781613
H	-5.4980086	0.8492449	0.1875069
K	0.8663732	-1.8429526	-0.4305668
C	2.5429432	2.0475078	-0.3459899
H	2.8839512	2.5016352	-1.2866480
H	2.6826631	2.7960347	0.4419818
C	3.2596876	0.7708332	-0.0603889
C	3.8043839	-0.0091663	-1.0971364
C	3.2736325	0.2383452	1.2434821
C	4.3574306	-1.2668502	-0.8398107
H	3.8018481	0.3803794	-2.1127414
C	3.8210144	-1.0193982	1.5029406
H	2.8529847	0.8214217	2.0601138
C	4.3663844	-1.7811853	0.4615947
H	4.7842170	-1.8449987	-1.6554233
H	3.8284182	-1.4048332	2.5191728
H	4.7970329	-2.7576554	0.6635003
K	-2.1368132	1.5459032	-0.6604176
C	0.9991922	1.8961434	-0.5252692
O	0.5079221	0.7916241	-0.7907343
C	-0.1414658	3.2708971	0.4684017
O	0.6094460	3.9639440	1.0722210

TS3a : TS of C-C coupling of carbene dimer

34

Energy = -1968.960168645

K	-2.0925358	-1.5025550	0.9489178
C	-1.3587295	1.3131990	-1.3765583
H	-0.7915406	2.1444819	-0.9472589
H	-1.4424969	1.4509012	-2.4621057
C	-2.6598383	1.1388003	-0.7166379
C	-2.8822058	1.6261155	0.5928938
C	-3.6607217	0.3104960	-1.2805642
C	-4.0490353	1.3283179	1.2926086
H	-2.1120371	2.2364939	1.0584972
C	-4.8251107	-0.0013006	-0.5704957
H	-3.5227632	-0.0762095	-2.2860720
C	-5.0313336	0.5022347	0.7182197
H	-4.1966775	1.7332882	2.2911214
H	-5.5782600	-0.6382704	-1.0292081
H	-5.9411071	0.2669032	1.2635048
C	-0.3640894	-0.0511309	-1.3074793
O	-0.9685221	-1.1178486	-1.4524288
K	2.0911874	1.4858439	-0.9779628
C	1.3465320	-1.3172890	1.3537393
H	1.4147797	-1.4571340	2.4400929
H	0.7870220	-2.1483893	0.9139394
C	2.6564504	-1.1389501	0.7128922
C	3.6447129	-0.3039667	1.2893382
C	2.9014200	-1.6294423	-0.5914541
C	4.8183088	0.0108952	0.5961817
H	3.4895793	0.0852859	2.2913220
C	4.0773540	-1.3281863	-1.2743390
H	2.1422284	-2.2463944	-1.0664498
C	5.0467598	-0.4956873	-0.6875998
H	5.5614122	0.6527727	1.0643409
H	4.2424897	-1.7361523	-2.2688857
H	5.9637514	-0.2578078	-1.2195233
C	0.3489015	0.0451519	1.2741219
O	0.9486523	1.1140542	1.4192791

TS3 : TS of ketene/benzyl anion addition

34

Energy = -1968.979107240

C	-2.9573155	0.8258899	-0.1220281
H	-3.3376901	0.5753803	-1.1130410
H	-3.5665116	0.5370772	0.7350822

C	-2.1282005	1.9568044	-0.0125230
C	-1.5639866	2.6049530	-1.1685257
C	-1.7189342	2.5162703	1.2488329
C	-0.6595198	3.6540367	-1.0664874
H	-1.8457536	2.2360447	-2.1529926
C	-0.8108650	3.5635731	1.3344542
H	-2.1206256	2.0816231	2.1627099
C	-0.2457032	4.1485373	0.1846745
H	-0.2602306	4.0961032	-1.9781920
H	-0.5288972	3.9353066	2.3185103
H	0.4598106	4.9700909	0.2606759
K	-3.3995453	-2.1249136	-0.2254446
C	0.8374785	-2.6978187	-0.0898770
H	0.6446107	-3.2932164	0.8089847
H	0.8179183	-3.3561286	-0.9673754
C	2.1495177	-1.9732544	0.0160316
C	2.6818753	-1.6309351	1.2702744
C	2.7917472	-1.4939360	-1.1386638
C	3.8141751	-0.8219990	1.3720791
H	2.1895287	-1.9903859	2.1713409
C	3.9203375	-0.6730333	-1.0375328
H	2.3910632	-1.7545288	-2.1135435
C	4.4323086	-0.3296732	0.2155943
H	4.2131987	-0.5713523	2.3513801
H	4.3985886	-0.3010777	-1.9400203
H	5.3088111	0.3074102	0.2933492
K	0.7734832	1.0554831	0.0096185
C	-0.3111707	-1.6561440	-0.3250553
O	-0.2709919	-0.9492658	-1.4049772
C	-0.9066491	-1.3721372	0.9002814
O	-1.6245966	-1.7283576	1.8234079

TS4 : TS of phenyl/ketene cyclization, 5-ring
34
Energy = -1968.978437455

K	0.4770590	-0.6609018	1.6049156
C	-1.4869529	1.6890721	-1.1295070
H	-1.9470131	2.6590973	-1.3719344
H	-0.6387880	1.5190138	-1.8001365
C	-2.4501321	0.5455598	-1.0882337
C	-3.5206245	0.7764263	-0.1489555
C	-2.2192752	-0.7414531	-1.5932945
C	-4.3831013	-0.3264795	0.1715949
H	-3.9136285	1.7856328	-0.0561128
C	-3.0406922	-1.7962955	-1.2218004
H	-1.3843208	-0.9061317	-2.2696170
C	-4.1159733	-1.5816271	-0.3206548
H	-5.2328216	-0.1614362	0.8271828
H	-2.8619294	-2.7906860	-1.6204582
H	-4.7575239	-2.4169700	-0.0532876
C	-1.0348421	1.5362077	0.3207374
O	0.0729825	1.9837883	0.8167090
K	2.3397714	2.9401543	0.1750317
C	3.7629362	0.6032121	-0.9037253
H	3.5711617	1.0259588	-1.8901518
H	4.7193242	0.8545017	-0.4465220
C	3.1081235	-0.5794450	-0.5266630
C	1.9749845	-1.1151832	-1.2445947
C	3.4959923	-1.3600304	0.6253813
C	1.3647680	-2.3111757	-0.8932825
H	1.6111032	-0.5695522	-2.1143863
C	2.8749078	-2.5554578	0.9625042
H	4.3358140	-1.0111298	1.2249124
C	1.7932659	-3.0653425	0.2170622
H	0.5287779	-2.6693199	-1.4918421
H	3.2367716	-3.1077564	1.8288571
H	1.3210287	-4.0074699	0.4768426
C	-2.0114355	0.8791540	1.1046472
O	-2.0395857	0.2172988	2.1459529

TS5 : TS of 5-ring deprotonation
34
Energy = -1968.981477517

K	1.8621476	1.3709352	-0.1555594
C	-1.4369991	0.0067520	2.0130906
H	-0.6938305	-0.6612327	2.4802384
H	-2.0287402	0.4311685	2.8363947
C	-2.2814384	-0.7460849	1.0259033
C	-1.8120945	-0.3998535	-0.3227154
C	-3.3501985	-1.5793244	1.2128388
C	-2.6774255	-0.7712161	-1.4316314
H	-0.8368729	-1.0663565	-0.4522990
C	-4.0704243	-2.0692302	0.0891193
H	-3.6809769	-1.8356194	2.2180713
C	-3.7329907	-1.6406500	-1.2056801
H	-2.4199773	-0.4418014	-2.4343798
H	-4.9143322	-2.7372146	0.2373052
H	-4.3356536	-1.9787191	-2.0466909
C	-0.7224263	1.0671997	1.1815419
O	-0.0256584	2.0575097	1.6805152
K	-1.4601923	3.6880548	0.1668337

C	0.6440394	-2.7149027	-0.4300170
H	0.1053208	-3.0503992	0.4511588
H	0.2929900	-3.0678523	-1.3949550
C	1.9030608	-2.0966451	-0.3034487
C	2.4418660	-1.7162200	0.9723468
C	2.7272648	-1.7847918	-1.4372480
C	3.6829124	-1.1018437	1.0955033
H	1.8552238	-1.9261502	1.8653210
C	3.9614768	-1.1616814	-1.3010688
H	2.3671016	-2.0531070	-2.4287913
C	4.4651727	-0.8043771	-0.0357755
H	4.0502737	-0.8428199	2.0870053
H	4.5485595	-0.9495703	-2.1925618
H	5.4349430	-0.3258332	0.0653181
C	-1.0109985	0.8866977	-0.1919112
O	-0.5814369	1.6089354	-1.1903405

Table S3. TPSS-D3/def2-TZVP + COSMO optimized Cartesian coordinates (in Å) in THF solution for the reactions of benzyl potassium **4** KBzt with CO. Each structure is labeled by the specific name (See also **Figure S2** and **Table S1**), followed by the number of atoms, the total energy (in hartrees), and the detailed atomic coordinates (in double-column text list).

4.CO : loose adduct of CO and monomeric KBzt
53
Energy = -1456.505675828

K	0.0553148	2.1834165	1.0324871
C	0.0935760	-0.7158601	2.3084382
H	1.0158101	-1.0009766	2.7920382
H	-0.7837795	-0.9830637	2.8779939
C	0.0266536	-0.6615580	0.8850911
C	1.2293339	-0.5715312	0.0631484
C	-1.2404625	-0.5520364	0.1764919
C	1.1180827	-0.3336259	-1.3056775
C	-1.2549284	-0.3153498	-1.2019328
C	-0.1016016	-0.1828109	-1.9784687
H	2.0277815	-0.2652822	-1.8915928
H	-2.2138580	-0.2343949	-1.6962059
C	2.6463004	-0.7444164	0.6556242
C	2.9752723	0.3863661	1.6640542
C	2.8032138	-2.1473958	1.2931321
C	3.7544618	-0.6609335	-0.4156169
H	3.0722822	1.3389012	1.1254160
H	2.2145602	0.5015090	2.4387802
H	3.9361036	0.1910044	2.1551070
H	2.7494627	-2.9070103	0.5052709
H	3.7817062	-2.2302529	1.7821785
H	2.0280441	-2.3729705	2.0262288
H	4.7240293	-0.8057660	0.0747308
H	3.6442474	-1.4388494	-1.1786982
H	3.7744386	0.3158994	-0.9117956
C	-2.5997229	-0.7035733	0.8964763
C	-2.7182766	-2.1032089	1.5497852
C	-2.8186912	0.4326333	1.9283543
C	-3.8006982	-0.6051183	-0.0681346
H	-2.7514583	-2.8649426	0.7627582
H	-1.8803106	-2.3402037	2.2062542
H	-3.6469193	-2.1701622	2.1301207
H	-2.9523520	1.3857956	1.3988513
H	-3.7321364	0.2510155	2.5075126
H	-1.9878056	0.5380879	2.6288302
H	-4.7229373	-0.7361513	0.5097090
H	-3.8528156	0.3715914	-0.5622389
H	-3.7721226	-1.3846504	-0.8369283
C	-0.1219733	0.0957072	-3.4838749
C	-1.5527658	0.2123604	-4.0339547
C	0.6173201	1.4215456	-3.7756966
C	0.5930884	-1.0490242	-4.2370426
H	-2.1169106	-0.7150242	-3.8838142
H	-2.1021108	1.0299577	-3.5532520
H	-1.5174565	0.4158253	-5.1103170
H	1.6514933	1.3822128	-3.4174445
H	0.6368602	1.6249857	-4.8538337
H	0.1154021	2.2584131	-3.2758167
H	0.5964049	-0.8563061	-5.3173313
H	1.6321436	-1.1510190	-3.9066834
H	0.0849860	-2.0022265	-4.0547572
C	0.1756649	1.2970512	3.7116885
O	0.2203937	0.8661020	4.7896149

(**4**)$_2$: K^+-bound dimer of **4** KBzt
102
Energy = -2686.253190489

C	-1.9900886	-1.5215243	-0.0603241
H	-2.5713704	-1.2607927	0.8174173
H	-2.5005133	-1.2782230	-0.9853092
C	-1.1516757	-2.6687266	-0.0160789
C	-0.6846604	-3.2517622	1.2368376
C	-0.6058183	-3.2843136	-1.2247384
C	0.4161979	-4.1111636	1.2388805
C	0.4878083	-4.1412364	-1.1305920
C	1.0814215	-4.5301937	0.0806467
H	0.7704835	-4.4835729	2.1914418
H	0.9022854	-4.5470389	-2.0473700
K	-1.4309112	1.4061980	0.0274081
C	1.9900398	1.5221096	-0.0620995
H	2.5717323	1.2599448	0.8149769
H	2.4984905	1.2771129	-0.9875863
C	1.1514658	2.6688374	-0.0169018
C	0.6840723	3.2508760	1.2363227
C	0.6060392	3.2852326	-1.2254856
C	-0.4159230	4.1114362	1.2383245
C	-0.4873076	4.1423388	-1.1312986
C	-1.0804582	4.5315003	0.0800849

H	-0.7701278	4.4838287	2.1909356
H	-0.9014917	4.5487382	-2.0479185
K	1.4318209	-1.4069502	0.0302493
C	-1.3565745	-2.9319456	2.5894200
C	-1.0909075	-1.4693274	3.0176400
C	-2.8713347	-3.2452501	2.5229924
C	-0.8134662	-3.8019940	3.7425361
H	-0.0273653	-1.3421291	3.2526635
H	-1.3489460	-0.7533523	2.2355001
H	-1.6631333	-1.2187209	3.9192509
H	-3.0111040	-4.3261364	2.4097548
H	-3.3612356	-2.9321468	3.4534162
H	-3.3673099	-2.7554965	1.6836854
H	-1.3813398	-3.5729485	4.6518549
H	-0.9276174	-4.8706762	3.5305302
H	0.2424774	-3.5990404	3.9530433
C	-1.1929087	-3.0037871	-2.6244392
C	-2.7085633	-3.3192137	-2.6416170
C	-0.9018853	-1.5525175	-3.0738549
C	-0.5783881	-3.9040925	-3.7166528
H	-2.8542772	-4.3961965	-2.5019817
H	-3.2554584	-2.8031769	-1.8511285
H	-3.1394815	-3.0378325	-3.6104748
H	0.1727844	-1.4330852	-3.2586305
H	-1.4261209	-1.3240820	-4.0098151
H	-1.1954274	-0.8152011	-2.3251813
H	-1.0857643	-3.6982707	-4.6663224
H	0.4893800	-3.7067329	-3.8637688
H	-0.7082656	-4.9669898	-3.4853025
C	2.3047762	-5.4492024	0.0870347
C	3.4582378	-4.7848775	-0.6981515
C	2.8017318	-5.7391267	1.5124287
C	1.9555344	-6.7949267	-0.5883845
H	3.1543771	-4.5490750	-1.7236864
H	3.7697610	-3.8524807	-0.2099495
H	4.3308197	-5.4483250	-0.7459731
H	2.0458643	-6.2699106	2.1016744
H	3.6980947	-6.3683355	1.4700015
H	3.0613658	-4.8143884	2.0411993
H	2.8278399	-7.4606345	-0.6006104
H	1.1445829	-7.2934140	-0.0463282
H	1.6288619	-6.6430642	-1.6225568
C	1.3552483	2.9298730	2.5889241
C	1.0905937	1.4665554	3.0153292
C	2.8698225	3.2442663	2.5234324
C	0.8107065	3.7978083	3.7429034
H	0.0270968	1.3382552	3.2499299
H	1.3494109	0.7515582	2.2325896
H	1.6627443	1.2155103	3.9168754
H	3.0090112	4.3253377	2.4112235
H	3.3596656	2.9304846	3.4536673
H	3.3660827	2.7554240	1.6837328
H	1.3775198	3.5671223	4.6524768
H	0.9251005	4.8669009	3.5330813
H	-0.2455087	3.5944459	3.9516729
C	1.1934734	3.0051530	-2.6251636
C	2.7096096	3.3183793	-2.6419388
C	0.9001443	1.5547490	-3.0757692
C	0.5808892	3.9076767	-3.7166771
H	2.8567898	4.3950602	-2.5014540
H	3.2560506	2.8011073	-1.8520022
H	3.1399698	3.0373051	-3.6111440
H	-0.1746382	1.4373248	-3.2611514
H	1.4244657	1.3257328	-4.0115423
H	1.1921091	0.8166155	-2.3272367
H	1.0888915	3.7027389	-4.6661985
H	-0.4869414	3.7116410	-3.8650876
H	0.7116865	4.9700449	-3.4834621
C	-2.3038634	5.4504124	0.0862595
C	-3.4582180	4.7831385	-0.6951923
C	-2.7984257	5.7437727	1.5117722
C	-1.9565458	6.7946334	-0.5931190
H	-3.1563610	4.5469947	-1.7212617
H	-3.7656307	3.8504114	-0.2050509
H	-4.3325259	5.4443735	-0.7414193
H	-2.0411582	6.2751428	2.0986963
H	-3.6943629	6.3735999	1.4693226
H	-3.0580832	4.8204251	2.0429740
H	-2.8293743	7.4596745	-0.6056685
H	-1.1452301	7.2951401	-0.0534915
H	-1.6313401	6.6406587	-1.6274256

4H : arene by-product BztH
51
Energy = -743.7664316287

H	0.9809760	-0.1942865	2.0895034
C	1.2687507	-1.2462911	2.2002062
H	2.1983963	-1.2680844	2.7641784
H	0.5110897	-1.7245686	2.8164922
C	1.4100106	-1.9229699	0.8511595
C	2.6613881	-1.9124311	0.1700401
C	0.2931179	-2.5572715	0.2447120

C	2.7525510	-2.5456260	-1.0724360
C	0.4612681	-3.1731438	-1.0042636
C	1.6746479	-3.1860981	-1.6814585
H	3.7004656	-2.5434593	-1.5948539
H	-0.3864418	-3.6587863	-1.4645227
C	3.9498019	-1.2530628	0.7304989
C	3.7359737	0.2343361	1.1021928
C	4.4980349	-2.0594400	1.9337501
C	5.0924461	-1.2440202	-0.3111352
H	3.3260702	0.7800235	0.2448901
H	3.0679227	0.3830296	1.9494126
H	4.7027658	0.6828586	1.3592915
H	4.7371151	-3.0800221	1.6155565
H	5.4182503	-1.5910696	2.3032365
H	3.7983468	-2.1272557	2.7693411
H	5.9559097	-0.7334080	0.1284031
H	5.4118432	-2.2546236	-0.5855200
H	4.8089432	-0.7059563	-1.2222217
C	-1.1179671	-2.6324994	0.8875283
C	-1.1125322	-3.5871125	2.1070927
C	-1.6667386	-1.2364267	1.2697991
C	-2.1658135	-3.2189557	-0.0868923
H	-0.8181323	-4.5924834	1.7870565
H	-0.4290280	-3.2774342	2.9003490
H	-2.1204979	-3.6451167	2.5355049
H	-1.6426155	-0.5725599	0.3983349
H	-2.7101558	-1.3365775	1.5911335
H	-1.1176761	-0.7549209	2.0777347
H	-3.1422700	-3.2075310	0.4090488
H	-2.2452640	-2.6260670	-1.0044874
H	-1.9462127	-4.2567114	-0.3578448
C	1.8603442	-3.8598485	-3.0453004
C	0.5664696	-4.5147440	-3.5551555
C	2.3118394	-2.8045689	-4.0794363
C	2.9464304	-4.9526907	-2.9317703
H	0.2125364	-5.2929279	-2.8697052
H	-0.2340528	-3.7777547	-3.6839841
H	0.7538338	-4.9823234	-4.5281194
H	3.2535414	-2.3332237	-3.7799996
H	2.4613838	-3.2739962	-5.0591249
H	1.5556954	-2.0185735	-4.1827926
H	3.1004933	-5.4360734	-3.9039192
H	3.9022661	-4.5298333	-2.6059943
H	2.6485236	-5.7179979	-2.2066979

4^{-} : bulky benzyl anion

50

Energy = -743.1999548920

C	1.3117123	-1.3984666	2.2327460
H	2.1423824	-0.9228232	2.7322768
H	0.3871785	-1.4000392	2.7904977
C	1.4246270	-1.9703788	0.9523674
C	2.6817324	-1.9664521	0.1955347
C	0.3003075	-2.6151589	0.2703885
C	2.7462941	-2.5466135	-1.0638710
C	0.4615827	-3.1716413	-0.9962640
C	1.6652796	-3.1648697	-1.7076722
H	3.6957709	-2.5229045	-1.5907997
H	-0.3993832	-3.6389316	-1.4595760
C	3.9652036	-1.3262768	0.7620618
C	3.7613999	0.1920961	0.9922970
C	4.4123306	-2.0530607	2.0550275
C	5.1713515	-1.4293982	-0.1945146
H	3.6819653	0.6924188	0.0195642
H	2.8474567	0.4014053	1.5514907
H	4.6183358	0.6217560	1.5296866
H	4.7663929	-3.0578540	1.7946294
H	5.2385496	-1.5128147	2.5378485
H	3.5921354	-2.1621405	2.7672419
H	6.0384205	-0.9522684	0.2803634
H	5.4350397	-2.4715965	-0.4084873
H	4.9830612	-0.9176145	-1.1453015
C	-1.0941000	-2.7025794	0.9224965
C	-1.0347420	-3.5348276	2.2279319
C	-1.6726857	-1.2857444	1.1644073
C	-2.1378631	-3.4167437	0.0384076
H	-0.8450514	-4.5840722	1.9714994
H	-0.2321442	-3.2025401	2.8892310
H	-1.9906037	-3.4812643	2.7678053
H	-1.9119777	-0.8299727	0.1960473
H	-2.5971220	-1.3383947	1.7564463
H	-0.9594078	-0.6340649	1.6728674
H	-3.0971789	-3.4351250	0.5717456
H	-2.2912921	-2.8961950	-0.9139339
H	-1.8515917	-4.4529009	-0.1760935
C	1.8399295	-3.7761630	-3.0972778
C	0.5397641	-4.4056343	-3.6233202
C	2.2844201	-2.6889889	-4.1034386
C	2.9223406	-4.8800109	-3.0594036
H	0.1909192	-5.2081877	-2.9633044
H	-0.2599147	-3.6602134	-3.7024100
H	0.7079038	-4.8324810	-4.6201257

H	3.2225799	-2.2235332	-3.7823641
H	2.4368893	-3.1137299	-5.1059762
H	1.5247233	-1.9017878	-4.1686991
H	3.0782121	-5.3180129	-4.0556668
H	3.8780171	-4.4744105	-2.7102200
H	2.6243500	-5.6774480	-2.3692252

4 : *t*Bu-substituted benzyl potassium KBzt
51
Energy = -1343.114693950

K	0.8155824	-0.0717769	-1.2189109
C	1.2012956	-1.0304407	2.0080548
H	2.0269751	-0.5414612	2.5032983
H	0.2762696	-1.0311563	2.5653874
C	1.3929362	-1.8587839	0.8927452
C	2.6821027	-1.9501376	0.1892275
C	0.3007706	-2.6204285	0.2726938
C	2.7364183	-2.4954274	-1.0880484
C	0.4531885	-3.1408946	-1.0114326
C	1.6324530	-3.0445487	-1.7654191
H	3.6945090	-2.5082337	-1.5977906
H	-0.3910178	-3.6541723	-1.4546931
C	3.9838734	-1.3945019	0.8024830
C	3.9570948	0.1554256	0.8453838
C	4.2291423	-2.0046424	2.2036385
C	5.2338264	-1.7625475	-0.0239417
H	3.9998573	0.5471969	-0.1799931
H	3.0579176	0.5501972	1.3234751
H	4.8326217	0.5412551	1.3826804
H	4.4418872	-3.0745541	2.0971756
H	5.0977282	-1.5285006	2.6758144
H	3.3665394	-1.8999003	2.8632090
H	6.1235972	-1.3994759	0.5040948
H	5.3302245	-2.8469952	-0.1477717
H	5.2265236	-1.2980660	-1.0164073
C	-1.0576574	-2.8107100	0.9787199
C	-0.8584354	-3.4336800	2.3816095
C	-1.8394015	-1.4731043	1.0494965
C	-1.9846337	-3.7901207	0.2287587
H	-0.4916973	-4.4604576	2.2694799
H	-0.1338377	-2.8844106	2.9845261
H	-1.8144260	-3.4685722	2.9193686
H	-2.1474858	-1.1789278	0.0370145
H	-2.7500130	-1.5897855	1.6507440
H	-1.2483020	-0.6591605	1.4752042
H	-2.8967852	-3.9337358	0.8201891
H	-2.2845743	-3.4078781	-0.7534903
H	-1.5102827	-4.7681785	0.0910016
C	1.7952641	-3.6289811	-3.1696774
C	0.4623283	-4.1401234	-3.7401109
C	2.3432503	-2.5501711	-4.1310098
C	2.7918792	-4.8110274	-3.1354736
H	0.0577629	-4.9636778	-3.1414584
H	-0.2884162	-3.3416775	-3.7747302
H	0.6138234	-4.5096713	-4.7610605
H	3.2911134	-2.1400171	-3.7664802
H	2.5158845	-2.9684160	-5.1311088
H	1.6286726	-1.7226588	-4.2273647
H	2.9291521	-5.2374061	-4.1378762
H	3.7705166	-4.4865663	-2.7657966
H	2.4230676	-5.5987441	-2.4693763

5p : product **5** with $SiPh_3$ instead of $SiMe_3$
172
Energy = -3683.105840064

C	0.9074992	-2.3731351	-0.5073845
H	0.5576254	-3.2925152	-0.0350827
H	0.6290355	-2.4723603	-1.5542267
C	2.4047690	-2.2409796	-0.3456779
C	3.0388702	-2.7271139	0.8304999
C	3.1736937	-1.5849072	-1.3395182
C	4.3760829	-2.3846589	1.0515490
C	4.5059967	-1.2662189	-1.0454033
C	5.1170355	-1.6143903	0.1538045
H	4.8649655	-2.7223123	1.9565046
H	5.0864801	-0.7318375	-1.7829117
C	0.1284784	-1.2009964	0.0649514
O	0.7359280	-0.4892553	1.0841601
C	-1.9044092	0.2736000	0.2511673
H	-1.6520346	0.3607554	1.3059996
H	-1.5203622	1.1878881	-0.2040671
C	-3.3993592	0.1756736	0.0487903
C	-4.2010578	-0.5097910	1.0033336
C	-4.0006436	0.7131227	-1.1161946
C	-5.5206288	-0.8110883	0.6593264
C	-5.3437693	0.4053030	-1.3778685
C	-6.1044506	-0.4023056	-0.5398992
H	-6.1275619	-1.3758469	1.3556268
H	-5.8027881	0.7871329	-2.2779360
C	-1.1326167	-0.9089399	-0.3113482
O	-1.7430794	-1.6223964	-1.3267566
C	2.3813589	-3.7607166	1.7886711

C	3.3304617	-4.1595546	2.9400522	H	-1.5990280	-1.2472790	2.2176049
C	1.0730186	-3.3187433	2.4891573	H	-2.3254118	-1.7529933	3.7399073
C	2.1409415	-5.0621613	0.9788095	H	-3.2654519	0.5174611	4.1667924
H	4.2580840	-4.6097786	2.5727538	H	-2.7744129	1.2195928	2.6216421
H	3.5849803	-3.3032431	3.5736055	H	-4.4838330	1.2062791	3.0697864
H	2.8238427	-4.9012435	3.5671589	C	-3.2950808	1.7325474	-2.0534552
H	0.2821495	-2.9864062	1.8213101	C	-4.2137378	2.1816494	-3.2112556
H	0.6876774	-4.1678871	3.0658739	C	-3.0014792	3.0174806	-1.2364084
H	1.2604622	-2.5045148	3.1939798	C	-2.0085310	1.2209720	-2.7456738
H	1.6852282	-5.8244575	1.6226962	H	-4.4852814	1.3456751	-3.8651593
H	1.4935905	-4.9135934	0.1112326	H	-5.1318572	2.6559174	-2.8503913
H	3.0967893	-5.4482973	0.6092562	H	-3.6731308	2.9168274	-3.8173240
C	2.6887500	-1.3685266	-2.7969935	H	-2.3827638	2.8351826	-0.3548628
C	3.7715071	-0.6986209	-3.6711097	H	-2.4926054	3.7553994	-1.8688378
C	2.4573753	-2.7678115	-3.4147880	H	-3.9429804	3.4543005	-0.8865232
C	1.4362912	-0.4799102	-2.9598698	H	-1.5921346	2.0343063	-3.3517964
H	4.0192191	0.3050991	-3.3120879	H	-1.2284649	0.8812516	-2.0685683
H	4.6885245	-1.2948140	-3.7225637	H	-2.2335787	0.3898368	-3.4197673
H	3.3787559	-0.6029485	-4.6893362	C	-7.5425582	-0.8190271	-0.8625426
H	1.6892236	-3.3435098	-2.8961184	C	-8.0277619	-0.2511241	-2.2054955
H	2.1490924	-2.6661710	-4.4610389	C	-7.6077737	-2.3609017	-0.9321146
H	3.3881560	-3.3451023	-3.3821763	C	-8.4923295	-0.3198065	0.2482844
H	0.5436514	-0.8610597	-2.4666981	H	-8.0267298	0.8448227	-2.2026612
H	1.6316522	0.5222226	-2.5735499	H	-7.4008375	-0.5987194	-3.0325548
H	1.2073325	-0.3914154	-4.0287602	H	-9.0538330	-0.5866124	-2.3933582
C	6.5698539	-1.2451446	0.4709010	H	-7.3149083	-2.8089990	0.0235933
C	7.1892267	-0.3400385	-0.6066357	H	-8.6293757	-2.6874373	-1.1616908
C	6.6286201	-0.4999505	1.8191074	H	-6.9378055	-2.7400510	-1.7097500
C	7.4124164	-2.5364002	0.5691560	H	-9.5226974	-0.6245144	0.0287470
H	7.2325208	-0.8380858	-1.5814488	H	-8.2168681	-0.7323299	1.2241487
H	6.6222236	0.5911038	-0.7200468	H	-8.4615720	0.7730145	0.3198961
H	8.2131454	-0.0789287	-0.3172952	Si	1.3304126	1.0506712	1.3158308
H	6.2467207	-1.1204342	2.6341757	C	2.8024251	0.9297558	2.4678573
H	7.6642456	-0.2255596	2.0540192	C	3.5420974	2.0996534	2.7236825
H	6.0236978	0.4115348	1.7889333	C	3.0801089	-0.2007551	3.2471845
H	8.4544432	-2.2925337	0.8091769	C	4.5188497	2.1354946	3.7179739
H	7.0271414	-3.1981946	1.3517486	H	3.3454099	3.0003566	2.1452858
H	7.3932419	-3.0837540	-0.3797737	C	4.0435068	-0.1684775	4.2556922
C	-3.7597324	-0.7575075	2.4700642	H	2.5324299	-1.1152739	3.0581916
C	-4.8704336	-1.4424996	3.2953392	C	4.7649208	1.0008520	4.4950244
C	-2.5093829	-1.6437811	2.6642835	H	5.0825456	3.0483015	3.8924894
C	-3.5508744	0.6334014	3.1152635	H	4.2369169	-1.0577748	4.8504576
H	-5.7893968	-0.8478503	3.3238521	H	5.5201564	1.0276937	5.2758977
H	-5.1039059	-2.4405339	2.9117524	C	1.8277317	1.9248653	-0.2617135
H	-4.5136562	-1.5546869	4.3249877	C	0.9163277	2.5915201	-1.0982679
H	-2.6810350	-2.6388879	2.2505323	C	3.1845211	1.9373381	-0.6339334

C	1.3362881	3.2522619	-2.2519314
H	-0.1424117	2.5891839	-0.8598392
C	3.6124899	2.5971102	-1.7852646
H	3.9109852	1.4171066	-0.0170026
C	2.6889728	3.2593953	-2.5963118
H	0.6097884	3.7553959	-2.8840014
H	4.6658745	2.5928194	-2.0527618
H	3.0204049	3.7727505	-3.4947265
C	0.1133711	2.0654878	2.3348830
C	-0.4516847	3.2905606	1.9454030
C	-0.1453736	1.6169320	3.6450398
C	-1.2574275	4.0275604	2.8162428
H	-0.2503109	3.6922043	0.9569541
C	-0.9341989	2.3545809	4.5244625
H	0.2900777	0.6805793	3.9871255
C	-1.4980201	3.5628667	4.1094216
H	-1.6877869	4.9698139	2.4871680
H	-1.1155568	1.9861891	5.5306875
H	-2.1183611	4.1390570	4.7905272
Si	-2.3268664	-3.1652465	-1.5651054
C	-2.7730799	-4.0677804	0.0131194
C	-4.1235413	-4.1206424	0.4051948
C	-1.8320250	-4.7140998	0.8326593
C	-4.5163815	-4.7997556	1.5578932
H	-4.8731179	-3.6169274	-0.1967171
C	-2.2164408	-5.3906135	1.9894881
H	-0.7772993	-4.6836517	0.5788886
C	-3.5631631	-5.4384249	2.3531270
H	-5.5658326	-4.8281005	1.8390517
H	-1.4668654	-5.8759483	2.6083518
H	-3.8667903	-5.9653977	3.2534731
C	-1.1314412	-4.1553959	-2.6313360
C	-0.9450912	-3.7127898	-3.9557204
C	-0.5124536	-5.3589198	-2.2563738
C	-0.1744918	-4.4358646	-4.8631002
H	-1.4237385	-2.7934775	-4.2860588
C	0.2739782	-6.0814453	-3.1562895
H	-0.6558186	-5.7551631	-1.2557142
C	0.4419818	-5.6233211	-4.4632563
H	-0.0493856	-4.0727710	-5.8797864
H	0.7463987	-7.0073389	-2.8387384
H	1.0476197	-6.1884107	-5.1665076
C	-3.8418395	-3.0344183	-2.6585719
C	-4.5824748	-4.2046120	-2.9124213
C	-4.1869528	-1.8768820	-3.3671702
C	-5.6330754	-4.2121214	-3.8283499
H	-4.3329092	-5.1258222	-2.3893801
C	-5.2291599	-1.8788089	-4.2954624
H	-3.6359340	-0.9635556	-3.1826446
C	-5.9568805	-3.0457988	-4.5269373
H	-6.1971680	-5.1249927	-4.0007349
H	-5.4758457	-0.9682510	-4.8355286
H	-6.7729597	-3.0484528	-5.2445178

8a : less stable enol isomer of **8** with two COH
106
Energy = -1714.312260188

C	-1.5467009	1.1987952	-0.2187801
H	-1.2534129	1.9809094	0.4814838
H	-1.3139514	1.5983365	-1.2059792
C	-3.0287335	0.8853818	-0.0952701
C	-3.6767855	0.9665320	1.1749727
C	-3.7615063	0.4226572	-1.2251788
C	-4.9881835	0.4957963	1.2839593
C	-5.0749080	-0.0315030	-1.0337562
C	-5.7004508	-0.0314557	0.2074638
H	-5.4815796	0.5376289	2.2458816
H	-5.6269227	-0.4004628	-1.8850517
C	-0.6691941	-0.0181080	0.0264676
O	-1.2778728	-1.2333727	0.3164127
C	1.5474987	-1.1998080	0.2237777
H	1.3156751	-1.5978929	1.2118005
H	1.2533918	-1.9828079	-0.4751129
C	3.0293366	-0.8864240	0.0979992
C	3.7633362	-0.4208237	1.2259291
C	3.6757108	-0.9696031	-1.1729856
C	5.0758347	0.0348313	1.0315425
C	4.9863656	-0.4976157	-1.2848788
C	5.6994786	0.0330659	-0.2106179
H	5.6286487	0.4062019	1.8812682
H	5.4784578	-0.5409891	-2.2474063
C	0.6698430	0.0169200	-0.0221347
O	1.2784343	1.2319346	-0.3132923
C	-3.0443426	1.6189380	2.4330720
C	-3.9854843	1.5435940	3.6563160
C	-1.7341391	0.9351202	2.8846653
C	-2.8213058	3.1299093	2.1745336
H	-4.9323972	2.0657114	3.4846773
H	-4.2004451	0.5086017	3.9439811
H	-3.4870937	2.0294720	4.5015901
H	-0.9105646	1.0547883	2.1823877

H	-1.4196016	1.3643949	3.8427854
H	-1.8945479	-0.1386661	3.0291731
H	-2.3905724	3.5954270	3.0687627
H	-2.1516582	3.3302158	1.3354638
H	-3.7792577	3.6164112	1.9611811
C	-3.2279130	0.4589469	-2.6827018
C	-4.2486303	-0.1236231	-3.6863333
C	-3.0142025	1.9305799	-3.1166199
C	-1.9396969	-0.3701189	-2.8860683
H	-4.4657100	-1.1780608	-3.4835816
H	-5.1902617	0.4350307	-3.6933097
H	-3.8162333	-0.0575934	-4.6901981
H	-2.2898424	2.4625726	-2.4961301
H	-2.6570090	1.9594768	-4.1527496
H	-3.9629983	2.4755697	-3.0647127
H	-1.6983586	-0.3974944	-3.9548821
H	-1.0725397	0.0280446	-2.3614681
H	-2.0907803	-1.3998487	-2.5448730
C	-7.1226489	-0.5588443	0.4216017
C	-7.7477741	-1.0975853	-0.8752294
C	-7.0867442	-1.7035862	1.4586217
C	-8.0164207	0.5818994	0.9571377
H	-7.8302672	-0.3154351	-1.6382243
H	-7.1627543	-1.9258883	-1.2901651
H	-8.7564392	-1.4693450	-0.6648379
H	-6.6806675	-1.3603046	2.4155017
H	-8.0996148	-2.0844910	1.6343820
H	-6.4631127	-2.5298952	1.1002335
H	-9.0356894	0.2144347	1.1243983
H	-7.6360832	0.9737831	1.9058286
H	-8.0589455	1.4082715	0.2392901
C	3.2320642	-0.4552475	2.6843531
C	4.2542809	0.1288931	3.6855251
C	1.9439586	0.3737608	2.8886814
C	3.0194150	-1.9263300	3.1206147
H	5.1961842	-0.4293143	3.6912947
H	4.4704786	1.1832293	3.4813349
H	3.8237467	0.0636831	4.6902486
H	1.0763552	-0.0247536	2.3650596
H	1.7036536	0.4015759	3.9577210
H	2.0946305	1.4033954	2.5469972
H	2.6640381	-1.9538575	4.1574062
H	2.2941217	-2.4593674	2.5021245
H	3.9682395	-2.4711923	3.0678442
C	3.0420541	-1.6250281	-2.4288681
C	3.9820873	-1.5527400	-3.6531535
C	2.8189680	-3.1353923	-2.1668992
C	1.7316381	-0.9417621	-2.8806812
H	4.1964370	-0.5184964	-3.9439507
H	4.9293372	-2.0740400	-3.4808443
H	3.4830933	-2.0411266	-4.4966246
H	2.1501657	-3.3340194	-1.3267647
H	2.3872272	-3.6026169	-3.0597475
H	3.7770678	-3.6216305	-1.9535978
H	1.4160530	-1.3728913	-3.8376273
H	0.9087388	-1.0597540	-2.1773384
H	1.8922715	0.1316840	-3.0273991
C	7.1205319	0.5621377	-0.4281449
C	7.0812519	1.7043102	-1.4678703
C	7.7467835	1.1049905	0.8664239
C	8.0152498	-0.5785097	-0.9623131
H	6.6741336	1.3580938	-2.4232488
H	6.4569461	2.5305463	-1.1104805
H	8.0932657	2.0862862	-1.6462053
H	7.8316302	0.3248516	1.6312121
H	8.7545577	1.4777754	0.6535984
H	7.1611354	1.9334265	1.2802139
H	9.0337459	-0.2099242	-1.1317993
H	8.0599730	-1.4031842	-0.2426479
H	7.6341911	-0.9731153	-1.9095849
H	-2.2405342	-1.0646871	0.3124345
H	2.2412723	1.0644330	-0.3040860

8k : single K/$SiPh_3$ replacement of complex **Dt**
139
Energy = -3298.113571172

C	0.8981928	-1.1288228	-2.0007900
H	0.2742632	-2.0158646	-2.0973528
H	0.9001430	-0.6560466	-2.9881401
C	2.3146907	-1.4609410	-1.5753115
C	2.5795135	-2.5642035	-0.7121860
C	3.3920662	-0.6187243	-1.9587275
C	3.8142699	-2.6190555	-0.0632509
C	4.6116031	-0.7360904	-1.2739202
C	4.8247961	-1.6809678	-0.2759939
H	4.0054656	-3.4226636	0.6368789
H	5.4169097	-0.0609444	-1.5252646
C	0.1479226	-0.2590761	-1.0150340
O	0.7776144	0.9481209	-0.6337262
C	-1.9487142	0.5844146	0.0704181
H	-1.4845878	0.6493207	1.0534337

H	-1.7936059	1.5698887	-0.3702654
C	-3.4226897	0.2713514	0.1786921
C	-3.9411516	-0.4601573	1.2830262
C	-4.2996829	0.6201796	-0.8929455
C	-5.2765612	-0.9016770	1.2448081
C	-5.6093554	0.1254908	-0.8777246
C	-6.1196378	-0.6548775	0.1638078
H	-5.6654210	-1.4618852	2.0832356
H	-6.2685195	0.3567001	-1.7047339
C	-1.1843050	-0.4613015	-0.7770796
O	-1.8459730	-1.4885730	-1.2442666
C	1.6411026	-3.7949988	-0.5826294
C	2.3006921	-4.9319933	0.2284237
C	0.2903079	-3.5219158	0.1089806
C	1.4069906	-4.3809021	-1.9981264
H	3.2537603	-5.2515544	-0.2065500
H	2.4721018	-4.6437991	1.2714597
H	1.6226227	-5.7931213	0.2291690
H	-0.3050557	-2.7386551	-0.3636804
H	-0.2996846	-4.4498564	0.0991631
H	0.4497412	-3.2528798	1.1574269
H	0.7959596	-5.2894814	-1.9238456
H	0.8973309	-3.6818318	-2.6636102
H	2.3659796	-4.6459146	-2.4569913
C	3.3615797	0.2860318	-3.2251337
C	4.7388893	0.9338651	-3.4915388
C	3.0840956	-0.6256163	-4.4476568
C	2.3647325	1.4666055	-3.2065395
H	5.0201910	1.6282342	-2.6931825
H	5.5309529	0.1861389	-3.6068020
H	4.6766082	1.5039437	-4.4254130
H	2.1107136	-1.1185918	-4.3879953
H	3.1110705	-0.0292950	-5.3684552
H	3.8516011	-1.4048611	-4.5160521
H	1.3252155	1.1676582	-3.0893846
H	2.6036654	2.1514895	-2.3909524
H	2.4613447	2.0116135	-4.1548575
C	6.1193205	-1.7614561	0.5392512
C	7.1342516	-0.6820259	0.1327374
C	5.7776170	-1.5737885	2.0332973
C	6.7806146	-3.1431001	0.3407842
H	7.4338581	-0.7831795	-0.9163941
H	6.7255847	0.3232089	0.2768831
H	8.0336002	-0.7725800	0.7522641
H	5.0764245	-2.3403098	2.3760537
H	6.6872843	-1.6366308	2.6438000
H	5.3069612	-0.6007572	2.2063225
H	7.7039121	-3.2095624	0.9292829
H	6.1159305	-3.9523044	0.6596131
H	7.0287754	-3.3027308	-0.7143585
C	-3.1183451	-0.7923202	2.5543829
C	-3.9418888	-1.5794323	3.5981909
C	-1.9043554	-1.6902361	2.2319037
C	-2.6900362	0.5108530	3.2726222
H	-4.8195648	-1.0210105	3.9415300
H	-4.2722698	-2.5516463	3.2149736
H	-3.3033160	-1.7651410	4.4687603
H	-1.2233364	-1.2656134	1.4972195
H	-1.3378348	-1.8841874	3.1502231
H	-2.2488742	-2.6539524	1.8377726
H	-2.0927038	0.2626001	4.1581786
H	-2.1007999	1.1833391	2.6503087
H	-3.5800119	1.0599218	3.6003892
C	-3.9193598	1.5989359	-2.0382995
C	-5.0368321	1.7015234	-3.1006069
C	-3.7688730	3.0130861	-1.4295744
C	-2.6363367	1.2106294	-2.8167722
H	-5.2209956	0.7398485	-3.5939752
H	-5.9803775	2.0669912	-2.6820708
H	-4.7181639	2.4162437	-3.8666208
H	-3.0221782	3.0389596	-0.6342933
H	-3.4688540	3.7253500	-2.2083632
H	-4.7226393	3.3459656	-1.0046394
H	-2.6423040	1.7215333	-3.7867679
H	-1.7179626	1.4928666	-2.3008169
H	-2.5923120	0.1301464	-2.9764525
C	-7.5580629	-1.1822714	0.0886075
C	-7.6971065	-2.1023077	-1.1452585
C	-7.9520218	-1.9868040	1.3377916
C	-8.5381080	0.0033509	-0.0560596
H	-7.4206358	-1.5781389	-2.0657504
H	-7.0529705	-2.9850996	-1.0436059
H	-8.7307615	-2.4527938	-1.2476721
H	-7.9011173	-1.3727820	2.2436022
H	-8.9813940	-2.3461874	1.2310126
H	-7.3034664	-2.8594255	1.4763704
H	-9.5698489	-0.3633381	-0.1119874
H	-8.4547079	0.6770171	0.8034327
H	-8.3327062	0.5816891	-0.9623135
Si	1.3963452	1.6998520	0.6875692
C	1.9520088	0.5562062	2.0599550
C	2.7142219	1.0431136	3.1393370

C 1.5974662 -0.7998077 2.0629862
C 3.0903125 0.2089579 4.1913403
H 3.0244261 2.0862443 3.1539137
C 1.9741254 -1.6400921 3.1109880
H 1.0278150 -1.1924344 1.2261992
C 2.7152115 -1.1373217 4.1798379
H 3.6802446 0.6034316 5.0149196
H 1.6953494 -2.6906317 3.0909805
H 3.0113228 -1.7915344 4.9959516
C 2.8817414 2.7279988 0.1512941
C 2.7381980 4.0244866 -0.3746594
C 4.1848843 2.2151937 0.2694542
C 3.8426589 4.7684995 -0.7912477
H 1.7478643 4.4640085 -0.4597079
C 5.2955266 2.9576116 -0.1333302
H 4.3283940 1.2170970 0.6722317
C 5.1273630 4.2346731 -0.6713883
H 3.7028977 5.7649322 -1.2029710
H 6.2947027 2.5414843 -0.0332409
H 5.9916016 4.8116536 -0.9900797
C 0.1355425 2.9252186 1.3683703
C -0.5909905 3.7594877 0.4980432
C -0.0775899 3.0852776 2.7472861
C -1.4842045 4.7146073 0.9818914
H -0.4684813 3.6480127 -0.5774927
C -0.9750548 4.0346755 3.2404239
H 0.4539815 2.4457363 3.4475841
C -1.6787242 4.8548182 2.3579693
H -2.0350531 5.3442096 0.2879852
H -1.1301633 4.1289920 4.3123544
H -2.3790616 5.5940751 2.7379083
K -3.6772912 -3.0494989 -1.1809447

8 : observed product **8** with *C*HOH and *C*=O sites
106
Energy = -1714.323084005
C -1.5756143 0.0413675 1.0295570
H -1.1908985 -0.9719752 1.1139737
H -1.3494298 0.5487009 1.9660872
C -3.0422700 0.0512450 0.6731305
C -3.5927612 -1.0329965 -0.0749062
C -3.8338922 1.2069596 0.9167928
C -4.7987331 -0.8181289 -0.7525062
C -5.0358221 1.3459513 0.2107709
C -5.5054951 0.3823016 -0.6789508
H -5.2104649 -1.6171515 -1.3558965
H -5.6257563 2.2388931 0.3583091
C -0.7758188 0.7824863 -0.0879656
O -1.0585450 0.2926041 -1.3982359
C 1.5562062 -0.1557381 -0.7599033
H 1.1458907 -1.1676691 -0.7306576
H 1.3067782 0.1885902 -1.7633034
C 3.0348785 -0.1141311 -0.4729056
C 3.8169152 0.9538499 -0.9963625
C 3.6360674 -1.0839282 0.3692362
C 5.1470572 1.0625025 -0.5840748
C 4.9789323 -0.9114471 0.7343465
C 5.7463263 0.1610667 0.2952902
H 5.7496270 1.8796431 -0.9582235
H 5.4403805 -1.6372367 1.3872873
C 0.7164757 0.6611289 0.2109751
O 1.1571708 1.1946228 1.2164559
C -3.0237473 -2.4821537 -0.0377688
C -4.0114036 -3.4838276 -0.6775437
C -1.6893412 -2.6916253 -0.7892891
C -2.8840551 -2.9243907 1.4405756
H -4.9989126 -3.4406964 -0.2068538
H -4.1262836 -3.3186718 -1.7541829
H -3.6140513 -4.4946987 -0.5386744
H -1.7871367 -2.4053948 -1.8409811
H -0.8591267 -2.1238143 -0.3711597
H -1.4235411 -3.7542781 -0.7462907
H -2.5383118 -3.9637883 1.4791874
H -3.8559751 -2.8658210 1.9418677
H -2.1788096 -2.3146701 2.0089791
C -3.5154080 2.2459952 2.0272024
C -4.6609545 3.2701225 2.1850051
C -3.4238798 1.4996198 3.3811476
C -2.2374035 3.0879580 1.8024245
H -4.7729710 3.9016095 1.2968612
H -5.6195027 2.7843852 2.3943639
H -4.4239299 3.9239138 3.0308007
H -2.6303462 0.7490999 3.3970030
H -3.2278866 2.2178624 4.1857601
H -4.3704455 0.9919178 3.5947845
H -2.2074207 3.8863549 2.5528505
H -2.2507022 3.5595463 0.8137027
H -1.3130726 2.5169849 1.9042819
C -6.7795384 0.5682837 -1.5086901
C -7.4117354 1.9544494 -1.3042987
C -6.4316049 0.4059939 -3.0053126

C	-7.8167156	-0.5054314	-1.1103663
H	-7.7257874	2.1059948	-0.2655533
H	-6.7169392	2.7566460	-1.5767660
H	-8.2996119	2.0457336	-1.9391874
H	-6.0054001	-0.5817166	-3.2088463
H	-7.3342730	0.5210104	-3.6166863
H	-5.7031042	1.1625224	-3.3167703
H	-8.7295684	-0.3859083	-1.7055631
H	-7.4301032	-1.5154451	-1.2801966
H	-8.0789742	-0.4140726	-0.0507510
C	3.3102336	1.9621272	-2.0650647
C	4.3776036	3.0314626	-2.3913555
C	3.0467911	1.2152463	-3.3961070
C	2.0541487	2.7614226	-1.6355619
H	4.6313997	3.6359942	-1.5136084
H	5.2957690	2.5901307	-2.7916699
H	3.9714509	3.7016828	-3.1559674
H	2.2903380	0.4314869	-3.3073959
H	2.7064986	1.9261207	-4.1583617
H	3.9711931	0.7464656	-3.7498408
H	1.1310862	2.1813641	-1.6861495
H	1.9260465	3.6120922	-2.3141872
H	2.1658472	3.1475330	-0.6183045
C	2.9200707	-2.3689000	0.8672285
C	3.8377444	-3.2274817	1.7671062
C	1.6701918	-2.0738755	1.7295519
C	2.5576108	-3.2756682	-0.3351266
H	4.7415859	-3.5534718	1.2425101
H	4.1320013	-2.6949997	2.6779651
H	3.2848067	-4.1241745	2.0663079
H	1.3399603	-2.9988643	2.2156769
H	0.8261966	-1.7001205	1.1504368
H	1.8987799	-1.3358852	2.5036287
H	2.0627709	-4.1857189	0.0243443
H	1.8920274	-2.7977066	-1.0577047
H	3.4687329	-3.5672144	-0.8682221
C	7.2044628	0.3679536	0.7184918
C	7.6933616	-0.7224848	1.6854052
C	8.1100509	0.3501565	-0.5330822
C	7.3388258	1.7375066	1.4206893
H	7.0973388	-0.7416510	2.6046441
H	7.6522896	-1.7163478	1.2257939
H	8.7343113	-0.5228287	1.9625322
H	7.8294080	1.1391243	-1.2381853
H	9.1562889	0.5069811	-0.2446684
H	8.0330885	-0.6122085	-1.0507706
H	8.3796000	1.9082386	1.7206547
H	7.0364121	2.5546396	0.7578001
H	6.7087759	1.7745727	2.3160555
H	-2.0151096	0.0954293	-1.4235292
H	-1.0231725	1.8486759	-0.0348335

(**At**)$_2$: dimer of carbene-like **At**

106

Energy = -2913.044709023

K	-1.2209946	-0.3067900	0.5962414
C	0.1748389	-0.5883960	-3.4522437
H	0.2320718	0.2102164	-4.1898257
H	0.7075682	-1.4325422	-3.8937170
C	-1.2211997	-0.9181883	-3.0374799
C	-2.1835236	0.1265477	-2.8750719
C	-1.5572023	-2.2349779	-2.5862137
C	-3.3933382	-0.1525037	-2.2188127
C	-2.7738824	-2.4340404	-1.9231556
C	-3.7041036	-1.4127308	-1.7060081
H	-4.1197482	0.6394057	-2.1036769
H	-3.0220689	-3.4269440	-1.5686324
C	1.1532359	-0.1172235	-2.2666609
O	0.7728385	-0.3813433	-1.1088945
K	2.3417013	1.6817703	-0.4704524
C	0.9760711	1.9161007	3.5907696
H	0.4492014	2.7543914	4.0500069
H	0.9229942	1.1060906	4.3161498
C	2.3696090	2.2491289	3.1700237
C	2.7047927	3.5721461	2.7366049
C	3.3283813	1.2051440	2.9834361
C	3.9162264	3.7790921	2.0664286
C	4.5327716	1.4918846	2.3204929
C	4.8421028	2.7593352	1.8250013
H	4.1638090	4.7770216	1.7258312
H	5.2562523	0.7002892	2.1864061
C	-0.0137374	1.4658834	2.4062376
O	0.3579603	1.7451847	1.2492618
C	-1.9912553	1.5609325	-3.4373970
C	-3.2144409	2.4638142	-3.1588128
C	-0.7921338	2.3032960	-2.7969634
C	-1.8578514	1.5170246	-4.9799020
H	-4.1310923	2.0674261	-3.6079175
H	-3.3833288	2.6081304	-2.0857553
H	-3.0245814	3.4478929	-3.6003988
H	0.1751654	1.8397217	-3.0043615
H	-0.7625229	3.3315102	-3.1774510

H	-0.9160724	2.3507377	-1.7082511
H	-2.7760741	1.1095866	-5.4169200
H	-1.7148551	2.5328607	-5.3674091
H	-1.0249100	0.9019526	-5.3241753
C	-0.6870436	-3.4863878	-2.8818788
C	-1.3607894	-4.7867157	-2.3884212
C	-0.5318450	-3.6597183	-4.4134747
C	0.6950409	-3.4589635	-2.1861583
H	-1.4734478	-4.8040085	-1.2983988
H	-2.3442050	-4.9401357	-2.8451953
H	-0.7249394	-5.6330752	-2.6693083
H	-0.0751715	-2.7952497	-4.8988774
H	0.0893031	-4.5383892	-4.6245513
H	-1.5157495	-3.8166738	-4.8691296
H	1.1949502	-4.4209461	-2.3527292
H	1.3538023	-2.6703757	-2.5463267
H	0.5744437	-3.3166490	-1.1072507
C	-5.0032061	-1.7098056	-0.9466719
C	-4.6592822	-2.1890637	0.4816774
C	-5.9104039	-0.4739409	-0.8351937
C	-5.7863370	-2.8253836	-1.6743943
H	-4.0037450	-3.0658618	0.4609591
H	-4.1573594	-1.3935103	1.0472379
H	-5.5711994	-2.4575775	1.0274808
H	-6.2147482	-0.1086562	-1.8221198
H	-6.8164233	-0.7349881	-0.2775844
H	-5.4127387	0.3448712	-0.3029704
H	-6.7162252	-3.0474921	-1.1375200
H	-6.0390773	-2.5139252	-2.6934989
H	-5.2004970	-3.7479899	-1.7359960
C	1.8403314	4.8207071	3.0598232
C	2.5133579	6.1273718	2.5823669
C	0.4524480	4.8083724	2.3753268
C	1.6983528	4.9690890	4.5953199
H	3.5008204	6.2708562	3.0335818
H	2.6171786	6.1623668	1.4918828
H	1.8819734	6.9705075	2.8823895
H	-0.2052858	4.0154886	2.7278037
H	-0.0436174	5.7686220	2.5622541
H	0.5637197	4.6839012	1.2932171
H	1.0817161	5.8459866	4.8261081
H	1.2431168	4.0980403	5.0702259
H	2.6864769	5.1156884	5.0452662
C	3.1380270	-0.2374580	3.5251939
C	4.3564550	-1.1385985	3.2212591
C	3.0189811	-0.2168010	5.0692904
C	1.9312316	-0.9674139	2.8850713
H	4.5148607	-1.2667084	2.1445552
H	5.2782569	-0.7513177	3.6677737
H	4.1684210	-2.1289669	3.6493838
H	2.1908110	0.3951913	5.4302433
H	2.8766498	-1.2380287	5.4426296
H	3.9422233	0.1815544	5.5041032
H	1.9052972	-2.0022985	3.2474148
H	0.9670281	-0.5072334	3.1129204
H	2.0418252	-0.9952247	1.7943643
C	6.1364257	3.0659857	1.0612536
C	7.0371605	1.8287109	0.9178433
C	5.7835384	3.5754870	-0.3544409
C	6.9302712	4.1632234	1.8050058
H	7.3479094	1.4425989	1.8947895
H	6.5314730	1.0227773	0.3736447
H	7.9397704	2.0968406	0.3580518
H	5.1325156	4.4547830	-0.3108706
H	6.6924308	3.8504850	-0.9020923
H	5.2731675	2.7939230	-0.9318450
H	7.8566037	4.3925232	1.2650031
H	6.3488060	5.0867026	1.8903797
H	7.1901731	3.8299250	2.8153768

At : carbene-like adduct of CO and **4** (KBzt)
53
Energy = -1456.504889981

K	-3.4375466	-0.1677271	2.6273830
C	-1.5950789	-0.3103326	-1.1494522
H	-1.1244301	0.5187851	-1.6760097
H	-1.3164216	-1.2093372	-1.6967065
C	-3.0541117	-0.1498037	-0.9519209
C	-3.6243655	1.1472656	-0.7323056
C	-3.8897887	-1.3001517	-0.7370089
C	-4.9389165	1.2502886	-0.2492125
C	-5.1904076	-1.1191880	-0.2538085
C	-5.7408202	0.1396641	0.0209330
H	-5.3589390	2.2335610	-0.0895789
H	-5.8162551	-1.9892978	-0.0956423
C	-0.6649270	-0.4247993	0.2249404
O	-1.2733536	-0.3236940	1.2980456
C	-2.8896363	2.4663691	-1.0909929
C	-3.7778871	3.7095749	-0.8578165
C	-1.6238102	2.7097031	-0.2338376
C	-2.5484668	2.4847466	-2.6019586
H	-4.6995010	3.6746162	-1.4485161

H	-4.0426972	3.8340184	0.1983868
H	-3.2161372	4.5979933	-1.1661267
H	-0.8405288	1.9670660	-0.3826116
H	-1.2091687	3.6953184	-0.4791255
H	-1.8843720	2.7071528	0.8307553
H	-2.0108033	3.4082087	-2.8488479
H	-1.9342070	1.6381657	-2.9127740
H	-3.4736717	2.4590299	-3.1885099
C	-3.4517598	-2.7438108	-1.0993019
C	-4.5824143	-3.7707597	-0.8642016
C	-3.1266657	-2.8311879	-2.6113342
C	-2.2637830	-3.2504523	-0.2459683
H	-4.8672952	-3.8342915	0.1922557
H	-5.4756993	-3.5428451	-1.4553171
H	-4.2211715	-4.7584755	-1.1703651
H	-2.3438858	-2.1376280	-2.9223112
H	-2.8030937	-3.8486633	-2.8616452
H	-4.0256621	-2.6034859	-3.1947568
H	-2.0575340	-4.2961567	-0.5052114
H	-1.3450443	-2.6804405	-0.3827861
H	-2.5218082	-3.2115929	0.8187624
C	-7.1711998	0.2492281	0.5637640
C	-7.2583532	-0.4632067	1.9326037
C	-7.6140100	1.7092493	0.7520571
C	-8.1521430	-0.4321676	-0.4169072
H	-6.9354440	-1.5070189	1.8590783
H	-6.6280607	0.0451813	2.6743385
H	-8.2875108	-0.4501177	2.3103065
H	-7.6036999	2.2566774	-0.1967961
H	-8.6363417	1.7337822	1.1451749
H	-6.9676677	2.2385791	1.4617023
H	-9.1791101	-0.3591227	-0.0392943
H	-8.1074889	0.0502157	-1.3991116
H	-7.9126472	-1.4921687	-0.5479479

Bta : less stable conformer of **Bt**
55
Energy = -1569.916589906

K	0.5112809	2.6402367	-0.1404367
C	-0.1273088	-0.8239624	2.2335691
H	0.6260092	-1.5471330	2.5444763
H	-1.0728910	-1.1714937	2.6483110
C	-0.2078102	-0.7672315	0.7212679
C	0.9644889	-0.9662910	-0.0702837
C	-1.4352051	-0.4432281	0.0766152
C	0.8882286	-0.7395618	-1.4506216
C	-1.4356229	-0.2364680	-1.3144047
C	-0.2886653	-0.3514277	-2.0962699
H	1.7765857	-0.8729893	-2.0545272
H	-2.3636659	0.0219855	-1.8028868
C	2.3153498	-1.4889792	0.4898876
C	2.9225368	-0.5750798	1.5786191
C	2.1348876	-2.9376525	1.0099939
C	3.4000801	-1.5837526	-0.6074667
H	2.2973616	-0.4584989	2.4639026
H	3.8896286	-0.9895097	1.8881714
H	3.0951598	0.4273238	1.1714869
H	1.8088952	-3.5882832	0.1910198
H	3.0934472	-3.3131759	1.3871957
H	1.4008191	-3.0201717	1.8139263
H	4.3196395	-1.9609374	-0.1477957
H	3.1225945	-2.2753874	-1.4100521
H	3.6241792	-0.6054427	-1.0474702
C	-2.8063851	-0.3643249	0.8021673
C	-3.1913345	-1.7665299	1.3382943
C	-2.8438980	0.6946639	1.9275197
C	-3.9507219	0.0305826	-0.1597686
H	-3.2625989	-2.4754547	0.5060157
H	-2.4731901	-2.1668063	2.0566568
H	-4.1696413	-1.7145574	1.8307096
H	-2.6263171	1.6858321	1.5148555
H	-3.8525166	0.7168075	2.3578474
H	-2.1294676	0.5179849	2.7316167
H	-4.8829301	0.0641746	0.4138603
H	-3.7931386	1.0223761	-0.5984387
H	-4.0851120	-0.6947115	-0.9692946
C	-0.2804569	-0.0932461	-3.6081816
C	-1.6689508	0.2916856	-4.1436102
C	0.6985570	1.0610156	-3.9207544
C	0.1907670	-1.3664343	-4.3459673
H	-2.3995832	-0.5073357	-3.9769816
H	-2.0442158	1.2065925	-3.6711432
H	-1.6063023	0.4715277	-5.2222823
H	1.7070364	0.8378277	-3.5579235
H	0.7544549	1.2319110	-5.0019746
H	0.3597036	1.9935118	-3.4518150
H	0.2046248	-1.1915885	-5.4281034
H	1.1990372	-1.6576117	-4.0353541
H	-0.4848037	-2.2030194	-4.1381229
C	0.2175112	0.5700023	2.8324812
O	0.4044271	1.6410497	2.0548565
C	0.3176782	0.6454813	4.1568108

O 0.4158682 0.7464786 5.3513962

Bt : ketene-like adduct of two CO and **4** (KBzt)
55
Energy = -1569.918587102

K	-1.3585926	2.8883333	3.8835699
C	-0.1080286	-0.7125218	2.2433912
H	0.6618150	-1.3818933	2.6356148
H	-1.0360033	-1.0017395	2.7434417
C	-0.2144750	-0.7771113	0.7338391
C	0.9587865	-0.9766411	-0.0502182
C	-1.4410099	-0.4581887	0.0909008
C	0.8920474	-0.7147210	-1.4212511
C	-1.4320604	-0.2148550	-1.2898134
C	-0.2756544	-0.2957597	-2.0584373
H	1.7836246	-0.8335230	-2.0233463
H	-2.3561755	0.0541255	-1.7801123
C	2.2835847	-1.5577753	0.5125694
C	2.9727518	-0.6539821	1.5626689
C	2.0186349	-2.9716549	1.0877760
C	3.3366886	-1.7594546	-0.6002167
H	2.4007997	-0.5284784	2.4813069
H	3.9439804	-1.0939689	1.8216212
H	3.1482679	0.3420845	1.1430249
H	1.6429529	-3.6299871	0.2966398
H	2.9546558	-3.3948765	1.4716989
H	1.2880860	-2.9740390	1.8991770
H	4.2263716	-2.2160672	-0.1533070
H	2.9784068	-2.4275047	-1.3905656
H	3.6411762	-0.8097682	-1.0535259
C	-2.8163895	-0.4554968	0.8108326
C	-3.0979503	-1.8641142	1.3894590
C	-2.9439275	0.6210945	1.9150596
C	-3.9789428	-0.1686460	-0.1656720
H	-3.1233959	-2.5980573	0.5769240
H	-2.3431619	-2.1877116	2.1087330
H	-4.0736127	-1.8717652	1.8904076
H	-2.7257075	1.6086953	1.4915035
H	-3.9738918	0.6274840	2.2921629
H	-2.2749262	0.4380902	2.7574648
H	-4.9203409	-0.2259320	0.3915161
H	-3.9097720	0.8327914	-0.6044394
H	-4.0242020	-0.9036701	-0.9755476
C	-0.2496230	0.0272614	-3.5558779
C	-1.6249977	0.4656077	-4.0841654
C	0.7560552	1.1733329	-3.8058598
C	0.1995434	-1.2214701	-4.3466313
H	-2.3735214	-0.3257089	-3.9644837
H	-1.9855491	1.3631063	-3.5692374
H	-1.5479854	0.6970955	-5.1522705
H	1.7624133	0.8943372	-3.4778838
H	0.7981166	1.4140310	-4.8749420
H	0.4579314	2.0733798	-3.2572636
H	0.2330938	-0.9979251	-5.4196967
H	1.1961282	-1.5511275	-4.0362771
H	-0.4984765	-2.0505018	-4.1863867
C	0.2489149	0.7054408	2.6499191
O	0.3696358	1.0589647	3.9397071
C	0.4405857	1.6111088	1.6788604
O	0.6721329	2.5321422	0.9434761

Ct : formal C-C coupling of two carbene **At**
106
Energy = -2913.098684704

K	-1.8055689	1.1047364	2.0376577
C	-1.3306330	-0.3108978	-1.4064666
H	-0.8833256	0.5110105	-1.9647896
H	-1.1406163	-1.2293814	-1.9766317
C	-2.8160334	-0.1447018	-1.1916607
C	-3.4031021	1.1440350	-1.0510384
C	-3.6154217	-1.2941485	-0.9276081
C	-4.6562716	1.2491315	-0.4221680
C	-4.8508659	-1.1200110	-0.2982251
C	-5.3673144	0.1368429	0.0250571
H	-5.0913806	2.2296252	-0.2851579
H	-5.4379501	-1.9929659	-0.0388770
C	-0.5929444	-0.3508828	-0.0385411
O	-1.1487266	-1.0189728	0.9763128
K	1.8266575	-1.0722797	-2.0447649
C	1.3357738	0.3251802	1.4082709
H	1.1509691	1.2429326	1.9813534
H	0.8882782	-0.4968632	1.9663194
C	2.8201861	0.1562756	1.1880213
C	3.6210274	1.3049811	0.9250931
C	3.4041433	-1.1330564	1.0406438
C	4.8534210	1.1307717	0.2899484
C	4.6550752	-1.2384187	0.4071427
C	5.3663648	-0.1259611	-0.0393572
H	5.4411089	2.0035493	0.0313169
H	5.0879758	-2.2192313	0.2654411
C	0.5946924	0.3694191	0.0421080

O	1.1491081	1.0403133	-0.9719359
C	-2.7772373	2.4378664	-1.6388592
C	-3.7594241	3.6286412	-1.5746725
C	-1.5005284	2.9019587	-0.9045334
C	-2.4863865	2.2261817	-3.1453037
H	-4.7069162	3.4087931	-2.0784840
H	-3.9746723	3.9307809	-0.5433589
H	-3.2971149	4.4848033	-2.0783944
H	-0.6836368	2.1740874	-0.9085657
H	-1.1302943	3.8202672	-1.3789864
H	-1.7462519	3.1728061	0.1326641
H	-2.0684637	3.1458911	-3.5724912
H	-1.7819423	1.4136813	-3.3288744
H	-3.4172229	1.9923166	-3.6739932
C	-3.2465627	-2.7107406	-1.4502692
C	-4.3380348	-3.7438620	-1.0971187
C	-3.2115491	-2.6322732	-2.9969557
C	-1.9028036	-3.2859095	-0.9130035
H	-4.4218657	-3.8918968	-0.0142951
H	-5.3208819	-3.4597130	-1.4884981
H	-4.0634566	-4.7045456	-1.5460730
H	-2.4638426	-1.9128545	-3.3439354
H	-2.9668840	-3.6161652	-3.4168092
H	-4.1872412	-2.3206070	-3.3874368
H	-2.0021996	-4.3657906	-0.7495813
H	-1.0894215	-3.1480670	-1.6345282
H	-1.6089397	-2.7850920	0.0156193
C	-6.6896022	0.2458847	0.7918914
C	-6.5398874	-0.4640733	2.1560396
C	-7.0973521	1.7054439	1.0497621
C	-7.8180094	-0.4390935	-0.0105820
H	-6.2673029	-1.5161933	2.0259383
H	-5.7589398	0.0164933	2.7569850
H	-7.4819994	-0.4193647	2.7158466
H	-7.2623283	2.2494624	0.1131633
H	-8.0316286	1.7288307	1.6218012
H	-6.3337997	2.2391502	1.6273564
H	-8.7664368	-0.3760410	0.5366926
H	-7.9458156	0.0465886	-0.9841760
H	-7.5948776	-1.4964608	-0.1849289
C	3.2568586	2.7203498	1.4541922
C	4.3494678	3.7523486	1.1014053
C	1.9125536	3.3002886	0.9227485
C	3.2266406	2.6364854	3.0008247
H	5.3328755	3.4644568	1.4885942
H	4.4301674	3.9042715	0.0188780
H	4.0784397	4.7119552	1.5548181
H	1.1026601	3.1673867	1.6492343
H	2.0155034	4.3794371	0.7566996
H	1.6118985	2.7992592	-0.0036829
H	2.9855809	3.6196168	3.4245653
H	2.4780328	1.9178709	3.3475455
H	4.2025383	2.3205323	3.3872978
C	2.7775343	-2.4280222	1.6248342
C	3.7569285	-3.6205944	1.5520544
C	2.4917736	-2.2224689	3.1330319
C	1.4975737	-2.8854776	0.8923496
H	3.9678104	-3.9188840	0.5187030
H	4.7067343	-3.4049130	2.0533081
H	3.2946094	-4.4778811	2.0538380
H	1.7896788	-1.4092326	3.3222398
H	2.0730049	-3.1431330	3.5573440
H	3.4246917	-1.9928934	3.6599090
H	1.1297118	-3.8084384	1.3595588
H	0.6809415	-2.1575663	0.9071535
H	1.7377026	-3.1455515	-0.1488736
C	6.6859593	-0.2349399	-0.8108557
C	7.0894586	-1.6943626	-1.0761131
C	6.5339332	0.4810872	-2.1715602
C	7.8183089	0.4440030	-0.0088030
H	7.2561115	-2.2427188	-0.1423561
H	6.3227842	-2.2238513	-1.6534823
H	8.0218515	-1.7175871	-1.6512217
H	6.2636461	1.5331516	-2.0363860
H	7.4744190	0.4369448	-2.7341394
H	5.7505604	0.0045163	-2.7725891
H	8.7650830	0.3808916	-0.5589289
H	7.5981386	1.5011648	0.1704991
H	7.9474935	-0.0459787	0.9624406

dAt : carbene-like adduct of CO and dimer (**4**)$_2$
104
Energy = -2799.656285563

C	-1.5002195	-3.0842020	0.5140737
H	-1.5946359	-3.9092967	-0.1784055
H	-1.0385158	-3.3522480	1.4547546
C	-2.3792104	-1.9844299	0.4389492
C	-3.2523254	-1.7550155	-0.7212769
C	-2.4728607	-0.9729339	1.4969764
C	-4.2273653	-0.7614338	-0.6697759
C	-3.4662268	0.0070888	1.4423766

C	-4.3945761	0.1336231	0.3997951
H	-4.9105979	-0.6720739	-1.5081527
H	-3.5382902	0.7031928	2.2678249
K	0.9120605	-1.7824389	-0.6625636
C	2.1023953	2.2464216	-1.5668583
H	2.4917562	2.3568609	-2.5806666
H	2.0194208	3.2618277	-1.1804133
C	2.9264096	1.3446761	-0.7038319
C	3.6973238	0.2816520	-1.2711423
C	2.7719149	1.3960188	0.7144551
C	4.1857775	-0.7257262	-0.4284431
C	3.2834133	0.3494820	1.4950533
C	3.9659293	-0.7383963	0.9527513
H	4.7654640	-1.5342950	-0.8575405
H	3.1434507	0.3820226	2.5657097
K	-2.1276984	1.3824378	-1.2363728
C	0.5952617	1.7708673	-1.8013755
O	0.3562659	0.5739416	-1.5216293
C	-3.1999829	-2.6373979	-1.9915577
C	-4.0768332	-2.0791459	-3.1341790
C	-1.7739254	-2.7135839	-2.5903459
C	-3.7429692	-4.0579180	-1.7004609
H	-5.1412083	-2.0782718	-2.8785701
H	-3.7865864	-1.0583642	-3.4113978
H	-3.9507251	-2.7166253	-4.0164419
H	-1.0771013	-3.2306550	-1.9292872
H	-1.8001404	-3.2571248	-3.5417974
H	-1.3927983	-1.7039413	-2.7922825
H	-3.6285363	-4.6984889	-2.5843551
H	-3.2351816	-4.5333738	-0.8593576
H	-4.8100242	-3.9976893	-1.4583813
C	-1.5779707	-1.0061280	2.7612476
C	-1.7414543	0.2592145	3.6329510
C	-1.9699768	-2.2001250	3.6663945
C	-0.0666598	-1.0646148	2.4329990
H	-1.5145144	1.1730641	3.0723278
H	-2.7505947	0.3485431	4.0473698
H	-1.0440615	0.1971920	4.4760955
H	-1.9415962	-3.1503479	3.1291563
H	-1.2918108	-2.2655597	4.5271112
H	-2.9885596	-2.0548703	4.0431868
H	0.5147862	-0.9482303	3.3546439
H	0.2252523	-2.0124286	1.9795245
H	0.2149013	-0.2447844	1.7622302
C	-5.5697314	1.1165403	0.4177037
C	-5.5446116	2.0239693	1.6582092
C	-5.5521333	2.0225816	-0.8345573
C	-6.8962821	0.3217303	0.4253062
H	-5.6190048	1.4402485	2.5821263
H	-4.6236370	2.6178435	1.7022692
H	-6.3925009	2.7177721	1.6269491
H	-5.5148636	1.4324371	-1.7568663
H	-6.4506816	2.6505101	-0.8750629
H	-4.6872034	2.6988793	-0.8148636
H	-7.7590482	1.0004202	0.4380901
H	-6.9744515	-0.3153739	-0.4621594
H	-6.9454659	-0.3229557	1.3093787
C	4.0934497	0.2229572	-2.7707645
C	5.0604068	-0.9448448	-3.0684932
C	2.8840641	-0.0048622	-3.7093079
C	4.8686072	1.5056500	-3.1622010
H	5.9786067	-0.8785570	-2.4754706
H	4.5959127	-1.9207641	-2.8878897
H	5.3393114	-0.9021653	-4.1267589
H	2.1276520	0.7762554	-3.6540188
H	3.2387578	-0.0648424	-4.7452859
H	2.3957806	-0.9574524	-3.4706394
H	5.1465647	1.4595471	-4.2218499
H	4.2982127	2.4215708	-2.9998506
H	5.7867475	1.5754549	-2.5688412
C	2.1328020	2.5991997	1.4600182
C	2.1674226	2.4143388	2.9942338
C	2.9567311	3.8798481	1.1782373
C	0.6403320	2.8254213	1.1167523
H	1.5737330	1.5530507	3.3175855
H	3.1888072	2.2997064	3.3716995
H	1.7375756	3.3081093	3.4589234
H	3.0122884	4.1294080	0.1170629
H	2.5089003	4.7304703	1.7059054
H	3.9806481	3.7484576	1.5447772
H	0.2454927	3.6273445	1.7520663
H	0.4623227	3.1036897	0.0758031
H	0.0653281	1.9171611	1.3262992
C	4.4810074	-1.9076816	1.8016235
C	4.1969225	-1.7114168	3.2996268
C	3.7862218	-3.2111656	1.3460075
C	6.0064485	-2.0592133	1.6106355
H	4.6883124	-0.8122586	3.6869965
H	3.1224337	-1.6300143	3.4971846
H	4.5775808	-2.5727252	3.8591887
H	3.9446172	-3.3939394	0.2775189
H	4.1802125	-4.0715188	1.8990323

H	2.7070512	-3.1603595	1.5388409
H	6.3819187	-2.8935801	2.2146122
H	6.2608973	-2.2564664	0.5644259
H	6.5240769	-1.1451417	1.9204602

dBt : ketene-like adduct of two CO and (**4**)$_2$
106
Energy = -2913.066950132

C	1.9209265	1.9954233	2.1259190
H	1.5488853	2.9612263	1.8128969
H	2.0676814	1.9054136	3.1930327
C	2.6458213	1.1841280	1.2326081
C	2.6743160	1.4386955	-0.2143840
C	3.4133418	0.0139166	1.6722033
C	3.5247913	0.7009789	-1.0329790
C	4.2516314	-0.6516251	0.7750933
C	4.3577277	-0.3370796	-0.5853953
H	3.5584902	0.9461484	-2.0889679
H	4.8540159	-1.4678326	1.1525890
K	-0.6922644	0.5512661	1.9861891
C	-2.5005280	-2.4712377	-0.5697891
H	-2.3846899	-2.7322931	-1.6203990
H	-3.2094688	-3.1880200	-0.1559172
C	-3.0294907	-1.0573686	-0.4283305
C	-2.6986738	-0.0648792	-1.3911791
C	-3.8031099	-0.6898424	0.7140025
C	-3.0370441	1.2722821	-1.1294544
C	-4.1037672	0.6643108	0.9089789
C	-3.7036994	1.6700232	0.0249167
H	-2.7630426	2.0293857	-1.8488187
H	-4.6721203	0.9554021	1.7831366
K	1.7109446	-1.6866492	-0.4243167
C	-1.1415983	-2.6317042	0.1571815
O	-0.5392573	-1.6049327	0.7806599
C	-0.5554009	-3.8282542	0.1621825
O	0.0067914	-4.8861540	0.2473749
C	1.8769035	2.5895542	-0.8724351
C	1.9376943	2.5425960	-2.4152812
C	0.3680920	2.5412217	-0.5359574
C	2.4757140	3.9583359	-0.4652903
H	2.9528455	2.6958761	-2.7947461
H	1.5600875	1.5926382	-2.8108085
H	1.3118370	3.3477692	-2.8163150
H	0.1676185	2.7460099	0.5158398
H	-0.1658908	3.2925752	-1.1282535
H	-0.0536780	1.5619257	-0.7919025
H	1.8683569	4.7766176	-0.8732111
H	2.5354386	4.0758684	0.6184150
H	3.4894174	4.0481941	-0.8709397
C	3.4265639	-0.4643698	3.1435006
C	4.1638861	-1.8104156	3.3188323
C	4.1804318	0.5506087	4.0378332
C	2.0024154	-0.7238628	3.6938481
H	3.7187250	-2.6022198	2.7040399
H	5.2278329	-1.7373707	3.0719021
H	4.0898541	-2.1167214	4.3683849
H	3.7812156	1.5624201	3.9440125
H	4.1226241	0.2483781	5.0914881
H	5.2363647	0.5775673	3.7464881
H	2.0688006	-1.1605330	4.6972449
H	1.4122924	0.1905926	3.7690202
H	1.4694696	-1.4374091	3.0520714
C	5.3003754	-1.0495960	-1.5581668
C	6.1317170	-2.1392726	-0.8628812
C	4.4887016	-1.7171251	-2.6920348
C	6.2722908	-0.0252395	-2.1859228
H	6.7522758	-1.7188874	-0.0639287
H	5.4926591	-2.9161279	-0.4265480
H	6.7949463	-2.6200856	-1.5909815
H	3.8547197	-0.9884751	-3.2091988
H	5.1514940	-2.1772419	-3.4353031
H	3.8463432	-2.5121891	-2.2900015
H	6.9502508	-0.5163848	-2.8957979
H	5.7255327	0.7575063	-2.7221121
H	6.8719084	0.4557680	-1.4057062
C	-2.0479840	-0.3711548	-2.7674973
C	-1.8369030	0.9065850	-3.6116644
C	-0.6439078	-1.0054883	-2.6482911
C	-2.9969719	-1.2609548	-3.6088243
H	-2.7806981	1.4161202	-3.8305298
H	-1.1590960	1.6143970	-3.1241247
H	-1.3853078	0.6163862	-4.5661549
H	-0.6339436	-1.9437960	-2.0895818
H	-0.2462681	-1.1982525	-3.6513192
H	0.0254812	-0.2911562	-2.1542970
H	-2.5468561	-1.4529145	-4.5898533
H	-3.2186237	-2.2239764	-3.1452402
H	-3.9471560	-0.7386838	-3.7630173
C	-4.4028558	-1.7052350	1.7250390
C	-5.1961221	-1.0015445	2.8496386
C	-5.4337960	-2.6074296	1.0006447
C	-3.3379651	-2.5553654	2.4548084

H	-4.5595863	-0.3422054	3.4506269
H	-6.0390155	-0.4192938	2.4630013
H	-5.6007629	-1.7704615	3.5159397
H	-5.0010149	-3.1909884	0.1855990
H	-5.8769049	-3.3071178	1.7190099
H	-6.2362375	-1.9909174	0.5811864
H	-3.8411195	-3.2057853	3.1800314
H	-2.7360164	-3.1797979	1.7951759
H	-2.6481802	-1.9068710	3.0068068
C	-4.0155317	3.1369647	0.3431663
C	-3.2717925	3.5354683	1.6384248
C	-3.5724938	4.0863886	-0.7814336
C	-5.5347309	3.3140564	0.5584331
H	-3.5548067	2.8866363	2.4742822
H	-2.1864191	3.4691658	1.4949269
H	-3.5099873	4.5689568	1.9142642
H	-4.0843981	3.8599046	-1.7231027
H	-3.8155124	5.1176749	-0.5041952
H	-2.4922555	4.0309109	-0.9532736
H	-5.7613479	4.3614667	0.7885415
H	-6.0866353	3.0297392	-0.3438201
H	-5.8976423	2.6992570	1.3880590

dDt : 5-ring from aryl/ketene sites of **dBt**
106
Energy = -2913.079354121

C	3.3220062	-2.1145536	1.3619468
H	3.3547629	-2.3343953	2.4202584
H	3.8322338	-2.8466895	0.7507185
C	3.1950571	-0.7815721	0.9133862
C	2.7945920	0.3146737	1.8009873
C	3.4135976	-0.4054192	-0.4890745
C	2.6129106	1.6026362	1.2927089
C	3.1850649	0.9044684	-0.9015251
C	2.7745352	1.9466762	-0.0545746
H	2.3342764	2.3869025	1.9841006
H	3.3497345	1.1482000	-1.9449588
K	0.7676177	-3.6441859	0.9321599
C	-2.7465132	-1.7058649	-1.8535123
H	-2.3390712	-1.5429457	-2.8480501
H	-3.5973321	-2.3905235	-1.9332727
C	-2.9713296	-0.4715429	-1.0315253
C	-2.7764600	0.8829953	-1.4255386
C	-3.2860349	-0.8794828	0.4065646
C	-2.7623552	1.8519181	-0.4389253
C	-3.0804599	0.2600644	1.3552825
C	-2.8318175	1.5348301	0.9659778
H	-2.6526055	2.8921404	-0.7181325
H	-3.0788725	0.0060299	2.4074104
K	0.4071965	-0.0197887	-0.0381983
C	-1.6323291	-2.1201479	-0.9019100
O	-0.5093353	-2.6041203	-1.2355448
C	-1.9804036	-1.7276485	0.4594194
O	-1.2027323	-1.8799402	1.4456243
C	2.6035700	0.1135093	3.3218972
C	2.1792256	1.4080359	4.0478611
C	1.4762814	-0.9007324	3.6337003
C	3.9419629	-0.3056761	3.9792598
H	2.9270365	2.2019037	3.9471296
H	1.2174809	1.7869236	3.6835695
H	2.0671578	1.1873418	5.1157438
H	1.6452270	-1.8724235	3.1688809
H	1.3964252	-1.0478790	4.7179514
H	0.5128020	-0.5217624	3.2740800
H	3.7862279	-0.5455481	5.0388620
H	4.3974657	-1.1674289	3.4885854
H	4.6500470	0.5284097	3.9175972
C	3.9622542	-1.4007386	-1.5394846
C	4.1007069	-0.7650901	-2.9398275
C	5.3906978	-1.8480887	-1.1417730
C	3.0399246	-2.6263232	-1.7549185
H	3.1332820	-0.4386715	-3.3398787
H	4.7837507	0.0907244	-2.9376740
H	4.5090535	-1.5171355	-3.6243731
H	5.4331327	-2.2367343	-0.1225052
H	5.7507430	-2.6238021	-1.8296683
H	6.0710174	-0.9913810	-1.2047699
H	3.4328956	-3.2389151	-2.5753744
H	2.9699500	-3.2620235	-0.8721254
H	2.0283029	-2.3058905	-2.0277388
C	2.6012178	3.3671449	-0.5991488
C	1.4408632	3.4020519	-1.6187865
C	2.2978360	4.3810584	0.5155638
C	3.8958723	3.8200969	-1.3116654
H	1.6222627	2.7003776	-2.4404050
H	0.4950428	3.1282309	-1.1348399
H	1.3201698	4.4042285	-2.0494352
H	3.1088860	4.4156024	1.2514444
H	2.1884375	5.3822283	0.0829949
H	1.3689285	4.1391846	1.0410137
H	3.7825805	4.8382661	-1.7052241
H	4.7389161	3.8084889	-0.6123523

H	4.1418667	3.1591697	-2.1491120
C	-2.5799087	1.2774486	-2.9033096
C	-2.6198727	2.8059514	-3.0996351
C	-1.1895097	0.8077762	-3.3973721
C	-3.7090702	0.6933322	-3.7824912
H	-3.5687172	3.2312525	-2.7558788
H	-1.8002764	3.3082834	-2.5753697
H	-2.5144914	3.0274271	-4.1670164
H	-1.0248556	-0.2650199	-3.2735668
H	-1.0701995	1.0447237	-4.4607621
H	-0.4028820	1.3407969	-2.8507942
H	-3.5612502	1.0034908	-4.8232473
H	-3.7498042	-0.3965533	-3.7574992
H	-4.6790398	1.0746301	-3.4453139
C	-4.6551187	-1.6485065	0.6624097
C	-5.1739441	-1.3951895	2.0920394
C	-5.7077476	-1.1111959	-0.3222335
C	-4.4911238	-3.1725406	0.5182548
H	-4.4567952	-1.7356404	2.8472235
H	-5.3799942	-0.3339246	2.2656243
H	-6.1075775	-1.9507140	2.2377494
H	-5.4358150	-1.3196235	-1.3618480
H	-6.6840056	-1.5705597	-0.1257409
H	-5.8113451	-0.0255539	-0.2129950
H	-5.4543216	-3.6630655	0.7030283
H	-4.1492356	-3.4780125	-0.4734518
H	-3.7735787	-3.5521831	1.2543727
C	-2.6145911	2.6740289	1.9700782
C	-1.2331384	3.3158503	1.7228347
C	-3.7129826	3.7416677	1.7704613
C	-2.6641868	2.1871675	3.4260876
H	-0.4323242	2.5819696	1.8729826
H	-1.1483023	3.7100639	0.7058662
H	-1.0658435	4.1431117	2.4217629
H	-4.7035398	3.3123828	1.9555406
H	-3.5587056	4.5736859	2.4671351
H	-3.7010274	4.1458541	0.7531068
H	-2.4936827	3.0343687	4.0990967
H	-3.6384554	1.7507737	3.6712201
H	-1.8904888	1.4367808	3.6237763

dTS1t : TS of first CO addition to dimer (**4**)$_2$

104

Energy = -2799.622237993

C	-2.2068977	-1.8903815	3.5559236
H	-3.2033647	-1.5779138	3.8395862
H	-2.0583279	-2.9614597	3.5979259
C	-1.1119364	-1.0217625	3.7479309
C	-1.2828316	0.4140916	4.0064296
C	0.2763105	-1.4821310	3.6735301
C	-0.1746033	1.2037763	4.3035771
C	1.3212038	-0.6126074	3.9951496
C	1.1493355	0.7354481	4.3308025
H	-0.3377620	2.2526224	4.5262198
H	2.3306653	-1.0014367	3.9778510
K	-2.8227250	-1.9609005	0.6965291
C	0.5041859	1.1376196	-1.4425743
H	0.1838277	2.1385174	-1.1797669
H	1.4169652	0.8741190	-0.9187315
C	0.4903660	0.7798260	-2.8484732
C	-0.4789142	1.3090495	-3.7584528
C	1.3641307	-0.2612027	-3.3288594
C	-0.4990808	0.8290246	-5.0730507
C	1.2679786	-0.6916592	-4.6519843
C	0.3489293	-0.1599965	-5.5573368
H	-1.2225179	1.2559567	-5.7616130
H	1.9358130	-1.4684304	-4.9974681
K	0.4364838	0.7189732	1.4073049
C	-1.0268750	-0.0596744	-0.7017980
O	-1.7079800	-0.4573013	-1.5937366
C	-2.6716004	1.0957276	4.0338617
C	-3.4611285	0.8675171	2.7230133
C	-3.4894694	0.6171324	5.2589008
C	-2.5765156	2.6309260	4.1721521
H	-2.8911551	1.2439074	1.8634539
H	-3.6812427	-0.1865327	2.5571166
H	-4.4110884	1.4129685	2.7592819
H	-2.9894777	0.9429179	6.1777991
H	-4.4941239	1.0582008	5.2399120
H	-3.5874183	-0.4690364	5.2989766
H	-3.5903317	3.0462739	4.1591500
H	-2.1071974	2.9346759	5.1132527
H	-2.0176060	3.0830626	3.3436840
C	0.6518255	-2.9421204	3.3210571
C	0.0190895	-3.4154729	1.9867625
C	2.1733273	-3.1235783	3.1202151
C	0.2580808	-3.9004018	4.4714623
H	-1.0476887	-3.6231861	2.0764863
H	0.1729192	-2.6655636	1.2003918
H	0.5052690	-4.3410926	1.6582726
H	2.7403087	-2.9186799	4.0336082

H	2.3678304	-4.1644568	2.8391309
H	2.5593536	-2.4800631	2.3205269
H	0.4646651	-4.9408968	4.1897545
H	0.8483666	-3.6629416	5.3632908
H	-0.7980546	-3.8152618	4.7353240
C	2.2999530	1.6791929	4.6897341
C	3.6513592	0.9490742	4.7422282
C	2.4048299	2.8094936	3.6397299
C	2.0420099	2.3165369	6.0736166
H	3.6417141	0.1470832	5.4884846
H	3.9113470	0.5103048	3.7716541
H	4.4435515	1.6565391	5.0121358
H	1.4546725	3.3466448	3.5405874
H	3.1765653	3.5379008	3.9179714
H	2.6805007	2.4028383	2.6566654
H	2.8583475	2.9981858	6.3437527
H	1.1074802	2.8872754	6.0786404
H	1.9672616	1.5391714	6.8415757
C	-1.5358166	2.4315205	-3.5130282
C	-2.9374580	1.8845223	-3.8863012
C	-1.7012597	3.0305732	-2.1001338
C	-1.1937502	3.6268749	-4.4368496
H	-2.9893497	1.5455185	-4.9241202
H	-3.1879889	1.0384540	-3.2390732
H	-3.6906823	2.6707702	-3.7493486
H	-0.8253388	3.6060732	-1.7859371
H	-2.5477194	3.7273107	-2.1357827
H	-1.9266474	2.2728518	-1.3463981
H	-1.9367236	4.4252362	-4.3131137
H	-0.2071090	4.0308816	-4.1835593
H	-1.1780082	3.3339043	-5.4905286
C	2.4434064	-0.9334642	-2.4404705
C	3.5379170	0.0914661	-2.0441519
C	1.8304960	-1.6161239	-1.1889423
C	3.2008528	-2.0623952	-3.1731742
H	3.1491276	0.9539717	-1.4988397
H	4.3004991	-0.3946429	-1.4225099
H	4.0242689	0.4692617	-2.9500760
H	1.2380807	-2.4861724	-1.4909384
H	2.6253881	-1.9579834	-0.5149670
H	1.1605774	-0.9442241	-0.6520156
H	3.9223975	-2.5056539	-2.4769068
H	2.5243348	-2.8561038	-3.5076211
H	3.7589470	-1.6898649	-4.0386600
C	0.2365350	-0.6257705	-7.0122357
C	1.2577607	-1.7230535	-7.3537348
C	-1.1806541	-1.1877767	-7.2634815
C	0.4763979	0.5704205	-7.9599281
H	2.2862216	-1.3714258	-7.2138682
H	1.1119439	-2.6142541	-6.7329282
H	1.1414214	-2.0192082	-8.4025979
H	-1.9451082	-0.4277643	-7.0711264
H	-1.2833965	-1.5212578	-8.3038523
H	-1.3772812	-2.0391760	-6.6026957
H	0.3830577	0.2555104	-9.0069071
H	-0.2498190	1.3693876	-7.7779283
H	1.4796191	0.9840906	-7.8089840

dTS2t : TS of second CO addition, ketene-like
106
Energy = -2913.039586485

C	-1.4478918	-3.0153044	0.5343726
H	-1.5166474	-3.8516075	-0.1481237
H	-1.0059079	-3.2653729	1.4896369
C	-2.3551033	-1.9411680	0.4332011
C	-3.2155328	-1.7471569	-0.7422307
C	-2.4992258	-0.9246885	1.4799465
C	-4.2548914	-0.8206362	-0.6928530
C	-3.5512476	-0.0080770	1.4217328
C	-4.4853080	0.0574778	0.3785510
H	-4.9322593	-0.7685124	-1.5384791
H	-3.6657051	0.6856922	2.2444823
K	0.8861768	-1.6311724	-0.6106566
C	2.2056805	2.3338827	-1.5104712
H	2.5834920	2.4101468	-2.5320154
H	2.2054451	3.3598614	-1.1396808
C	2.9831857	1.3715176	-0.6638461
C	3.6897711	0.2814290	-1.2597044
C	2.8553477	1.4220601	0.7557639
C	4.1436129	-0.7556641	-0.4342126
C	3.3299388	0.3434894	1.5161147
C	3.9491515	-0.7700462	0.9508003
H	4.6756472	-1.5866255	-0.8807706
H	3.2116681	0.3721334	2.5892697
K	-2.2675336	1.4251348	-1.1046788
C	0.7139146	1.9476037	-1.6862759
O	0.3207505	0.7947117	-1.4801895
C	-0.5697680	3.4712455	-2.1674615
O	0.0898058	4.4222760	-2.4464903
C	-3.0898299	-2.6080018	-2.0222804
C	-3.9421031	-2.0569702	-3.1867567

C	-1.6419477	-2.6301800	-2.5689831
C	-3.5934568	-4.0504585	-1.7728061
H	-5.0152290	-2.1076588	-2.9778478
H	-3.6846417	-1.0172329	-3.4233625
H	-3.7522437	-2.6633094	-4.0793355
H	-0.9535126	-3.1140695	-1.8744425
H	-1.6093691	-3.1818959	-3.5150457
H	-1.2974667	-1.6066898	-2.7690102
H	-3.4518734	-4.6634472	-2.6720828
H	-3.0794175	-4.5341171	-0.9401947
H	-4.6638012	-4.0278572	-1.5395895
C	-1.6096005	-0.8998458	2.7474533
C	-1.8099679	0.3857233	3.5813320
C	-1.9745961	-2.0801339	3.6816358
C	-0.0958806	-0.9293679	2.4285963
H	-1.6178436	1.2890888	2.9909086
H	-2.8182937	0.4553182	4.0006719
H	-1.1060020	0.3740532	4.4209710
H	-1.8992225	-3.0439874	3.1741386
H	-1.3114792	-2.0937903	4.5562784
H	-3.0052621	-1.9629957	4.0336591
H	0.4782364	-0.7757449	3.3488910
H	0.2201278	-1.8810296	1.9997960
H	0.1690209	-0.1176398	1.7403824
C	-5.7220964	0.9610580	0.3998642
C	-5.7014575	1.9385581	1.5863638
C	-5.8323118	1.7905502	-0.8993363
C	-6.9867425	0.0783292	0.5182994
H	-5.7078979	1.4074696	2.5442952
H	-4.8150929	2.5840074	1.5595720
H	-6.5882688	2.5814379	1.5518107
H	-5.8379594	1.1503093	-1.7875898
H	-6.7588291	2.3772551	-0.9042582
H	-4.9995950	2.5003391	-0.9937575
H	-7.8952149	0.6943983	0.5337145
H	-7.0550183	-0.6147344	-0.3272237
H	-6.9501160	-0.5137470	1.4390725
C	4.0512241	0.2166841	-2.7676814
C	4.9507983	-0.9969596	-3.0916349
C	2.8138734	0.0567547	-3.6843265
C	4.8837334	1.4608380	-3.1661117
H	5.8814379	-0.9827516	-2.5151183
H	4.4398918	-1.9491691	-2.9107942
H	5.2126366	-0.9589827	-4.1540555
H	2.1106404	0.8870863	-3.6355294
H	3.1469958	-0.0393829	-4.7239848
H	2.2674801	-0.8592041	-3.4284584
H	5.1526934	1.3980369	-4.2267850
H	4.3622888	2.4062996	-3.0066225
H	5.8073895	1.4886670	-2.5782690
C	2.2889839	2.6479673	1.5236708
C	2.3338004	2.4394917	3.0541171
C	3.1741586	3.8872230	1.2441375
C	0.8074198	2.9574915	1.2006980
H	1.6974246	1.6078722	3.3744146
H	3.3520185	2.2633309	3.4155112
H	1.9613310	3.3491614	3.5362515
H	3.2136635	4.1576566	0.1872486
H	2.7867855	4.7490895	1.7998132
H	4.1990793	3.6944642	1.5785423
H	0.4466905	3.7329277	1.8857510
H	0.6467196	3.3235594	0.1856208
H	0.1904728	2.0653263	1.3486551
C	4.4206622	-1.9714604	1.7792386
C	4.1684108	-1.7779326	3.2831867
C	3.6569143	-3.2346229	1.3204598
C	5.9339658	-2.1920103	1.5612586
H	4.7076734	-0.9076272	3.6725772
H	3.1019327	-1.6486815	3.4984154
H	4.5161243	-2.6623310	3.8274934
H	3.7908005	-3.4148132	0.2480818
H	4.0177809	-4.1183408	1.8585303
H	2.5844760	-3.1359303	1.5313567
H	6.2787032	-3.0479165	2.1528388
H	6.1620958	-2.3930598	0.5097436
H	6.4992916	-1.3062179	1.8695894

dTS4t : TS of 5-ring formation from aryl/ketene
106
Energy = -2913.055730925

C	3.6045965	-2.3013265	0.8748174
H	3.7721667	-2.5496736	1.9139234
H	4.0525370	-3.0032913	0.1849732
C	3.4129784	-0.9560926	0.4895330
C	3.0998963	0.1015304	1.4551590
C	3.4802146	-0.5275154	-0.9138432
C	2.9071871	1.4146519	1.0220594
C	3.2695680	0.8088478	-1.2445697
C	2.9728086	1.8205766	-0.3165201
H	2.6960896	2.1695913	1.7676301
H	3.3432303	1.0964410	-2.2874978

K	0.8959057	-3.6007138	0.7581636
C	-2.7879979	-1.6646022	-1.7715892
H	-2.4569667	-1.4788149	-2.7909333
H	-3.5696876	-2.4303391	-1.8196850
C	-3.1650736	-0.4186646	-1.0109069
C	-2.7525587	0.8850440	-1.4132769
C	-3.6773182	-0.6466576	0.3150980
C	-2.6615786	1.8715668	-0.4352319
C	-3.5059986	0.4042983	1.2575752
C	-2.9446860	1.6221038	0.9265462
H	-2.3445512	2.8684304	-0.7196377
H	-3.8028530	0.2284949	2.2819380
K	0.5418531	0.0287303	-0.2633319
C	-1.5875148	-2.0639037	-0.9309386
O	-0.6165704	-2.8483865	-1.3081091
C	-1.6658476	-1.6801310	0.4048816
O	-0.9749555	-1.4665043	1.3978444
C	3.0470414	-0.1615668	2.9788089
C	2.6462526	1.0925855	3.7852802
C	1.9897966	-1.2267934	3.3602743
C	4.4516949	-0.5578303	3.4974778
H	3.3667417	1.9085765	3.6659240
H	1.6530798	1.4610769	3.5029413
H	2.6154463	0.8283175	4.8484886
H	2.1926129	-2.1958985	2.9029088
H	1.9797023	-1.3629081	4.4485881
H	0.9906759	-0.8998956	3.0515890
H	4.4018771	-0.8336025	4.5586526
H	4.8814709	-1.3918894	2.9396983
H	5.1285979	0.2979874	3.3971759
C	3.8491120	-1.4990580	-2.0606747
C	3.7790739	-0.8254143	-3.4485799
C	5.3134603	-1.9761785	-1.8988588
C	2.8841232	-2.7067436	-2.1625874
H	2.7714412	-0.4551419	-3.6729805
H	4.4842849	0.0072325	-3.5404331
H	4.0408526	-1.5682818	-4.2103422
H	5.5028061	-2.4008950	-0.9110705
H	5.5529048	-2.7320118	-2.6577002
H	5.9911182	-1.1259453	-2.0359289
H	3.1342364	-3.2976791	-3.0519202
H	2.9428597	-3.3673701	-1.2975433
H	1.8452454	-2.3706970	-2.2645844
C	2.7916394	3.2706257	-0.7735715
C	1.5870356	3.3761950	-1.7351552
C	2.5445569	4.2227026	0.4077726

C	4.0589125	3.7478029	-1.5183094
H	1.7146773	2.7180810	-2.6012719
H	0.6591860	3.0931840	-1.2207038
H	1.4622324	4.4018816	-2.1043103
H	3.3867746	4.2155431	1.1082835
H	2.4203809	5.2463874	0.0365935
H	1.6381389	3.9529472	0.9604880
H	3.9409649	4.7844813	-1.8586887
H	4.9305559	3.6947429	-0.8570756
H	4.2608167	3.1231221	-2.3946602
C	-2.4367261	1.2359292	-2.8856246
C	-2.3752335	2.7627822	-3.1035521
C	-1.0569479	0.6824608	-3.3226861
C	-3.5634200	0.7224620	-3.8133819
H	-3.3047809	3.2501302	-2.7913856
H	-1.5404236	3.2253362	-2.5674398
H	-2.2278598	2.9593763	-4.1708035
H	-0.9292901	-0.3849862	-3.1274073
H	-0.9116866	0.8471472	-4.3961471
H	-0.2613821	1.2320553	-2.8055577
H	-3.3459044	1.0143813	-4.8470125
H	-3.6857334	-0.3611089	-3.7874762
H	-4.5172300	1.1751956	-3.5219924
C	-4.7431658	-1.7465756	0.6261717
C	-5.4071650	-1.4946638	1.9971129
C	-5.8626285	-1.6148491	-0.4363213
C	-4.2346057	-3.2023506	0.6717672
H	-4.6911134	-1.5917314	2.8204078
H	-5.8714419	-0.5044906	2.0510656
H	-6.1909343	-2.2450866	2.1467594
H	-5.4987722	-1.8395561	-1.4426187
H	-6.6802382	-2.3075719	-0.2042660
H	-6.2637502	-0.5955970	-0.4413389
H	-5.0863138	-3.8641109	0.8745644
H	-3.7674175	-3.5264137	-0.2587838
H	-3.5032739	-3.3230368	1.4764430
C	-2.6488926	2.7099093	1.9633034
C	-1.1397594	3.0395472	1.9119679
C	-3.4579789	3.9835138	1.6347636
C	-2.9939006	2.2621543	3.3919409
H	-0.5439789	2.1543557	2.1632699
H	-0.8425454	3.3856198	0.9167994
H	-0.8947620	3.8290283	2.6313743
H	-4.5326275	3.7747604	1.6668357
H	-3.2346207	4.7707660	2.3640029
H	-3.2151022	4.3665474	0.6382105

H	-2.7426380	3.0632126	4.0954300
H	-4.0620679	2.0437191	3.4987246
H	-2.4275158	1.3686724	3.6773323

Dt : 5-ring from ketene/aryl sites of **Bt**
55
Energy = -1569.926517680

K	2.4312871	-2.0211966	-4.0207634
C	2.2633486	0.9040193	-0.1796393
H	3.0843338	0.7329769	0.5321741
H	2.3873422	1.9101922	-0.5846001
C	0.8685314	0.5992444	0.2965045
C	0.6363365	-0.9076769	0.3491362
C	-0.1761226	1.5169563	0.5176757
C	-0.8096075	-1.2332122	0.1103364
C	-1.4800398	1.0321214	0.5771925
C	-1.8076767	-0.3212442	0.2495954
H	-1.0272059	-2.2484219	-0.1985153
H	-2.2931790	1.7197794	0.7725186
C	1.1853531	-1.6803450	1.6210315
C	2.7095632	-1.5366880	1.7583065
C	0.5046323	-1.1132410	2.8756753
C	0.8556862	-3.1809712	1.4965110
H	2.9907283	-0.5355736	2.0968426
H	3.0868995	-2.2565315	2.4946854
H	3.2145273	-1.7347107	0.8041555
H	-0.5838211	-1.2105602	2.7980772
H	0.8387788	-1.6490967	3.7731816
H	0.7438275	-0.0508923	2.9975628
H	1.3021038	-3.7285385	2.3355369
H	-0.2244898	-3.3570288	1.5188603
H	1.2499687	-3.5895506	0.5602141
C	0.0744039	3.0327745	0.6657403
C	-1.1484039	3.7584749	1.2642747
C	1.2546099	3.3103859	1.6228187
C	0.3335159	3.6642875	-0.7217313
H	-2.0125070	3.7318007	0.5924851
H	-1.4410874	3.3230237	2.2259097
H	-0.8903267	4.8106491	1.4289611
H	2.1952428	2.8851202	1.2698637
H	1.3924282	4.3925771	1.7352820
H	1.0436164	2.8879944	2.6118912
H	0.4838292	4.7463240	-0.6195003
H	1.2140251	3.2457740	-1.2154054
H	-0.5298802	3.4943891	-1.3742900
C	-3.2872085	-0.6773836	0.0567081
C	-3.8867304	0.1979491	-1.0658866
C	-3.4869675	-2.1525493	-0.3218081
C	-4.0484779	-0.4070573	1.3732861
H	-3.8047269	1.2629502	-0.8263367
H	-3.3598572	0.0218876	-2.0102624
H	-4.9477289	-0.0407900	-1.2090600
H	-3.1054778	-2.8206180	0.4582414
H	-4.5561437	-2.3559788	-0.4508501
H	-2.9787721	-2.3957955	-1.2615108
H	-5.1132325	-0.6387771	1.2487589
H	-3.6500902	-1.0301816	2.1816615
H	-3.9627761	0.6408508	1.6781579
C	2.2269601	-0.0950522	-1.3290639
O	2.8747326	0.0259337	-2.4086241
C	1.3634137	-1.2202117	-0.9856694
O	1.2095990	-2.2435314	-1.7096570

*t*Be : alkene Me$_2$C=CH$_2$ by-product
12
Energy = -157.3223473872

C	-0.0000015	0.1238137	-0.0000133
C	-0.0000011	1.4626367	-0.0000168
C	-1.2764860	-0.6768529	-0.0003710
C	1.2764879	-0.6768473	0.0003650
H	0.9277382	2.0305491	0.0003252
H	-0.9277438	2.0305473	-0.0003001
H	-1.3215214	-1.3323532	-0.8802736
H	-1.3225896	-1.3312780	0.8802856
H	-2.1586831	-0.0305144	-0.0012944
H	1.3215264	-1.3323156	0.8802915
H	1.3225970	-1.3313051	-0.8802670
H	2.1586810	-0.0305033	0.0012647

TS1t : TS of CO addition to benzyl potassium **4**
53
Energy = -1456.503608953

K	0.0575337	2.4719047	0.6242933
C	0.1269871	-0.5957733	2.3603453
H	1.0340656	-1.0128949	2.7839204
H	-0.7324459	-0.9974829	2.8858775
C	0.0418273	-0.6157209	0.9005092
C	1.2328699	-0.5424749	0.0852364
C	-1.2289206	-0.5253390	0.2261368
C	1.1113929	-0.2970468	-1.2854800
C	-1.2637669	-0.2824889	-1.1551912

C	-0.1170728	-0.1374084	-1.9372118
H	2.0119559	-0.2328595	-1.8841707
H	-2.2258959	-0.2101089	-1.6427844
C	2.6556402	-0.7935051	0.6457424
C	3.0883594	0.2843586	1.6685472
C	2.7447211	-2.2153259	1.2539911
C	3.7356032	-0.7557717	-0.4582598
H	3.1089675	1.2694262	1.1851070
H	2.4357458	0.3512686	2.5378211
H	4.1048082	0.0687348	2.0200820
H	2.5763056	-2.9596623	0.4677471
H	3.7459718	-2.3773301	1.6709772
H	2.0121619	-2.3923657	2.0426224
H	4.7058994	-0.9755381	0.0003485
H	3.5549681	-1.5053195	-1.2360087
H	3.8074829	0.2303228	-0.9309751
C	-2.5826564	-0.7533913	0.9452179
C	-2.6258152	-2.1729999	1.5636817
C	-2.8787938	0.3320378	2.0080604
C	-3.7812848	-0.6982503	-0.0280542
H	-2.5606012	-2.9205973	0.7653874
H	-1.8111140	-2.3621516	2.2641566
H	-3.5754373	-2.3177818	2.0927161
H	-2.9470982	1.3156677	1.5262838
H	-3.8475883	0.1280986	2.4804877
H	-2.1255768	0.3949254	2.7921403
H	-4.6964092	-0.8997431	0.5395527
H	-3.8893250	0.2882023	-0.4930468
H	-3.7043792	-1.4520540	-0.8186714
C	-0.1555536	0.1731382	-3.4374215
C	-1.5920072	0.2510013	-3.9795094
C	0.5347638	1.5325551	-3.6925255
C	0.5982701	-0.9257485	-4.2196250
H	-2.1251291	-0.6960771	-3.8402934
H	-2.1657930	1.0446030	-3.4875110
H	-1.5665680	0.4697882	-5.0527913
H	1.5700519	1.5219805	-3.3355253
H	0.5450461	1.7644009	-4.7644524
H	0.0013575	2.3389450	-3.1745787
H	0.5777648	-0.7091859	-5.2945463
H	1.6454296	-0.9907282	-3.9070573
H	0.1327737	-1.9031218	-4.0529019
C	0.1832075	1.0986520	3.1073986
O	0.2534920	0.9856221	4.3079440

TS2t : TS of 2nd CO addition, ketene-like

55
Energy = -1569.886556025

K	-0.0062736	2.4606266	-0.4012029
C	0.1432707	-0.7616395	2.0783408
H	1.0116565	-1.2859673	2.4724139
H	-0.7243341	-1.2090070	2.5623111
C	0.0665783	-0.7643504	0.5922432
C	1.2658259	-0.6474447	-0.1873718
C	-1.1933972	-0.6549603	-0.0770389
C	1.1643212	-0.3455422	-1.5489760
C	-1.2147758	-0.3434847	-1.4467131
C	-0.0588284	-0.1616574	-2.2058933
H	2.0707633	-0.2601285	-2.1353454
H	-2.1700538	-0.2574117	-1.9447637
C	2.6816604	-0.9035317	0.3905722
C	3.0818419	0.1219365	1.4792416
C	2.7798254	-2.3547854	0.9233573
C	3.7788979	-0.7909941	-0.6915808
H	2.4337311	0.1211305	2.3569986
H	4.1040219	-0.0924021	1.8135980
H	3.0721491	1.1358326	1.0615982
H	2.6166931	-3.0606979	0.1015789
H	3.7823876	-2.5289748	1.3314363
H	2.0520733	-2.5802027	1.7042712
H	4.7457108	-1.0101092	-0.2261766
H	3.6300769	-1.5077020	-1.5061253
H	3.8365163	0.2176518	-1.1161585
C	-2.5521116	-0.9675857	0.6054345
C	-2.5369617	-2.4201815	1.1427794
C	-2.9251814	0.0309764	1.7262890
C	-3.7272722	-0.9118888	-0.3961710
H	-2.4026379	-3.1219625	0.3122122
H	-1.7384222	-2.6021953	1.8643607
H	-3.4939558	-2.6419300	1.6295934
H	-2.9328334	1.0539199	1.3338292
H	-3.9359352	-0.2011964	2.0835102
H	-2.2576876	0.0076190	2.5873970
H	-4.6443421	-1.1893921	0.1341534
H	-3.8730524	0.0947417	-0.8044833
H	-3.5956018	-1.6125342	-1.2276380
C	-0.0815246	0.1758251	-3.7012683
C	-1.5120484	0.3026929	-4.2485677
C	0.6504365	1.5154566	-3.9411993
C	0.6450867	-0.9374334	-4.4888346
H	-2.0693990	-0.6336239	-4.1369880
H	-2.0698968	1.0981030	-3.7408115

H	-1.4742531	0.5474491	-5.3155546
H	1.6745642	1.4867623	-3.5545162
H	0.6981956	1.7399341	-5.0129041
H	0.1175280	2.3451211	-3.4578962
H	0.6329560	-0.7114421	-5.5615702
H	1.6888344	-1.0329266	-4.1732346
H	0.1520216	-1.9024592	-4.3311085
C	0.2009621	0.6961789	2.7454369
O	-0.0738674	1.6801404	2.0517891
C	-0.6671161	0.5813601	4.6830543
O	-0.1755286	1.2200492	5.5368714

TS3td : TS of C-C coupling of two carbene
At
106
Energy = -2913.010551214

K	-1.8255138	1.5791647	1.2708577
C	-1.7112563	-1.0999495	-1.2935897
H	-1.1713956	-0.5308568	-2.0487348
H	-1.6418446	-2.1603033	-1.5551533
C	-3.0884307	-0.6170976	-1.0446616
C	-3.5193648	0.6900661	-1.4422593
C	-3.9324114	-1.3476390	-0.1386621
C	-4.5576097	1.3132001	-0.7330232
C	-4.9329025	-0.6577946	0.5493286
C	-5.2259230	0.6933036	0.3278638
H	-4.8669210	2.3075026	-1.0281663
H	-5.5272182	-1.1928875	1.2816599
C	-0.7069893	-1.0438813	0.0664145
O	-1.2820297	-0.9659310	1.1551923
K	1.8435112	-1.5132956	-1.2773905
C	1.7031851	1.0881621	1.3025789
H	1.6355654	2.1380146	1.6038013
H	1.1543051	0.4916297	2.0296997
C	3.0792383	0.6092706	1.0438051
C	3.9285700	1.3631794	0.1625438
C	3.5038193	-0.7113337	1.4030181
C	4.9274938	0.6900385	-0.5438273
C	4.5386321	-1.3181293	0.6750432
C	5.2124709	-0.6690794	-0.3648211
H	5.5267649	1.2447293	-1.2572881
H	4.8434199	-2.3221874	0.9405671
C	0.7103858	1.0855300	-0.0690087
O	1.2920603	1.0236390	-1.1551371
C	-2.9609476	1.4124247	-2.6954959
C	-3.9156484	2.5302095	-3.1732503
C	-1.5992514	2.0907605	-2.4371098
C	-2.8619813	0.4241019	-3.8817088
H	-4.9308423	2.1532666	-3.3386170
H	-3.9648644	3.3642971	-2.4648372
H	-3.5377339	2.9299293	-4.1211913
H	-0.8108568	1.4052214	-2.1260648
H	-1.2676868	2.6082392	-3.3472410
H	-1.7006168	2.8491971	-1.6500566
H	-3.8490880	-0.0000319	-4.0969808
H	-2.5132523	0.9576262	-4.7744428
H	-2.1791054	-0.4041512	-3.6887818
C	-3.9164843	-2.8989833	-0.0625602
C	-5.1196435	-3.4346864	0.7440258
C	-4.0898500	-3.4398958	-1.5037989
C	-2.6676489	-3.5449717	0.5791016
H	-5.0574051	-3.1583781	1.8026761
H	-6.0727651	-3.0722559	0.3444486
H	-5.1210698	-4.5288860	0.6859830
H	-3.2756381	-3.1219571	-2.1599646
H	-4.1168464	-4.5366328	-1.4902355
H	-5.0285944	-3.0741984	-1.9342287
H	-2.8343541	-4.6278006	0.6494389
H	-1.7558120	-3.3802953	0.0039626
H	-2.4958155	-3.1470169	1.5814620
C	-6.2926623	1.3979298	1.1732474
C	-5.8733033	1.3555968	2.6600991
C	-6.4796874	2.8690519	0.7678113
C	-7.6502624	0.6760781	1.0171956
H	-5.7270111	0.3253004	3.0001426
H	-4.9336721	1.9008369	2.8133456
H	-6.6408397	1.8209377	3.2905412
H	-6.8276330	2.9606266	-0.2669742
H	-7.2279298	3.3367959	1.4175083
H	-5.5454938	3.4343940	0.8662295
H	-8.4186762	1.1710068	1.6236879
H	-7.9714847	0.6893987	-0.0299687
H	-7.5827558	-0.3681410	1.3386352
C	3.9184739	2.9159599	0.1337032
C	5.1235746	3.4717472	-0.6561703
C	2.6719845	3.5858847	-0.4877684
C	4.0928938	3.4119125	1.5909066
H	6.0755035	3.0951651	-0.2670053
H	5.0610229	3.2267422	-1.7224733
H	5.1280536	4.5637640	-0.5659917
H	1.7591194	3.4048053	0.0808202
H	2.8417568	4.6699843	-0.5221624

H	2.5000344	3.2215490	-1.5028077
H	4.1242808	4.5084438	1.6108588
H	3.2767407	3.0773714	2.2363239
H	5.0296732	3.0294491	2.0107907
C	2.9529087	-1.4604238	2.6438528
C	3.9121767	-2.5866160	3.0916344
C	2.8596449	-0.4963388	3.8499679
C	1.5896563	-2.1342548	2.3821781
H	3.9569763	-3.4060738	2.3660510
H	4.9282562	-2.2119448	3.2568499
H	3.5417969	-3.0061378	4.0340045
H	2.1723492	0.3328595	3.6773987
H	2.5186465	-1.0486752	4.7342079
H	3.8467486	-0.0730150	4.0668998
H	1.2679175	-2.6746755	3.2824683
H	0.7981568	-1.4408532	2.0970524
H	1.6835353	-2.8731838	1.5758084
C	6.2787960	-1.3518959	-1.2283302
C	6.4508545	-2.8381644	-0.8748373
C	5.8705389	-1.2527569	-2.7155126
C	7.6410236	-0.6473652	-1.0370606
H	6.7931446	-2.9699148	0.1575044
H	5.5120941	-3.3911108	-0.9976107
H	7.1979686	-3.2891761	-1.5374916
H	5.7377449	-0.2096889	-3.0202188
H	6.6377834	-1.7039325	-3.3565152
H	4.9261654	-1.7816981	-2.8943507
H	8.4095376	-1.1255646	-1.6567160
H	7.5834581	0.4085526	-1.3198975
H	7.9553595	-0.7020209	0.0108728

TS3t : TS of ketene/benzyl anion addition
106
Energy = -2913.048709863

C	-3.0464704	-0.7719998	0.1336867
H	-3.3386538	-1.4851690	-0.6296355
H	-3.4079614	-1.0168828	1.1215498
C	-2.8774551	0.5906985	-0.2405326
C	-2.6230677	1.0233487	-1.6079605
C	-2.8531771	1.6643032	0.7491783
C	-1.9545442	2.2329242	-1.8303779
C	-2.1631249	2.8348043	0.4552848
C	-1.6038330	3.1171697	-0.8039578
H	-1.7038656	2.5015725	-2.8494662
H	-2.0756040	3.5910912	1.2287429
K	-2.1419948	-3.8545675	-0.9241147
C	1.2618454	-2.9504302	1.2472163
H	1.1278272	-3.0497879	2.3154404
H	1.5122520	-3.9367299	0.8694420
C	2.3253561	-1.9279205	0.9001082
C	2.5513876	-0.8007952	1.7486222
C	2.9337314	-1.9441858	-0.3966191
C	3.2201083	0.3188090	1.2205931
C	3.5958891	-0.7895599	-0.8394181
C	3.7178482	0.3701984	-0.0782890
H	3.3368261	1.1915501	1.8487723
H	4.0272298	-0.7933067	-1.8350918
K	0.0469090	0.1918177	-0.4750997
C	-0.0826048	-2.5227393	0.5948396
O	-0.1123978	-2.3699533	-0.7272628
C	-1.0213493	-2.1596889	1.5139725
O	-1.5970017	-2.2924856	2.5790001
C	-3.0460339	0.1814939	-2.8307241
C	-2.0973704	-1.0257915	-3.0284542
C	-4.5191395	-0.2699473	-2.6909634
C	-2.9964510	0.9929448	-4.1428358
H	-1.1561093	-0.6886729	-3.4805708
H	-1.8276949	-1.5029492	-2.0832087
H	-2.5390645	-1.7671546	-3.7084379
H	-5.1686747	0.6118681	-2.6561459
H	-4.8062550	-0.8751474	-3.5601116
H	-4.7032443	-0.8491334	-1.7849241
H	-3.3824542	0.3695831	-4.9586414
H	-3.6128171	1.8967090	-4.0791371
H	-1.9758548	1.2855711	-4.4110626
C	-3.6200070	1.5758955	2.0878407
C	-5.0577368	1.0610253	1.8342829
C	-2.9046333	0.6998477	3.1385922
C	-3.7893622	2.9688835	2.7355258
H	-5.5863779	1.7491070	1.1636138
H	-5.0685669	0.0707061	1.3751465
H	-5.6056188	1.0147740	2.7835446
H	-1.9426879	1.1527953	3.4037390
H	-3.5142842	0.6447964	4.0507975
H	-2.7037736	-0.3147296	2.7960646
H	-4.4326307	2.8693011	3.6176770
H	-2.8345739	3.3869319	3.0735932
H	-4.2567440	3.6817304	2.0464297
C	-0.7781113	4.3875568	-1.0172763
C	0.4069568	4.3993042	-0.0246254
C	-0.2098760	4.4749404	-2.4431295
C	-1.6488328	5.6395525	-0.7630119

H	0.0565454	4.3450648	1.0110995
H	1.0660576	3.5408020	-0.2012071
H	1.0037046	5.3131653	-0.1392021
H	-1.0071577	4.5421882	-3.1919420
H	0.4167591	5.3693323	-2.5381967
H	0.4083067	3.6011281	-2.6807354
H	-1.0592578	6.5558576	-0.8956753
H	-2.4929647	5.6643454	-1.4612218
H	-2.0507666	5.6374090	0.2556000
C	2.1085863	-0.5898534	3.2325960
C	0.9714246	0.4569184	3.2518652
C	1.6223657	-1.8076218	4.0478123
C	3.3193096	-0.0444737	4.0396706
H	1.2866189	1.3987553	2.7898319
H	0.0983685	0.0721285	2.7150566
H	0.6657372	0.6686040	4.2836139
H	2.3000005	-2.6630863	3.9576302
H	1.6015472	-1.5128281	5.1034276
H	0.6073408	-2.1115017	3.7853088
H	3.0169617	0.1001023	5.0826741
H	4.1478998	-0.7614155	4.0182153
H	3.6908761	0.9137698	3.6707841
C	3.0272199	-3.1087965	-1.4334434
C	2.3889920	-4.4701977	-1.1021884
C	2.3808224	-2.6565248	-2.7652585
C	4.5341556	-3.4024387	-1.6626209
H	2.7978464	-4.9144416	-0.1887513
H	1.3030801	-4.3859463	-1.0260016
H	2.6237810	-5.1519147	-1.9289827
H	2.8532160	-1.7570216	-3.1711798
H	2.4822309	-3.4538045	-3.5119865
H	1.3197728	-2.4573084	-2.5905930
H	4.6436079	-4.2001978	-2.4066871
H	5.0796552	-2.5260331	-2.0231912
H	5.0052087	-3.7336829	-0.7302109
C	4.3949357	1.6115413	-0.6708447
C	3.6164857	2.0623884	-1.9268250
C	4.4370554	2.7837184	0.3220279
C	5.8443232	1.2635059	-1.0770663
H	3.5765350	1.2666365	-2.6770506
H	2.5885093	2.3409507	-1.6670180
H	4.0965890	2.9367974	-2.3805440
H	5.0135224	2.5299125	1.2180266
H	4.9153857	3.6469458	-0.1530189
H	3.4312702	3.0865905	0.6336057
H	6.3380467	2.1428031	-1.5074653
H	6.4192976	0.9342325	-0.2050545
H	5.8664431	0.4621029	-1.8217575

TS4t : TS of 5-ring from ketene/aryl sites of **Bt**
55
Energy = -1569.908964994

K	-1.3233189	2.1191993	2.6324580
C	0.2442116	-1.5479801	1.6886680
H	1.0726843	-2.2393939	1.8790145
H	-0.6465168	-1.9870105	2.1336690
C	0.0818886	-1.1272645	0.2521910
C	1.2915137	-0.5292799	-0.2905595
C	-1.1674124	-0.9885926	-0.4136942
C	1.1295465	0.2781941	-1.4655987
C	-1.2159032	-0.2057767	-1.5594032
C	-0.0870676	0.4817335	-2.0688556
H	2.0000850	0.7805245	-1.8622793
H	-2.1587997	-0.0914901	-2.0797430
C	2.7034615	-1.1884137	-0.0875453
C	3.3533831	-0.9793133	1.2954625
C	2.5653254	-2.6997271	-0.3913053
C	3.7268525	-0.6352486	-1.1044766
H	2.7529227	-1.3577901	2.1229658
H	4.3194477	-1.4999472	1.3111765
H	3.5327127	0.0853742	1.4723412
H	2.1577151	-2.8465191	-1.3976857
H	3.5489906	-3.1826131	-0.3443014
H	1.9028052	-3.2033522	0.3175873
H	4.6844070	-1.1425234	-0.9431388
H	3.4182278	-0.8175377	-2.1388200
H	3.8915191	0.4398309	-0.9722221
C	-2.4608062	-1.6525812	0.1019079
C	-2.2468592	-3.1631718	0.3546118
C	-2.9650103	-0.9324539	1.3770332
C	-3.6084351	-1.5556058	-0.9250089
H	-1.9899591	-3.6605696	-0.5870307
H	-1.4510905	-3.3734917	1.0699698
H	-3.1736099	-3.6085761	0.7347782
H	-3.2084231	0.1090251	1.1304072
H	-3.8824392	-1.4104570	1.7396940
H	-2.2381632	-0.9393165	2.1925455
H	-4.4760954	-2.0985759	-0.5345187
H	-3.9176995	-0.5199440	-1.1009380
H	-3.3290264	-2.0049493	-1.8837736
C	-0.2708035	1.4083072	-3.2744448

C	-1.2579389	2.5339353	-2.8905576
C	1.0492928	2.0556662	-3.7206231
C	-0.8483839	0.6096125	-4.4635991
H	-2.2304896	2.1251290	-2.5972658
H	-0.8651046	3.1152807	-2.0492365
H	-1.4122935	3.2090349	-3.7407926
H	1.7830673	1.3017482	-4.0265922
H	0.8619574	2.7125437	-4.5772838
H	1.4885062	2.6603855	-2.9196855
H	-0.9990663	1.2713797	-5.3247033
H	-0.1617930	-0.1916849	-4.7579031
H	-1.8126024	0.1562855	-4.2124120
C	0.4276789	-0.1512478	2.2621425
O	0.2405979	0.1697476	3.5018282
C	0.8858865	0.7667621	1.2923672
O	0.8561076	1.9785377	1.0661909

TS5t : TS of tert-butyl cation elimination
106
Energy = -2913.043360244

K	-0.9779808	1.1927537	-3.8741117
C	-1.8242241	2.0358345	0.0242863
H	-1.2950959	2.1488952	0.9891710
H	-2.1979349	3.0334658	-0.2347945
C	-2.9011261	0.9817594	0.0568988
C	-2.3594101	-0.2881370	-0.4047387
C	-4.2661223	1.1152389	0.3392550
C	-3.2958706	-1.2645446	-0.8932978
C	-5.0876424	0.0144465	0.0473991
C	-4.6383157	-1.1572198	-0.6136326
H	-2.8947208	-2.1112150	-1.4393835
H	-6.1435876	0.0813357	0.2823538
C	-1.6347323	-1.2663034	1.2199766
C	-0.2190416	-0.8035344	1.2348737
C	-2.4700603	-0.8011394	2.3924145
C	-1.7548660	-2.7605295	0.9886802
H	-0.1284352	0.2871323	1.1870884
H	0.3338258	-1.1100859	2.1567570
H	0.3217295	-1.2550643	0.3994041
H	-3.5354452	-0.9888427	2.2190792
H	-2.1769180	-1.3518103	3.3018829
H	-2.3389488	0.2670394	2.5865916
H	-1.3126214	-3.3006823	1.8413326
H	-2.8002396	-3.0748061	0.9052769
H	-1.2222392	-3.0728637	0.0848663
C	-4.8931865	2.4105291	0.8889938
C	-6.3327588	2.1868103	1.3932766
C	-4.0823428	2.9528975	2.0851580
C	-4.9631489	3.4704579	-0.2346954
H	-7.0158314	1.9102865	0.5831862
H	-6.3720407	1.4081629	2.1633117
H	-6.7030263	3.1193799	1.8329338
H	-3.0565249	3.2058535	1.8123975
H	-4.5622865	3.8583970	2.4745585
H	-4.0481318	2.2107100	2.8901671
H	-5.4192345	4.3920362	0.1470745
H	-3.9735666	3.7187299	-0.6267757
H	-5.5751079	3.1002309	-1.0646248
C	-5.6636625	-2.2325605	-0.9883337
C	-6.7480733	-1.6232271	-1.9034758
C	-5.0131526	-3.4134153	-1.7253158
C	-6.3276936	-2.7668778	0.3004481
H	-7.2690050	-0.7962454	-1.4101651
H	-6.3004080	-1.2402292	-2.8271992
H	-7.4919440	-2.3842005	-2.1675982
H	-4.2507993	-3.9006854	-1.1076453
H	-5.7783251	-4.1583531	-1.9698925
H	-4.5427080	-3.0911647	-2.6609824
H	-7.0721846	-3.5329736	0.0531445
H	-5.5782124	-3.2132603	0.9632573
H	-6.8327196	-1.9659246	0.8502068
C	-0.9005559	1.4744340	-1.0508442
O	0.0496745	2.1784675	-1.6264522
C	-1.1704837	0.1098498	-1.2626270
O	-0.5307071	-0.6950392	-2.0753384
K	1.7707494	0.2979994	-1.0333003
C	2.4108846	-1.2489183	3.0755634
H	2.0761897	-0.5860194	3.8567429
H	2.2095425	-2.2946452	3.2420577
C	3.2006606	-0.8032324	2.0071770
C	3.5358290	0.6140123	1.8333851
C	3.7177029	-1.7203543	0.9904977
C	4.2872799	1.0115110	0.7327915
C	4.4618586	-1.2207763	-0.0788130
C	4.7660958	0.1346548	-0.2547374
H	4.5339810	2.0621919	0.6308597
H	4.8393422	-1.9225173	-0.8102498
C	3.1376401	1.6946994	2.8641976
C	1.6008833	1.8441018	2.9854514
C	3.7884897	1.3941726	4.2381162
C	3.6439020	3.0996943	2.4722357
H	1.1910023	2.2110722	2.0366932

H	1.0923171	0.9118371	3.2281256
H	1.3625123	2.5833151	3.7599657
H	4.8787247	1.4544291	4.1443267
H	3.4657980	2.1412640	4.9736328
H	3.5416496	0.4016405	4.6174736
H	3.3289637	3.8095532	3.2453471
H	4.7364837	3.1399539	2.4054254
H	3.2212830	3.4365779	1.5187375
C	3.5312232	-3.2520444	1.0833635
C	4.2071683	-3.7979379	2.3671173
C	2.0405028	-3.6637040	1.0114969
C	4.2084083	-4.0029545	-0.0835852
H	5.2887395	-3.6310349	2.3080200
H	3.8447142	-3.3141823	3.2752064
H	4.0308833	-4.8775035	2.4505296
H	1.6343756	-3.4067369	0.0262838
H	1.9502567	-4.7494666	1.1396474
H	1.4196252	-3.1754029	1.7610999
H	4.0382720	-5.0772200	0.0507189
H	3.7867934	-3.7174821	-1.0542397
H	5.2906486	-3.8356028	-0.1089637
C	5.6111343	0.6685805	-1.4149109
C	6.0616648	-0.4497692	-2.3681829
C	4.8000415	1.6990519	-2.2340299
C	6.8718467	1.3673117	-0.8559570
H	6.6824464	-1.1903294	-1.8522111
H	5.2043333	-0.9701175	-2.8112810
H	6.6539620	-0.0211302	-3.1843866
H	4.4097814	2.4994873	-1.5964468
H	5.4249659	2.1579114	-3.0096304
H	3.9548743	1.2161426	-2.7435318
H	7.4917206	1.7543347	-1.6743107
H	6.6033490	2.2063131	-0.2058494
H	7.4689671	0.6612948	-0.2690902

Table S4. TPSS-D3/def2-TZVP + COSMO optimized Cartesian coordinates (in Å) in THF solution for the reactions of very bulky benzyl potassium **11** KBzpt with CO. Each structure is labeled by the specific name (See also **Figure S3** and **Table S1**), followed by the number of atoms, the total energy (in hartrees), and the detailed atomic coordinates (in double-column text list).

11H : arene by-product BzptH
117
Energy = -1909.435733465

C	-0.1114838	0.0172336	-3.0540828
H	0.3554307	0.9138624	-3.4643086
H	0.3790539	-0.8652094	-3.4726192
C	-0.0508912	0.0086354	-1.5438646
C	-0.0238311	1.2175649	-0.8153394
C	-0.0326378	-1.2059250	-0.8216939
C	-0.0023435	1.2005756	0.5826681
C	0.0228059	-1.1903831	0.5765068
C	0.0285669	0.0028006	1.3057085
H	-0.0150567	2.1500084	1.1109633
H	0.0617710	-2.1407812	1.1016190
C	-0.0275335	2.5494779	-1.4851107
C	1.0616890	2.9730550	-2.2537743
C	-1.1082643	3.4174952	-1.3067064
C	1.0813172	4.2351089	-2.8549763
H	1.9105062	2.3031283	-2.3652243
C	-1.1243198	4.6899908	-1.8897446
H	-1.9479757	3.0807961	-0.7043406
C	-0.0228055	5.0758845	-2.6624960
H	-0.0216930	6.0560865	-3.1219852
C	-0.0531435	-2.5339031	-1.4917393
C	0.9218538	-3.4862361	-1.1732688
C	-1.0558715	-2.8776477	-2.4044523
C	0.9163656	-4.7596066	-1.7510822
H	1.7020787	-3.2098831	-0.4687832
C	-1.0952908	-4.1400288	-3.0039615
H	-1.8274884	-2.1479214	-2.6308050
C	-0.0991597	-5.0648244	-2.6658096
H	-0.1161760	-6.0456776	-3.1237258
C	0.0662819	-0.0025272	2.7851703
C	0.7314376	1.0097259	3.4885930
C	-0.5612768	-1.0215201	3.5130839
C	0.7770079	1.0214386	4.8850677
H	1.2418418	1.7862389	2.9256779
C	-0.5336555	-1.0465766	4.9097372
H	-1.1001618	-1.7931380	2.9703597
C	0.1394434	-0.0157149	5.5773408
H	0.1678710	-0.0208126	6.6593979
C	2.2983336	4.6530268	-3.6891696
C	2.1480426	6.0637752	-4.2807928
C	3.5593731	4.6308627	-2.7968696
C	2.4838596	3.6542778	-4.8533538
H	1.2792584	6.1317463	-4.9450038
H	2.0437159	6.8209563	-3.4957694
H	3.0403385	6.3078623	-4.8674506
H	3.7353141	3.6332935	-2.3820753
H	4.4405895	4.9179007	-3.3826409
H	3.4532294	5.3322446	-1.9622194
H	3.3563844	3.9343211	-5.4551580
H	2.6391100	2.6359279	-4.4826464
H	1.6015885	3.6496235	-5.5026919
C	-2.3324041	5.6066171	-1.6639798
C	-3.6049580	4.9146591	-2.2013700
C	-2.4978448	5.8647619	-0.1495129
C	-2.1816763	6.9621622	-2.3728200
H	-3.5136206	4.7202370	-3.2755562
H	-3.7809795	3.9597506	-1.6961424
H	-4.4797878	5.5549348	-2.0374075
H	-1.6079292	6.3573391	0.2572802
H	-3.3652521	6.5108585	0.0299383
H	-2.6491590	4.9295022	0.3985439
H	-3.0687526	7.5743082	-2.1769089
H	-1.3062966	7.5119262	-2.0094239
H	-2.0887510	6.8415607	-3.4578900
C	2.0107272	-5.7641532	-1.3709073
C	3.3915584	-5.1778684	-1.7401505
C	1.9596719	-6.0191014	0.1522732
C	1.8474348	-7.1112450	-2.0929441
H	3.4545324	-4.9888570	-2.8173423
H	3.5746971	-4.2334910	-1.2179622
H	4.1858248	-5.8813916	-1.4638409
H	0.9892592	-6.4372763	0.4411863
H	2.7439373	-6.7282186	0.4422792
H	2.1117721	-5.0930368	0.7156101
H	2.6510477	-7.7882117	-1.7833195

H	0.8920163	-7.5875609	-1.8462549
H	1.9055038	-6.9949526	-3.1808505
C	-2.2182333	-4.4657687	-3.9962011
C	-3.5826800	-4.3335534	-3.2837474
C	-2.1615353	-3.4660733	-5.1727851
C	-2.1048087	-5.8893935	-4.5647298
H	-3.6485224	-5.0337594	-2.4439470
H	-3.7317500	-3.3214524	-2.8943860
H	-4.3965908	-4.5534815	-3.9846132
H	-1.2033495	-3.5428304	-5.6981316
H	-2.9665554	-3.6770735	-5.8865757
H	-2.2762724	-2.4345809	-4.8247150
H	-2.9266941	-6.0659991	-5.2671413
H	-1.1628528	-6.0333949	-5.1055465
H	-2.1692111	-6.6453581	-3.7742712
C	1.5251281	2.1508929	5.6042201
C	3.0091154	2.1331714	5.1753171
C	0.8994583	3.5074760	5.2107754
C	1.4617098	2.0151406	7.1339296
H	3.4778543	1.1807522	5.4447109
H	3.1125629	2.2676343	4.0942070
H	3.5562875	2.9421718	5.6735654
H	-0.1545443	3.5470846	5.5056773
H	1.4299816	4.3273619	5.7092130
H	0.9554651	3.6735449	4.1305463
H	2.0049154	2.8466635	7.5958879
H	0.4291522	2.0445370	7.4988246
H	1.9230416	1.0821772	7.4757206
C	-1.2420908	-2.1839649	5.6560443
C	-2.7464025	-2.1652569	5.3050204
C	-0.6356189	-3.5356248	5.2177476
C	-1.0993671	-2.0624804	7.1815939
H	-3.2019979	-1.2163812	5.6073783
H	-2.9057974	-2.2898478	4.2295400
H	-3.2656097	-2.9800163	5.8234008
H	0.4323870	-3.5757712	5.4569993
H	-1.1381491	-4.3612244	5.7353872
H	-0.7476080	-3.6917686	4.1403519
H	-1.6168083	-2.8992134	7.6633541
H	-0.0491893	-2.0934864	7.4920181
H	-1.5434571	-1.1335348	7.5556664
H	-1.1510985	0.0098272	-3.4066800

11r : radical Bzpt
116
Energy = -1908.793732147

C	-0.0037772	0.0011523	-3.0022287
H	-0.0106878	0.9296688	-3.5593466
H	0.0011134	-0.9269859	-3.5599852
C	-0.0019738	0.0005914	-1.6041865
C	0.1135324	1.2270707	-0.8492550
C	-0.1159142	-1.2263956	-0.8499007
C	0.0936601	1.1984168	0.5379259
C	-0.0960667	-1.1986260	0.5372727
C	-0.0015950	-0.0002555	1.2701512
H	0.2212862	2.1375895	1.0686924
H	-0.2228172	-2.1382415	1.0674631
C	0.2902284	2.5437266	-1.5084605
C	1.3056579	2.7661555	-2.4472107
C	-0.5334020	3.6148457	-1.1408567
C	1.5005143	4.0248882	-3.0230825
H	1.9621348	1.9412328	-2.7084259
C	-0.3664784	4.8886128	-1.6918730
H	-1.3253541	3.4321872	-0.4192818
C	0.6536272	5.0708235	-2.6338426
H	0.7941021	6.0504642	-3.0725632
C	-0.2904893	-2.5430067	-1.5099194
C	0.5376383	-3.6117352	-1.1455964
C	-1.3078608	-2.7678267	-2.4459892
C	0.3736474	-4.8854771	-1.6976217
H	1.3305906	-3.4272342	-0.4256045
C	-1.5005706	-4.0268760	-3.0220627
H	-1.9677905	-1.9448116	-2.7045270
C	-0.6486599	-5.0702303	-2.6367082
H	-0.7873043	-6.0499765	-3.0757774
C	-0.0020170	-0.0003953	2.7431943
C	-0.4656896	1.1118756	3.4638444
C	0.4610800	-1.1126255	3.4642679
C	-0.4714722	1.1316395	4.8601809
H	-0.8520243	1.9662323	2.9157911
C	0.4659457	-1.1321458	4.8606331
H	0.8478209	-1.9670522	2.9166090
C	-0.0029642	-0.0001960	5.5412854
H	-0.0033706	-0.0001364	6.6235771
C	2.6276108	4.2161530	-4.0451832
C	2.6890836	5.6525758	-4.5887304
C	3.9817669	3.8893155	-3.3772031
C	2.4032419	3.2543564	-5.2334187
H	1.7586640	5.9317647	-5.0955865
H	2.8797580	6.3781647	-3.7901535
H	3.5049139	5.7304093	-5.3155746
H	4.0043461	2.8592072	-3.0078123

H	4.7971080	4.0108422	-4.1000105
H	4.1659753	4.5595187	-2.5305391
H	3.2075003	3.3707512	-5.9692613
H	2.3919113	2.2106051	-4.9035276
H	1.4484066	3.4664092	-5.7267380
C	-1.2963400	6.0261124	-1.2530155
C	-2.7558330	5.6523509	-1.5951003
C	-1.1690765	6.2259728	0.2740718
C	-0.9612059	7.3572591	-1.9445427
H	-2.8736367	5.5063333	-2.6742848
H	-3.0583130	4.7283815	-1.0919195
H	-3.4340807	6.4526118	-1.2765579
H	-0.1417832	6.4929061	0.5446987
H	-1.8361797	7.0300816	0.6057307
H	-1.4380110	5.3147948	0.8181928
H	-1.6499039	8.1325549	-1.5917522
H	0.0594038	7.6840471	-1.7162916
H	-1.0661848	7.2836739	-3.0326334
C	1.3083618	-6.0203711	-1.2623500
C	2.7660296	-5.6407796	-1.6057559
C	1.1839253	-6.2234436	0.2645606
C	0.9770542	-7.3514127	-1.9559214
H	2.8819615	-5.4926582	-2.6848570
H	3.0659290	-4.7165751	-1.1014728
H	3.4475157	-6.4391372	-1.2893761
H	0.1579723	-6.4945593	0.5361047
H	1.8543618	-7.0257512	0.5938392
H	1.4503288	-5.3122640	0.8099192
H	1.6693155	-8.1247666	-1.6058559
H	-0.0418886	-7.6825855	-1.7265394
H	1.0798392	-7.2752391	-3.0440447
C	-2.6314575	-4.2216088	-4.0393536
C	-3.9837543	-3.9002003	-3.3649880
C	-2.4159598	-3.2582535	-5.2278981
C	-2.6900727	-5.6578764	-4.5836216
H	-4.1615610	-4.5716111	-2.5179219
H	-4.0084848	-2.8704124	-2.9948587
H	-4.8018982	-4.0243881	-4.0841557
H	-1.4629697	-3.4672588	-5.7261135
H	-3.2232490	-3.3766768	-5.9600713
H	-2.4062953	-2.2146691	-4.8974462
H	-3.5089076	-5.7382248	-5.3067943
H	-1.7609500	-5.9332728	-5.0949208
H	-2.8744521	-6.3847039	-3.7847027
C	-0.9943073	2.3741230	5.5921188
C	-0.1533726	3.6017313	5.1757847
C	-2.4691019	2.6143589	5.1995634
C	-0.9166946	2.2295962	7.1204676
H	0.8990258	3.4590568	5.4440132
H	-0.2084026	3.7750581	4.0960749
H	-0.5225967	4.5000974	5.6838483
H	-3.0896926	1.7580749	5.4849945
H	-2.8529669	3.5062333	5.7081851
H	-2.5741893	2.7660664	4.1204723
H	-1.2962432	3.1432974	7.5903427
H	-1.5241040	1.3905063	7.4773240
H	0.1145015	2.0801251	7.4592212
C	0.9880789	-2.3745707	5.5931691
C	0.1465047	-3.6019347	5.1774114
C	2.4627667	-2.6157733	5.2008129
C	0.9105437	-2.2292405	7.1214456
H	-0.9058007	-3.4586382	5.4456799
H	0.2013566	-3.7756787	4.0977590
H	0.5153386	-4.5002803	5.6857958
H	3.0837490	-1.7595883	5.4856838
H	2.8461972	-3.5074869	5.7100394
H	2.5678334	-2.7682712	4.1218346
H	1.2897751	-3.1428308	7.5917890
H	1.5182572	-1.3901768	7.4778458
H	-0.1205911	-2.0792517	7.4601580

11⁻ : bulky benzyl anion Bzpt⁻
116
Energy = -1908.885696757

C	0.0021678	0.0015595	-3.0841811
H	-0.0200159	0.9246581	-3.6497376
H	0.0189659	-0.9222551	-3.6488288
C	0.0068409	0.0019164	-1.7075475
C	0.0545805	1.2311210	-0.9093834
C	-0.0363386	-1.2277681	-0.9102098
C	0.0368318	1.1950934	0.4722089
C	-0.0173543	-1.1935027	0.4711706
C	0.0088752	0.0005827	1.2370870
H	0.1093806	2.1520259	0.9848237
H	-0.0888384	-2.1511672	0.9825223
C	0.1651932	2.5650977	-1.5420358
C	1.1234439	2.8409128	-2.5291738
C	-0.6507971	3.6230235	-1.1128175
C	1.2755005	4.1170761	-3.0735049
H	1.7646008	2.0278746	-2.8559216
C	-0.5216860	4.9177439	-1.6246857
H	-1.4074749	3.4087857	-0.3619473

C	0.4439902	5.1487718	-2.6119044
H	0.5493351	6.1424973	-3.0295968
C	-0.1479216	-2.5614761	-1.5436837
C	0.6768552	-3.6164632	-1.1247303
C	-1.1176187	-2.8399227	-2.5186551
C	0.5443907	-4.9120134	-1.6339731
H	1.4422686	-3.3994787	-0.3835566
C	-1.2741394	-4.1170846	-3.0593300
H	-1.7649586	-2.0285825	-2.8374007
C	-0.4341463	-5.1462990	-2.6075531
H	-0.5433282	-6.1408987	-3.0221522
C	0.0039226	-0.0001231	2.6925698
C	-0.1640370	1.1892344	3.4465625
C	0.1644195	-1.1906401	3.4463252
C	-0.1691698	1.2059066	4.8400501
H	-0.3066922	2.1257700	2.9154517
C	0.1528510	-1.2099422	4.8397243
H	0.3126362	-2.1263142	2.9152286
C	-0.0125243	-0.0027008	5.5383293
H	-0.0196260	-0.0038114	6.6202081
C	2.3460421	4.3429751	-4.1494973
C	2.3752230	5.7929208	-4.6589200
C	3.7361425	4.0052764	-3.5670282
C	2.0613421	3.4108052	-5.3480821
H	1.4171825	6.0805706	-5.1064872
H	2.6036281	6.4985029	-3.8520905
H	3.1510125	5.8952756	-5.4265144
H	3.7784905	2.9659529	-3.2267525
H	4.5136441	4.1504822	-4.3275103
H	3.9611884	4.6523554	-2.7119350
H	2.8276489	3.5402568	-6.1226065
H	2.0554565	2.3609024	-5.0387649
H	1.0829494	3.6381661	-5.7858809
C	-1.4422339	6.0265651	-1.0979190
C	-2.9138038	5.6425126	-1.3681285
C	-1.2335088	6.1822345	0.4251576
C	-1.1684301	7.3849340	-1.7632486
H	-3.0880910	5.5263373	-2.4435219
H	-3.1717871	4.6977441	-0.8795356
H	-3.5867101	6.4212887	-0.9881294
H	-0.1966455	6.4593464	0.6451323
H	-1.8945966	6.9624318	0.8224339
H	-1.4512500	5.2474568	0.9512903
H	-1.8478998	8.1384246	-1.3486772
H	-0.1409731	7.7206666	-1.5830890
H	-1.3316581	7.3420930	-2.8459613

C	1.4752954	-6.0179101	-1.1194739
C	2.9412344	-5.6329373	-1.4175581
C	1.2942506	-6.1684239	0.4076724
C	1.1912124	-7.3790749	-1.7746936
H	3.0963050	-5.5219057	-2.4964310
H	3.2061929	-4.6852827	-0.9383223
H	3.6220491	-6.4087774	-1.0456636
H	0.2616094	-6.4448897	0.6473738
H	1.9625872	-6.9472333	0.7953965
H	1.5214207	-5.2319646	0.9267033
H	1.8783138	-8.1304139	-1.3688653
H	0.1672540	-7.7147388	-1.5755385
H	1.3357736	-7.3405673	-2.8602143
C	-2.3594829	-4.3471351	-4.1194676
C	-3.7415998	-4.0104649	-3.5176186
C	-2.0937584	-3.4172205	-5.3240463
C	-2.3935084	-5.7982545	-4.6252016
H	-3.9527989	-4.6555786	-2.6575527
H	-3.7807811	-2.9703159	-3.1795551
H	-4.5299135	-4.1591668	-4.2662017
H	-1.1217573	-3.6445185	-5.7758685
H	-2.8712711	-3.5492779	-6.0868704
H	-2.0845155	-2.3665695	-5.0173764
H	-3.1801054	-5.9036120	-5.3812965
H	-1.4415011	-6.0852852	-5.0858491
H	-2.6091420	-6.5024380	-3.8136519
C	-0.3537026	2.5476173	5.5663359
C	0.7899320	3.5076399	5.1707567
C	-1.7028191	3.1766527	5.1540694
C	-0.3446506	2.3948917	7.0962216
H	1.7608348	3.0867684	5.4549956
H	0.8004290	3.6848545	4.0906862
H	0.6687729	4.4739268	5.6763333
H	-2.5327830	2.5134257	5.4215257
H	-1.8472256	4.1373887	5.6639518
H	-1.7438994	3.3531179	4.0745636
H	-0.4783085	3.3780457	7.5621121
H	-1.1568380	1.7444335	7.4395541
H	0.6038941	1.9777625	7.4520978
C	0.3271698	-2.5532631	5.5655773
C	-0.8109248	-3.5122099	5.1516795
C	1.6818395	-3.1815426	5.1709116
C	0.2963627	-2.4041241	7.0955346
H	-1.7857190	-3.0914728	5.4224625
H	-0.8056912	-3.6874372	4.0712578
H	-0.6974917	-4.4794569	5.6572052

H	2.5081383	-2.5195815	5.4524863
H	1.8184641	-4.1439851	5.6797051
H	1.7384143	-3.3545480	4.0915291
H	0.4228403	-3.3884327	7.5609672
H	1.1038311	-1.7548768	7.4520587
H	-0.6569779	-1.9873224	7.4387859

11 : aryl-substituted benzyl potassium
KBzpt
117
Energy = -2508.793472233

K	-2.5984060	-0.2120408	-0.0736687
C	-0.9610328	2.8288656	-0.0191979
H	-1.1520471	3.3431695	-0.9533825
H	-1.2032175	3.3489045	0.8996715
C	-0.3379243	1.5939599	0.0010476
C	-0.0055265	0.8544911	-1.2228714
C	-0.0878368	0.8526003	1.2438732
C	0.2526899	-0.5083136	-1.1750211
C	0.1766112	-0.5093373	1.2139968
C	0.2929286	-1.2656426	0.0215365
H	0.4871026	-1.0027785	-2.1145064
H	0.3027007	-1.0097231	2.1709348
C	-0.0049467	1.5159660	-2.5470079
C	-0.5601631	0.8638915	-3.6660893
C	0.5738271	2.7755456	-2.7409601
C	-0.5314605	1.4323661	-4.9386023
H	-1.0265942	-0.1046862	-3.5170038
C	0.6157818	3.3821080	-4.0019348
H	1.0022358	3.2813472	-1.8808637
C	0.0600931	2.6974744	-5.0870729
H	0.0767156	3.1551537	-6.0704417
C	-0.2011097	1.5131298	2.5642360
C	-0.8598376	0.8708157	3.6305342
C	0.3716550	2.7669601	2.8038225
C	-0.9384844	1.4457278	4.8982695
H	-1.3170173	-0.0960877	3.4451085
C	0.3085646	3.3784935	4.0608021
H	0.8825252	3.2627492	1.9839495
C	-0.3497591	2.7049771	5.0934698
H	-0.4143661	3.1659941	6.0732271
C	0.5238081	-2.7139456	0.0169682
C	0.2961626	-3.4785811	-1.1477685
C	0.9644561	-3.4017211	1.1674313
C	0.5015262	-4.8571338	-1.1818730
H	-0.0704013	-2.9758311	-2.0355165
C	1.1672771	-4.7821643	1.1707873
H	1.1668111	-2.8351161	2.0688341
C	0.9363301	-5.4971844	-0.0127053
H	1.0939568	-6.5722967	-0.0229691
C	-1.1336258	0.7327693	-6.1633812
C	-0.0358160	0.5411135	-7.2332094
C	-1.7186221	-0.6475031	-5.8217941
C	-2.2636824	1.6087120	-6.7483363
H	0.3903524	1.5006737	-7.5422343
H	0.7763599	-0.0827441	-6.8442715
H	-0.4545239	0.0515929	-8.1204909
H	-2.5326637	-0.5709365	-5.0921721
H	-2.1239855	-1.1058271	-6.7303966
H	-0.9542039	-1.3204290	-5.4170508
H	-2.6963887	1.1268473	-7.6331080
H	-3.0589520	1.7555603	-6.0091446
H	-1.8885881	2.5934285	-7.0444466
C	1.2593941	4.7673489	-4.1421451
C	0.5047027	5.7669409	-3.2373764
C	2.7358316	4.6942135	-3.6942083
C	1.2169549	5.2917545	-5.5862776
H	-0.5444366	5.8461756	-3.5430648
H	0.5299604	5.4514391	-2.1893386
H	0.9623912	6.7608905	-3.3078415
H	3.2955543	3.9941843	-4.3241641
H	3.2038408	5.6825888	-3.7735944
H	2.8194702	4.3592951	-2.6555149
H	1.6865787	6.2806696	-5.6281116
H	1.7609385	4.6312223	-6.2706821
H	0.1874734	5.3921493	-5.9478056
C	-1.6479068	0.7572806	6.0708539
C	-2.8050448	1.6516385	6.5677138
C	-2.2280310	-0.6121435	5.6822169
C	-0.6395343	0.5458868	7.2220072
H	-2.4392085	2.6300799	6.8937930
H	-3.5382088	1.8129580	5.7700272
H	-3.3138354	1.1776147	7.4155443
H	-1.4454202	-1.2991866	5.3417935
H	-2.7152990	-1.0629289	6.5536482
H	-2.9780437	-0.5214803	4.8885409
H	-1.1328655	0.0634700	8.0741968
H	0.1901693	-0.0903323	6.8960066
H	-0.2218335	1.4976636	7.5643636
C	0.9575225	4.7545528	4.2544658
C	2.4679037	4.6549839	3.9468503
C	0.3066426	5.7609325	3.2796853

C	0.7888561	5.2869204	5.6862005
H	2.9546127	3.9478587	4.6270347
H	2.6427071	4.3143250	2.9217947
H	2.9438717	5.6354844	4.0669810
H	-0.7651707	5.8576052	3.4844342
H	0.7701035	6.7486893	3.3890427
H	0.4254683	5.4412764	2.2396379
H	1.2659025	6.2696251	5.7686863
H	-0.2679852	5.4035537	5.9506672
H	1.2581717	4.6231677	6.4206643
C	0.2510746	-5.6882441	-2.4470626
C	-0.8377015	-6.7449419	-2.1572147
C	-0.2134647	-4.8294109	-3.6349260
C	1.5574727	-6.4063301	-2.8520518
H	-0.5370811	-7.4098424	-1.3414555
H	-1.7779796	-6.2601800	-1.8718562
H	-1.0190667	-7.3567113	-3.0491791
H	0.5325655	-4.0719362	-3.8999703
H	-0.3679863	-5.4710249	-4.5094622
H	-1.1600909	-4.3210639	-3.4194936
H	1.3917533	-7.0167158	-3.7479051
H	2.3453918	-5.6772735	-3.0704557
H	1.9136035	-7.0628510	-2.0520787
C	1.6388535	-5.5350127	2.4220602
C	0.5850904	-6.5993133	2.8010729
C	2.9819497	-6.2371926	2.1232155
C	1.8389814	-4.6052710	3.6299333
H	-0.3779914	-6.1254217	3.0207052
H	0.4335889	-7.3152923	1.9871885
H	0.9102011	-7.1539253	3.6895162
H	3.7498502	-5.5021560	1.8583645
H	3.3231884	-6.7926435	3.0051179
H	2.8844832	-6.9423092	1.2917177
H	2.1671040	-5.1954291	4.4927576
H	2.6022233	-3.8446063	3.4319355
H	0.9078162	-4.0964752	3.9037935

16H : radical **16** with K replaced by H-atom
118
Energy = -2022.191008502

C	1.1878539	0.4423740	-0.4404644
C	0.0484557	1.2662956	-0.4184798
C	-1.2074970	0.6810742	-0.1891321
C	-1.3571404	-0.6931976	0.0090603
C	-0.2069995	-1.4939691	-0.0204691
C	1.0615267	-0.9475415	-0.2444061
H	-2.0882394	1.3125218	-0.1483032
H	-0.2899986	-2.5633495	0.1502877
C	2.5204663	1.1031807	-0.6497403
H	3.2972674	0.5456955	-0.1042832
H	2.8170535	1.0429760	-1.7145642
C	2.5591487	2.5421117	-0.2406309
O	3.8339544	3.0281365	-0.0901133
H	4.4567183	2.2817933	-0.1127829
C	0.1788511	2.7274225	-0.5584177
C	-0.9257660	3.4763365	-0.9428952
C	1.4380950	3.3855321	-0.2401710
C	-0.9135355	4.8726664	-0.9519777
H	-1.8245924	2.9502674	-1.2495610
C	1.3886377	4.8277073	-0.0332036
C	0.2349704	5.4941165	-0.4386401
H	0.2148148	6.5697745	-0.3368646
C	-2.6935645	-1.2816741	0.2596933
C	-3.0065307	-2.5689125	-0.1947293
C	-3.6730585	-0.5597090	0.9538089
C	-4.2635087	-3.1378189	0.0295069
H	-2.2570964	-3.1222013	-0.7539263
C	-4.9430726	-1.0913309	1.1919085
H	-3.4235046	0.4266402	1.3348240
C	-5.2182476	-2.3820648	0.7219276
H	-6.1969974	-2.8084807	0.9004073
C	2.2353547	-1.8583484	-0.2313708
C	2.3952177	-2.7675279	0.8213861
C	3.1785795	-1.8522185	-1.2649592
C	3.4741657	-3.6557024	0.8617511
H	1.6648453	-2.7604201	1.6261644
C	4.2759818	-2.7186157	-1.2554399
H	3.0365801	-1.1727913	-2.1003789
C	4.4053849	-3.6104953	-0.1835787
H	5.2503992	-4.2867819	-0.1631075
C	2.4841014	5.6679712	0.6625576
C	2.9426164	4.9761538	1.9682626
C	3.6689609	5.9780047	-0.2840755
C	1.9420209	7.0462798	1.1125602
H	2.0893943	4.8774703	2.6493882
H	3.3684437	3.9892839	1.7986154
H	3.7001697	5.5971777	2.4612038
H	3.3108194	6.5737493	-1.1318125
H	4.4209175	6.5705272	0.2522875
H	4.1456847	5.0776045	-0.6665376
H	2.7216993	7.5444459	1.6992819
H	1.7038142	7.7005673	0.2675927

H	1.0515573	6.9466640	1.7424826
C	-2.1391335	5.6491134	-1.4346962
C	-2.4347607	5.2675045	-2.9028296
C	-3.3615831	5.2788216	-0.5630966
C	-1.9343541	7.1708336	-1.3602020
H	-1.5894915	5.5317612	-3.5475091
H	-2.6164771	4.1924901	-3.0036646
H	-3.3244132	5.7993378	-3.2605639
H	-3.1800960	5.5371085	0.4857250
H	-4.2498054	5.8219490	-0.9070690
H	-3.5783247	4.2067771	-0.6165447
H	-2.8289108	7.6773262	-1.7387171
H	-1.7696839	7.5060379	-0.3301206
H	-1.0812199	7.4908695	-1.9687308
C	-5.9748146	-0.2577469	1.9617486
C	-7.3213076	-0.9840164	2.1110340
C	-5.4278194	0.0509327	3.3733830
C	-6.2198741	1.0709579	1.2119173
H	-7.7670063	-1.2108045	1.1361097
H	-7.2143084	-1.9205284	2.6695184
H	-8.0197848	-0.3432399	2.6599882
H	-4.4860414	0.6067456	3.3225074
H	-6.1510638	0.6551862	3.9334004
H	-5.2460325	-0.8759200	3.9280429
H	-6.9493707	1.6813888	1.7566808
H	-5.2962781	1.6510154	1.1158802
H	-6.6086139	0.8812886	0.2056011
C	-4.5540859	-4.5503966	-0.4919469
C	-4.3926597	-4.5707103	-2.0282960
C	-3.5484271	-5.5409048	0.1366130
C	-5.9762361	-5.0213981	-0.1478679
H	-5.0960838	-3.8758391	-2.4996585
H	-3.3793116	-4.2833186	-2.3261501
H	-4.5886417	-5.5781357	-2.4137588
H	-3.6426774	-5.5468420	1.2279197
H	-3.7371615	-6.5550973	-0.2342407
H	-2.5168656	-5.2734135	-0.1138444
H	-6.1267342	-6.0339693	-0.5376523
H	-6.1404630	-5.0496682	0.9350554
H	-6.7356988	-4.3717641	-0.5967712
C	3.6022629	-4.6331374	2.0361300
C	4.8405635	-5.5357406	1.9153443
C	3.7067640	-3.8355977	3.3551570
C	2.3468361	-5.5327935	2.0856961
H	4.8071973	-6.1467345	1.0064083
H	5.7667844	-4.9504420	1.9057075
H	4.8798003	-6.2134134	2.7749630
H	2.8250334	-3.2059617	3.5103693
H	3.7890708	-4.5234045	4.2048706
H	4.5903807	-3.1882145	3.3458912
H	2.4176147	-6.2318061	2.9271341
H	1.4364054	-4.9383827	2.2124146
H	2.2497089	-6.1113805	1.1606950
C	5.2878013	-2.6604045	-2.4059680
C	6.4347845	-3.6683674	-2.2300777
C	4.5664653	-2.9685674	-3.7366939
C	5.8930556	-1.2400907	-2.4744862
H	7.0000341	-3.4793971	-1.3106982
H	6.0645064	-4.6991787	-2.2034933
H	7.1268266	-3.5812682	-3.0746541
H	3.7632312	-2.2504105	-3.9289405
H	5.2767740	-2.9180308	-4.5702041
H	4.1280746	-3.9720703	-3.7143649
H	6.6150211	-1.1767597	-3.2969000
H	5.1179472	-0.4855222	-2.6446707
H	6.4098095	-0.9949937	-1.5400547

16$^{–}$: anion of radical **16** after K^+ elimination
117
Energy = -2021.699979608

C	0.4668015	0.7807699	2.6274113
H	-0.3670872	0.7399094	3.3274824
H	0.8784577	1.8086054	2.6473129
C	0.0763511	0.4663611	1.2194045
C	1.1699127	0.3958767	0.3160737
C	-1.2380929	0.2976353	0.7484668
C	0.9035202	0.3205633	-1.0596644
C	-1.4690885	0.2225428	-0.6327118
C	-0.4052142	0.2661481	-1.5573708
H	1.7318906	0.2179156	-1.7553085
H	-2.4912176	0.1171723	-0.9854166
C	2.5144542	0.2597160	0.8725489
C	3.6270108	0.5172257	0.0651198
C	2.6844156	-0.3009145	2.2136270
C	4.9174594	0.1756464	0.4464020
H	3.4604516	0.9926768	-0.8995928
C	3.9989797	-0.8399171	2.5142112
C	5.0496292	-0.5508272	1.6519542
H	6.0356175	-0.9105767	1.9123249
C	-2.3845280	0.1506101	1.6776453
C	-3.5937021	0.8226064	1.4666372
C	-2.2841320	-0.7229734	2.7680970

C	-4.6892470	0.6391428	2.3172002
H	-3.6664932	1.5062550	0.6242735
C	-3.3508958	-0.9227099	3.6461525
H	-1.3491448	-1.2546530	2.9193207
C	-4.5472075	-0.2337738	3.4038786
H	-5.3847271	-0.3809143	4.0745148
C	-0.6527457	0.1818184	-3.0123693
C	0.2353101	0.7694728	-3.9279441
C	-1.7799610	-0.4803926	-3.5250534
C	0.0286030	0.7002364	-5.3074322
H	1.0941233	1.3107120	-3.5403719
C	-2.0262071	-0.5631040	-4.8981938
H	-2.4612452	-0.9611071	-2.8287811
C	-1.1105052	0.0307618	-5.7770691
H	-1.2871249	-0.0274488	-6.8434021
C	6.1076965	0.5255003	-0.4508193
C	5.9361657	-0.1445255	-1.8335486
C	6.1724235	2.0571623	-0.6446611
C	7.4477145	0.0608850	0.1433932
H	5.8916003	-1.2339689	-1.7272050
H	5.0116298	0.1850980	-2.3190878
H	6.7767609	0.1086991	-2.4926593
H	6.3133339	2.5589957	0.3191186
H	7.0061812	2.3286404	-1.3054437
H	5.2455367	2.4346250	-1.0887555
H	8.2674094	0.3588067	-0.5211623
H	7.6259921	0.5107244	1.1265979
H	7.4821080	-1.0287474	0.2542781
C	4.3046100	-1.7321732	3.7356161
C	4.2507770	-0.9155554	5.0458501
C	3.3097057	-2.9144352	3.7814196
C	5.7137145	-2.3608348	3.6680810
H	4.9952210	-0.1105124	5.0101010
H	3.2543559	-0.4957937	5.1892150
H	4.4872808	-1.5651565	5.8999828
H	3.3825885	-3.4944507	2.8527842
H	3.5631663	-3.5763264	4.6209182
H	2.2886871	-2.5539334	3.9109084
H	5.8222174	-3.0537295	4.5110616
H	5.8651556	-2.9259940	2.7414397
H	6.5101394	-1.6123039	3.7522492
C	-5.9936946	1.3960036	2.0364596
C	-5.7225602	2.9162277	2.0776650
C	-6.5127077	1.0133066	0.6322615
C	-7.0930798	1.0735616	3.0613270
H	-5.3552655	3.2145742	3.0656859
H	-4.9722432	3.2051792	1.3350442
H	-6.6447624	3.4714043	1.8670831
H	-6.7149929	-0.0618919	0.5760740
H	-7.4408709	1.5542312	0.4109583
H	-5.7798387	1.2607768	-0.1421644
H	-7.9995288	1.6363994	2.8115598
H	-7.3460230	0.0074290	3.0579849
H	-6.7913745	1.3535196	4.0767746
C	-3.1659291	-1.8767812	4.8319570
C	-2.8007551	-3.2824290	4.3050990
C	-2.0125489	-1.3577893	5.7206513
C	-4.4346506	-1.9959070	5.6911584
H	-3.5990152	-3.6755021	3.6654489
H	-1.8755974	-3.2567505	3.7215508
H	-2.6551509	-3.9725906	5.1449711
H	-2.2577251	-0.3665995	6.1200413
H	-1.8572931	-2.0406515	6.5654870
H	-1.0721480	-1.2776659	5.1656926
H	-4.2485287	-2.6872819	6.5207129
H	-4.7234367	-1.0286063	6.1178100
H	-5.2804972	-2.3837107	5.1116452
C	1.0379010	1.3660337	-6.2520853
C	1.0933926	2.8809297	-5.9543637
C	2.4370588	0.7535736	-6.0189372
C	0.6694942	1.1771669	-7.7325493
H	0.1124222	3.3413349	-6.1149855
H	1.3920427	3.0708511	-4.9185936
H	1.8195213	3.3697217	-6.6150117
H	2.4267650	-0.3218972	-6.2268295
H	3.1725675	1.2281605	-6.6793889
H	2.7663928	0.8958124	-4.9846733
H	1.4244825	1.6640454	-8.3597603
H	0.6358314	0.1167588	-8.0065657
H	-0.3026079	1.6255811	-7.9661506
C	-3.2736586	-1.3070934	-5.3930075
C	-3.2130250	-2.7769952	-4.9212781
C	-4.5346151	-0.6381072	-4.8019629
C	-3.3949717	-1.2965120	-6.9254186
H	-2.3260522	-3.2756688	-5.3270079
H	-3.1694131	-2.8424923	-3.8295840
H	-4.1032438	-3.3197640	-5.2612605
H	-4.6052201	0.4058755	-5.1264319
H	-5.4352985	-1.1672491	-5.1355609
H	-4.5156270	-0.6525235	-3.7076296
H	-4.3013556	-1.8361426	-7.2216868
H	-3.4671236	-0.2754793	-7.3165205

H -2.5391409 -1.7889187 -7.4003778
C 1.5602292 -0.1786568 3.1120707
O 1.3905042 -0.7011921 4.2574658

16 : bulky radical after first CO addition to **11**
118
Energy = -2621.605676088
K 3.8288640 1.8231121 4.1992907
C 0.4508890 0.3001771 2.7991699
H -0.2569310 -0.2938402 3.3912846
H 0.2917502 1.3376624 3.1479517
C 0.1385128 0.1962379 1.3304439
C 1.2070774 0.2039409 0.4073829
C -1.1856528 0.0932014 0.8530947
C 0.9273401 0.1293321 -0.9672510
C -1.4260367 0.0050641 -0.5228330
C -0.3773058 0.0304246 -1.4544055
H 1.7518985 0.1251303 -1.6728145
H -2.4514090 -0.0985454 -0.8663861
C 2.5959325 0.1924588 0.8863951
C 3.6453382 0.5782229 0.0605398
C 2.8727878 -0.2521001 2.2419625
C 4.9900333 0.4553367 0.4461760
H 3.4119997 0.9945837 -0.9160355
C 4.2445846 -0.6890944 2.5113927
C 5.2401355 -0.2604298 1.6347833
H 6.2649203 -0.5404119 1.8495920
C -2.3601171 0.0443865 1.7642594
C -3.3069307 -0.9767505 1.6198963
C -2.5709318 1.0191761 2.7447669
C -4.4426043 -1.0423641 2.4325766
H -3.1334104 -1.7373797 0.8631917
C -3.6909831 0.9854321 3.5799758
H -1.8510588 1.8259046 2.8416004
C -4.6139926 -0.0535124 3.4089883
H -5.4851278 -0.0934044 4.0505015
C -0.6413205 -0.0702344 -2.9085398
C 0.2755927 -0.6995317 -3.7615068
C -1.8130887 0.4595651 -3.4655002
C 0.0496380 -0.8003515 -5.1365684
H 1.1707464 -1.1395280 -3.3309696
C -2.0793561 0.3711325 -4.8350271
H -2.5177172 0.9664832 -2.8118556
C -1.1343321 -0.2593305 -5.6548150
H -1.3255622 -0.3333375 -6.7176729
C 6.1005896 0.9839110 -0.4630127
C 6.0268017 0.2918839 -1.8440305
C 5.9180234 2.5065572 -0.6578347
C 7.5005416 0.7389072 0.1232984
H 6.1572998 -0.7904249 -1.7376533
H 5.0597988 0.4701644 -2.3257234
H 6.8123478 0.6749821 -2.5071010
H 5.9946846 3.0286726 0.3030373
H 6.6885486 2.9039704 -1.3302389
H 4.9372342 2.7332853 -1.0889473
H 8.2576108 1.1703736 -0.5412028
H 7.6081621 1.2058917 1.1091500
H 7.7138271 -0.3310496 0.2247958
C 4.6146325 -1.7400357 3.5886753
C 4.9421418 -1.1246604 4.9697636
C 3.4691668 -2.7615515 3.7469174
C 5.8647987 -2.5418073 3.1551929
H 5.7508639 -0.3856619 4.8810729
H 4.0492807 -0.6650612 5.4009710
H 5.2918619 -1.9062018 5.6558966
H 3.2367968 -3.2244550 2.7814655
H 3.7802641 -3.5512951 4.4423634
H 2.5690055 -2.2852857 4.1369590
H 6.0105384 -3.3734111 3.8543397
H 5.7436289 -2.9514661 2.1469453
H 6.7775619 -1.9366492 3.1748066
C -5.4464399 -2.1844000 2.2330147
C -5.9899765 -2.1366983 0.7875811
C -4.7339798 -3.5356469 2.4627503
C -6.6372782 -2.0940634 3.2004419
H -6.5031186 -1.1876808 0.5988682
H -5.1829426 -2.2373413 0.0552701
H -6.7020564 -2.9542225 0.6240134
H -4.3415330 -3.5966276 3.4833242
H -5.4361491 -4.3639658 2.3109400
H -3.8972296 -3.6669844 1.7698738
H -7.3232758 -2.9272205 3.0124630
H -6.3136684 -2.1553818 4.2452437
H -7.1963744 -1.1616579 3.0655590
C -3.8642081 2.0748456 4.6452766
C -2.6534679 2.0389975 5.6046006
C -3.9280086 3.4571572 3.9589723
C -5.1449225 1.8867243 5.4737245
H -2.5899847 1.0697102 6.1102974
H -1.7137624 2.1992756 5.0668383
H -2.7503503 2.8222115 6.3658304

H	-4.7802309	3.5092113	3.2730550
H	-4.0391772	4.2477110	4.7105891
H	-3.0185222	3.6592213	3.3848557
H	-5.2183800	2.6871559	6.2180484
H	-6.0413633	1.9291853	4.8453736
H	-5.1422341	0.9305831	6.0084112
C	1.0869346	-1.5066941	-6.0185768
C	2.4490992	-0.7906380	-5.8790236
C	1.2359467	-2.9726119	-5.5538787
C	0.6911902	-1.5069321	-7.5039448
H	2.3727140	0.2524250	-6.2047217
H	2.7985597	-0.7980048	-4.8415175
H	3.2035374	-1.2926635	-6.4959661
H	0.2824831	-3.5043706	-5.6433253
H	1.9814214	-3.4892175	-6.1698531
H	1.5589919	-3.0277936	-4.5094472
H	1.4677209	-2.0149458	-8.0861199
H	-0.2535851	-2.0365507	-7.6691242
H	0.5897948	-0.4879870	-7.8939458
C	-3.3805641	0.9653353	-5.3887208
C	-4.5839076	0.2822379	-4.7010658
C	-3.4192417	2.4804506	-5.0894946
C	-3.5159073	0.7685682	-6.9072232
H	-4.5810787	-0.7950946	-4.8989432
H	-4.5576439	0.4281017	-3.6165114
H	-5.5228714	0.7029535	-5.0795701
H	-2.5772072	2.9901695	-5.5703333
H	-4.3509531	2.9158486	-5.4694821
H	-3.3660406	2.6754168	-4.0136575
H	-4.4611736	1.2059224	-7.2466061
H	-2.7025567	1.2610754	-7.4516420
H	-3.5199760	-0.2933590	-7.1770182
C	1.8568638	-0.0895450	3.2208921
O	2.0680679	-0.1085764	4.4957163

17 : 6-ring ketone product after 1st CO addition
117
Energy = -2021.632127700

C	0.4791111	-0.3949338	2.6986876
H	-0.3352571	-0.8650859	3.2527644
H	0.6787732	0.5892029	3.1519136
C	0.1365905	-0.2405044	1.2361118
C	1.1992109	-0.2328170	0.3113740
C	-1.1942994	-0.1851022	0.7783847
C	0.9141689	-0.2137890	-1.0594264
C	-1.4395600	-0.1651172	-0.5999199
C	-0.3999644	-0.1872390	-1.5374520
H	1.7272541	-0.2240722	-1.7769405
H	-2.4706183	-0.1491687	-0.9412658
C	2.6002269	-0.2363864	0.7995626
C	3.6280610	0.1972257	-0.0399407
C	2.9141732	-0.7018492	2.1078601
C	4.9592504	0.1892452	0.3705967
H	3.3789807	0.5665916	-1.0288449
C	4.2554794	-0.6736049	2.5723527
C	5.2357656	-0.2332858	1.6736896
H	6.2613528	-0.1984459	2.0110757
C	-2.3530333	-0.1699964	1.7060931
C	-3.4308635	-1.0373539	1.4937690
C	-2.4070815	0.7252152	2.7799703
C	-4.5483333	-1.0297981	2.3338669
H	-3.3772134	-1.7386394	0.6651692
C	-3.5044992	0.7583282	3.6453309
H	-1.5849228	1.4204651	2.9238645
C	-4.5622671	-0.1274529	3.4050242
H	-5.4180658	-0.1136238	4.0677395
C	-0.6785103	-0.1836723	-2.9919646
C	0.1676780	-0.8573959	-3.8817490
C	-1.7911030	0.4934996	-3.5066755
C	-0.0720815	-0.8606308	-5.2581774
H	1.0138499	-1.4098477	-3.4825457
C	-2.0681496	0.5077674	-4.8764589
H	-2.4354466	1.0376510	-2.8216209
C	-1.1954088	-0.1729107	-5.7349712
H	-1.3954285	-0.1679499	-6.7987358
C	6.0520012	0.6617289	-0.5924968
C	6.0177027	-0.2183973	-1.8627931
C	5.7821661	2.1315803	-0.9856816
C	7.4571150	0.5707058	0.0237638
H	6.2027166	-1.2684500	-1.6126223
H	5.0479767	-0.1537651	-2.3669789
H	6.7894283	0.1122512	-2.5668993
H	5.8033339	2.7787919	-0.1023854
H	6.5497586	2.4765795	-1.6875802
H	4.8058029	2.2441055	-1.4678525
H	8.1961336	0.9049931	-0.7119206
H	7.5521445	1.2094107	0.9087391
H	7.7064623	-0.4574458	0.3083679
C	4.6914731	-1.0241965	4.0118667
C	3.7593355	-0.3638877	5.0533127
C	4.7363982	-2.5581489	4.2050202

C	6.1100065	-0.4938469	4.3192877
H	3.7270141	0.7208647	4.9017947
H	2.7422980	-0.7536072	5.0218829
H	4.1527846	-0.5542925	6.0579960
H	5.4449365	-3.0044167	3.4977589
H	5.0802779	-2.7868243	5.2210848
H	3.7540198	-3.0038842	4.0504806
H	6.3285683	-0.6895738	5.3740947
H	6.8804974	-0.9993843	3.7280607
H	6.1883055	0.5857731	4.1496644
C	-5.7027533	-2.0013048	2.0612923
C	-6.2623730	-1.7402798	0.6448228
C	-5.1768546	-3.4519455	2.1394257
C	-6.8506607	-1.8459045	3.0717082
H	-6.6425749	-0.7163287	0.5622030
H	-5.4914279	-1.8799306	-0.1197384
H	-7.0835751	-2.4342214	0.4310943
H	-4.7738369	-3.6632199	3.1357844
H	-5.9904799	-4.1581831	1.9367308
H	-4.3826595	-3.6272721	1.4068429
H	-7.6468526	-2.5573987	2.8274083
H	-6.5177998	-2.0520967	4.0949560
H	-7.2792949	-0.8379531	3.0429686
C	-3.5113712	1.7529414	4.8119353
C	-2.2859308	1.4838259	5.7139924
C	-3.4251039	3.1913148	4.2550977
C	-4.7803217	1.6398487	5.6717078
H	-2.3208425	0.4673621	6.1207795
H	-1.3498386	1.5957291	5.1575166
H	-2.2711141	2.1923621	6.5503346
H	-4.2855714	3.4089880	3.6131988
H	-3.4147359	3.9139364	5.0794171
H	-2.5149348	3.3344183	3.6642491
H	-4.7295039	2.3665560	6.4897041
H	-5.6829068	1.8527926	5.0882607
H	-4.8804013	0.6421490	6.1134027
C	0.8857121	-1.6168833	-6.1868214
C	2.3117718	-1.0446794	-6.0232764
C	0.8931621	-3.1131054	-5.8018253
C	0.4837425	-1.4982892	-7.6657010
H	2.3352396	0.0178745	-6.2880881
H	2.6656784	-1.1460029	-4.9920687
H	3.0098550	-1.5813886	-6.6760995
H	-0.1077634	-3.5445367	-5.9101273
H	1.5820660	-3.6651886	-6.4517282
H	1.2143780	-3.2559398	-4.7650842
H	1.2024621	-2.0492973	-8.2817893
H	-0.5100657	-1.9218527	-7.8482912
H	0.4828850	-0.4550660	-8.0007632
C	-3.2985136	1.2669748	-5.3876923
C	-4.5674989	0.6784785	-4.7311885
C	-3.1741991	2.7583212	-5.0036987
C	-3.4540913	1.1710033	-6.9139118
H	-4.6800346	-0.3803474	-4.9881151
H	-4.5259931	0.7609383	-3.6403211
H	-5.4561592	1.2172772	-5.0799024
H	-2.2801182	3.2003618	-5.4565659
H	-4.0521589	3.3118324	-5.3566274
H	-3.1051095	2.8861321	-3.9186977
H	-4.3472745	1.7249139	-7.2223896
H	-2.5930061	1.6039972	-7.4349411
H	-3.5717723	0.1324201	-7.2426071
C	1.7374409	-1.2218713	2.8576133
O	1.7299108	-2.2565364	3.5168988

12 : 7-ring radical after two CO addition
120
Energy = -2735.030013105

K	-2.5471612	2.3918589	3.9142641
C	-1.8342627	1.6486647	-0.7459230
H	-2.5598376	1.2331360	-1.4556611
H	-1.5566588	2.6574468	-1.0507141
C	-0.6340242	0.7394921	-0.6417451
C	-0.8522295	-0.6457823	-0.4759665
C	0.6823489	1.2394124	-0.5770374
C	0.2438462	-1.5014480	-0.3177710
C	1.7539877	0.3614594	-0.4032339
C	1.5587182	-1.0223637	-0.2845635
H	0.0617077	-2.5670915	-0.2112584
H	2.7573594	0.7725826	-0.3317679
C	-2.2221091	-1.2174782	-0.4953094
C	-2.4145431	-2.3785254	-1.2605111
C	-3.2927470	-0.6585675	0.2510929
C	-3.6490411	-3.0094998	-1.3269749
H	-1.5659261	-2.7677098	-1.8132861
C	-4.5856199	-1.2542177	0.1374154
C	-4.7102582	-2.4108719	-0.6345878
H	-5.6875741	-2.8701911	-0.7228872
C	0.9423386	2.7064137	-0.6052236
C	1.2684380	3.3573348	0.5863057
C	0.8329107	3.4528134	-1.7808279
C	1.4618953	4.7407395	0.6300310

H	1.3384530	2.7638213	1.4935699
C	1.0341944	4.8377133	-1.7803405
H	0.5796596	2.9367654	-2.7033764
C	1.3424673	5.4616060	-0.5644805
H	1.4873390	6.5345090	-0.5474796
C	2.7034046	-1.9446328	-0.1147856
C	2.5814077	-3.1081278	0.6563178
C	3.9342397	-1.6772203	-0.7286299
C	3.6488094	-3.9949504	0.8176699
H	1.6371417	-3.3043789	1.1565424
C	5.0259144	-2.5377300	-0.5869727
H	4.0242386	-0.7887932	-1.3477936
C	4.8633549	-3.6921609	0.1891265
H	5.6995154	-4.3694552	0.3061269
C	-3.8925004	-4.2881503	-2.1336562
C	-4.9579123	-4.0202371	-3.2198984
C	-2.6138195	-4.7928562	-2.8211922
C	-4.4048968	-5.3948187	-1.1849268
H	-5.9042126	-3.6943733	-2.7763345
H	-4.6179783	-3.2390766	-3.9085332
H	-5.1477118	-4.9333755	-3.7966808
H	-1.8284219	-5.0172912	-2.0907640
H	-2.8348196	-5.7128531	-3.3734126
H	-2.2223556	-4.0577159	-3.5329912
H	-4.5958435	-6.3167574	-1.7470760
H	-3.6630677	-5.6088686	-0.4077691
H	-5.3359373	-5.0956764	-0.6927631
C	-5.8693839	-0.6722567	0.7752173
C	-5.9787225	-1.1021809	2.2560957
C	-5.9194757	0.8685907	0.6667347
C	-7.1382564	-1.1875557	0.0571703
H	-6.0167177	-2.1957502	2.3285263
H	-5.1176754	-0.7339479	2.8163479
H	-6.9026004	-0.6988609	2.6914251
H	-5.7783446	1.1867033	-0.3725962
H	-6.9054266	1.2162272	0.9985724
H	-5.1642863	1.3538872	1.2844193
H	-8.0083024	-0.6674910	0.4728385
H	-7.1024941	-0.9905481	-1.0202440
H	-7.2989672	-2.2597156	0.2097281
C	1.7778784	5.4100719	1.9725028
C	0.6643827	5.0646782	2.9883460
C	3.1264757	4.8736063	2.5013722
C	1.8702727	6.9399765	1.8587063
H	-0.3120905	5.3933863	2.6169896
H	0.6150326	3.9848450	3.1633009
H	0.8625993	5.5589643	3.9468030
H	3.9361907	5.1138160	1.8039865
H	3.3597372	5.3240509	3.4737200
H	3.0959503	3.7862397	2.6243347
H	2.0845519	7.3665339	2.8448378
H	2.6735752	7.2452048	1.1791220
H	0.9303433	7.3735221	1.4992236
C	0.8971200	5.6178704	-3.0933262
C	1.9136245	5.0718488	-4.1204895
C	-0.5331700	5.4299307	-3.6470207
C	1.1523695	7.1227659	-2.9123117
H	2.9380329	5.1953399	-3.7524062
H	1.7482914	4.0074805	-4.3146084
H	1.8179148	5.6123160	-5.0697653
H	-1.2746008	5.8085292	-2.9350672
H	-0.6472753	5.9752353	-4.5914080
H	-0.7505928	4.3735514	-3.8337941
H	1.0444669	7.6279707	-3.8783890
H	0.4359963	7.5722891	-2.2156732
H	2.1647807	7.3177341	-2.5411903
C	3.4595617	-5.2511468	1.6773334
C	3.0943983	-4.8321478	3.1186948
C	2.3114590	-6.1020209	1.0902645
C	4.7259138	-6.1206313	1.7319976
H	3.8945530	-4.2281502	3.5595462
H	2.1733026	-4.2416019	3.1399381
H	2.9450998	-5.7200831	3.7443829
H	2.5454118	-6.4130543	0.0665393
H	2.1571377	-7.0006500	1.6991000
H	1.3718166	-5.5413388	1.0677360
H	4.5346734	-7.0036796	2.3513128
H	5.0206193	-6.4666310	0.7351710
H	5.5691483	-5.5778646	2.1729667
C	6.3467912	-2.1997840	-1.2896934
C	6.1101510	-2.1329010	-2.8148568
C	6.8509685	-0.8261948	-0.7942545
C	7.4409158	-3.2438807	-1.0153327
H	5.7531267	-3.0966869	-3.1930219
H	5.3652331	-1.3724543	-3.0687406
H	7.0439130	-1.8818181	-3.3318532
H	7.0279135	-0.8463099	0.2863250
H	7.7909682	-0.5651466	-1.2947787
H	6.1235155	-0.0359590	-1.0039059
H	8.3605276	-2.9524483	-1.5343784
H	7.6677597	-3.3185183	0.0538301
H	7.1514479	-4.2363397	-1.3776926

C	-2.4182018	1.6965451	0.6598707
O	-2.2777225	2.7369554	1.3744725
C	-3.0043238	0.4772222	1.1810149
O	-3.3037839	0.3899013	2.4267242

14a : more stable 7-ring diketone isomer of **14**

119

Energy = -2135.007124610

C	-1.9330036	1.6585420	-0.6460401
H	-2.6208858	1.2781377	-1.4123761
H	-1.6626794	2.6868924	-0.8794880
C	-0.7260111	0.7517537	-0.5291969
C	-0.9213576	-0.6424197	-0.4308314
C	0.5813386	1.2715993	-0.4268185
C	0.1768861	-1.4885825	-0.2501948
C	1.6545110	0.3982690	-0.2252819
C	1.4780685	-0.9880039	-0.1350335
H	0.0066651	-2.5600792	-0.2007148
H	2.6509577	0.8203610	-0.1308789
C	-2.2734673	-1.2488292	-0.5642749
C	-2.4377320	-2.3144143	-1.4566531
C	-3.3638368	-0.8165737	0.2196567
C	-3.6719917	-2.9378325	-1.6161847
H	-1.5772617	-2.6308112	-2.0358591
C	-4.6417941	-1.4109705	0.0626446
C	-4.7476929	-2.4549060	-0.8586481
H	-5.7163662	-2.9174981	-1.0025227
C	0.8722320	2.7268906	-0.5225204
C	1.5257524	3.3746110	0.5296969
C	0.5380649	3.4574114	-1.6678277
C	1.8362471	4.7368920	0.4633975
H	1.7757740	2.7985924	1.4166199
C	0.8304419	4.8206353	-1.7699756
H	0.0568199	2.9402799	-2.4938748
C	1.4767075	5.4398390	-0.6925940
H	1.7067796	6.4956001	-0.7566340
C	2.6292146	-1.8938335	0.0724804
C	2.4900566	-3.0621999	0.8320631
C	3.8786597	-1.6003532	-0.4878602
C	3.5647775	-3.9311123	1.0372527
H	1.5283948	-3.2756055	1.2903988
C	4.9768744	-2.4442649	-0.3037645
H	3.9798529	-0.7085391	-1.1000769
C	4.7988351	-3.6038480	0.4612347
H	5.6405346	-4.2675225	0.6117875
C	-3.8938933	-4.0985248	-2.5887897
C	-4.9433276	-3.6833997	-3.6445551
C	-2.6016875	-4.5017564	-3.3164344
C	-4.4176464	-5.3223810	-1.8040998
H	-5.8983076	-3.4238642	-3.1768927
H	-4.5949697	-2.8166087	-4.2163195
H	-5.1186931	-4.5110163	-4.3410973
H	-1.8267930	-4.8230526	-2.6113608
H	-2.8110389	-5.3394843	-3.9896193
H	-2.2029509	-3.6780993	-3.9187332
H	-4.5887839	-6.1591320	-2.4905788
H	-3.6910602	-5.6376457	-1.0475431
H	-5.3619085	-5.0988525	-1.2979107
C	-5.8924710	-0.9428431	0.8294978
C	-5.8570641	-1.4885763	2.2768403
C	-5.9922683	0.5984512	0.8430737
C	-7.1868015	-1.4626476	0.1699062
H	-5.8348394	-2.5839056	2.2668181
H	-4.9789488	-1.1306883	2.8170685
H	-6.7591782	-1.1679485	2.8111184
H	-5.9465053	1.0004528	-0.1750947
H	-6.9506819	0.8948038	1.2828044
H	-5.2124638	1.0731260	1.4426802
H	-8.0440387	-1.0396419	0.7034046
H	-7.2557274	-1.1611986	-0.8811272
H	-7.2708340	-2.5525175	0.2285223
C	2.5425976	5.4062021	1.6478976
C	1.6570284	5.2743856	2.9069501
C	3.8931460	4.6994887	1.8998253
C	2.8154106	6.8982630	1.4002588
H	0.6911560	5.7671076	2.7537568
H	1.4662625	4.2251020	3.1519328
H	2.1520900	5.7408056	3.7666346
H	4.5418403	4.7795471	1.0209970
H	4.4063939	5.1596815	2.7521532
H	3.7520807	3.6372126	2.1222813
H	3.3229556	7.3245140	2.2722260
H	3.4607879	7.0512798	0.5282869
H	1.8865888	7.4582484	1.2464899
C	0.4384671	5.5808420	-3.0425166
C	1.1539418	4.9470845	-4.2560899
C	-1.0900833	5.4782974	-3.2429132
C	0.8195435	7.0683637	-2.9768773
H	2.2411044	5.0092113	-4.1397733
H	0.8849206	3.8924994	-4.3713571
H	0.8733158	5.4725219	-5.1763403

H	-1.6222909	5.9173086	-2.3923746
H	-1.3888129	6.0130104	-4.1519817
H	-1.4100938	4.4362931	-3.3428137
H	0.5149642	7.5630372	-3.9053888
H	0.3185381	7.5767253	-2.1458698
H	1.9008372	7.2033767	-2.8643017
C	3.3621758	-5.1922029	1.8860690
C	2.9348053	-4.7814820	3.3127250
C	2.2513081	-6.0594873	1.2532238
C	4.6394095	-6.0411771	1.9878379
H	3.7076429	-4.1665394	3.7858466
H	2.0042869	-4.2055530	3.2996677
H	2.7749725	-5.6730397	3.9302289
H	2.5288627	-6.3643717	0.2386335
H	2.0887747	-6.9617917	1.8540584
H	1.3036310	-5.5148835	1.1966954
H	4.4380625	-6.9291181	2.5965666
H	4.9785763	-6.3793252	1.0025405
H	5.4557008	-5.4869250	2.4637738
C	6.3208243	-2.0824859	-0.9478444
C	6.1480375	-2.0142542	-2.4815346
C	6.7799716	-0.7024654	-0.4267979
C	7.4186559	-3.1098649	-0.6291013
H	5.8242144	-2.9824989	-2.8776679
H	5.4022089	-1.2653719	-2.7654402
H	7.0986933	-1.7456809	-2.9569093
H	6.9104025	-0.7231629	0.6603354
H	7.7361943	-0.4247631	-0.8853573
H	6.0500355	0.0771023	-0.6661603
H	8.3547401	-2.8018339	-1.1071390
H	7.6008290	-3.1843649	0.4485710
H	7.1614910	-4.1055395	-1.0065825
C	-2.6541516	1.6425271	0.6873364
O	-2.9171972	2.6317231	1.3404769
C	-3.0452976	0.2352089	1.2340554
O	-3.0518622	0.0566375	2.4405056

14c : less stable epoxide isomer of **14**
119
Energy = -2134.938837083

C	-0.8331071	-2.7367633	-0.4721592
H	0.0513923	-2.3949386	-2.4419646
H	-0.7313427	-3.3812211	0.4011897
C	-0.6182796	-1.2827697	-0.2744242
C	0.7684478	-0.9640969	-0.3026425
C	-1.5881371	-0.2971633	-0.0313080
C	1.1138390	0.3942431	-0.2478365
C	-1.1760293	1.0386604	0.0790201
C	0.1637578	1.4064958	-0.0618636
H	2.1490963	0.6787861	-0.3970625
H	-1.9281878	1.7996021	0.2645800
C	1.9411059	-1.9375079	-0.3224263
C	3.0243164	-1.4621758	0.4454303
C	2.1345102	-3.1956579	-0.9987181
C	4.2780938	-2.0581656	0.4577975
H	2.8514146	-0.5959113	1.0696492
C	3.4500234	-3.7252673	-1.1363387
C	4.4793766	-3.1427131	-0.3995394
H	5.4771366	-3.5562949	-0.4815269
C	-3.0283910	-0.6044449	0.1667395
C	-3.6887915	-0.0265488	1.2598563
C	-3.7549882	-1.4145335	-0.7111522
C	-5.0492486	-0.2449578	1.4897319
H	-3.1140008	0.5885132	1.9472234
C	-5.1186276	-1.6559692	-0.5112765
H	-3.2489953	-1.8475822	-1.5658829
C	-5.7453678	-1.0636545	0.5914512
H	-6.8003720	-1.2432376	0.7549253
C	0.5857893	2.8240143	-0.0000437
C	1.8322974	3.1679915	0.5388540
C	-0.2487770	3.8466359	-0.4679414
C	2.2557785	4.4969495	0.6136673
H	2.4673639	2.3800481	0.9330417
C	0.1369876	5.1887516	-0.4053817
H	-1.2055988	3.5806603	-0.9086022
C	1.3929235	5.4921703	0.1366890
H	1.7052340	6.5272227	0.1899732
C	5.4297924	-1.5383458	1.3205927
C	6.5947082	-1.1013799	0.4036883
C	5.0173046	-0.3371002	2.1866912
C	5.9113440	-2.6748516	2.2501318
H	6.9584598	-1.9337267	-0.2066089
H	6.2769502	-0.2975070	-0.2691886
H	7.4294799	-0.7351721	1.0119286
H	4.1935380	-0.5890373	2.8634102
H	5.8697964	-0.0204608	2.7964027
H	4.7128651	0.5171194	1.5712878
H	6.7507808	-2.3267686	2.8625555
H	5.1047166	-2.9972881	2.9170337
H	6.2461789	-3.5433482	1.6738827
C	3.8330297	-4.8554731	-2.1250779
C	3.8036267	-6.2372166	-1.4343314

C	2.9441119	-4.8590321	-3.3890814
C	5.2782382	-4.6297280	-2.6391108
H	4.4781379	-6.2420221	-0.5704384
H	2.7971993	-6.4835222	-1.0951662
H	4.1461208	-7.0053458	-2.1382257
H	2.9126122	-3.8615432	-3.8418554
H	3.3762008	-5.5482035	-4.1226752
H	1.9244830	-5.1916050	-3.1974332
H	5.4764270	-5.3478228	-3.4414339
H	5.4088114	-3.6189153	-3.0403820
H	6.0314559	-4.7976640	-1.8639118
C	-5.7225297	0.4057776	2.7040967
C	-5.0403999	-0.0981452	3.9954912
C	-5.5642078	1.9401013	2.6129647
C	-7.2220517	0.0782387	2.7878127
H	-5.1408597	-1.1851743	4.0861077
H	-3.9734768	0.1467761	4.0029717
H	-5.5042034	0.3679229	4.8727826
H	-6.0372628	2.3237001	1.7025090
H	-6.0361919	2.4195683	3.4784874
H	-4.5090577	2.2312818	2.5965725
H	-7.6519916	0.5712040	3.6664654
H	-7.7622632	0.4342169	1.9035273
H	-7.3943991	-0.9990282	2.8886172
C	-5.8708658	-2.5576088	-1.4972506
C	-5.7401261	-1.9812908	-2.9245778
C	-5.2448007	-3.9701553	-1.4615827
C	-7.3655982	-2.6777440	-1.1598224
H	-6.1717757	-0.9757411	-2.9770125
H	-4.6925605	-1.9194471	-3.2353463
H	-6.2692853	-2.6239326	-3.6379312
H	-5.3409001	-4.4072096	-0.4615444
H	-5.7558317	-4.6256977	-2.1768791
H	-4.1816041	-3.9395705	-1.7168112
H	-7.8521293	-3.3261244	-1.8965952
H	-7.5214731	-3.1196956	-0.1693804
H	-7.8651383	-1.7026753	-1.1865896
C	3.6303477	4.8136277	1.2153289
C	3.6885873	4.2854356	2.6660952
C	4.7234543	4.1120979	0.3780851
C	3.9274131	6.3214760	1.2354247
H	2.9191525	4.7621008	3.2827750
H	3.5317534	3.2026175	2.7034580
H	4.6694017	4.5020469	3.1049538
H	4.7104527	4.4727938	-0.6560466
H	5.7127670	4.3149660	0.8041937
H	4.5763875	3.0272913	0.3611820
H	4.9175169	6.4900650	1.6724663
H	3.9291393	6.7455112	0.2252123
H	3.1948976	6.8679268	1.8396893
C	-0.8130929	6.2702806	-0.9338293
C	-1.0830630	6.0223735	-2.4347579
C	-2.1481620	6.1965614	-0.1593119
C	-0.2373033	7.6862663	-0.7719488
H	-0.1510241	6.0712357	-3.0081780
H	-1.5340636	5.0389290	-2.6015794
H	-1.7702253	6.7829019	-2.8233586
H	-1.9849875	6.3689930	0.9100531
H	-2.8416394	6.9589819	-0.5324659
H	-2.6236766	5.2176461	-0.2771709
H	-0.9567029	8.4158500	-1.1589913
H	-0.0473462	7.9263642	0.2801032
H	0.6977941	7.8076374	-1.3297804
C	-0.1778939	-3.1831645	-1.7277817
O	-1.5705146	-3.4045121	-1.5253721
C	0.9430786	-4.0559411	-1.3454998
O	0.8761051	-5.2522296	-1.0911849

14 : proposed enol product
119
Energy = -2135.004071053

C	-0.0318048	-2.6673094	-0.8174718
H	-1.5573788	-4.6590130	-2.6523874
H	0.9363051	-3.1563605	-0.8713156
C	-0.0100209	-1.2973625	-0.3934026
C	-1.1546779	-0.4429613	-0.3516046
C	1.2791374	-0.7060395	-0.1881920
C	-0.9468770	0.9442182	-0.3906176
C	1.4190869	0.6765911	-0.1708877
C	0.3190385	1.5312060	-0.3351443
H	-1.8125061	1.5939707	-0.4456228
H	2.4152648	1.0919503	-0.0522157
C	-2.5374656	-0.9163956	-0.1135808
C	-3.3211521	-0.0510544	0.6810835
C	-3.1233034	-2.1337548	-0.5646206
C	-4.6287522	-0.3342824	1.0280216
H	-2.8547809	0.8464464	1.0639886
C	-4.4242319	-2.5184180	-0.0908032
C	-5.1368569	-1.5868702	0.6542395
H	-6.1246182	-1.8534766	1.0073766
C	2.5008778	-1.5299382	-0.0161270
C	3.6564160	-1.2465409	-0.7527060

C	2.5349386	-2.5690232	0.9224490
C	4.8328907	-1.9802063	-0.5725666
H	3.6186759	-0.4490313	-1.4898669
C	3.6901048	-3.3281312	1.1259523
H	1.6400248	-2.7701575	1.5049282
C	4.8269198	-3.0168391	0.3689714
H	5.7292134	-3.5962558	0.5164757
C	0.4808610	2.9986659	-0.3983486
C	-0.4231659	3.7846215	-1.1260221
C	1.5353038	3.6352551	0.2705205
C	-0.2962777	5.1743117	-1.1901350
H	-1.2255219	3.2938705	-1.6699242
C	1.6979827	5.0222336	0.2254812
H	2.2216785	3.0348866	0.8606192
C	0.7710219	5.7735410	-0.5086302
H	0.8826245	6.8493595	-0.5504142
C	-5.4919330	0.6188195	1.8562349
C	-5.8472335	-0.0504854	3.2031770
C	-4.7787759	1.9498772	2.1428283
C	-6.7925129	0.9168163	1.0768878
H	-6.3970932	-0.9846867	3.0515191
H	-4.9400022	-0.2755319	3.7740304
H	-6.4749051	0.6214593	3.7993577
H	-4.5039662	2.4656599	1.2156954
H	-5.4490015	2.6058712	2.7079114
H	-3.8721334	1.8034987	2.7399496
H	-7.4308421	1.5884952	1.6617167
H	-6.5685863	1.3978921	0.1186613
H	-7.3573105	0.0010564	0.8755770
C	-5.0462758	-3.9352023	-0.2273576
C	-5.8425340	-4.0940455	-1.5427797
C	-3.9833623	-5.0492841	-0.0811405
C	-6.0525845	-4.1915498	0.9242021
H	-6.6326558	-3.3360953	-1.5956982
H	-5.1973472	-3.9910888	-2.4140010
H	-6.3191890	-5.0817422	-1.5604289
H	-3.4078287	-4.9108143	0.8407727
H	-4.4938674	-6.0163347	-0.0181646
H	-3.2918909	-5.1055661	-0.9199070
H	-6.3598509	-5.2414500	0.8824957
H	-5.6039880	-4.0031342	1.9055631
H	-6.9605547	-3.5878636	0.8309514
C	6.0710109	-1.6341379	-1.4081716
C	5.7389705	-1.8105477	-2.9066046
C	6.4668872	-0.1639498	-1.1454169
C	7.2747853	-2.5276219	-1.0693401

H	5.4563462	-2.8466483	-3.1208190
H	4.9101172	-1.1634431	-3.2098714
H	6.6117146	-1.5556351	-3.5189562
H	6.7081146	-0.0109496	-0.0881246
H	7.3460864	0.1025753	-1.7433634
H	5.6559153	0.5212715	-1.4113860
H	8.1299350	-2.2355223	-1.6881929
H	7.5700077	-2.4257060	-0.0192622
H	7.0624197	-3.5836827	-1.2686513
C	3.6715794	-4.4614296	2.1583929
C	3.3436676	-3.8780198	3.5506239
C	2.5815447	-5.4837306	1.7656026
C	5.0183119	-5.1967907	2.2449144
H	4.1031876	-3.1495890	3.8536522
H	2.3714393	-3.3755886	3.5534279
H	3.3146182	-4.6795237	4.2978612
H	2.7918448	-5.9144849	0.7808735
H	2.5456080	-6.2977146	2.4989646
H	1.5921152	-5.0175436	1.7269357
H	4.9489355	-5.9956631	2.9908953
H	5.2902048	-5.6547450	1.2875275
H	5.8271860	-4.5234608	2.5490187
C	-1.3154968	5.9854174	-1.9989537
C	-1.2728803	5.5298975	-3.4745861
C	-2.7298603	5.7325645	-1.4300481
C	-1.0353847	7.4958596	-1.9484254
H	-0.2792266	5.7007784	-3.9027808
H	-1.5058053	4.4645864	-3.5690633
H	-2.0061469	6.0933956	-4.0631469
H	-2.7864241	6.0458450	-0.3819043
H	-3.4726853	6.2996343	-2.0028884
H	-2.9961217	4.6720613	-1.4833013
H	-1.7934342	8.0244836	-2.5362649
H	-1.0766970	7.8795197	-0.9229468
H	-0.0535776	7.7381224	-2.3701112
C	2.8642322	5.6683419	0.9832930
C	2.7306722	5.3506632	2.4894087
C	4.1966101	5.0879043	0.4585534
C	2.9005557	7.1953641	0.8108851
H	1.7923830	5.7523894	2.8868084
H	2.7429548	4.2713253	2.6717627
H	3.5632974	5.7995793	3.0433359
H	4.3174399	5.3006597	-0.6091503
H	5.0398721	5.5333444	0.9988052
H	4.2404572	4.0026419	0.5968434
H	3.7536798	7.6019776	1.3645489

H	3.0155348	7.4796549	-0.2410264
H	1.9914899	7.6668178	1.2003028
C	-1.0375029	-3.3305674	-1.4502126
O	-0.7389642	-4.4757284	-2.1297049
C	-2.4486228	-2.9514150	-1.5847827
O	-3.0656839	-3.4685351	-2.5393290

Ap : unstable carbene-like adduct of CO and **11**

119

Energy = -2622.176314260

K	3.7132760	2.5331318	3.2000283
C	0.3477516	0.0411768	2.8664313
H	0.6311522	-0.8915235	3.3744380
H	-0.5730142	0.3840557	3.3556115
C	0.0901421	-0.1384558	1.3965049
C	1.1635280	-0.1874346	0.4703232
C	-1.2271337	-0.1182997	0.8775202
C	0.9005795	-0.2211622	-0.9063479
C	-1.4520348	-0.1362150	-0.5039570
C	-0.3980935	-0.1874352	-1.4227670
H	1.7406469	-0.3183056	-1.5884602
H	-2.4780226	-0.1104968	-0.8608873
C	2.5976602	-0.2243255	0.8651471
C	3.5269809	0.5548965	0.1543402
C	3.0796979	-1.0886718	1.8569496
C	4.8976469	0.4982848	0.4226507
H	3.1527128	1.2302860	-0.6104777
C	4.4423102	-1.1627769	2.1674303
H	2.3730240	-1.7113463	2.3940903
C	5.3371428	-0.3603066	1.4440948
H	6.3951659	-0.4177807	1.6662514
C	-2.4249983	-0.0659407	1.7572209
C	-3.4203072	0.8903667	1.5289814
C	-2.6049459	-0.9892264	2.7932042
C	-4.5759326	0.9451209	2.3149048
H	-3.2700330	1.6116831	0.7298345
C	-3.7439449	-0.9648279	3.6022934
H	-1.8376022	-1.7403028	2.9555583
C	-4.7165877	0.0106217	3.3476874
H	-5.6030495	0.0426546	3.9683763
C	-0.6417274	-0.2179042	-2.8823714
C	0.2768681	0.3589260	-3.7701424
C	-1.7919925	-0.8188393	-3.4107463
C	0.0723534	0.3412452	-5.1521543
H	1.1559598	0.8509330	-3.3633156
C	-2.0360316	-0.8502620	-4.7866017
H	-2.4953992	-1.2885803	-2.7288779
C	-1.0910965	-0.2672651	-5.6411362
H	-1.2654316	-0.2865143	-6.7092781
C	5.8631843	1.3715369	-0.3925234
C	5.6754469	1.0753463	-1.8976288
C	5.5506859	2.8621899	-0.1315092
C	7.3347619	1.1151245	-0.0297625
H	5.8779020	0.0204413	-2.1103944
H	4.6553147	1.2985419	-2.2253202
H	6.3642891	1.6884998	-2.4899410
H	5.7564551	3.1227204	0.9147272
H	6.1821234	3.5009453	-0.7593920
H	4.5037003	3.0951695	-0.3515522
H	7.9775201	1.7516983	-0.6473028
H	7.5393840	1.3512090	1.0205185
H	7.6163207	0.0722196	-0.2109434
C	4.9078462	-2.1030505	3.2867345
C	4.3210361	-1.6076143	4.6272347
C	4.4005266	-3.5350092	3.0073255
C	6.4392309	-2.1533558	3.4127225
H	4.7225849	-0.6200631	4.8815538
H	3.2319118	-1.5155920	4.5804862
H	4.5836769	-2.3030718	5.4332728
H	4.7908622	-3.9045212	2.0528356
H	4.7338882	-4.2103145	3.8037417
H	3.3077739	-3.5744729	2.9669365
H	6.7138172	-2.8464463	4.2152570
H	6.9088433	-2.5042582	2.4869243
H	6.8567256	-1.1716040	3.6628375
C	-5.6321767	2.0186759	2.0263534
C	-4.9948349	3.4168078	2.1863803
C	-6.1403013	1.8559431	0.5764437
C	-6.8379934	1.9249082	2.9746525
H	-4.6287691	3.5594233	3.2086593
H	-4.1506229	3.5496158	1.5028662
H	-5.7347015	4.1967927	1.9708038
H	-6.5996381	0.8716464	0.4361779
H	-6.8895588	2.6235759	0.3495896
H	-5.3233565	1.9548802	-0.1450944
H	-7.5611236	2.7083652	2.7229442
H	-7.3457053	0.9581788	2.8866874
H	-6.5410000	2.0658618	4.0196352
C	-3.8819161	-1.9909714	4.7333314
C	-3.8504772	-3.4163402	4.1391593
C	-2.6986692	-1.8224189	5.7125451

C	-5.1910243	-1.8235777	5.5207737
H	-4.6817711	-3.5627561	3.4412528
H	-2.9174073	-3.6018623	3.5984380
H	-3.9343291	-4.1611314	4.9394056
H	-2.7042529	-0.8208674	6.1558102
H	-2.7683592	-2.5598229	6.5209926
H	-1.7386009	-1.9605975	5.2055958
H	-5.2379888	-2.5775347	6.3141344
H	-5.2546414	-0.8366439	5.9920004
H	-6.0681040	-1.9572849	4.8780293
C	1.1111451	0.9924616	-6.0738036
C	1.2347500	2.4920301	-5.7232018
C	2.4807098	0.3093204	-5.8612407
C	0.7349860	0.8711070	-7.5592376
H	0.2756413	3.0020248	-5.8637695
H	1.5448798	2.6326396	-4.6827027
H	1.9807401	2.9695583	-6.3692384
H	2.4228730	-0.7570703	-6.1045766
H	3.2368553	0.7722056	-6.5060020
H	2.8157784	0.4033145	-4.8230160
H	1.5107069	1.3457197	-8.1699007
H	0.6553491	-0.1765116	-7.8702862
H	-0.2161369	1.3699512	-7.7758570
C	-3.3122715	-1.5217622	-5.3087189
C	-3.3209647	-3.0060357	-4.8799030
C	-4.5438808	-0.8122820	-4.7025887
C	-3.4232721	-1.4594942	-6.8405725
H	-2.4565658	-3.5337179	-5.2973168
H	-3.2868166	-3.1060798	-3.7903776
H	-4.2337116	-3.4959644	-5.2388530
H	-4.5632690	0.2437058	-4.9931196
H	-5.4654131	-1.2877664	-5.0582378
H	-4.5342893	-0.8634601	-3.6091256
H	-4.3513269	-1.9479569	-7.1567940
H	-3.4473748	-0.4250970	-7.2011428
H	-2.5883440	-1.9763738	-7.3265485
C	1.4169433	1.1429843	3.2036404
O	1.9277590	0.9938068	4.3412655

Bp : 6-ring of carbene/aryl sites
119
Energy = -2622.191645209

K	4.6907993	2.4523182	2.9125989
C	0.4498259	0.3343065	2.6149816
H	0.0389898	-0.4162585	3.3058808
H	-0.0150417	1.2894001	2.8857080
C	0.1247123	-0.0187224	1.1720930
C	1.2032549	-0.0387744	0.2450671
C	-1.2013637	-0.1014794	0.7147287
C	0.9013759	-0.0573695	-1.1344601
C	-1.4620217	-0.1257296	-0.6614940
C	-0.4142872	-0.0854775	-1.6005394
H	1.7205834	-0.1095010	-1.8468626
H	-2.4940216	-0.1538274	-0.9986473
C	2.5525694	-0.0596393	0.7602566
C	3.6612469	0.4844812	0.1095148
C	2.7099825	-0.6729637	2.1609547
C	4.9664559	0.4338015	0.6031905
H	3.4757640	0.9622612	-0.8528569
C	4.1576608	-0.8906512	2.5490295
H	2.1259373	-1.6109147	2.2146597
C	5.1618306	-0.3425473	1.8030325
H	6.1843429	-0.5369854	2.1151083
C	-2.3479718	-0.0958400	1.6603343
C	-3.3694434	0.8507511	1.5305872
C	-2.4399880	-1.0496304	2.6799322
C	-4.4630144	0.8690072	2.4031590
H	-3.2905991	1.5879628	0.7357661
C	-3.5144057	-1.0627579	3.5730781
H	-1.6618972	-1.8050108	2.7529488
C	-4.5138406	-0.0930037	3.4189841
H	-5.3519445	-0.0895878	4.1041722
C	-0.7013066	-0.1103853	-3.0543158
C	0.1350314	0.5565226	-3.9599059
C	-1.8124113	-0.7992421	-3.5584780
C	-0.1141317	0.5454267	-5.3348650
H	0.9829788	1.1127994	-3.5695547
C	-2.0982243	-0.8298615	-4.9263897
H	-2.4498312	-1.3377521	-2.8627277
C	-1.2365214	-0.1530710	-5.7990530
H	-1.4444128	-0.1686923	-6.8612797
C	6.1412879	0.9451034	-0.2403965
C	6.3321372	0.0298687	-1.4747589
C	5.8773864	2.3822917	-0.7406531
C	7.4632444	0.9617012	0.5479893
H	6.5290857	-0.9995069	-1.1562420
H	5.4262406	0.0251386	-2.0905657
H	7.1708554	0.3700479	-2.0967977
H	5.8138021	3.0879932	0.0982149
H	6.6896783	2.7179100	-1.3974033
H	4.9403166	2.4462416	-1.3019988
H	8.2576572	1.3956309	-0.0699791

H	7.3847259	1.5660926	1.4616461
H	7.7791820	-0.0457504	0.8374107
C	4.4057671	-1.8553317	3.7046337
C	3.4719331	-1.5394522	4.8940321
C	4.1108697	-3.2995037	3.2250297
C	5.8567579	-1.8071094	4.2099123
H	3.6429402	-0.5244522	5.2666616
H	2.4176201	-1.6183106	4.6063104
H	3.6486255	-2.2517801	5.7086528
H	4.8016751	-3.5815671	2.4237867
H	4.2234718	-4.0093414	4.0540445
H	3.0878595	-3.3893848	2.8431288
H	5.9729404	-2.4851646	5.0631268
H	6.5663418	-2.1205061	3.4367231
H	6.1281548	-0.7961994	4.5369860
C	-5.5517622	1.9339831	2.2237520
C	-4.9210690	3.3368357	2.3685090
C	-6.1684565	1.7982852	0.8137364
C	-6.6791073	1.8009049	3.2599069
H	-4.4785011	3.4606365	3.3626744
H	-4.1345714	3.4978651	1.6247860
H	-5.6849726	4.1111058	2.2303327
H	-6.6269217	0.8119360	0.6859481
H	-6.9410777	2.5616514	0.6638497
H	-5.4107984	1.9235108	0.0340459
H	-7.4285410	2.5804651	3.0848720
H	-7.1811087	0.8299669	3.1867635
H	-6.3036450	1.9201998	4.2821355
C	-3.5637951	-2.1289068	4.6743603
C	-3.6062228	-3.5300019	4.0253597
C	-2.2964678	-2.0143424	5.5505471
C	-4.7941724	-1.9773133	5.5828289
H	-4.4994700	-3.6395774	3.4013458
H	-2.7290229	-3.7022000	3.3940423
H	-3.6269316	-4.3056447	4.7999947
H	-2.2378625	-1.0263236	6.0195230
H	-2.3130752	-2.7731884	6.3416108
H	-1.3878341	-2.1634868	4.9585131
H	-4.7764286	-2.7559706	6.3530799
H	-4.8050142	-1.0050869	6.0878112
H	-5.7273065	-2.0845579	5.0191728
C	0.8315729	1.3007712	-6.2772404
C	0.8363748	2.7990655	-5.9006133
C	2.2617560	0.7364793	-6.1250980
C	0.4162624	1.1733846	-7.7517645
H	-0.1679267	3.2248791	-5.9998563
H	1.1675837	2.9476588	-4.8678755
H	1.5155865	3.3515542	-6.5605831
H	2.2880206	-0.3258992	-6.3906642
H	2.9526615	1.2762228	-6.7833802
H	2.6220619	0.8385488	-5.0963235
H	1.1263020	1.7249365	-8.3776290
H	0.4172620	0.1284956	-8.0816189
H	-0.5816161	1.5906397	-7.9266723
C	-3.3275393	-1.6023581	-5.4208277
C	-3.1919263	-3.0885434	-5.0210774
C	-4.5959598	-1.0147703	-4.7626167
C	-3.4947265	-1.5252934	-6.9469478
H	-2.2989113	-3.5304582	-5.4762237
H	-3.1121808	-3.2027480	-3.9353908
H	-4.0695637	-3.6514864	-5.3601988
H	-4.7171942	0.0401136	-5.0318265
H	-5.4836919	-1.5637819	-5.0981487
H	-4.5451002	-1.0826247	-3.6712661
H	-4.3863207	-2.0887872	-7.2429442
H	-3.6214338	-0.4912910	-7.2866770
H	-2.6341559	-1.9581714	-7.4689996
C	1.9333629	0.4000508	2.8935249
O	2.4492095	1.3348155	3.5400956

Cp : stable 6-ring after aryl proton shift to CO
119
Energy = -2622.226294162

K	-0.0523733	-0.8234347	2.9996861
C	-1.0798114	-2.4704738	-0.3939426
H	-1.0829125	-2.8810567	-1.4170126
H	-2.1051859	-2.5131497	-0.0159098
C	-0.5769336	-1.0538903	-0.3892551
C	0.8279529	-0.8813899	-0.3894739
C	-1.4081703	0.0793854	-0.2514699
C	1.3709184	0.4006244	-0.2266358
C	-0.8305274	1.3503833	-0.0906942
C	0.5583269	1.5329749	-0.0697534
H	2.4498409	0.5208358	-0.2073000
H	-1.4857780	2.2080770	0.0352072
C	1.7098025	-2.0669784	-0.5028994
C	3.0035588	-1.9188224	-1.0125725
C	1.2435578	-3.3298997	-0.0645979
C	3.8859021	-2.9957959	-1.0777017
H	3.3150515	-0.9460015	-1.3846408
C	2.1267459	-4.4324792	-0.1146291

H	-0.5861105	-4.3698376	0.4206622
C	3.4231313	-4.2303040	-0.6164866
H	4.0971703	-5.0750046	-0.6598121
C	-2.8896232	-0.0244370	-0.2154746
C	-3.6113482	0.6496978	0.7844938
C	-3.5908327	-0.7667126	-1.1665534
C	-5.0016416	0.5802275	0.8485694
H	-3.0586307	1.2221789	1.5226952
C	-4.9889537	-0.8617221	-1.1341429
H	-3.0321468	-1.2698223	-1.9500264
C	-5.6703945	-0.1847115	-0.1216137
H	-6.7523987	-0.2489010	-0.0775689
C	1.1513021	2.8764822	0.1269778
C	2.3645135	3.0245644	0.8227163
C	0.5192798	4.0193561	-0.3697112
C	2.9384032	4.2781268	1.0217638
H	2.8433946	2.1386733	1.2256253
C	1.0640306	5.2977166	-0.1857668
H	-0.4037855	3.9043686	-0.9309537
C	2.2699898	5.4032295	0.5090771
H	2.7093467	6.3831628	0.6613140
C	5.2968611	-2.7945292	-1.6421191
C	5.1990291	-2.3066372	-3.1045625
C	6.0355986	-1.7278898	-0.8029077
C	6.1281340	-4.0872961	-1.6180161
H	4.6850104	-3.0494792	-3.7243554
H	4.6440474	-1.3652864	-3.1722279
H	6.2013868	-2.1426088	-3.5182726
H	6.1196969	-2.0496918	0.2408339
H	7.0447836	-1.5635869	-1.1994824
H	5.5029335	-0.7713886	-0.8204559
H	7.1253566	-3.8866502	-2.0253129
H	6.2501695	-4.4678922	-0.5977996
H	5.6671692	-4.8733201	-2.2264015
C	1.7246879	-5.8489807	0.3464868
C	0.6527138	-6.4369222	-0.6031969
C	2.9076574	-6.8392185	0.3091020
C	1.2285100	-5.8229166	1.8121447
H	-0.2658895	-5.8466400	-0.6130041
H	1.0416628	-6.4750319	-1.6271395
H	0.4017691	-7.4588052	-0.2920510
H	3.7390306	-6.5004108	0.9370588
H	2.5651551	-7.8056046	0.6964873
H	3.2815859	-7.0024404	-0.7080596
H	0.8048730	-6.8012083	2.0748649
H	2.0809041	-5.6338371	2.4764362
H	0.4969308	-5.0335607	2.0031858
C	-5.8133745	1.2974461	1.9329725
C	-4.9205796	2.0780680	2.9108195
C	-6.7875351	2.2925225	1.2636781
C	-6.6236768	0.2574751	2.7382449
H	-4.2271014	1.4146047	3.4401782
H	-4.3370010	2.8490735	2.3953601
H	-5.5471484	2.5744992	3.6594268
H	-7.4729380	1.7794990	0.5816523
H	-7.3848932	2.8059303	2.0261770
H	-6.2362417	3.0452690	0.6896366
H	-7.2178669	0.7591972	3.5109532
H	-7.3076015	-0.3010582	2.0915667
H	-5.9542297	-0.4595404	3.2258780
C	-5.7066495	-1.7039347	-2.1946360
C	-5.3837251	-1.1431094	-3.5971723
C	-5.2045899	-3.1625090	-2.1029144
C	-7.2323515	-1.7025306	-2.0103949
H	-5.7330746	-0.1089132	-3.6892540
H	-4.3070512	-1.1576080	-3.7927665
H	-5.8789315	-1.7469356	-4.3666044
H	-5.4252870	-3.5851189	-1.1166923
H	-5.6977514	-3.7789904	-2.8635815
H	-4.1234101	-3.2218764	-2.2628151
H	-7.6926164	-2.3180130	-2.7908790
H	-7.5212360	-2.1194318	-1.0391673
H	-7.6469223	-0.6914286	-2.0906303
C	4.2561640	4.4683751	1.7814491
C	4.0108282	5.3697953	3.0119437
C	4.8493861	3.1358661	2.2664940
C	5.2855224	5.1484192	0.8513661
H	3.6231199	6.3496758	2.7159904
H	3.2860457	4.9072753	3.6908733
H	4.9485692	5.5244197	3.5582498
H	5.0665566	2.4631241	1.4290980
H	5.7889464	3.3273469	2.7956314
H	4.1732146	2.6208876	2.9580889
H	6.2306707	5.3019500	1.3850035
H	5.4816118	4.5256882	-0.0282618
H	4.9272849	6.1231794	0.5056359
C	0.3292973	6.5168211	-0.7553453
C	0.2076670	6.3687195	-2.2883886
C	-1.0857289	6.5909859	-0.1392152
C	1.0594824	7.8347201	-0.4517956
H	1.1991210	6.3169940	-2.7511741
H	-0.3421988	5.4618079	-2.5592753

H	-0.3264684	7.2292804	-2.7077363
H	-1.0280262	6.6980820	0.9493903
H	-1.6280560	7.4529002	-0.5449748
H	-1.6649425	5.6892800	-0.3623175
H	0.4915043	8.6698778	-0.8756965
H	1.1533007	8.0042942	0.6266612
H	2.0618224	7.8511002	-0.8938621
C	-0.1792662	-3.3473870	0.5172525
O	-0.1779266	-2.9102162	1.8254023

Dp : salt-like 6-ring after aryl deprotonation
119
Energy = -3221.580270427

K	-4.3026843	-1.8475140	2.1843985
C	-2.3076381	1.0116397	0.9550968
H	-2.4803487	2.0877978	0.9217745
H	-2.5572147	0.6594850	1.9839800
C	-0.8911032	0.6214976	0.6573484
C	-0.6899692	-0.7985279	0.6146404
C	0.2040794	1.4871467	0.4474173
C	0.6273168	-1.2884598	0.5256520
C	1.4903506	0.9563763	0.2817769
C	1.7257876	-0.4400775	0.3619148
H	0.7800148	-2.3634565	0.4670427
H	2.3253312	1.6423847	0.1726961
C	-1.8595673	-1.6311876	0.4854497
C	-1.8117899	-3.0047453	0.7988007
C	-3.0613682	-1.0432560	-0.1986229
C	-2.8052040	-3.8938325	0.4236259
H	-0.9477066	-3.3615832	1.3550017
C	-3.9632006	-2.0459528	-0.8038818
C	-3.8276217	-3.3700223	-0.4393633
H	-4.5371416	-4.0838856	-0.8424754
C	0.0371357	2.9640863	0.3721139
C	-0.6085924	3.6944865	1.3715560
C	0.5406810	3.6536915	-0.7472179
C	-0.7833757	5.0821855	1.2684671
H	-0.9685059	3.1675481	2.2502398
C	0.3874307	5.0337006	-0.8843842
H	1.0624527	3.0782431	-1.5068246
C	-0.2857748	5.7276951	0.1346959
H	-0.4173236	6.7999031	0.0351839
C	3.0870430	-0.9902804	0.1992418
C	3.4379328	-2.2331658	0.7567424
C	4.0691949	-0.2868393	-0.5213806
C	4.7137196	-2.7760077	0.5972621
H	2.6944956	-2.7655948	1.3393900
C	5.3597907	-0.7938777	-0.6911373
H	3.7982435	0.6592365	-0.9756611
C	5.6621604	-2.0399792	-0.1276310
H	6.6614439	-2.4483727	-0.2545860
C	-2.8139310	-5.3775729	0.7843308
C	-2.7339046	-6.2367264	-0.4993771
C	-1.6303216	-5.7573151	1.6876995
C	-4.1219674	-5.7299539	1.5308245
H	-3.5731938	-6.0258309	-1.1704258
H	-1.8058162	-6.0233267	-1.0408814
H	-2.7564586	-7.3061739	-0.2530134
H	-1.6398473	-5.1820104	2.6208819
H	-1.6839202	-6.8220338	1.9430231
H	-0.6725088	-5.5778939	1.1865410
H	-4.1766042	-6.8052241	1.7446378
H	-4.1744086	-5.1935568	2.4873906
H	-5.0005465	-5.4564529	0.9362478
C	-5.1116989	-1.6710705	-1.7574502
C	-4.5622324	-0.8545074	-2.9483001
C	-5.8270925	-2.9007200	-2.3511351
C	-6.1775915	-0.8450369	-0.9997285
H	-4.2280550	0.1173092	-2.5731593
H	-3.7371502	-1.3933951	-3.4306369
H	-5.3499487	-0.6920943	-3.6954255
H	-6.3304475	-3.4988138	-1.5832268
H	-6.5941699	-2.5518143	-3.0523486
H	-5.1355180	-3.5502385	-2.8994470
H	-6.9953031	-0.5660608	-1.6780386
H	-6.6106125	-1.4505839	-0.1908898
H	-5.7305060	0.0675610	-0.5954262
C	-1.5113618	5.8298356	2.3912022
C	-2.9337674	5.2467047	2.5472985
C	-0.7367429	5.6397882	3.7141194
C	-1.6323979	7.3361559	2.1115633
H	-3.5047385	5.3688527	1.6205099
H	-2.9024037	4.1791908	2.7868935
H	-3.4661843	5.7638017	3.3543103
H	0.2750065	6.0516422	3.6313669
H	-1.2552988	6.1545402	4.5315644
H	-0.6531575	4.5804238	3.9758669
H	-2.1608552	7.8180705	2.9413240
H	-0.6483459	7.8091008	2.0192037
H	-2.1979455	7.5312350	1.1935812
C	0.9195766	5.8048430	-2.0976467
C	1.6266643	4.8882224	-3.1088346

C	-0.2560849	6.5047686	-2.8149052
C	1.9302427	6.8714537	-1.6199459
H	2.4848673	4.3772569	-2.6585082
H	0.9455667	4.1292185	-3.5111030
H	1.9934000	5.4869087	-3.9494420
H	-0.7738010	7.1991564	-2.1458204
H	0.1117850	7.0717407	-3.6781094
H	-0.9852788	5.7679321	-3.1703200
H	2.3126828	7.4389697	-2.4764027
H	1.4643531	7.5759913	-0.9238527
H	2.7768968	6.3988048	-1.1104594
C	5.1077720	-4.1345811	1.1901878
C	6.2842304	-3.9441836	2.1729722
C	3.9482003	-4.8029218	1.9463716
C	5.5494571	-5.0773262	0.0486085
H	7.1526550	-3.5046605	1.6720706
H	5.9948418	-3.2826859	2.9968756
H	6.5852711	-4.9109670	2.5937469
H	3.0910450	-4.9846443	1.2882950
H	4.2806719	-5.7691274	2.3414272
H	3.6113896	-4.1911187	2.7905049
H	5.8453990	-6.0512603	0.4562126
H	4.7296300	-5.2335810	-0.6611226
H	6.4010363	-4.6641974	-0.5011907
C	6.4398676	-0.0400153	-1.4777415
C	5.9353242	1.3049335	-2.0257016
C	7.6448625	0.2351605	-0.5509793
C	6.9051325	-0.9065368	-2.6688421
H	5.0964851	1.1687122	-2.7173639
H	5.6130862	1.9723298	-1.2187415
H	6.7449245	1.8016685	-2.5716839
H	8.0667143	-0.6966384	-0.1613196
H	8.4322802	0.7631963	-1.1019602
H	7.3426423	0.8546882	0.3004378
H	7.6874724	-0.3846369	-3.2328077
H	7.3110991	-1.8644146	-2.3284858
H	6.0687336	-1.1121946	-3.3458823
C	-3.2215414	0.3556626	-0.0910834
O	-3.9523390	1.1809985	-0.8108869
K	-1.8587867	1.3148112	-2.3445863

Ep : ketene-like second CO addition
121
Energy = -2735.594447413

K	3.7240118	2.0496377	3.5196540
C	0.4070688	0.1158588	2.8176175
H	1.1613665	-0.5611922	3.2408075
H	-0.4958408	0.0048649	3.4252727
C	0.1234648	-0.1910419	1.3650300
C	1.1904184	-0.2905761	0.4416772
C	-1.1967798	-0.1775486	0.8637619
C	0.9260291	-0.3534168	-0.9315245
C	-1.4275899	-0.2350469	-0.5158009
C	-0.3777623	-0.3143245	-1.4367895
H	1.7629761	-0.4639489	-1.6155248
H	-2.4539975	-0.1961593	-0.8701573
C	2.6223240	-0.3039422	0.8493163
C	3.5166160	0.5791607	0.2240386
C	3.1304835	-1.2331463	1.7673874
C	4.8881328	0.5570895	0.5011220
H	3.1115312	1.3057026	-0.4745814
C	4.4952791	-1.2755770	2.0858084
H	2.4488687	-1.9482841	2.2179793
C	5.3588772	-0.3765827	1.4406028
H	6.4176932	-0.4111449	1.6642901
C	-2.3819602	-0.0690377	1.7550513
C	-3.3022445	0.9662205	1.5686804
C	-2.6199987	-1.0143306	2.7583882
C	-4.4433722	1.0788242	2.3696345
H	-3.1018886	1.7012374	0.7937402
C	-3.7479382	-0.9345353	3.5798923
H	-1.9119329	-1.8295755	2.8815276
C	-4.6452392	0.1202138	3.3696212
H	-5.5222377	0.1959734	3.9997958
C	-0.6298071	-0.3546994	-2.8943106
C	0.2690100	0.2471451	-3.7846933
C	-1.7665845	-0.9863917	-3.4143207
C	0.0560899	0.2269242	-5.1652811
H	1.1345245	0.7641018	-3.3798273
C	-2.0157003	-1.0258527	-4.7892432
H	-2.4534194	-1.4730497	-2.7272689
C	-1.0918613	-0.4154087	-5.6475976
H	-1.2714122	-0.4388129	-6.7148106
C	5.8136082	1.5550308	-0.2090671
C	5.6477427	1.4090466	-1.7385290
C	5.4214019	2.9921007	0.2049643
C	7.2935648	1.3338570	0.1415816
H	5.9044687	0.3938886	-2.0590177
H	4.6193595	1.6153138	-2.0511098
H	6.3076430	2.1146398	-2.2556124
H	5.6149851	3.1533490	1.2734507
H	6.0179243	3.7243635	-0.3504678

H	4.3628713	3.1912538	0.0089042
H	7.9077533	2.0616089	-0.3991620
H	7.4802868	1.4697443	1.2128945
H	7.6279130	0.3304103	-0.1435663
C	4.9933773	-2.2948071	3.1193096
C	4.3104995	-2.0074731	4.4757421
C	4.6222681	-3.7215865	2.6575236
C	6.5158592	-2.2309883	3.3203730
H	4.5922625	-1.0160941	4.8512429
H	3.2198007	-2.0419826	4.3897247
H	4.6191216	-2.7494667	5.2206160
H	5.0908399	-3.9490951	1.6941054
H	4.9690740	-4.4549239	3.3943489
H	3.5398890	-3.8385857	2.5460612
H	6.8141992	-2.9754806	4.0659997
H	7.0540151	-2.4491197	2.3915180
H	6.8350851	-1.2473792	3.6831934
C	-5.4160582	2.2401633	2.1337877
C	-4.6705599	3.5770867	2.3428568
C	-5.9447867	2.1749083	0.6836400
C	-6.6190622	2.2008282	3.0893990
H	-4.2904342	3.6509622	3.3671874
H	-3.8207170	3.6698433	1.6597623
H	-5.3482618	4.4198334	2.1621219
H	-6.4799168	1.2353219	0.5091956
H	-6.6340331	3.0063869	0.4948872
H	-5.1277359	2.2397413	-0.0414762
H	-7.2820586	3.0461050	2.8750601
H	-7.1996662	1.2795695	2.9690154
H	-6.3054035	2.2779518	4.1361819
C	-3.9589918	-1.9907208	4.6713900
C	-4.0562835	-3.3877201	4.0195530
C	-2.7551731	-1.9644830	5.6395437
C	-5.2399378	-1.7455494	5.4845219
H	-4.9068752	-3.4346194	3.3312581
H	-3.1500632	-3.6274717	3.4548319
H	-4.1910882	-4.1560694	4.7900537
H	-2.6630919	-0.9824393	6.1153176
H	-2.8834970	-2.7198112	6.4238056
H	-1.8179649	-2.1747379	5.1147870
H	-5.3407631	-2.5238074	6.2488616
H	-5.2145984	-0.7760185	5.9938509
H	-6.1327031	-1.7801777	4.8505348
C	1.0652587	0.9167678	-6.0914852
C	1.1235944	2.4216230	-5.7459920
C	2.4635044	0.2946918	-5.8794300
C	0.6920168	0.7735901	-7.5756953
H	0.1433935	2.8889061	-5.8890625
H	1.4256156	2.5787057	-4.7054333
H	1.8489593	2.9289115	-6.3927970
H	2.4518633	-0.7740380	-6.1192215
H	3.1972062	0.7879597	-6.5275339
H	2.7969102	0.4069278	-4.8425015
H	1.4477993	1.2751166	-8.1897931
H	0.6526963	-0.2779089	-7.8813921
H	-0.2779254	1.2341836	-7.7934945
C	-3.2737968	-1.7342965	-5.3062343
C	-3.2362096	-3.2184600	-4.8785648
C	-4.5231910	-1.0621301	-4.6941630
C	-3.3932410	-1.6743991	-6.8375507
H	-2.3583378	-3.7198375	-5.3002919
H	-3.1941870	-3.3180402	-3.7892530
H	-4.1354892	-3.7352239	-5.2338021
H	-4.5745814	-0.0067306	-4.9828267
H	-5.4317029	-1.5639256	-5.0471087
H	-4.5079691	-1.1146134	-3.6008484
H	-4.3076008	-2.1902989	-7.1500382
H	-3.4500203	-0.6409818	-7.1972665
H	-2.5453618	-2.1657240	-7.3275960
C	0.9140297	1.5386420	2.9669951
O	1.3004066	2.0154915	4.1566595
C	0.9916950	2.3219791	1.8768847
O	1.1334813	3.1347738	1.0081243

Fp : 7-ring from ketene/aryl sites
121
Energy = -2735.607322623

K	3.5859561	3.6837168	2.5294160
C	0.3545737	0.4114744	2.8581400
H	0.4897121	-0.5364697	3.4008734
H	-0.4886655	0.9266662	3.3250292
C	0.0559121	0.1485562	1.3964583
C	1.1456259	0.1399152	0.4983248
C	-1.2570906	0.0481754	0.8989308
C	0.9007792	0.0737660	-0.8825048
C	-1.4657099	-0.0078263	-0.4870673
C	-0.4004706	0.0129816	-1.3946762
H	1.7486057	0.0141236	-1.5589761
H	-2.4864207	-0.0510558	-0.8561838
C	2.5270737	0.1803933	0.9821756
C	3.5564927	0.8188870	0.2450719
C	2.9590678	-0.6514459	2.1781757

C	4.8903017	0.5490395	0.4653057	H	2.6736577	-3.4281632	2.3453458
H	3.2569559	1.5369848	-0.5146880	H	6.2445857	-3.8382471	3.3485553
C	4.3195553	-1.2492185	2.0498141	H	6.3799389	-3.1364204	1.7262995
H	2.1949454	-1.3709302	2.4749004	H	6.7410146	-2.1458004	3.1572208
C	5.2314441	-0.5858425	1.3004014	C	-5.8483910	1.8206215	1.9256235
H	6.2574200	-0.9399009	1.2705082	C	-5.3581986	3.2857498	1.9312593
C	-2.4460720	0.0557414	1.7878938	C	-6.3545822	1.4616131	0.5105008
C	-3.5366770	0.8829042	1.4933658	C	-7.0262238	1.6997859	2.9058430
C	-2.5198186	-0.7765900	2.9101190	H	-4.9932736	3.5662917	2.9252191
C	-4.6826669	0.8947796	2.2937835	H	-4.5436349	3.4350080	1.2154678
H	-3.4692393	1.5383511	0.6291244	H	-6.1800292	3.9588326	1.6596520
C	-3.6451514	-0.7889235	3.7388951	H	-6.7102496	0.4260205	0.4789460
H	-1.6853760	-1.4383032	3.1228696	H	-7.1825091	2.1222926	0.2273875
C	-4.7151263	0.0543243	3.4137973	H	-5.5605848	1.5710018	-0.2350495
H	-5.5931804	0.0564124	4.0470843	H	-7.8289811	2.3766228	2.5933891
C	-0.6379351	-0.0571377	-2.8545800	H	-7.4319366	0.6821258	2.9246304
C	0.2211549	0.5956918	-3.7484012	H	-6.7321903	1.9741496	3.9250070
C	-1.7197912	-0.7790654	-3.3752239	C	-3.6672448	-1.7144347	4.9612269
C	0.0232636	0.5371958	-5.1304200	C	-3.5155318	-3.1797656	4.4962489
H	1.0445436	1.1786421	-3.3450348	C	-2.4872946	-1.3525782	5.8909499
C	-1.9544689	-0.8567909	-4.7508489	C	-4.9716821	-1.5939492	5.7652553
H	-2.3733184	-1.3072738	-2.6864773	H	-4.3418235	-3.4622447	3.8350054
C	-1.0705263	-0.1939351	-5.6121923	H	-2.5784303	-3.3291144	3.9505371
H	-1.2376633	-0.2477947	-6.6803326	H	-3.5173074	-3.8521538	5.3621833
C	6.0191715	1.2848384	-0.2532666	H	-2.5721443	-0.3169903	6.2375805
C	6.7581902	0.3086949	-1.2001442	H	-2.4790279	-2.0131501	6.7658344
C	5.5099092	2.4656398	-1.0941065	H	-1.5277107	-1.4588674	5.3747540
C	7.0246435	1.8234729	0.7891843	H	-4.9316533	-2.2711300	6.6254358
H	7.1809705	-0.5380564	-0.6509157	H	-5.1186049	-0.5764826	6.1442913
H	6.0700880	-0.0845680	-1.9561610	H	-5.8438512	-1.8678567	5.1612868
H	7.5789898	0.8263865	-1.7106180	C	0.9910952	1.2791487	-6.0606760
H	4.9655190	3.1945495	-0.4810510	C	0.9431935	2.7902473	-5.7423633
H	6.3576011	2.9831821	-1.5558057	C	2.4265611	0.7580833	-5.8262923
H	4.8421431	2.1324710	-1.8959646	C	0.6431143	1.0847137	-7.5452520
H	7.8662626	2.3164068	0.2880299	H	-0.0655768	3.1862434	-5.9015924
H	6.5430684	2.5496837	1.4540214	H	1.2245913	2.9859033	-4.7026081
H	7.4185335	1.0152070	1.4124780	H	1.6376264	3.3349016	-6.3928646
C	4.6085512	-2.5109251	2.8470045	H	2.4900041	-0.3129145	-6.0472710
C	4.2494823	-2.2672607	4.3313092	H	3.1313572	1.2890991	-6.4767265
C	3.7409884	-3.6703364	2.3025924	H	2.7409424	0.9098675	-4.7885799
C	6.0841548	-2.9277739	2.7604556	H	1.3680465	1.6270672	-8.1619393
H	4.8393819	-1.4395188	4.7375062	H	0.6802200	0.0279117	-7.8321289
H	3.1901146	-2.0154087	4.4496596	H	-0.3545037	1.4722612	-7.7796886
H	4.4505572	-3.1697018	4.9202642	C	-3.1528688	-1.6641570	-5.2648815
H	3.9999750	-3.8873452	1.2609652	C	-3.0111472	-3.1342583	-4.8114178
H	3.9046622	-4.5751575	2.8999004	C	-4.4533794	-1.0752768	-4.6737135

C	-3.2633031	-1.6391347	-6.7978455
H	-2.0950009	-3.5768246	-5.2172404
H	-2.9722316	-3.2124023	-3.7202725
H	-3.8668312	-3.7213418	-5.1651584
H	-4.5799855	-0.0317732	-4.9819631
H	-5.3197745	-1.6488436	-5.0233794
H	-4.4426611	-1.1070568	-3.5795114
H	-4.1346627	-2.2260506	-7.1080754
H	-3.3924001	-0.6189096	-7.1761061
H	-2.3774350	-2.0751187	-7.2728309
C	1.5953044	1.2659083	3.0957128
O	1.4513505	2.4428266	3.5230620
C	2.9302302	0.6446469	3.0230730
O	3.9831491	1.2714577	3.3176580

Gp : 7-ring after slow aryl proton shift to CO

121

Energy = -2735.624899709

K	0.4149293	-4.7312841	4.3813395
C	0.4497568	-2.4166995	0.0263532
H	1.4257886	-2.7310919	-0.3453139
H	-0.3246734	-2.7994633	-0.6555287
C	0.3259407	-0.9136310	0.0946817
C	-0.9577445	-0.3427939	0.2548228
C	1.4480744	-0.0672102	-0.0318739
C	-1.1015601	1.0485548	0.2334455
C	1.2603930	1.3211232	-0.0642489
C	-0.0062326	1.9024445	0.0562437
H	-2.0877811	1.4723227	0.3990713
H	2.1340155	1.9564075	-0.1799788
C	-2.1674638	-1.1926788	0.3999686
C	-3.2805928	-0.8210812	-0.3698624
C	-2.2056736	-2.3229809	1.2577965
C	-4.4609455	-1.5486838	-0.3287280
H	-3.1872270	0.0463948	-1.0146015
C	-3.3733567	-3.1412016	1.2291946
H	-0.5876483	-1.4488616	2.2755131
C	-4.4589716	-2.7051131	0.4581438
H	-5.3572689	-3.3103634	0.4549430
C	2.8406542	-0.5781194	-0.1113147
C	3.7196432	-0.0796822	-1.0797973
C	3.3217336	-1.5056106	0.8212064
C	5.0568195	-0.4857000	-1.1301507
H	3.3397466	0.6324888	-1.8076876
C	4.6517154	-1.9350992	0.8038742
H	2.6460382	-1.8793629	1.5833167
C	5.5016796	-1.4129356	-0.1798209
H	6.5351071	-1.7341089	-0.2044780
C	-0.1869443	3.3712795	0.0151232
C	-1.3492274	3.9257642	-0.5374817
C	0.7941296	4.2363148	0.5172532
C	-1.5462986	5.3080220	-0.5941295
H	-2.0985980	3.2574586	-0.9524755
C	0.6353861	5.6246394	0.4752793
H	1.6846167	3.8093553	0.9702777
C	-0.5415337	6.1403135	-0.0835097
H	-0.6787401	7.2133441	-0.1212804
C	-5.7119828	-1.1625180	-1.1229848
C	-6.8993370	-0.9901837	-0.1501042
C	-5.5229574	0.1537323	-1.8945985
C	-6.0447649	-2.2811155	-2.1350438
H	-7.0975653	-1.9138406	0.4028868
H	-6.6910144	-0.1969288	0.5761807
H	-7.8064051	-0.7231021	-0.7050835
H	-4.7172848	0.0768415	-2.6329926
H	-6.4468698	0.3999068	-2.4290903
H	-5.2925898	0.9845250	-1.2177682
H	-6.9467771	-2.0224547	-2.7022604
H	-5.2186471	-2.4200853	-2.8409675
H	-6.2226783	-3.2343146	-1.6268589
C	-3.5375016	-4.4974199	1.9550501
C	-2.2655664	-5.3511003	1.7897932
C	-3.8534564	-4.2763795	3.4513061
C	-4.6940886	-5.3352743	1.3641481
H	-2.0443437	-5.5157852	0.7285579
H	-1.4177491	-4.8584767	2.2656900
H	-2.4167960	-6.3307233	2.2612983
H	-4.7969900	-3.7271167	3.5570314
H	-3.9672002	-5.2470775	3.9530289
H	-3.0387431	-3.7126343	3.9118694
H	-4.6751325	-6.3240219	1.8361537
H	-5.6776622	-4.8986980	1.5674301
H	-4.5892195	-5.4721236	0.2817000
C	5.9793775	0.0930840	-2.2096062
C	5.4185011	-0.2635391	-3.6041974
C	6.0322297	1.6304072	-2.0623692
C	7.4130979	-0.4520046	-2.1098919
H	5.3747255	-1.3501659	-3.7359188
H	4.4091956	0.1374271	-3.7414036
H	6.0616461	0.1553131	-4.3870843
H	6.4264763	1.9105538	-1.0795177

H	6.6822994	2.0605313	-2.8332137
H	5.0375910	2.0747278	-2.1693235
H	8.0264438	-0.0051263	-2.8998488
H	7.8722237	-0.2047668	-1.1462623
H	7.4402785	-1.5398832	-2.2376135
C	5.1262760	-2.9720301	1.8299279
C	4.7104562	-2.5407859	3.2533721
C	4.4612608	-4.3280726	1.5007462
C	6.6529794	-3.1541401	1.8124967
H	5.1492062	-1.5708747	3.5114380
H	3.6232033	-2.4577235	3.3480979
H	5.0574391	-3.2828722	3.9819866
H	4.7604362	-4.6683850	0.5029757
H	4.7720888	-5.0865654	2.2302899
H	3.3699931	-4.2466541	1.5227700
H	6.9413595	-3.8718234	2.5883957
H	7.0029821	-3.5455564	0.8512063
H	7.1723662	-2.2100490	2.0118408
C	-2.8341715	5.8587398	-1.2187821
C	-2.9192588	5.4039388	-2.6929345
C	-4.0528051	5.3041636	-0.4476156
C	-2.8943045	7.3941620	-1.1799303
H	-2.0662892	5.7847951	-3.2649256
H	-2.9217963	4.3120647	-2.7725241
H	-3.8411702	5.7815338	-3.1504035
H	-4.0187757	5.6118336	0.6030918
H	-4.9817123	5.6819917	-0.8901663
H	-4.0812332	4.2101499	-0.4807505
H	-3.8341596	7.7314826	-1.6300347
H	-2.8577688	7.7727993	-0.1524196
H	-2.0703242	7.8442041	-1.7446473
C	1.7375731	6.5259015	1.0454598
C	1.9325706	6.2063780	2.5444657
C	3.0589236	6.2521473	0.2928354
C	1.4029333	8.0198817	0.9080055
H	1.0085249	6.3927324	3.1023343
H	2.2156918	5.1597637	2.6952594
H	2.7246878	6.8378902	2.9634392
H	2.9490587	6.4743384	-0.7741514
H	3.8601269	6.8812073	0.6976485
H	3.3649447	5.2057221	0.3931010
H	2.2229253	8.6145727	1.3248601
H	1.2762105	8.3097936	-0.1410240
H	0.4883006	8.2797293	1.4523670
C	0.1841106	-3.0904870	1.3689364
O	0.9074156	-3.9931362	1.7883973
C	-0.9693795	-2.5002825	2.2028045
O	-1.1307533	-3.0435242	3.4258107

HCO : radical after H-atom transfer to CO
3
Energy = -113.9234881189

H	-0.9227822	0.3440677	-0.0000032
C	0.0092137	-0.2861682	0.0000076
O	1.1136037	0.1420993	-0.0000043

Hp : stable 7-ring after aryl deprotonation
121
Energy = -3335.021977309

K	1.8594930	2.8299505	5.0081871
C	0.5886786	-0.5476641	2.7232744
H	1.3892815	-1.2791975	2.8805321
H	-0.2515272	-0.7746151	3.3783497
C	0.1973487	-0.4977981	1.2694934
C	1.2502049	-0.3686676	0.3279542
C	-1.1388515	-0.3566302	0.8195037
C	0.9579047	-0.1399973	-1.0199702
C	-1.3906763	-0.0858457	-0.5330114
C	-0.3617641	0.0176209	-1.4784240
H	1.7822430	-0.0687264	-1.7241060
H	-2.4232531	0.0407724	-0.8484203
C	2.6749489	-0.4486439	0.7445845
C	3.5004095	-1.2713130	-0.0301286
C	3.1995900	0.3183780	1.8309777
C	4.8655919	-1.3833462	0.2131065
H	3.0416173	-1.8339794	-0.8398440
C	4.5918044	0.1552522	2.1251245
K	0.3835369	2.9866900	1.2769578
C	5.3701309	-0.6699373	1.3041327
H	6.4204890	-0.7799875	1.5382719
C	-2.3010212	-0.4537943	1.7393288
C	-3.2714102	0.5536582	1.7474064
C	-2.4699059	-1.5511061	2.5905799
C	-4.3801791	0.4986648	2.5951696
H	-3.1335591	1.4081278	1.0891056
C	-3.5647195	-1.6428375	3.4543901
H	-1.7297944	-2.3457910	2.5633170
C	-4.5074326	-0.6071002	3.4446372
H	-5.3564993	-0.6619125	4.1139961
C	-0.6520696	0.2833658	-2.9033183
C	0.2638600	0.9914408	-3.7040279
C	-1.8387088	-0.1666027	-3.4915648

C	0.0112596	1.2426474	-5.0502532
H	1.1746923	1.3597179	-3.2450065
C	-2.1304214	0.0740042	-4.8399520
H	-2.5350133	-0.7404142	-2.8865452
C	-1.1948154	0.7769324	-5.6008894
H	-1.3990201	0.9701640	-6.6484615
C	5.7350990	-2.2771711	-0.6764957
C	5.2321884	-3.7352490	-0.5891274
C	5.6295572	-1.7958826	-2.1410752
C	7.2162055	-2.2509701	-0.2657396
H	5.3056054	-4.1045509	0.4398882
H	4.1858001	-3.8098247	-0.9022322
H	5.8323532	-4.3870371	-1.2363934
H	5.9844797	-0.7633901	-2.2320433
H	6.2357713	-2.4325608	-2.7975402
H	4.5935587	-1.8285102	-2.4934243
H	7.7947877	-2.8916157	-0.9411260
H	7.6299974	-1.2378598	-0.3221721
H	7.3569184	-2.6230772	0.7552556
C	5.2919899	0.7909500	3.3488350
C	4.4308086	0.5765402	4.6128535
C	6.6572646	0.1391631	3.6589314
C	5.5642279	2.2928123	3.1096179
H	3.3782645	0.7876146	4.4068569
H	4.4866648	-0.4712836	4.9306639
H	4.7979691	1.2030926	5.4362728
H	7.4053212	0.3514292	2.8872218
H	7.0323473	0.5558702	4.6014817
H	6.5737921	-0.9476078	3.7748467
H	6.0547054	2.7268969	3.9926676
H	6.2387189	2.4110658	2.2525472
H	4.6204623	2.8049682	2.9015167
C	-5.3939870	1.6491611	2.5805976
C	-4.6729696	2.9647590	2.9503335
C	-5.9970459	1.7817677	1.1647231
C	-6.5428654	1.4310998	3.5779522
H	-4.2235298	2.8922227	3.9460779
H	-3.8743525	3.1921455	2.2372267
H	-5.3830041	3.8006382	2.9461034
H	-6.5202677	0.8625945	0.8803150
H	-6.7124972	2.6122739	1.1326866
H	-5.2197713	1.9730726	0.4181267
H	-7.2371830	2.2770214	3.5257590
H	-7.1057667	0.5193071	3.3502337
H	-6.1742842	1.3616262	4.6072230
C	-3.6883309	-2.8581824	4.3815212
C	-3.7579516	-4.1462991	3.5322098
C	-2.4465000	-2.9206186	5.2987376
C	-4.9420847	-2.7948857	5.2683871
H	-4.6342920	-4.1289390	2.8754305
H	-2.8676056	-4.2577510	2.9059646
H	-3.8296998	-5.0258648	4.1832725
H	-2.3731447	-2.0149371	5.9100043
H	-2.5118951	-3.7878134	5.9668490
H	-1.5251027	-3.0082148	4.7149165
H	-4.9804474	-3.6828104	5.9090144
H	-4.9319543	-1.9124624	5.9175659
H	-5.8590795	-2.7728474	4.6691847
C	0.9954650	2.0084702	-5.9426921
C	0.3047982	3.2709705	-6.5038866
C	2.2575534	2.4448253	-5.1818147
C	1.4270313	1.1021920	-7.1171477
H	-0.5822217	3.0119965	-7.0902336
H	-0.0086614	3.9334661	-5.6899783
H	0.9941746	3.8227163	-7.1540501
H	2.8040437	1.5842310	-4.7809598
H	2.9271881	2.9810908	-5.8631789
H	2.0153546	3.1167318	-4.3511226
H	2.1245984	1.6377622	-7.7721501
H	1.9239338	0.1997814	-6.7455224
H	0.5664096	0.7918450	-7.7178339
C	-3.4462415	-0.4503992	-5.4278583
C	-3.4893978	-1.9885157	-5.2912640
C	-4.6314692	0.1614560	-4.6484744
C	-3.6078853	-0.0921146	-6.9136448
H	-2.6596585	-2.4481251	-5.8387451
H	-3.4162429	-2.2975596	-4.2441252
H	-4.4307836	-2.3778072	-5.6969703
H	-4.6259059	1.2535422	-4.7319549
H	-5.5813063	-0.2121865	-5.0491058
H	-4.5852321	-0.0973643	-3.5862032
H	-4.5622562	-0.4844716	-7.2814610
H	-3.6080805	0.9924315	-7.0688519
H	-2.8086047	-0.5288222	-7.5225005
C	1.0483724	0.8844812	2.9940808
O	0.1576999	1.7160824	3.5179708
C	2.2906838	1.2978405	2.5014509
O	2.6546367	2.5983467	2.5721156

K·**11**H : K-atom adduct of arene BzptH
118
Energy = -2509.380523014

K	0.6477411	-0.5420676	-1.6362288
C	1.1159440	-1.1347068	2.1166761
H	1.7012233	-0.2084395	2.0748319
H	0.0526231	-0.8743654	2.1746201
C	1.3880331	-1.9922244	0.9039687
C	2.6435219	-1.9320967	0.2038889
C	0.3748201	-2.8410624	0.3383415
C	2.7885396	-2.5269835	-1.0466505
C	0.5520704	-3.4227238	-0.9140601
C	1.7354804	-3.2424216	-1.6981036
H	3.7703585	-2.4816298	-1.5072658
H	-0.2290766	-4.0878105	-1.2684207
C	3.8281869	-1.2570137	0.7807594
C	4.6497635	-0.4398032	-0.0137826
C	4.1853092	-1.4343151	2.1269637
C	5.7964867	0.1737676	0.4932275
H	4.3720835	-0.2882747	-1.0546689
C	5.3195417	-0.8311587	2.6735223
H	3.5682547	-2.0822554	2.7417495
C	6.1155002	-0.0267362	1.8442668
H	6.9941416	0.4521856	2.2571826
C	-0.8838626	-3.1411679	1.0584524
C	-2.1157720	-3.1385322	0.3833622
C	-0.8844400	-3.4638515	2.4245552
C	-3.3128328	-3.4588101	1.0264173
H	-2.1230653	-2.8757831	-0.6721449
C	-2.0612474	-3.7821628	3.1050564
H	0.0664273	-3.4880165	2.9479846
C	-3.2697382	-3.7732869	2.3924240
H	-4.1903103	-4.0118825	2.9096178
C	1.8480619	-3.7270366	-3.0635535
C	3.0209160	-3.5260142	-3.8363957
C	0.7694807	-4.3773567	-3.7170888
C	3.1224854	-3.9357150	-5.1627260
H	3.8751293	-3.0359261	-3.3793574
C	0.8411733	-4.7976656	-5.0420684
H	-0.1499838	-4.5532205	-3.1672582
C	2.0247609	-4.5734543	-5.7657764
H	2.0914734	-4.8943870	-6.7965017
C	6.6551679	1.0429576	-0.4348870
C	7.1552398	0.1845494	-1.6183894
C	5.8016148	2.2082357	-0.9823692
C	7.8785056	1.6366463	0.2820992
H	7.7631480	-0.6520143	-1.2574152
H	6.3191141	-0.2286186	-2.1915335
H	7.7665642	0.7928676	-2.2957878
H	5.4309720	2.8331008	-0.1624200
H	6.4005713	2.8346912	-1.6541983
H	4.9380477	1.8362962	-1.5436370
H	8.4570941	2.2415174	-0.4249375
H	7.5819706	2.2831489	1.1155319
H	8.5360106	0.8515337	0.6714164
C	5.6584040	-1.0750991	4.1500511
C	4.4876255	-0.5829253	5.0295738
C	5.8642204	-2.5882855	4.3827203
C	6.9352749	-0.3410751	4.5901509
H	4.3257658	0.4914490	4.8888040
H	3.5571620	-1.1011525	4.7763507
H	4.7053657	-0.7654482	6.0887960
H	6.6885529	-2.9634113	3.7664135
H	6.1015372	-2.7810308	5.4359133
H	4.9635415	-3.1548030	4.1260537
H	7.1283233	-0.5491202	5.6484082
H	7.8078400	-0.6727817	4.0164590
H	6.8367275	0.7438373	4.4723355
C	-4.6211813	-3.4480106	0.2249440
C	-4.8565340	-2.0352896	-0.3540414
C	-4.5139497	-4.4587815	-0.9389546
C	-5.8388964	-3.8267502	1.0833075
H	-4.9339746	-1.2967757	0.4514308
H	-4.0344771	-1.7357016	-1.0121880
H	-5.7854878	-2.0137234	-0.9365404
H	-4.3454158	-5.4709442	-0.5556701
H	-5.4393974	-4.4586469	-1.5273823
H	-3.6842689	-4.2084943	-1.6077262
H	-6.7418258	-3.8082566	0.4628703
H	-5.7376400	-4.8342920	1.5016636
H	-5.9831767	-3.1225772	1.9102495
C	-1.9908018	-4.1357152	4.5965359
C	-1.0752859	-5.3653613	4.7865557
C	-1.3998369	-2.9400422	5.3761130
C	-3.3702806	-4.4650959	5.1893864
H	-1.4715737	-6.2278854	4.2396754
H	-0.0633145	-5.1668154	4.4195261
H	-1.0087257	-5.6277656	5.8492409
H	-2.0369891	-2.0557013	5.2653691
H	-1.3245260	-3.1822144	6.4430485
H	-0.4000013	-2.6846849	5.0107788
H	-3.2609920	-4.7093220	6.2519128
H	-4.0569263	-3.6151204	5.1078221
H	-3.8269518	-5.3270347	4.6903670
C	4.4335137	-3.6800001	-5.9221169

C	4.7355037	-2.1650114	-5.9375495
C	5.5896418	-4.4170855	-5.2108804
C	4.3743201	-4.1705869	-7.3778715
H	3.9285531	-1.6166773	-6.4359260
H	4.8365988	-1.7705594	-4.9213401
H	5.6716915	-1.9679860	-6.4746139
H	5.4014359	-5.4956750	-5.1885251
H	6.5361416	-4.2400452	-5.7368169
H	5.7018614	-4.0736563	-4.1777723
H	5.3294265	-3.9623160	-7.8733980
H	4.1959722	-5.2500354	-7.4302185
H	3.5835978	-3.6631755	-7.9412831
C	-0.3763493	-5.4949862	-5.6681983
C	-0.6891046	-6.7859039	-4.8797919
C	-1.6009849	-4.5556223	-5.6027689
C	-0.1433301	-5.8747444	-7.1395225
H	0.1633617	-7.4725292	-4.9129576
H	-0.9032818	-6.5657559	-3.8293764
H	-1.5615607	-7.2939656	-5.3095318
H	-1.4052618	-3.6289111	-6.1532721
H	-2.4792941	-5.0423718	-6.0446671
H	-1.8429575	-4.2909814	-4.5684132
H	-1.0395740	-6.3640778	-7.5376058
H	0.0611973	-4.9918188	-7.7548456
H	0.6958766	-6.5705475	-7.2460143
H	1.3656624	-1.6080359	3.0807266

K^+·**11**H : K^+ adduct of arene BzptH
118
Energy = -2509.286839498

K	0.9837105	-0.7123818	-1.1307378
C	1.4661935	-2.0097110	2.5106682
H	2.1300502	-1.1467659	2.6091644
H	0.4300694	-1.6966133	2.6633137
C	1.6362476	-2.6703449	1.1618775
C	2.8603107	-2.5735732	0.4507272
C	0.5385311	-3.2997247	0.5192091
C	2.9639510	-3.1010039	-0.8467309
C	0.6741978	-3.7949480	-0.7875248
C	1.8772575	-3.6978257	-1.5005335
H	3.9248477	-3.0442946	-1.3486263
H	-0.1952410	-4.2410958	-1.2615218
C	4.0126804	-1.7816158	0.9538568
C	4.5604709	-0.8155922	0.0980534
C	4.5445348	-1.9217642	2.2384065
C	5.5874885	0.0364856	0.5065583
H	4.1654328	-0.7284657	-0.9126651
C	5.5884454	-1.1006237	2.6808992
H	4.1461411	-2.6895737	2.8940792
C	6.0836467	-0.1235433	1.8067238
H	6.8756604	0.5301428	2.1482682
C	-0.8208556	-3.3133191	1.1191036
C	-1.8852470	-2.8051390	0.3638391
C	-1.0732779	-3.7840306	2.4104705
C	-3.1789277	-2.7158370	0.8822769
H	-1.6933407	-2.4655367	-0.6527678
C	-2.3602672	-3.7366213	2.9576571
H	-0.2548220	-4.2026056	2.9878084
C	-3.3917695	-3.1880801	2.1832866
H	-4.3862537	-3.1263186	2.6056552
C	1.9684085	-4.1174570	-2.9164644
C	2.7936028	-3.4055275	-3.7975565
C	1.1983019	-5.1745364	-3.4151062
C	2.8520371	-3.7171525	-5.1577882
H	3.3892683	-2.5805541	-3.4146835
C	1.2364362	-5.5227701	-4.7689183
H	0.5784737	-5.7453206	-2.7295652
C	2.0647693	-4.7792448	-5.6208115
H	2.0961157	-5.0333652	-6.6723098
C	6.1320588	1.0827576	-0.4716752
C	6.9328919	0.3558150	-1.5764116
C	4.9616155	1.8523324	-1.1260599
C	7.0527476	2.0995869	0.2225374
H	7.7812632	-0.1870675	-1.1475374
H	6.3025525	-0.3661104	-2.1081999
H	7.3150592	1.0798440	-2.3046667
H	4.3092818	2.3016117	-0.3693792
H	5.3505603	2.6499744	-1.7672358
H	4.3552496	1.1954561	-1.7613431
H	7.3857558	2.8447273	-0.5070706
H	6.5326761	2.6235376	1.0319584
H	7.9450218	1.6187158	0.6365955
C	6.1345136	-1.2842196	4.1009820
C	4.9887336	-1.0606939	5.1139109
C	6.6753225	-2.7231631	4.2542386
C	7.2687373	-0.2987318	4.4241803
H	4.5809498	-0.0482292	5.0222567
H	4.1726410	-1.7739281	4.9578929
H	5.3618735	-1.1927867	6.1354083
H	7.4902556	-2.9087075	3.5465652
H	7.0576556	-2.8710313	5.2702875
H	5.8914011	-3.4662138	4.0756878

H 7.6202174 -0.4761055 5.4457892
H 8.1212307 -0.4291695 3.7486185
H 6.9305276 0.7415152 4.3619804
C -4.2938109 -2.1186607 0.0176659
C -3.8475204 -0.7398293 -0.5207035
C -4.5606339 -3.0642666 -1.1753982
C -5.6016058 -1.9330449 0.8034365
H -3.5903748 -0.0613335 0.2997535
H -2.9761818 -0.8294367 -1.1804145
H -4.6544127 -0.2871771 -1.1066700
H -4.8889249 -4.0479367 -0.8242962
H -5.3414849 -2.6467155 -1.8206241
H -3.6571875 -3.2024165 -1.7796307
H -6.3553833 -1.4799664 0.1513863
H -5.9986751 -2.8905996 1.1565696
H -5.4607431 -1.2748522 1.6678217
C -2.5910576 -4.2686938 4.3760975
C -2.2129616 -5.7661778 4.4215059
C -1.6945512 -3.4864269 5.3622920
C -4.0530905 -4.1205928 4.8261503
H -2.8375166 -6.3442576 3.7321874
H -1.1650713 -5.9232305 4.1460829
H -2.3603579 -6.1575037 5.4342520
H -1.9381012 -2.4186807 5.3452176
H -1.8455494 -3.8598866 6.3811164
H -0.6338318 -3.5998055 5.1155908
H -4.1591130 -4.5146259 5.8421227
H -4.3678620 -3.0712131 4.8386586
H -4.7341373 -4.6808364 4.1762406
C 3.7571614 -2.8909279 -6.0782147
C 3.3621928 -1.3998553 -5.9805827
C 5.2245111 -3.0562205 -5.6226079
C 3.6475660 -3.3266299 -7.5479504
H 2.3160587 -1.2522664 -6.2696639
H 3.4950363 -1.0177658 -4.9620020
H 3.9935979 -0.8001819 -6.6450044
H 5.5350411 -4.1041228 -5.6886736
H 5.8851309 -2.4575124 -6.2594491
H 5.3604320 -2.7270908 -4.5865977
H 4.3055319 -2.7019807 -8.1607586
H 3.9548078 -4.3692707 -7.6823473
H 2.6258083 -3.2132534 -7.9266594
C 0.3794015 -6.6905338 -5.2700089
C 0.7942923 -7.9802044 -4.5270576
C -1.1076081 -6.3896197 -4.9753085
C 0.5372846 -6.9258499 -6.7805997
H 1.8461372 -8.2168420 -4.7193112
H 0.6585716 -7.8805065 -3.4454120
H 0.1814668 -8.8213518 -4.8698176
H -1.4277273 -5.4762074 -5.4882016
H -1.7330920 -7.2191737 -5.3229627
H -1.2842123 -6.2604134 -3.9025164
H -0.0961770 -7.7661801 -7.0826556
H 0.2301890 -6.0485863 -7.3606438
H 1.5713159 -7.1733001 -7.0445232
H 1.7131397 -2.6928564 3.3312806

K_4H_4 : salt-like KH tetramer
8
Energy = -2402.228685172
K -1.9262792 -0.2577155 -1.3770249
K 1.9272874 0.2630892 -1.3757827
K 0.2598799 -1.9259206 1.3729917
K -0.2605854 1.9279253 1.3752717
H -0.2443347 1.8150306 -1.2924096
H 0.2452276 -1.8094062 -1.2945718
H -1.8122254 -0.2432959 1.2905236
H 1.8118478 0.2455783 1.2918092

TS1p : TS of 6-ring from ketene/aryl sites
119
Energy = -2622.150611913
K 4.8919468 1.9617335 0.7920420
C 1.4419043 0.1119203 2.2155920
H 1.4068142 -0.7514613 2.9018282
H 1.0391799 0.9439422 2.8091061
C 0.5334601 -0.0907337 1.0014255
C 0.9943038 -0.2391919 -0.3364521
C -0.8664427 -0.0390644 1.2148296
C 0.0576162 -0.3200445 -1.3814411
C -1.7630575 -0.0935948 0.1446511
C -1.3184095 -0.2354918 -1.1760474
H 0.4303957 -0.4975487 -2.3861746
H -2.8260719 -0.0135276 0.3545370
C 2.4236307 -0.3133421 -0.6901297
C 2.9158378 0.2044875 -1.8760258
C 3.3476033 -0.7541442 0.3165202
C 4.3002548 0.2076327 -2.1823249
H 2.2182401 0.6506324 -2.5808085
C 4.7118949 -0.9743605 -0.0546730
H 2.9326836 -1.4393842 1.0558147
C 5.1551051 -0.4270669 -1.2678960

H	6.2066317	-0.5264408	-1.5230728
C	-1.4507182	0.1297687	2.5745970
C	-2.2959146	1.2134154	2.8354999
C	-1.2121089	-0.7978550	3.5936373
C	-2.8917185	1.3910598	4.0887338
H	-2.4740019	1.9305950	2.0383910
C	-1.7869587	-0.6542355	4.8594534
H	-0.5786367	-1.6537891	3.3804095
C	-2.6209022	0.4480313	5.0869266
H	-3.0710865	0.5729210	6.0635000
C	-2.2653280	-0.3076687	-2.3114294
C	-1.9039827	0.1892616	-3.5709658
C	-3.5377892	-0.8730779	-2.1579190
C	-2.7727964	0.1235589	-4.6628887
H	-0.9301419	0.6567947	-3.6856194
C	-4.4388219	-0.9494749	-3.2239984
H	-3.8144382	-1.2792028	-1.1889246
C	-4.0367684	-0.4490511	-4.4691769
H	-4.7225454	-0.5050680	-5.3048578
C	4.7820313	0.8231442	-3.4982316
C	4.1010680	0.1108492	-4.6902906
C	4.4063016	2.3227200	-3.5378536
C	6.3043412	0.7051417	-3.6743832
H	4.3568381	-0.9540827	-4.6971740
H	3.0109154	0.1971406	-4.6335125
H	4.4275457	0.5540277	-5.6389575
H	4.9137341	2.8682575	-2.7328806
H	4.7035198	2.7702681	-4.4942726
H	3.3273882	2.4635662	-3.4153253
H	6.6029534	1.1767996	-4.6171786
H	6.8427536	1.2058582	-2.8611034
H	6.6251484	-0.3420232	-3.7047014
C	5.6894329	-1.7350024	0.8531397
C	6.6614612	-0.7504230	1.5466222
C	4.9501969	-2.5255591	1.9479250
C	6.5186884	-2.7364245	0.0202541
H	7.1872651	-0.1334407	0.8062808
H	6.1154246	-0.1059102	2.2456932
H	7.4207659	-1.2945374	2.1217763
H	4.2555442	-3.2513154	1.5093394
H	5.6778749	-3.0786115	2.5531393
H	4.3935938	-1.8589084	2.6136652
H	7.1860021	-3.3076983	0.6770316
H	5.8603814	-3.4384748	-0.5024751
H	7.1385553	-2.2309107	-0.7271202
C	-3.8076666	2.5982449	4.3226555
C	-3.0070934	3.8987146	4.0896598
C	-4.9863214	2.5426075	3.3254345
C	-4.3810953	2.6313631	5.7481652
H	-2.1645961	3.9637207	4.7863769
H	-2.6086894	3.9434516	3.0714936
H	-3.6515963	4.7724500	4.2424682
H	-5.5724779	1.6289558	3.4708760
H	-5.6473127	3.4051451	3.4715148
H	-4.6327470	2.5572027	2.2898987
H	-5.0279755	3.5082443	5.8604854
H	-4.9824694	1.7409077	5.9614295
H	-3.5878359	2.7012010	6.5004515
C	-1.4830011	-1.6941993	5.9446866
C	-1.9326654	-3.0896925	5.4587352
C	0.0387494	-1.7144243	6.2108401
C	-2.2023082	-1.3884226	7.2680381
H	-3.0100073	-3.1022948	5.2624874
H	-1.4171646	-3.3763292	4.5369486
H	-1.7104242	-3.8451597	6.2215837
H	0.3823127	-0.7364150	6.5639256
H	0.2798734	-2.4635011	6.9743480
H	0.5990741	-1.9599051	5.3032770
H	-1.9509188	-2.1581091	8.0058354
H	-1.8980097	-0.4191986	7.6780622
H	-3.2907703	-1.3849966	7.1438830
C	-2.3216680	0.6865054	-6.0165349
C	-2.0149307	2.1931231	-5.8647103
C	-1.0403580	-0.0467498	-6.4726392
C	-3.3904406	0.5165567	-7.1081702
H	-2.9081682	2.7380385	-5.5407648
H	-1.2258509	2.3651038	-5.1256484
H	-1.6820071	2.6084017	-6.8231601
H	-1.2297292	-1.1191422	-6.5910219
H	-0.6988742	0.3538619	-7.4341838
H	-0.2307721	0.0768993	-5.7459543
H	-3.0146081	0.9280539	-8.0512334
H	-3.6323715	-0.5391690	-7.2736834
H	-4.3142451	1.0477246	-6.8536235
C	-5.8187760	-1.5798841	-2.9989552
C	-5.6430106	-3.0435005	-2.5365853
C	-6.5658951	-0.7882692	-1.9023012
C	-6.6809784	-1.5747357	-4.2715172
H	-5.1160465	-3.6283016	-3.2983137
H	-5.0687097	-3.1008334	-1.6065261
H	-6.6227591	-3.5034511	-2.3617564
H	-6.7048820	0.2548827	-2.2062348

H	-7.5518612	-1.2325403	-1.7220781
H	-6.0109417	-0.7956216	-0.9588029
H	-7.6527353	-2.0305264	-4.0527187
H	-6.8605194	-0.5558795	-4.6324542
H	-6.2130968	-2.1504170	-5.0778226
C	2.8955585	0.4954775	1.9278830
O	3.6747623	0.5867331	2.9018092

TS2p : TS of 1,2-H-shift of aryl proton to CO
119
Energy = -2622.150388287

K	-5.6203926	0.6326510	1.1986563
C	-1.9064747	1.6057229	0.8296088
H	-1.7019851	2.6762322	0.8273805
H	-2.2199139	1.3362067	1.8599305
C	-0.7063434	0.7739171	0.4705981
C	-0.9400540	-0.6209817	0.3622773
C	0.6099209	1.2755572	0.4105081
C	0.1622659	-1.4938923	0.3443762
C	1.6807985	0.3753469	0.3919005
C	1.4737141	-1.0155913	0.3957261
H	-0.0044988	-2.5585948	0.2103870
H	2.6929695	0.7683464	0.3598520
C	-2.3020510	-1.0706373	0.1113636
C	-2.6875865	-2.4099339	0.2162838
C	-3.2289951	-0.0873737	-0.4718156
C	-3.9002834	-2.8915415	-0.2673001
H	-1.9942365	-3.0871533	0.7068192
C	-4.3614057	-0.6436286	-1.2407418
H	-2.4820995	0.7780874	-1.2351389
C	-4.6538892	-1.9890899	-1.0508932
H	-5.5150353	-2.3884045	-1.5785564
C	0.8878065	2.7299111	0.3349757
C	1.9260637	3.3155097	1.0676727
C	0.1369966	3.5385206	-0.5280006
C	2.2165669	4.6795920	0.9598363
H	2.5037956	2.6885323	1.7419854
C	0.3849177	4.9069513	-0.6507125
H	-0.6457784	3.0714044	-1.1193942
C	1.4301013	5.4593423	0.1019843
H	1.6384175	6.5183058	0.0147666
C	2.6181141	-1.9556518	0.3773312
C	2.5177024	-3.2105799	0.9931838
C	3.8235200	-1.6192888	-0.2526833
C	3.5777878	-4.1204059	0.9845150
H	1.5936330	-3.4638436	1.5057828
C	4.9091898	-2.4989681	-0.2760492
H	3.8980107	-0.6598331	-0.7565685
C	4.7671628	-3.7455232	0.3466239
H	5.5986894	-4.4383723	0.3336476
C	-4.3889154	-4.3268237	-0.0680957
C	-4.5564328	-5.0321034	-1.4332597
C	-3.4088177	-5.1532674	0.7799979
C	-5.7562970	-4.3071119	0.6527573
H	-5.2809832	-4.5080780	-2.0656086
H	-3.6001374	-5.0610074	-1.9667899
H	-4.9103787	-6.0616185	-1.2948949
H	-3.2601015	-4.7054004	1.7691499
H	-3.8040041	-6.1657517	0.9207313
H	-2.4312564	-5.2386241	0.2918089
H	-6.1387917	-5.3262277	0.7927703
H	-5.6631140	-3.8341230	1.6374517
H	-6.4951801	-3.7424715	0.0731271
C	-5.0845953	0.1328560	-2.3734499
C	-4.0962435	1.0660934	-3.1046601
C	-5.6299507	-0.8494676	-3.4356494
C	-6.2877221	0.9750629	-1.8885934
H	-3.7666944	1.8837723	-2.4575254
H	-3.2248385	0.5055719	-3.4624738
H	-4.5959748	1.5071635	-3.9753481
H	-6.4541162	-1.4606231	-3.0531764
H	-6.0183711	-0.2741059	-4.2836486
H	-4.8433136	-1.5189733	-3.7985065
H	-6.7966617	1.4339457	-2.7455639
H	-7.0245794	0.3423443	-1.3747587
H	-5.9415142	1.7782170	-1.2326055
C	3.3710687	5.2691949	1.7796100
C	3.0965707	5.0481784	3.2836854
C	4.6842897	4.5519922	1.3939676
C	3.5542355	6.7760180	1.5372946
H	2.1674704	5.5454203	3.5832066
H	3.0032404	3.9831101	3.5182096
H	3.9187455	5.4590278	3.8816818
H	4.9010205	4.6909958	0.3292974
H	5.5211658	4.9567946	1.9753197
H	4.6215843	3.4767574	1.5895462
H	4.3942157	7.1408748	2.1385458
H	3.7741332	6.9910626	0.4856686
H	2.6618710	7.3420845	1.8267808
C	-0.4995003	5.7455134	-1.5811731
C	-0.4863303	5.1382867	-3.0013241

C	-1.9468749	5.7266059	-1.0379669
C	-0.0295510	7.2058064	-1.6766273
H	0.5315930	5.1198875	-3.4057897
H	-0.8725895	4.1143835	-3.0022039
H	-1.1150745	5.7377717	-3.6702073
H	-1.9835762	6.1596648	-0.0320389
H	-2.6019313	6.3154657	-1.6917291
H	-2.3424090	4.7071094	-0.9833625
H	-0.6859340	7.7551518	-2.3604797
H	-0.0666304	7.7062196	-0.7025831
H	0.9942453	7.2744530	-2.0615165
C	3.4082696	-5.4790091	1.6760178
C	3.1251243	-5.2549119	3.1780944
C	2.2150877	-6.2283908	1.0420070
C	4.6583041	-6.3636731	1.5451667
H	3.9572741	-4.7245038	3.6531704
H	2.2165638	-4.6631806	3.3266657
H	2.9924971	-6.2176799	3.6857620
H	2.3889212	-6.3987511	-0.0257201
H	2.0765934	-7.2001886	1.5304568
H	1.2859327	-5.6596618	1.1475170
H	4.4814955	-7.3201270	2.0490581
H	4.8941416	-6.5743093	0.4963029
H	5.5337436	-5.8966615	2.0095019
C	6.2008733	-2.0793005	-0.9889727
C	5.8961223	-1.8148939	-2.4803356
C	6.7416938	-0.7835286	-0.3444787
C	7.2950372	-3.1553742	-0.9016938
H	5.5106685	-2.7197918	-2.9618279
H	5.1498100	-1.0235113	-2.5996741
H	6.8088243	-1.5053137	-3.0032116
H	6.9674092	-0.9439679	0.7152068
H	7.6607118	-0.4648585	-0.8503334
H	6.0141656	0.0308490	-0.4168980
H	8.1945316	-2.8023384	-1.4177199
H	7.5661713	-3.3708446	0.1376934
H	6.9799919	-4.0902453	-1.3780199
C	-3.1047778	1.3273911	-0.0580249
O	-4.0037976	2.2225155	-0.2181342

TS3p : TS of slow 1,2-H-shift
121
Energy = -2735.552395733

K	-1.6534034	-5.9737290	1.8928024
C	-0.3667071	-2.4042242	-1.1521975
H	0.4901921	-2.9432262	-1.5591001
H	-1.1331414	-2.2800570	-1.9364804
C	-0.0044814	-1.0258955	-0.6749119
C	-1.0874202	-0.1370525	-0.5580464
C	1.3147676	-0.5702312	-0.4598967
C	-0.8443899	1.2158084	-0.2816735
C	1.5158682	0.7888436	-0.1830397
C	0.4553463	1.7016268	-0.1024928
H	-1.6927741	1.8808345	-0.1518574
H	2.5328470	1.1403526	-0.0354117
C	-2.4892330	-0.5829671	-0.6936386
C	-3.3377301	0.2387111	-1.4442755
C	-3.0556156	-1.6691355	0.1158091
C	-4.7220776	0.1337738	-1.4015409
H	-2.8597737	0.9877127	-2.0685429
C	-4.4795559	-1.5053900	0.4499132
H	-2.2191063	-1.7388536	1.1468880
C	-5.2328603	-0.6865234	-0.3771949
H	-6.3001027	-0.6365884	-0.1868681
C	2.5062034	-1.4512072	-0.5457724
C	3.6727617	-0.9891640	-1.1708110
C	2.5170708	-2.7255688	0.0362337
C	4.8373320	-1.7602652	-1.2109917
H	3.6569321	-0.0099493	-1.6418507
C	3.6624885	-3.5273944	0.0114498
H	1.6188534	-3.0926425	0.5199815
C	4.8119263	-3.0261709	-0.6125827
H	5.7068318	-3.6349523	-0.6342405
C	0.6987542	3.1335058	0.1850245
C	-0.1460670	4.1168743	-0.3459339
C	1.7763029	3.5395179	0.9824427
C	0.0611399	5.4753976	-0.0948895
H	-0.9672857	3.8069263	-0.9860452
C	2.0220852	4.8891298	1.2497630
H	2.4189350	2.7798338	1.4185243
C	1.1525380	5.8420851	0.7035252
H	1.3284066	6.8911229	0.9042832
C	-5.6658602	0.9448538	-2.2868594
C	-6.5154503	1.8998572	-1.4171754
C	-4.8999013	1.7862010	-3.3203983
C	-6.6087901	-0.0182863	-3.0414233
H	-7.1171527	1.3462620	-0.6892342
H	-5.8696777	2.5925638	-0.8666685
H	-7.1964299	2.4847175	-2.0472905
H	-4.2726522	1.1568188	-3.9614434
H	-5.6105478	2.3231767	-3.9581812
H	-4.2575782	2.5299613	-2.8353521

H	-7.3118798	0.5447360	-3.6671846
H	-6.0331002	-0.6923433	-3.6849822
H	-7.1881894	-0.6294617	-2.3411998
C	-5.1598179	-2.0364393	1.7377388
C	-5.7613138	-3.4511018	1.5754261
C	-4.1684363	-1.9783696	2.9220208
C	-6.3354720	-1.1111721	2.1458810
H	-6.5282018	-3.4371680	0.7909852
H	-4.9846870	-4.1678134	1.3115882
H	-6.2440312	-3.7580933	2.5133504
H	-3.8042230	-0.9525476	3.0584308
H	-4.6842815	-2.2762459	3.8432043
H	-3.3253738	-2.6559396	2.7694930
H	-6.6894102	-1.4185054	3.1366851
H	-6.0237218	-0.0623779	2.1983864
H	-7.1874741	-1.1871221	1.4622969
C	6.0877978	-1.2004505	-1.9007812
C	5.7683766	-0.9161343	-3.3853765
C	6.5027054	0.1183316	-1.2102868
C	7.2766529	-2.1726363	-1.8387591
H	5.4729244	-1.8373505	-3.8994617
H	4.9521635	-0.1937429	-3.4848046
H	6.6512713	-0.5051954	-3.8894542
H	6.7326315	-0.0552800	-0.1532550
H	7.3929579	0.5348886	-1.6962081
H	5.7039545	0.8646905	-1.2659409
H	8.1401614	-1.7220900	-2.3404110
H	7.5641731	-2.3920353	-0.8043445
H	7.0500270	-3.1185926	-2.3430361
C	3.6147909	-4.9308470	0.6290632
C	2.9967636	-4.8706093	2.0431103
C	2.7250452	-5.8252786	-0.2648994
C	5.0095364	-5.5683062	0.7398262
H	3.5779067	-4.2101140	2.6962242
H	1.9660711	-4.5048726	2.0104920
H	2.9874089	-5.8740117	2.4858822
H	3.1455708	-5.8965368	-1.2744430
H	2.6658323	-6.8377440	0.1552352
H	1.7135854	-5.4125632	-0.3337805
H	4.9245184	-6.5457293	1.2279916
H	5.4619042	-5.7274229	-0.2451672
H	5.6883974	-4.9475985	1.3358809
C	-0.8961189	6.5063999	-0.7055783
C	-0.8457804	6.3986795	-2.2458973
C	-2.3349672	6.2131250	-0.2249934
C	-0.5376279	7.9472443	-0.3079040
H	0.1654354	6.6024442	-2.6143047
H	-1.1353574	5.3985307	-2.5838983
H	-1.5327672	7.1241011	-2.6972056
H	-2.3990711	6.2788534	0.8665215
H	-3.0323963	6.9394631	-0.6583405
H	-2.6579854	5.2107368	-0.5238380
H	-1.2555624	8.6382616	-0.7627973
H	-0.5770826	8.0868160	0.7781633
H	0.4633427	8.2243697	-0.6565814
C	3.2170523	5.2753417	2.1302955
C	3.0521921	4.6324106	3.5253465
C	4.5176042	4.7510411	1.4812374
C	3.3459065	6.7964518	2.3102275
H	2.1355906	4.9881576	4.0082831
H	2.9986491	3.5412522	3.4569248
H	3.9048939	4.8932665	4.1631393
H	4.6587837	5.1921361	0.4885918
H	5.3815918	5.0120762	2.1035025
H	4.4953818	3.6623592	1.3686687
H	4.2139439	7.0146817	2.9417197
H	3.4911231	7.3046937	1.3505231
H	2.4607115	7.2211946	2.7966031
C	-0.9700504	-3.2475815	-0.0533609
O	-0.4077430	-4.3228430	0.2784791
C	-2.3269882	-2.9689559	0.4667584
O	-2.9084353	-3.9052995	1.1235346

References

1. Xu, M.; Jupp, A. R.; Ong, M. S. E.; Burton, K. I.; Chitnis, S. S.; Stephan, D. W., Synthesis of Urea Derivatives from CO2 and Silylamines. *Angewandte Chemie International Edition* **2019,** *58* (17), 5707-5711.

2. Kundu, S.; Sinhababu, S.; Siddiqui, M. M.; Luebben, A. V.; Dittrich, B.; Yang, T.; Frenking, G.; Roesky, H. W., Comparison of Two Phosphinidenes Binding to Silicon(IV)dichloride as well as to Silylene. *J. Am. Chem. Soc.* **2018,** *140* (30), 9409-9412.

3. Bader, S. L.; Luescher, M. U.; Gademann, K., Synthesis of maculalactone A and derivatives for environmental fate tracking studies. *Organic & Biomolecular Chemistry* **2015,** *13* (1), 199-206.

4. Ionic and Organometallic-Catalyzed Organosilane Reductions. In *Organic Reactions*, pp 1-737.

5. Liu, L.; Zhang, Y.; Xin, B., Synthesis of Biaryls and Polyaryls by Ligand-Free Suzuki Reaction in Aqueous Phase. *The Journal of Organic Chemistry* **2006,** *71* (10), 3994-3997.

6. *TURBOMOLE V7.0, 2015, a development of University of Karlsruhe and Forschungszentrum Karlsruhe GmbH, 1989-2007, TURBOMOLE GmbH, since 2007; available from http://www.turbomole.com.*

7. Tao, J.; Perdew, J. P.; Staroverov, V. N.; Scuseria, G. E., Climbing the Density Functional Ladder: Nonempirical Meta--Generalized Gradient Approximation Designed for Molecules and Solids. *Physical Review Letters* **2003,** *91* (14), 146401.

8. Grimme, S.; Antony, J.; Ehrlich, S.; Krieg, H., A consistent and accurate ab initio parametrization of density functional dispersion correction (DFT-D) for the 94 elements H-Pu. *The Journal of Chemical Physics* **2010,** *132* (15), 154104-154119.

9. Grimme, S.; Ehrlich, S.; Goerigk, L., Effect of the damping function in dispersion corrected density functional theory. *Journal of Computational Chemistry* **2011,** *32* (7), 1456-1465.

10. Weigend, F.; Häser, M.; Patzelt, H.; Ahlrichs, R., RI-MP2: optimized auxiliary basis sets and demonstration of efficiency. *Chemical Physics Letters* **1998,** *294* (1), 143-152.

11. Weigend, F.; Ahlrichs, R., Balanced basis sets of split valence, triple zeta valence and quadruple zeta valence quality for H to Rn: Design and assessment of accuracy. *Physical Chemistry Chemical Physics* **2005,** *7* (18), 3297-3305.

12. Klamt, A.; Schüürmann, G., COSMO: a new approach to dielectric screening in solvents with explicit expressions for the screening energy and its gradient. *Journal of the Chemical Society, Perkin Transactions 2* **1993,** (5), 799-805.

13. Eichkorn, K.; Weigend, F.; Treutler, O.; Ahlrichs, R., Auxiliary basis sets for main row atoms and transition metals and their use to approximate Coulomb potentials. *Theoretical Chemistry Accounts* **1997,** *97* (1), 119-124.

14. Weigend, F., Accurate Coulomb-fitting basis sets for H to Rn. *Physical chemistry chemical physics* **2006,** *8* (9), 1057-1065.

15. Deglmann, P.; May, K.; Furche, F.; Ahlrichs, R., Nuclear second analytical derivative calculations using auxiliary basis set expansions. *Chemical Physics Letters* **2004,** *384* (1-3), 103-107.
16. Grimme, S., Supramolecular Binding Thermodynamics by Dispersion-Corrected Density Functional Theory. *Chem. Eur. J.* **2012,** *18* (32), 9955-9964.
17. Eckert, F.; Klamt, A., Fast solvent screening via quantum chemistry: COSMO-RS approach. *AIChE Journal* **2002,** *48* (2), 369-385.
18. Eckert, F.; Klamt, A. *COSMOtherm, Version C3.0, Release 16.01; COSMOlogic GmbH & Co. KG, Leverkusen, Germany* 2015.
19. Zhao, Y.; Truhlar, D. G., Design of Density Functionals That Are Broadly Accurate for Thermochemistry, Thermochemical Kinetics, and Nonbonded Interactions. *The Journal of Physical Chemistry A* **2005,** *109* (25), 5656-5667.
20. Weigend, F.; Furche, F.; Ahlrichs, R., Gaussian basis sets of quadruple zeta valence quality for atoms H-Kr. *The Journal of Chemical Physics* **2003,** *119* (24), 12753-12762.
